# Supplementary material for: Ruminant inner ear shape records 35 million years of neutral evolution
Source: Nat Commun. 2022 Dec 6;13:7222. doi: 10.1038/s41467-022-34656-0 (PMC9726890; doi:10.1038/s41467-022-34656-0)
Supplement: Supplementary file 1 — Supplementary Information [file 41467_2022_34656_MOESM1_ESM.pdf]

Supplementary Figures

Ruminant inner ear shape records 35 million years of neutral evolution

Bastien Mennecart[1]*, Laura Dziomber[2,3], Manuela Aiglstorfer[4], Faysal Bibi[5], Daniel DeMiguel[6,7,8], Masaki Fujita[9], Mugino O. Kubo[10], Flavie Laurens[11], Jin Meng[12], Grégoire Métais[13], Bert Müller[14], María Ríos[15], Gertrud E. Rössner[16,17], Israel M. Sánchez[8], Georg Schulz[14,18], Shiqi Wang[19], & Loïc Costeur[1]

[1]Naturhistorisches Museum Basel, Augustinergasse 2, 4001 Basel, Switzerland; emails: mennecartbastien@gmail.com, loic.costeur@bs.ch
[2]Institute of Plant Sciences, University of Bern, 3013 Bern, Switzerland; email: laura.dziomber@ips.unibe.ch
[3]Oeschger Centre for Climate Change Research, University of Bern, 3012 Bern, Switzerland
[4]Naturhistorisches Museum Mainz / Landessammlung für Naturkunde Rheinland-Pfalz, Reichklarastraße 10, 55116 Mainz, Germany; email: maiglstorfer@gmail.com
[5]Museum für Naturkunde, Leibniz Institute for Evolution and Biodiversity Science, Berlin 10115, Germany; email: faysal.bibi@mfn.berlin
[6]Fundación ARAID, Zaragoza, Spain; email: demiguel@unizar.es
[7]Departamento de Ciencias de la Tierra, Área de Paleontología / Instituto Universitario de Investigación en Ciencias Ambientales de Aragón (IUCA). Universidad de Zaragoza, Pedro Cerbuna 12, 50009, Zaragoza, Spain.
[8]Institut Català de Palaeontologia Miquel Crusafont (ICP), Edifici Z, c/de les columnes s/n, Universitat Autònoma de Barcelona, 08193, Cerdanyola del Vallès, Barcelona, Spain, emails: daniel.demiguel@icp.cat, micromeryx@gmail.com
[9]National Museum of Nature and Science, Tsukuba, Japan; email: masaki_fujita@kahaku.go.jp
[10]Department of Natural Environmental Studies, Graduate School of Frontier Sciences, The University of Tokyo, Chiba, Japan; email: mugino@k.u-tokyo.ac.jp
[11]Swiss National Data and Service Center for the Humanities, 4123 Allschwil, Switzerland; email: flavie.laurens@unibas.ch
[12]American Museum of Natural History, 10024 New York, USA; email: jmeng@amnh.org
[13]CR2P - Centre de Recherches sur la Paléobiodiversité et les Paléoenvironnements, UMR 7207, Muséum National d'Histoire Naturelle, CNRS, UPMC, Sorbonne Universités. MNHN, CP38, 8 rue Buffon, 75005 Paris, France; email: metais@mnhn.fr
[14]Biomaterials Science Center, Department of Biomedical Engineering, University of Basel Gewerbestrasse 14, 4123, Allschwil, Switzerland; emails: bert.mueller@unibas.ch, georg.schulz@unibas.ch
[15]Department of Earth Sciences, GeoBioTec , Nova School of Science and Technology, Universidade NOVA de Lisboa, Campus de Caparica, 2829-516, Caparica, Portugal; email: maria.rios.iba@fct.unl.pt
[16]Staatliche Naturwissenschaftliche Sammlungen Bayerns - Bayerische Staatssammlung für Paläontologie und Geologie, Richard-Wagner-Strasse 10, 80333 Munich, Germany; email: roessner@snsb.de
[17]Department für Geo- und Umweltwissenschaften, Paläontologie & Geobiologie, Ludwig-Maximilians-Universität München, Richard-Wagner-Strasse 10, 80333 Munich, Germany.
[18]Micro- and Nanotomography Core Facility, Department of Biomedical Engineering, University of Basel Gewerbestrasse 14, 4123, Allschwil, Switzerland.
[19]Institute of Vertebrate Paleontology and Paleoanthropology, Chinese Academy of Sciences, 142 Xizhimenwai Street, Beijing, 100044, China; email: wangshiqi@ivpp.ac.cn
*corresponding author

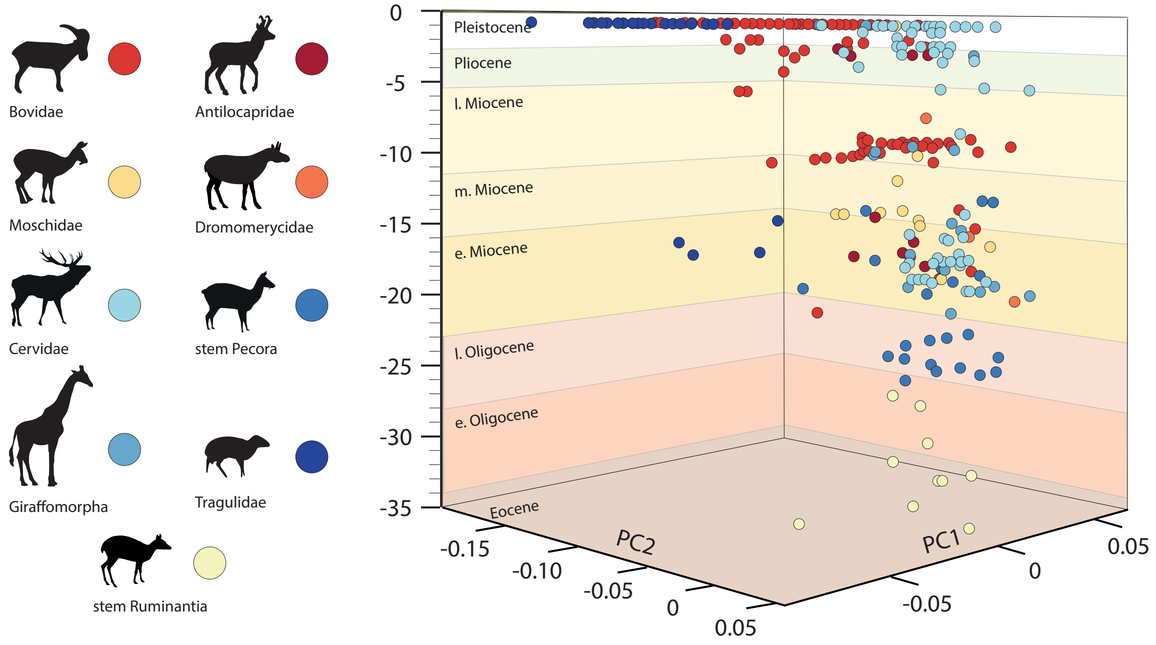

Supplementary Figure 1. Evolution of the ruminant BL morphology through time. Shape changes are observed along PC axes (PC1 21.99% & PC2 10.60%). Groups are Stem Ruminantia (light yellow), Tragulidae (dark blue), Stem Pecora (light dark blue), Antilocapridae (dark red), Dromomerycidae (orange), Giraffomorpha (dark light blue), Cervidae (light blue), Moschidae (yellow), Bovidae (red). Bivariate plot of PC1 vs PC2 and animated gif of the Supplementary Figure 1 can be found in Supplementary Material 1-1. Silhouettes of the families modified from[22].

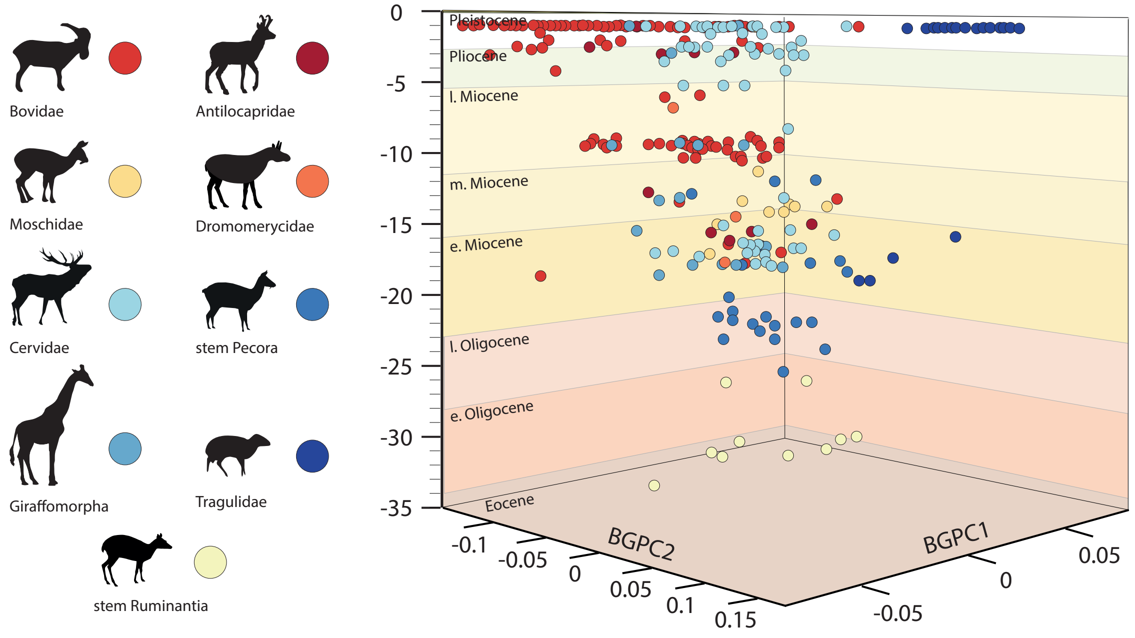

Supplementary Figure 2. Evolution of the ruminant BL morphology through time. Shape changes are observed along bg-PC axes (bg-PC 1 70.92% & bg-PC 2 12.85%). Groups are Stem Ruminantia (light yellow), Tragulidae (dark blue), Stem Pecora (light dark blue), Antilocapridae (dark red), Dromomerycidae (orange), Giraffomorpha (dark light blue), Cervidae (light blue), Moschidae (yellow), Bovidae (red). Bivariate plot of bg-PC1 vs bg-PC2 and animated gif of the Supplementary Figure 2 can be found in Supplementary Material 1-1. Silhouettes of the families modified from[22].

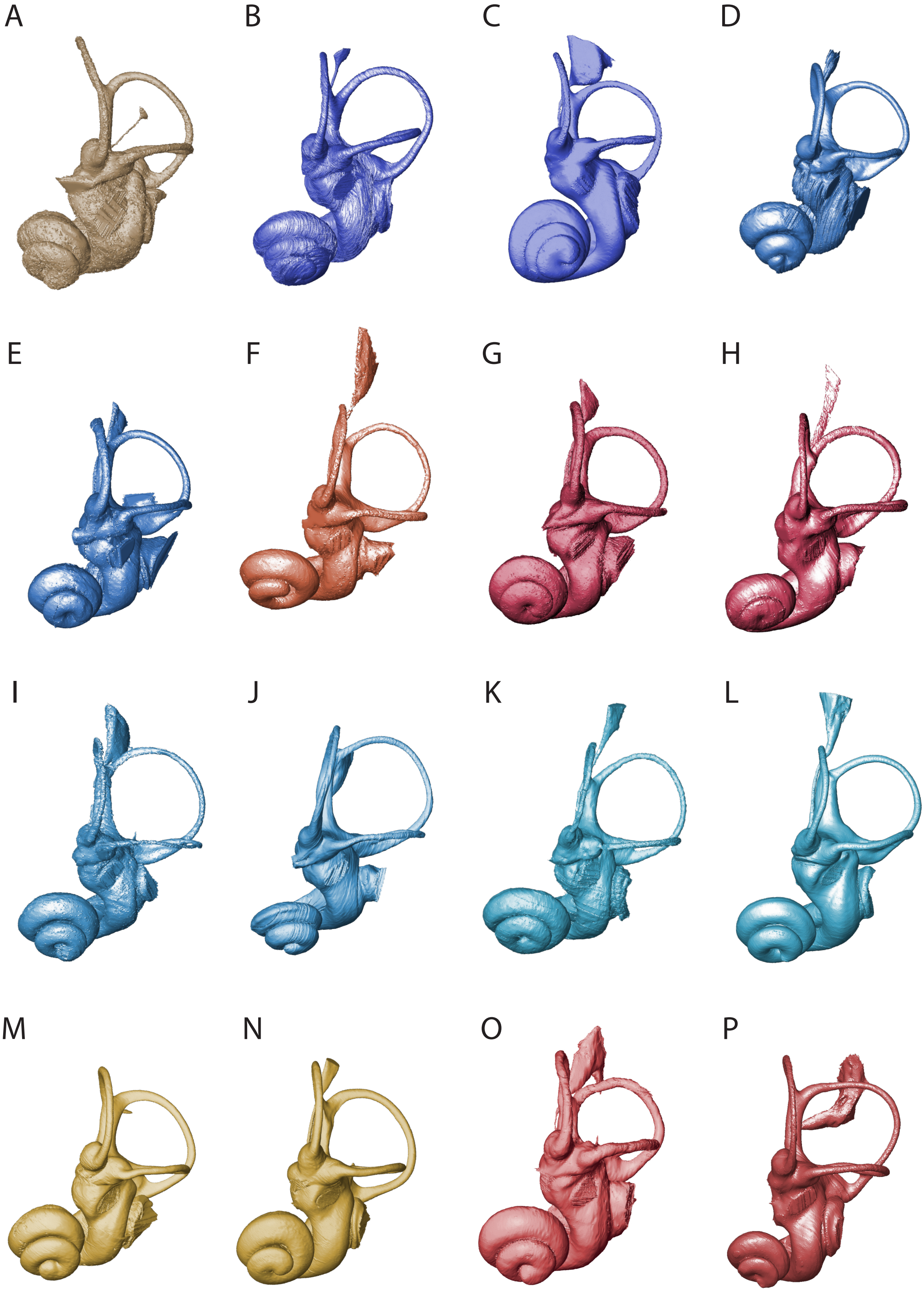
A
B
C
D
E
F
G
H
I
J
K
L
M
N
O
P

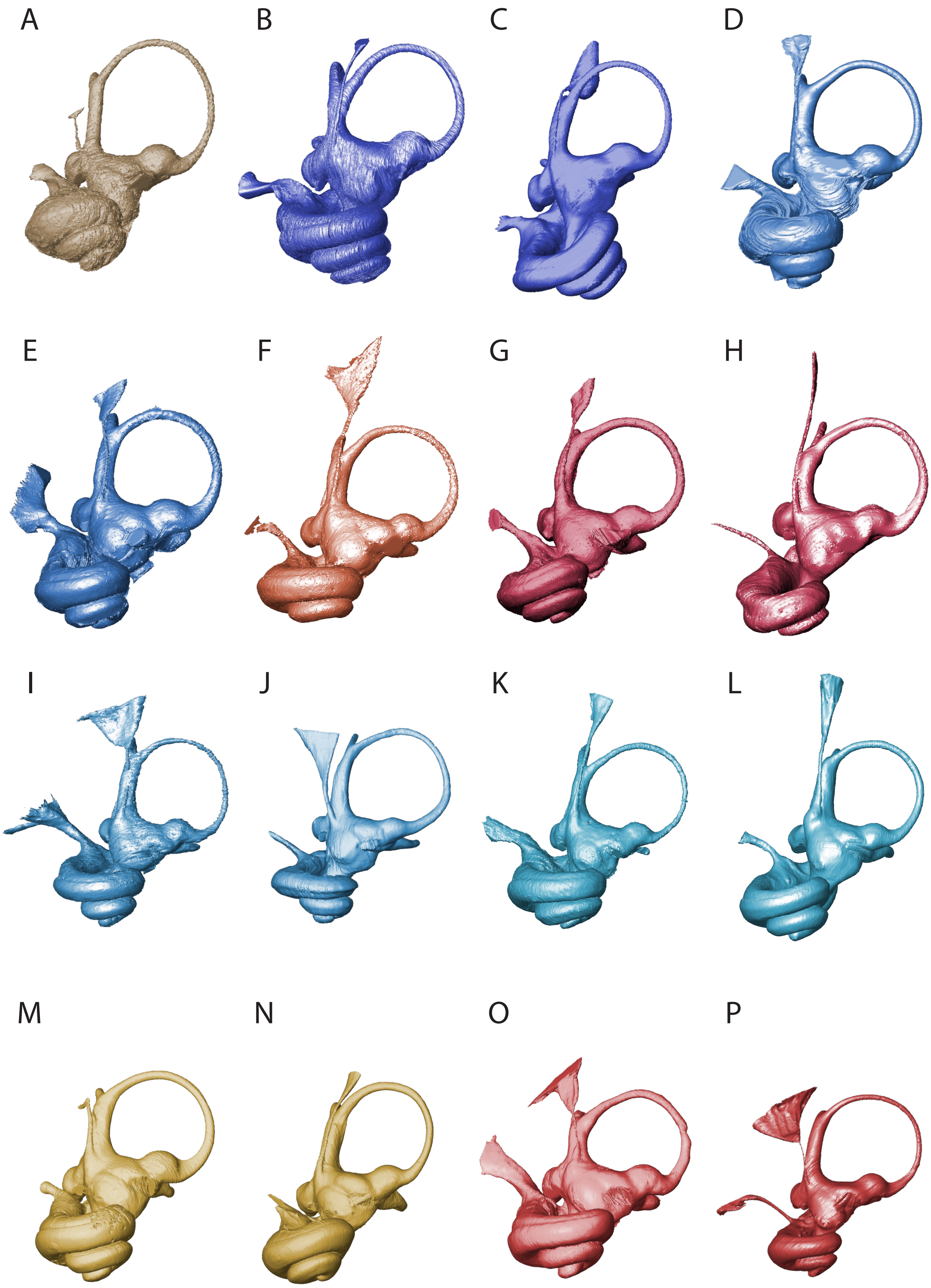
A
B
C
D
E
F
G
H
I
J
K
L
M
N
O
P

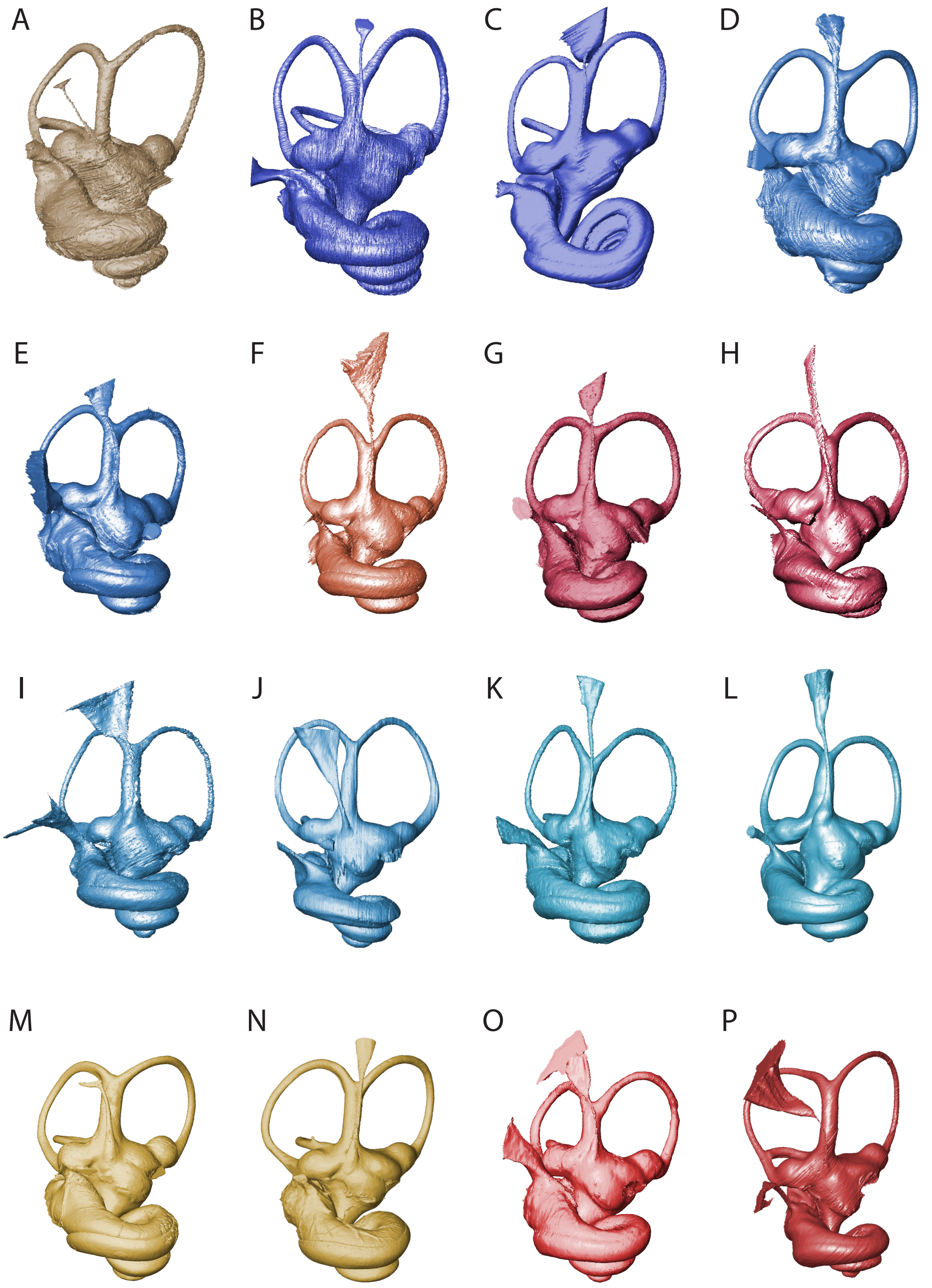
A
B
C
D
E
F
G
H
I
J
K
L
M
N
O
P

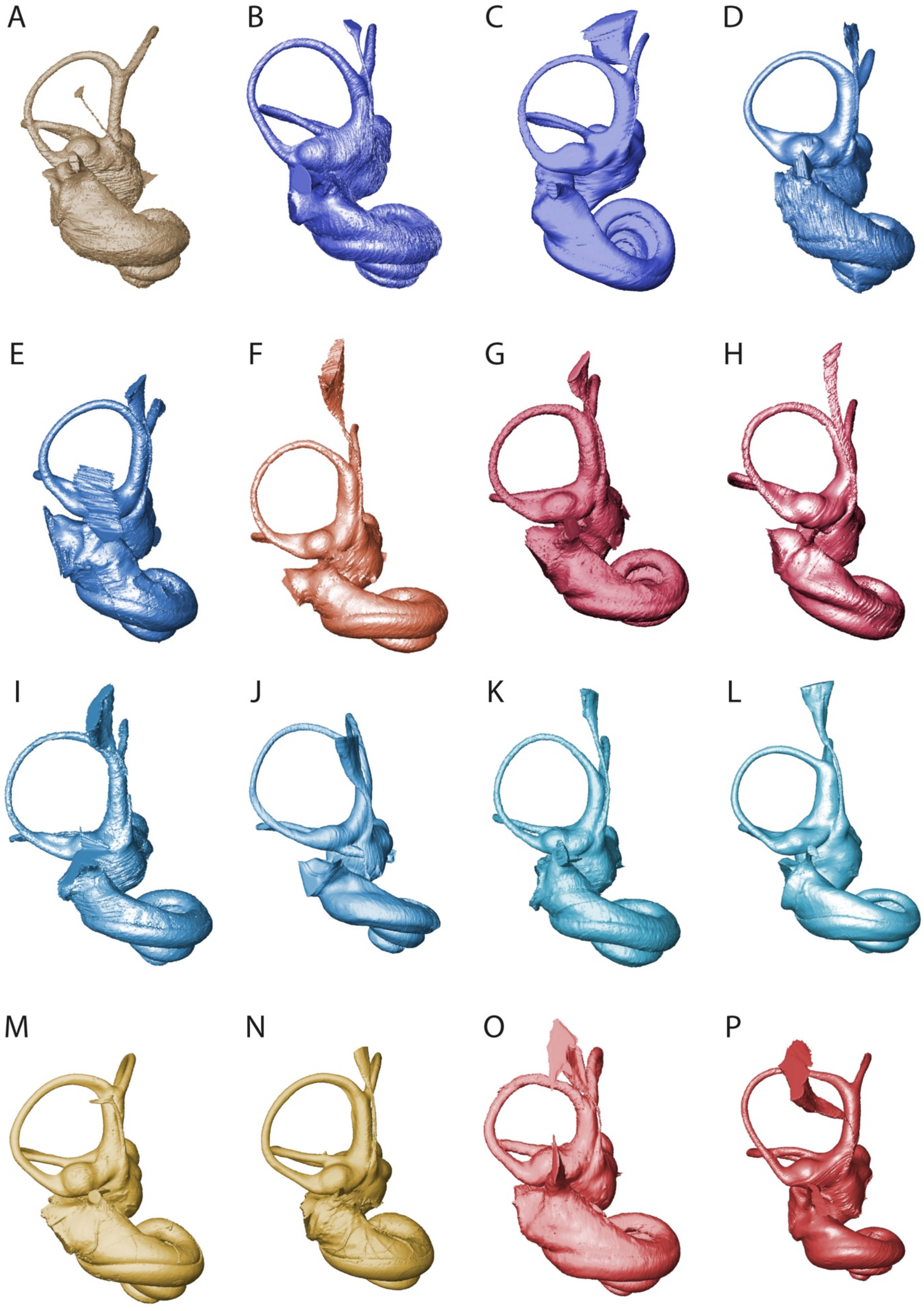
A
B
C
D
E
F
G
H
I
J
K
L
M
N
O
P

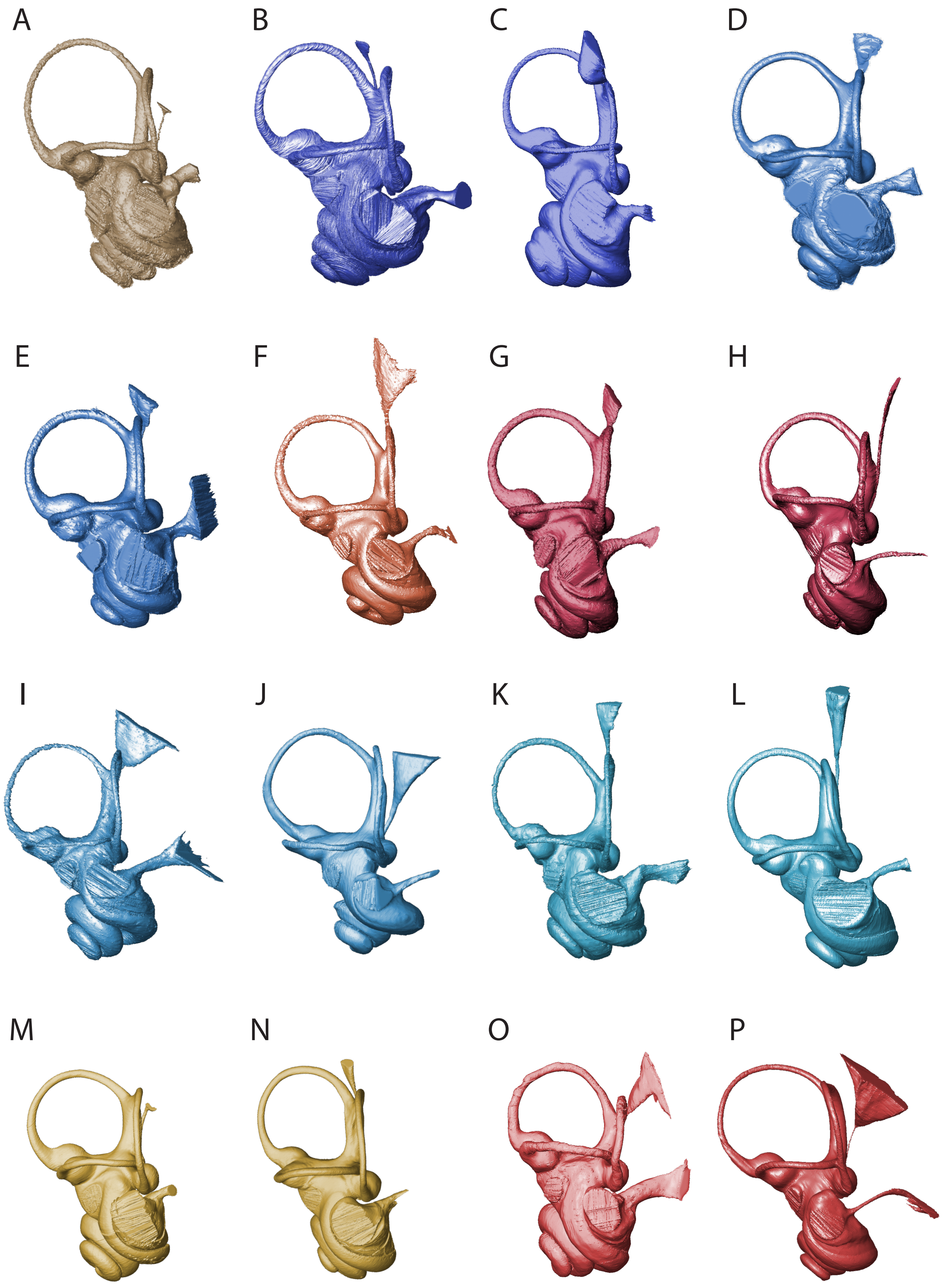
A
B
C
D
E
F
G
H
I
J
K
L
M
N
O
P

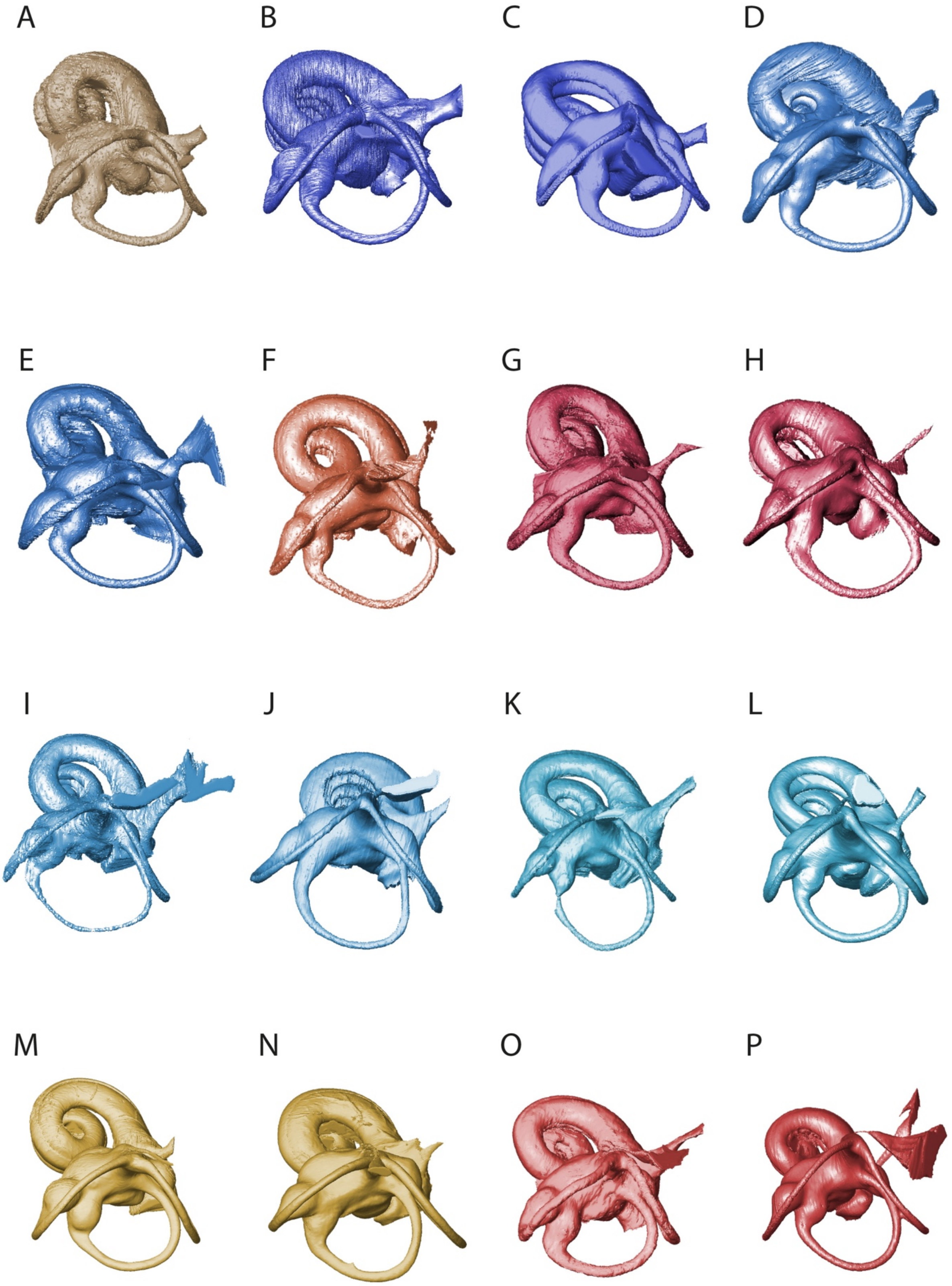
A
B
C
D
E
F
G
H
I
J
K
L
M
N
O
P

Supplementary Figure 3. BL morphology of the different groups of ruminants, including the BL of the oldest currently known genera of all the extant pecoran families: Stem Ruminantia (A *Hypisodus minimus* AMNH9354), Tragulidae (B *Dorcatherium crassum* NMBSan15053 and C *Moschiola meminna* NMB2319), Stem Pecora (D *Prodremotherium elongatum* MNHN.Qu4596 and E *Parablastomeryx primus* AMNH13822), Dromomerycidae (F *Dromomeryx scotti* AMNH FAM33800), Antilocapridae (G *Cosoryx furcatus* AMNH FAM32426 and H *Antilocapra americana* NMB.C.1618), Giraffomorpha (I *Ampelomeryx ginsburgi* Beon91G4 261 and J *Okapia johnstoni* NMB10811), Cervidae (K *Procervulus dichotomus* SNSB-BSPG1979XV555 and L *Cervus elaphus* NMB11147), Moschidae (M *Micromeryx flourensianus* NMBSth825 and N *Moschus moschiferus* NMB4201), and Bovidae (O *Eotragus artenensis* SMNS50Mu and P *Capra ibex* NMB5837). The BL are in rostral view (page 1), medial view (page 2), dorsolateral view (page 3), occipital view (page 4), lateral view (page 5), and dorsal view (page 6).

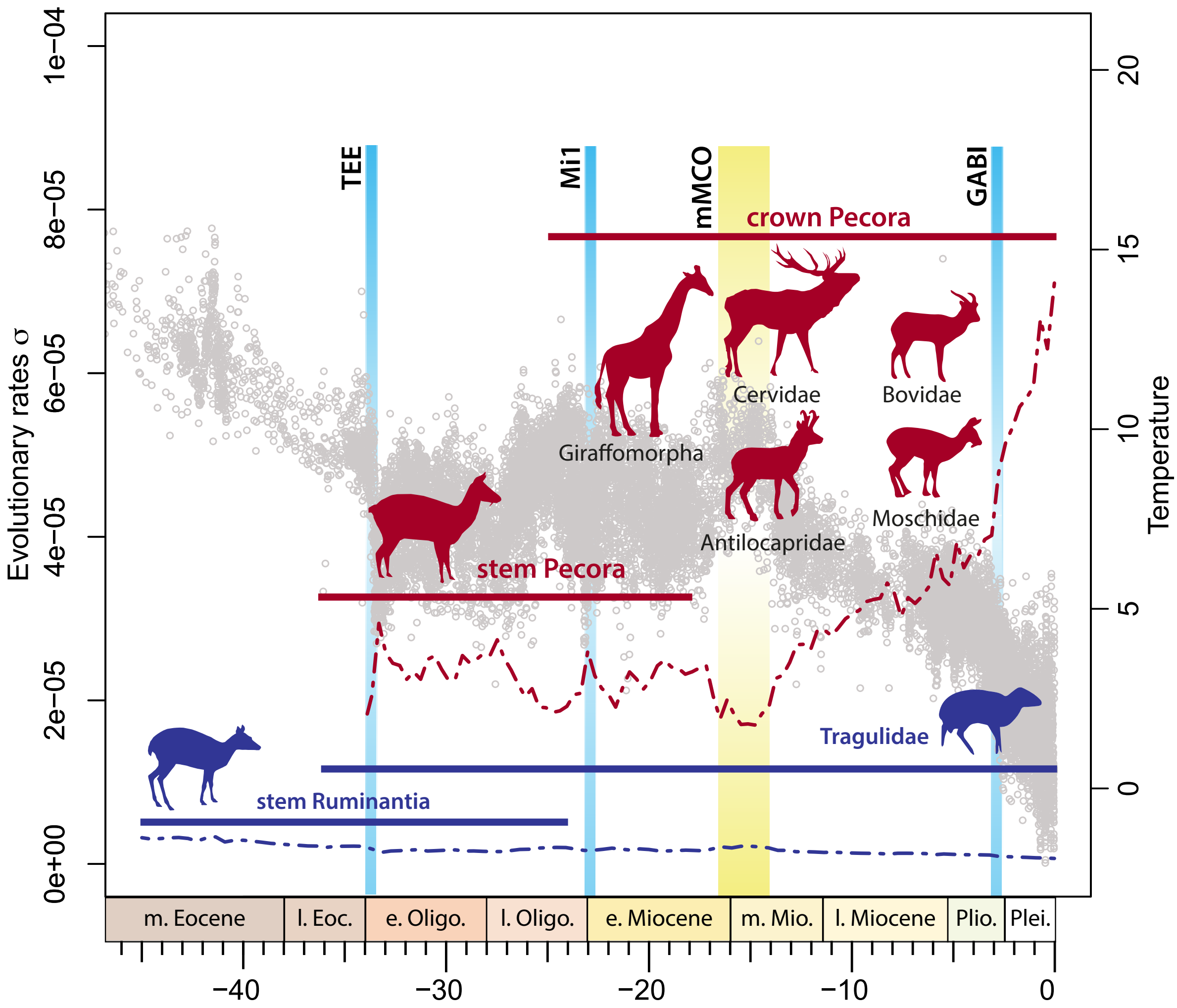

Supplementary Figure 4. Evolutionary rates of all the ruminant BL morphology based on PC scores compared with the global temperature curve[1] (grey dots). The red dashed line represents the evolutionary rates of Stem and Crown Pecora, the dark blue dashed line represents the evolutionary rates of Stem Ruminantia and Tragulidae. Red solid line with associated silhouettes indicate clades included in Pecora with the biostratigraphic range of the Stem and Crown Pecora and dark blue solid line with associated silhouettes indicate clades included in Stem Ruminantia and Tragulidae with their biostratigraphic range. Important biotic (GABI = Great American Biotic Interchange) and abiotic (TEE = Terminal Eocene Event, Mi1 = first Miocene glaciation, mMCO = Middle Miocene Climatic Optimum). Events in blue are related to global cooling, while the event in yellow is linked to global warming. Methodology and statistical results produced by the *R* package *RPanda*[75] are provided in "Material and Methods" and Supplementary Material 1-3. Silhouettes of the families modified from[22].

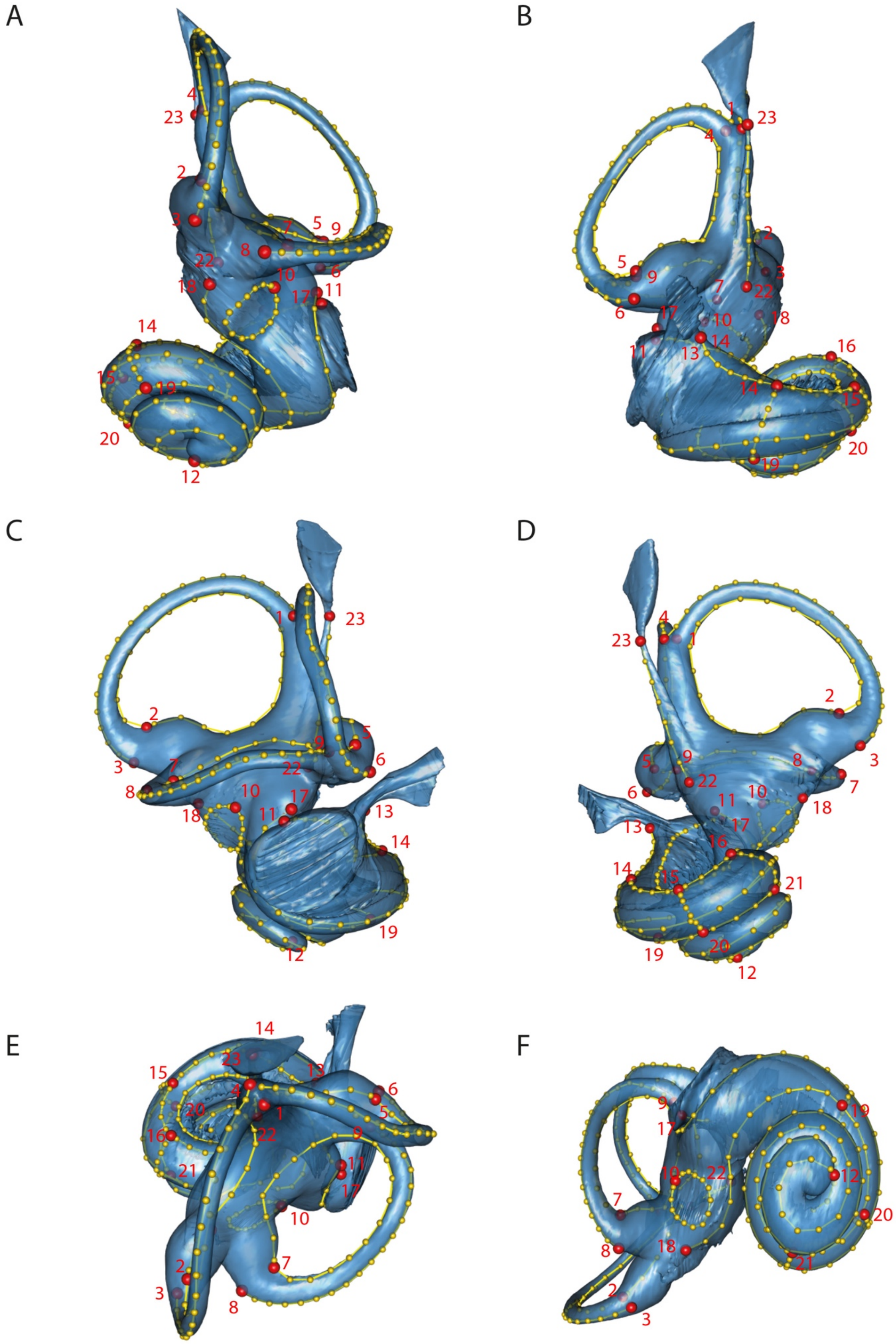
A
4
23
2
3
7
5
9
8
22
6
10
18
17
11
14
15
19
20
12
B
1
23
4
2
5
9
3
7
22
6
17
10
18
11
13
14
16
14
15
20
19
C
1
23
2
5
3
7
9
6
22
8
10
17
18
11
13
14
19
12
D
4
23
1
2
5
9
3
8
22
7
6
11
10
18
13
17
16
14
15
21
20
19
12
E
14
23
15
13
4
6
20
1
5
22
16
9
21
11
17
10
2
7
3
8
F
9
19
17
10
22
12
7
20
8
18
21
2
3

Supplementary Figure 5. Landmarking procedure based on the bony labyrinth of *Hydropotes inermis* NMB9892. Fixed landmarks in red are placed on the following structures: 1 apex of the common crus; 2 anterior portion of the posterior ampula; 3 projection of 2 on the ventral side of the posterior semicircular canal; 4 point of fusion of the posterior and anterior semicircular canals; 5 anterior portion of the anterior ampula; 6 projection of 5 on the ventral side of the anterior semicircular canal; 7 anterior portion of the lateral ampula; 8 projection of 7 on the rostral side of the lateral semicircular canal; 9 insertion of the lateral semicircular canal in the posterior ampula; 10 dorsalmost extension of the stapedial fenestra; 11 beginning of the cochlea inner curve; 12 apex of the cochlea; 13 beginning of the cochlea dorsal curve; 14 first quarter of the first turn of the cochlea dorsal curve; 15 second quarter of the first turn of the cochlea dorsal curve; 16 third quarter of the first turn of the cochlea dorsal curve; 17 beginning of the cochlea lateral curve; 18 beginning of the cochlea lower curve; 19 first quarter of the first turn of the cochlea lower curve; 20 second quarter of the first turn of the cochlea lower curve; 21 third quarter of the first turn of the cochlea lower curve; 22 base of the vestibular aqueduct; 23 apex of the vestibular aqueduct at the base of the endolymphatic sac. 15 curves containing semi-landmarks (SM) in yellow have been placed between the landmarks. 1 thickness of the first quarter of the first turn of the cochlea (SM: 8); 2 thickness of the second quarter of the first turn of the cochlea (SM: 8); 3 thickness of the third quarter of the first turn of the cochlea (SM: 8); 4 inner section of the anterior semicircular canal (SM: 20); 5 outer section of the anterior semicircular canal (SM: 20); 6 cochlea inner curve (SM: 30); 7 cochlea dorsal curve (SM: 30); 8 cochlea lateral curve (SM: 40); 9 cochlea lower curve (SM: 40); 10 stapedial fenestra (SM: 15); 11 inner section of the lateral semicircular canal (SM: 20); 12 outer section of the lateral semicircular canal (SM: 20); 13 inner section of the posterior semicircular canal (SM: 20); 14 outer section of the posterior semicircular canal (SM: 20); 15 vestibular aqueduct (SM: 8). The BL are in rostral rostral (A), occipital (B), lateral (C), medial (D), dorsal (E), and ventral (F) views.

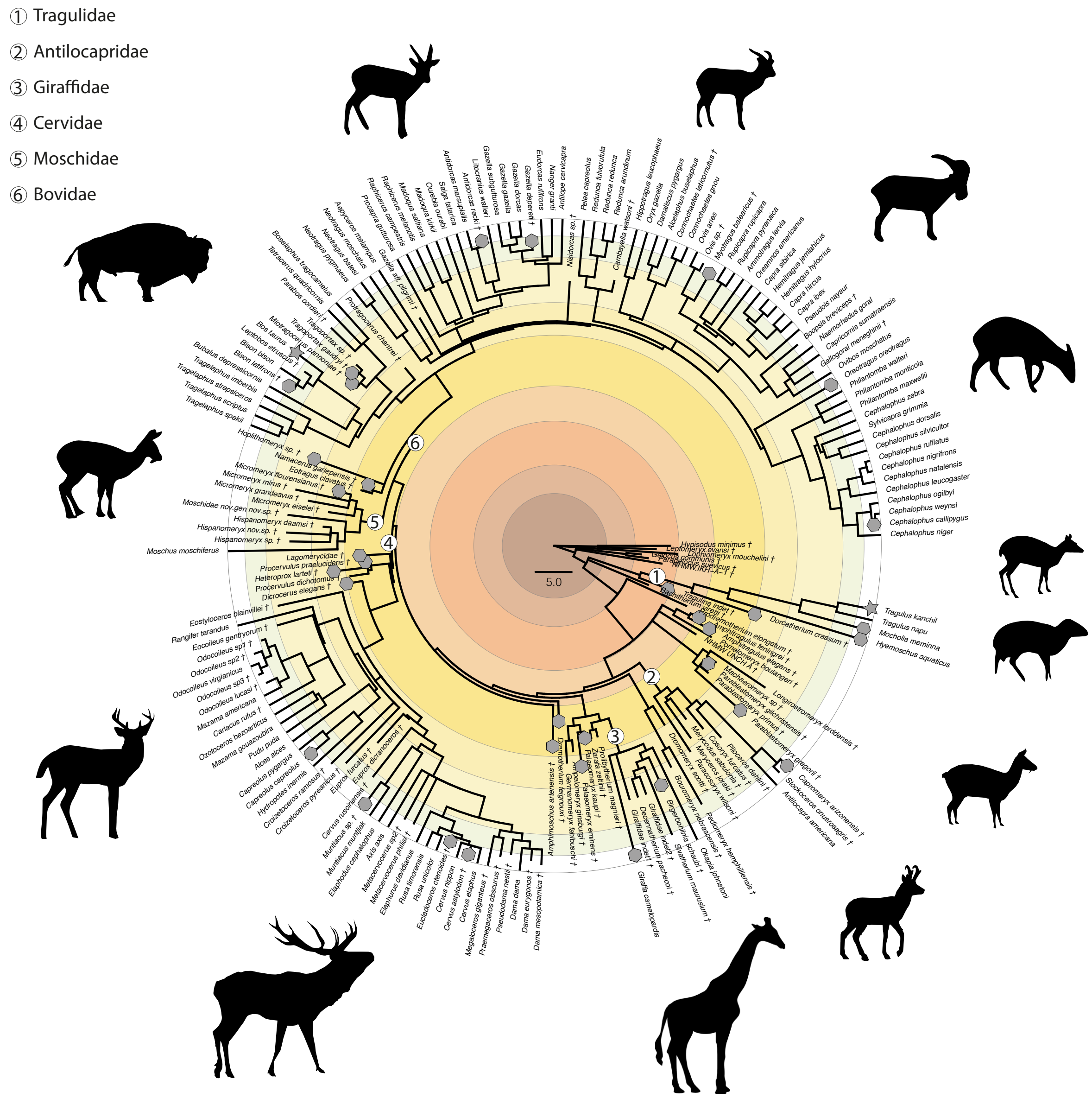

Supplementary Figure 6. Calibrated phylogenetic hypothesis of the studied ruminants. Topology and calibration are explained in the Material and Methods section. Hexagons indicate species where several specimens were sampled and stars indicate species where several specimens were sampled including fetuses. Silhouettes of the families modified from[22].

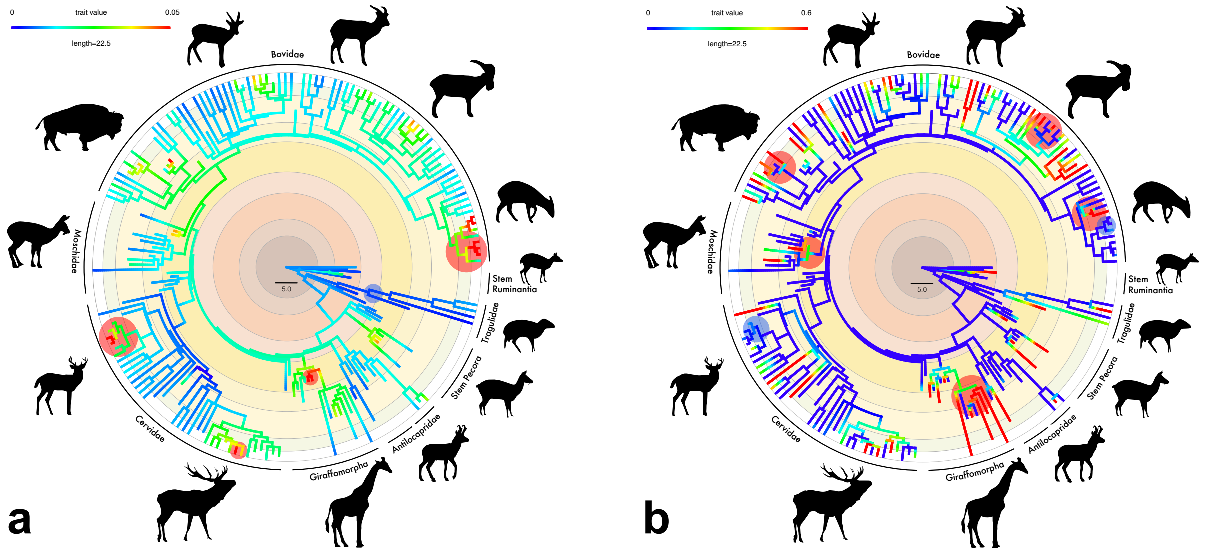

Supplementary Figure 7. Comparative evolutionary rates of the ruminant BL morphology based on PC scores (**a**) and of the size based on the centroid size (**b**) through the phylogenetic tree. Significant decrease in the evolutionary rates are marked by a blue circle, while a significant increase of the evolutionary rates is marked by a red circle. We can observe that number of significant shifts, their location in the tree, and the nature of the shift (significant decrease or increase of the evolutionary rate) differ when considering the evolutionary rates of the ruminant BL morphology and of the size based on the centroid size. Methodology and statistical results produced by the R packages RRphylo[93] and Phytools[90] are provided in Material and Methods and Supplementary Material 1-2. Silhouettes of the families modified from[22]. Same color code as in Figure 2 for ages.

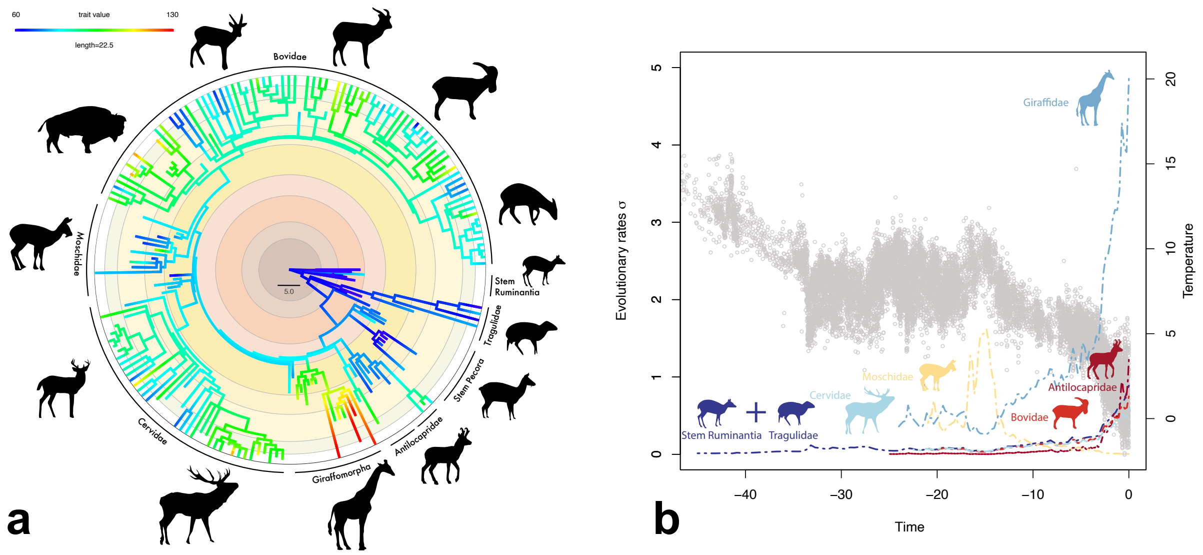

Supplementary Figure 8. Comparative evolutionary rates of the size based on the centroid size (**a**) through the phylogenetic tree and evolutionary rates of all the ruminant BL centroid compared with the global temperature curve[12] (grey dots) (**b**). We can observe a gradual evolution toward larger forms (**a**), dark blue species (the smallest ones) being located close to the root of the tree, while green and red ones are observed in younger forms. Except for the Moschidae, all the ruminants present an increase in the evolutionary rates of their centroid size (**b**). Methodology and statistical results produced by RRphylo[93] and Phytools[90] are provided in Material and Methods and Supplementary Material 1-2 and by the *R* package *RPanda*[75] are provided in "Material and Methods" and Supplementary Material 1-3. Silhouettes of the families modified from[22]. Same color code as in Figure 2 for ages.
