# Supplementary material for: Ruminant inner ear shape records 35 million years of neutral evolution
Source: Nat Commun. 2022 Dec 6;13:7222. doi: 10.1038/s41467-022-34656-0 (PMC9726890; doi:10.1038/s41467-022-34656-0)
Supplement: Supplementary file 2 — Description of Additional Supplementary Files [file 41467_2022_34656_MOESM2_ESM.pdf]

Description of Additional Supplementary Information

Supplementary Data 1. R code, statistical results, and supplementary figures of the PCA (306 specimens), bg-PCA (306 specimens), CVA (306 specimens) using the R packages geomorph v.4.0.3 and Morpho v.2.9, phylogenetic signal (191 species) based on the PC scores using the permutation test implemented in MorphoJ and the fonction "*phylosig*" of the package *Phytools* v.1.0.39[90], evolutionary rates (191 species) using the function "*RRphylo*" in the *R* package *RRphylo* v2.6.0[93], significance of the evolutionary rate shifts using the function "*search.shift*" in the *R* package *RRphylo* v2.6.0[93], correlation, anticorrelation, or no correlation between the Bony labyrinth morphology evolutionary rate and an environmental function through time using the function "*fit_t_env*" in the *R* package *RPANDA* v2.0[95], and R Script.

Supplementary Data 1-1: geometric morphometrics**.** Results of the geometric morphometrics study (PCA, between-group PCA (bg-PCA) and CVA) for the dataset 306 specimens. Contains also a legend that can be used for all graphs of this file, and a file with an animated 3D representation of the results through time in two different views (3Dgraphs_gif). **Supplementary Data 1-2: RRphylo.** This file contains datasets necessary for calculation of evolutionary rates and shifts (PCScores, CV-Scores bg-PC-Scores), the phylogenetic tree and the results for each analysis. Each sub-folder contains (XXX for each dataset: PCA and centroid sizes): Evolutionary rates plotted on phylogenetic tree (XXX_evorates_no_ancestral_states.pdf). Evolutionary rates plotted on phylogenetic tree including number of nodes (XXX_evorates_nodes.pdf). Significant shifts plotted on phylogenetic tree (XXX_AR results for rate differences.pdf). Table with significant shifts (XXX_SC_shift_191.csv). Evolutionary rates per species (evorates_XXX.csv). **Supplementary Data 1-3: RPANDA.** Folder contains datasets necessary for correlation with environmental function (F-evorates_XXX.csv) and phylogenetic tree. Each subfolder contains (XXX for each dataset: PCA and centroid size): Correlation with environmental function for Tragulina (Stem Ruminantia and Tragulidae) and Pecora (Antilocapridae, Bovidae, Cervidae, Giraffidae, Moschidae) (RPANDA_XXX_VS_temperature_Pecora-Tragulina.pdf). Correlation with environmental function for Tragulina (Stem Ruminantia and Tragulidae), Antilocapridae, Bovidae, Cervidae, Giraffidae, Moschidae (RPANDA_XXX_VS_temperature_Tragulina_ Pecoran_families.pdf). AIC/AICC/likelihood results for Tragulina (Stem Ruminantia and Tragulidae) and Pecora (Antilocapridae, Bovidae, Cervidae, Giraffidae, Moschidae) (AIC_results_XXX_Pecora_Tragulina.csv). AIC/AICC/likelihood results for Tragulina (Stem Ruminantia and Tragulidae), Antilocapridae, Bovidae, Cervidae, Giraffidae, Moschidae (AIC_results _XXX_evorates_Tragulina_ Pecoran_families.csv). AIC/AICC/likelihood scores for different degrees of smoothing (AIC_significance_XXX_per-family_per-df.csv). Table with sigma and beta parameters for different degrees of smoothing (parameters_sigma_beta_XXX_per-family_per-df.csv). **Supplementary Data 1-4: raw dataset and R code.** Contains raw dataset for all 306 specimens, all 191 species, and R code. Data_306 contains: Species list (speclist_0322.csv). landmarks data (ruminants_resampled_2d_array_0322.csv). classifiers (classifier_family_species_0322.csv, family_0322.csv). curve matrix for geometric morphometric analyses (curves_0322.csv). centroid size per specimen and age of the specimen (centroid_306_rum.csv). Data_191 contains: Classifiers (family_191_0322.csv). curve matrix for geometric morphometric analyses (curves_0322.csv). centroid size per species and age of the specimen (centroid_191_rum.csv). Phylogenetic signal (physignal.csv). Phylogenetic tree (tree_191.tre). Code contains (run in RStudio): Resampling procedure for semilandmarks (resampling_semilandmarks.Rmd). Geometric morphometric analyses on 306 specimens (PCA_ 306sp.Rmd). Calculation of evolutionary rates and shifts (RRPhylo_191.Rmd). Correlation with environmental function for centroid results (Rpanda_centroid.Rmd). Correlation with environmental function for PCA results (Rpanda_pca.Rmd). Parameters (AiC, AiCC, likelihood, beta, sigma) (params_rpanda.Rmd).

Supplementary Data 2. List of specimens and related information (taxonomic group, inventory number, host institution, age, and locality).
