# Supplementary material for: Ruminant inner ear shape records 35 million years of neutral evolution
Source: Nat Commun. 2022 Dec 6;13:7222. doi: 10.1038/s41467-022-34656-0 (PMC9726890; doi:10.1038/s41467-022-34656-0)
Supplement: Supplementary file 3 — Supplementary Data 1 [file 41467_2022_34656_MOESM3_ESM.zip › Supplementary data_1/Content of Supplementary Data 1.docx]

**- Supplementary material 1-1: geometric morphometrics**

Results of the geometric morphometrics study (PCA, between-group PCA (bg-PCA) and CVA) for the dataset 306 specimens

Contains also a legend that can be used for all graphs of this file, and a file with an animated 3D representation of the results through time in two different views (3Dgraphs_gif)

**- Supplementary material 1-2: RRphylo**

This file contains datasets necessary for calculation of evolutionary rates and shifts (PC- Scores, CV-Scores bg-PC-Scores), the phylogenetic tree and the results for each analysis.

Each sub-folder contains (XXX for each dataset: PCA and centroid sizes):

Evolutionary rates plotted on phylogenetic tree (XXX_evorates_no_ancestral_states.pdf)

Evolutionary rates plotted on phylogenetic tree including number of nodes (XXX_evorates_nodes.pdf)

Significant shifts plotted on phylogenetic tree (XXX_AR results for rate differences.pdf)

Table with significant shifts (XXX_SC_shift_191.csv)

Evolutionary rates per species (evorates_XXX.csv)

**- Supplementary material 1-3: RPANDA**

Folder contains datasets necessary for correlation with environmental function (F-evorates_XXX.csv) and phylogenetic tree.

Each subfolder contains (XXX for each dataset : PCA and centroid size):

Correlation with environmental function for Tragulina (Stem Ruminantia and Tragulidae) and Pecora (Antilocapridae, Bovidae, Cervidae, Giraffidae, Moschidae) (RPANDA_XXX_VS_temperature_Pecora-Tragulina.pdf)

Correlation with environmental function for Tragulina (Stem Ruminantia and Tragulidae), Antilocapridae, Bovidae, Cervidae, Giraffidae, Moschidae (RPANDA_XXX_VS_temperature_Tragulina_ Pecoran_families.pdf)

AIC/AICC/likelihood results for Tragulina (Stem Ruminantia and Tragulidae) and Pecora (Antilocapridae, Bovidae, Cervidae, Giraffidae, Moschidae) (AIC_results_XXX_Pecora_Tragulina.csv)

AIC/AICC/likelihood results for Tragulina (Stem Ruminantia and Tragulidae), Antilocapridae, Bovidae, Cervidae, Giraffidae, Moschidae (AIC_results _XXX_evorates_Tragulina_ Pecoran_families.csv)

AIC/AICC/likelihood scores for different degrees of smoothing (AIC_significance_XXX_per-family_per-df.csv)

Table with sigma and beta parameters for different degrees of smoothing (parameters_sigma_beta_XXX_per-family_per-df.csv)

**- Supplementary material 1-4: raw dataset and R code**

Contains raw dataset for all 306 specimens, all 191 species, and R code

Data_306 contains:

Species list (speclist_0322.csv)

landmarks data (ruminants_resampled_2d_array_0322.csv)

classifiers (classifier_family_species_0322.csv, family_0322.csv)

curve matrix for geometric morphometric analyses (curves_0322.csv)

centroid size per specimen and age of the specimen (centroid_306_rum.csv)

Data_191 contains:

Classifiers (family_191_0322.csv)

curve matrix for geometric morphometric analyses (curves_0322.csv)

centroid size per species and age of the specimen (centroid_191_rum.csv)

Phylogenetic signal (physignal.csv)

Phylogenetic tree (tree_191.tre)

Code contains (run in RStudio):

Resampling procedure for semilandmarks (resampling_semilandmarks.Rmd)

Geometric morphometric analyses on 306 specimens (PCA_ 306sp.Rmd)

Calculation of evolutionary rates and shifts (RRPhylo_191.Rmd)

Correlation with environmental function for centroid results (Rpanda_centroid.Rmd)

Correlation with environmental function for PCA results (Rpanda_pca.Rmd)

Parameters (AiC, AiCC, likelihood) (params_rpanda.Rmd)
