# Supplementary figures and images for: Ruminant inner ear shape records 35 million years of neutral evolution (part 2 of 2)
Source: Nat Commun. 2022 Dec 6;13:7222. doi: 10.1038/s41467-022-34656-0 (PMC9726890; doi:10.1038/s41467-022-34656-0)

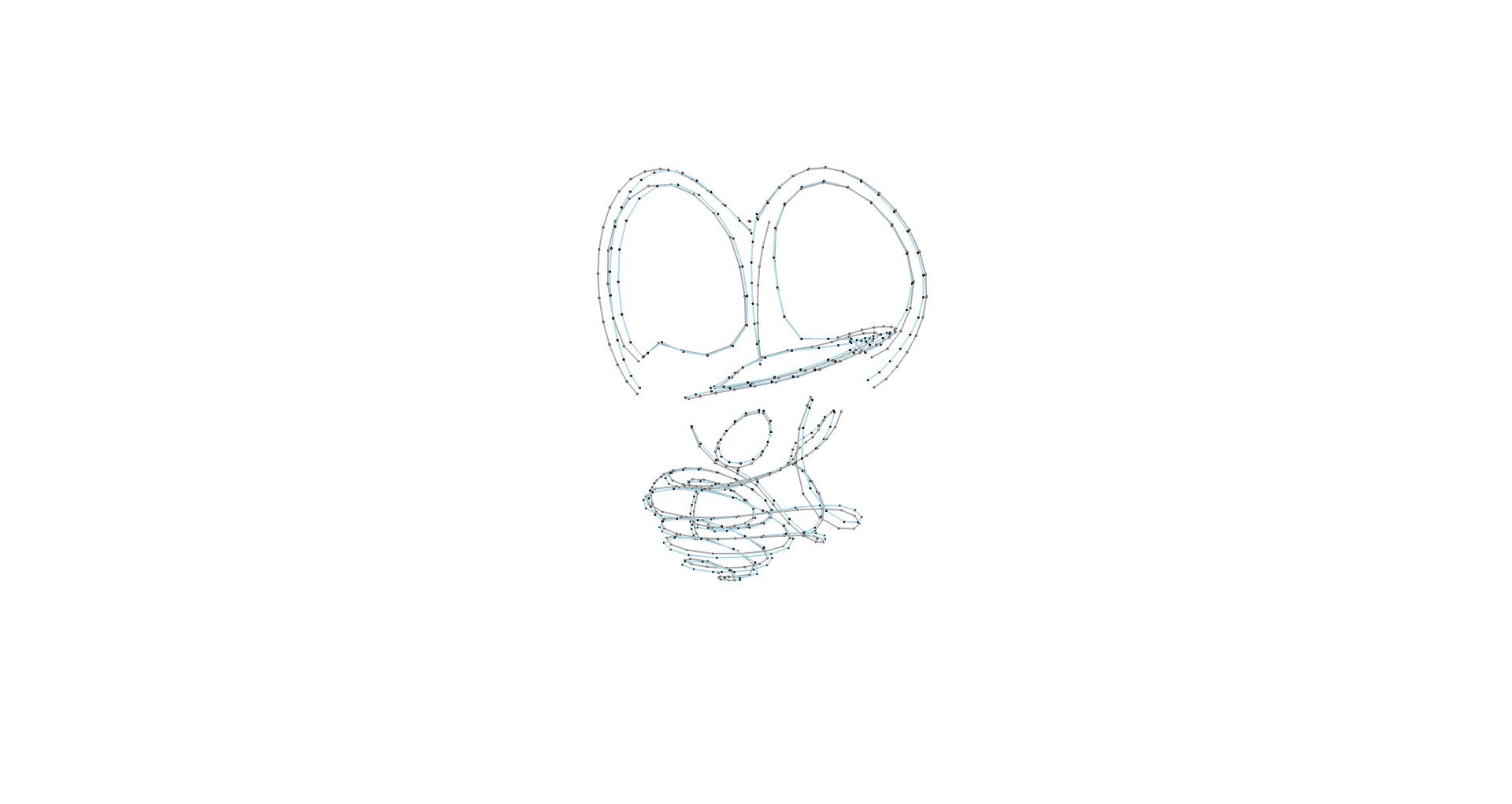

Supplement: Supplementary file 3 — Supplementary Data 1 [file 41467_2022_34656_MOESM3_ESM.zip › Supplementary data_1/Supplementary_material_1-1 Geometric morphometrics/CVA_306/mean_shapes_per_clade_CVA/Cervidae-vl.png]

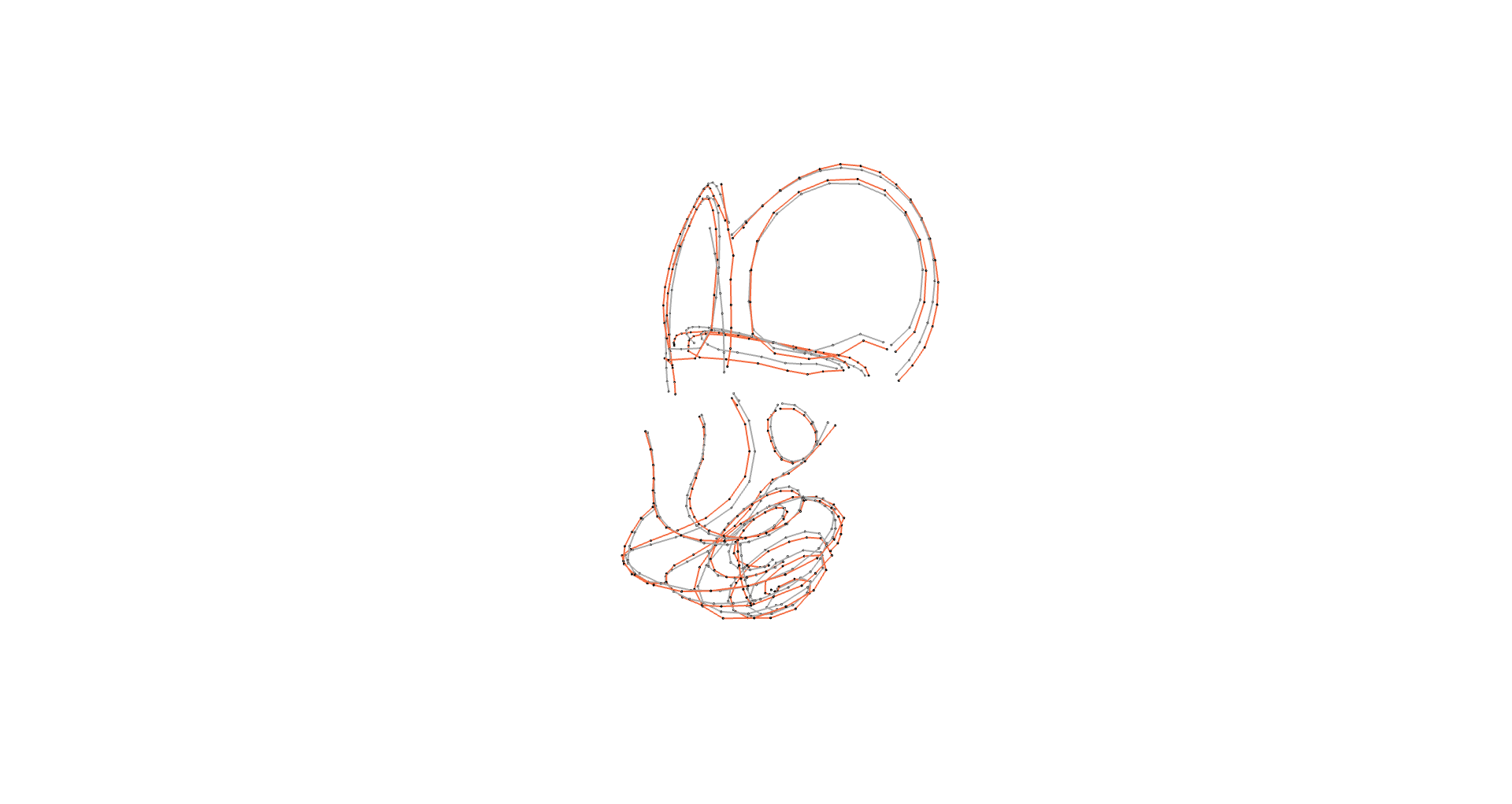

Supplement: Supplementary file 3 — Supplementary Data 1 [file 41467_2022_34656_MOESM3_ESM.zip › Supplementary data_1/Supplementary_material_1-1 Geometric morphometrics/CVA_306/mean_shapes_per_clade_CVA/Dromomerycidae-dl.png]

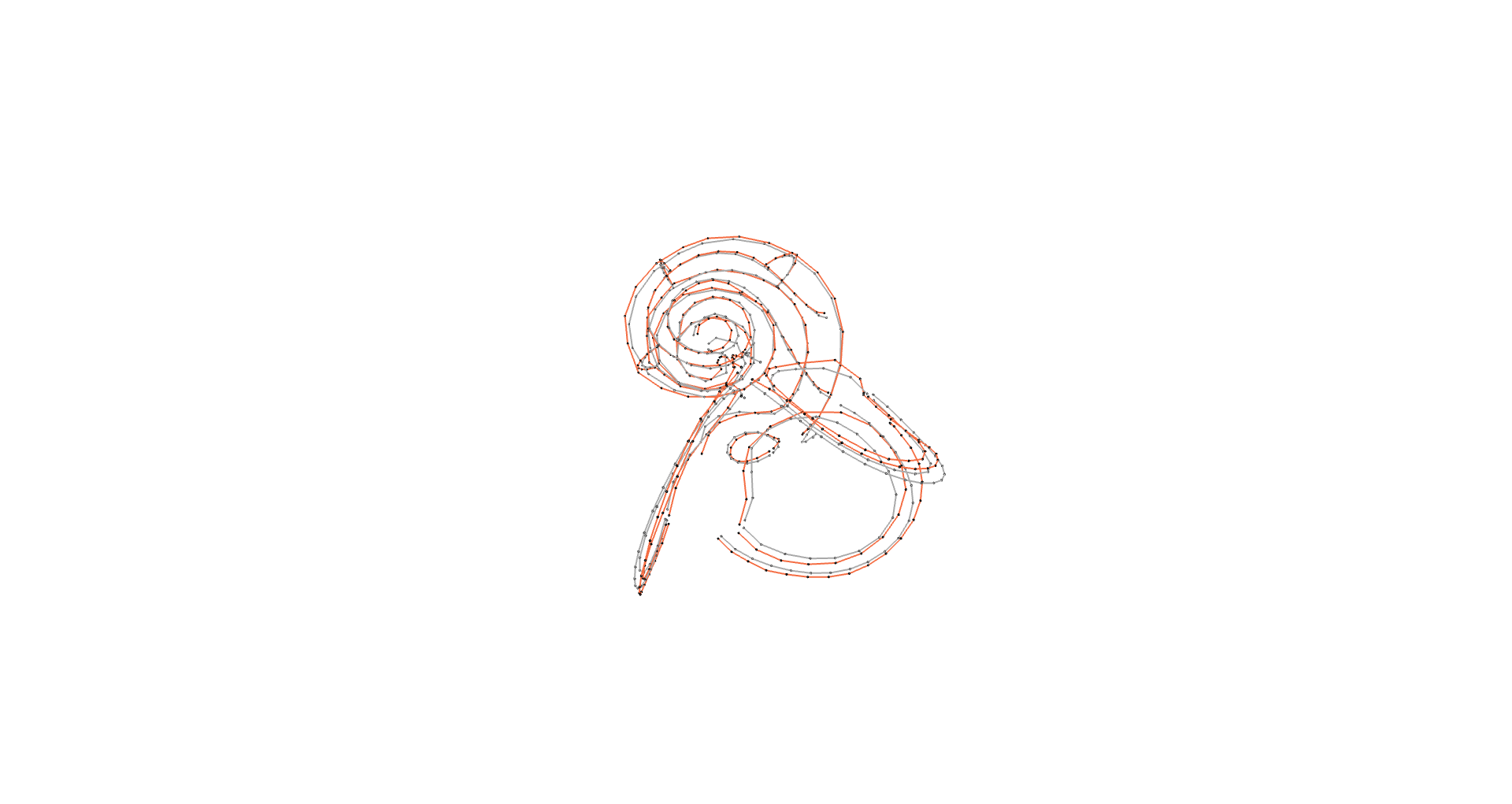

Supplement: Supplementary file 3 — Supplementary Data 1 [file 41467_2022_34656_MOESM3_ESM.zip › Supplementary data_1/Supplementary_material_1-1 Geometric morphometrics/CVA_306/mean_shapes_per_clade_CVA/Dromomerycidae-do.png]

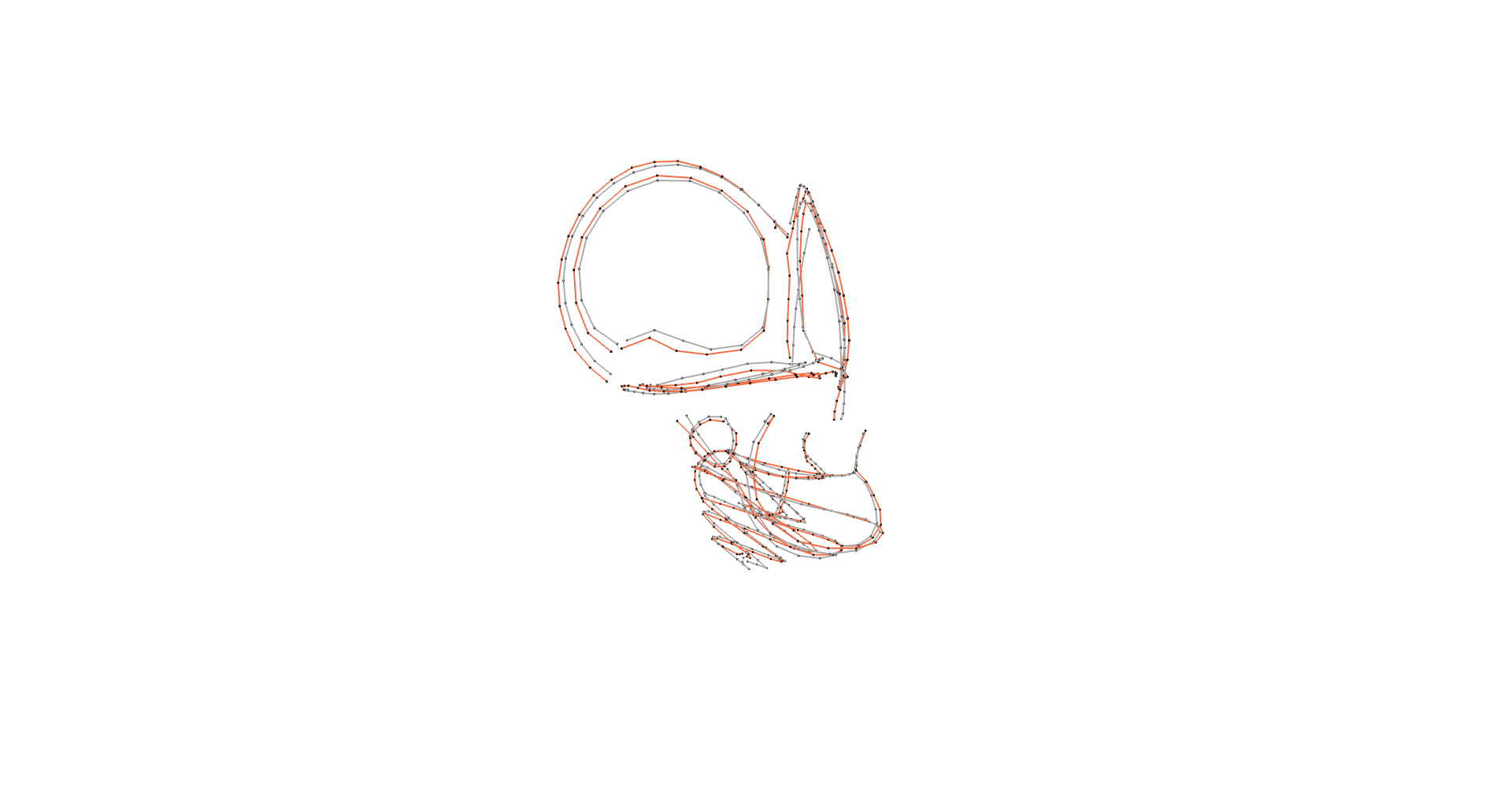

Supplement: Supplementary file 3 — Supplementary Data 1 [file 41467_2022_34656_MOESM3_ESM.zip › Supplementary data_1/Supplementary_material_1-1 Geometric morphometrics/CVA_306/mean_shapes_per_clade_CVA/Dromomerycidae-la.png]

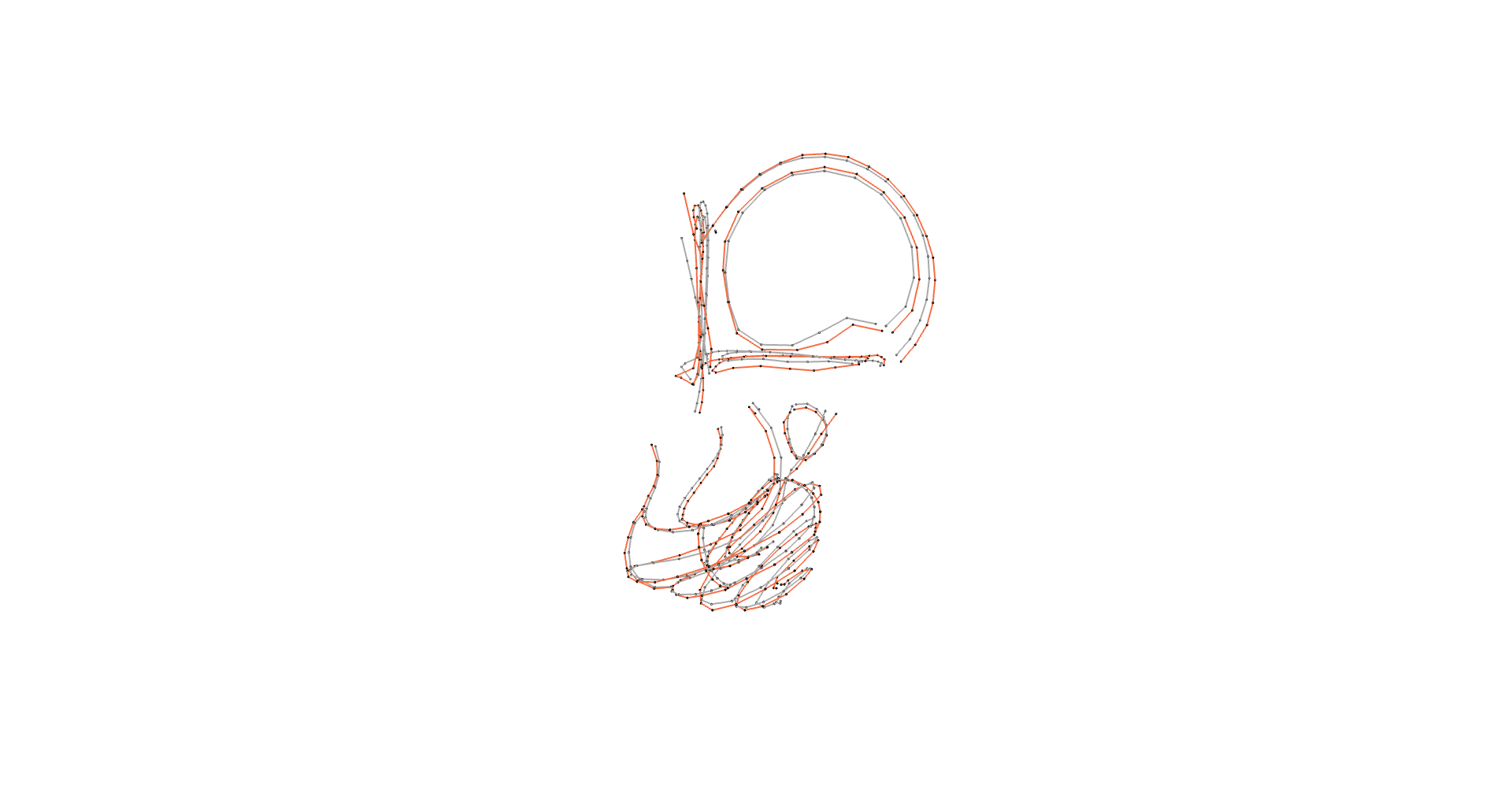

Supplement: Supplementary file 3 — Supplementary Data 1 [file 41467_2022_34656_MOESM3_ESM.zip › Supplementary data_1/Supplementary_material_1-1 Geometric morphometrics/CVA_306/mean_shapes_per_clade_CVA/Dromomerycidae-me.png]

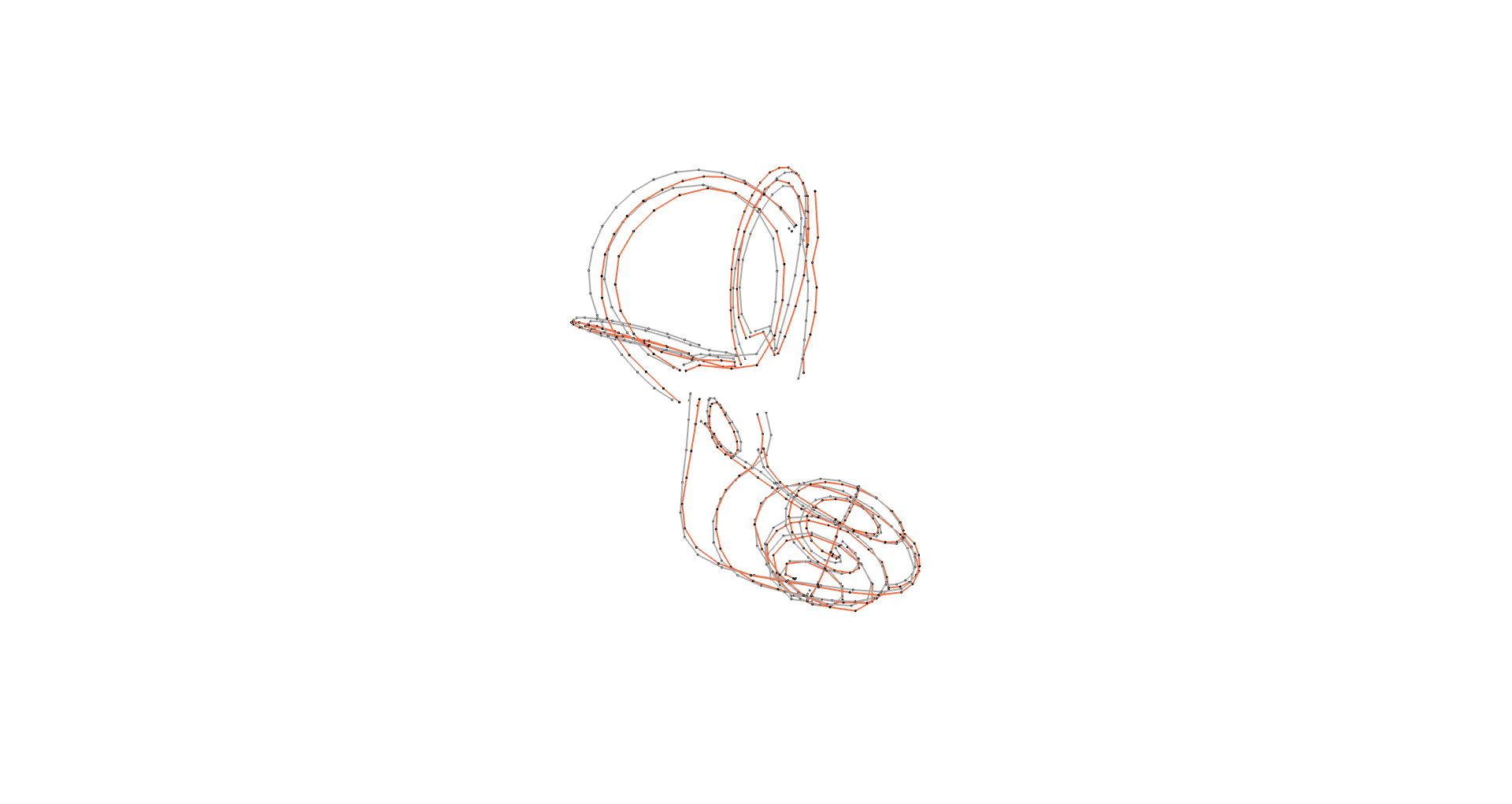

Supplement: Supplementary file 3 — Supplementary Data 1 [file 41467_2022_34656_MOESM3_ESM.zip › Supplementary data_1/Supplementary_material_1-1 Geometric morphometrics/CVA_306/mean_shapes_per_clade_CVA/Dromomerycidae-oc.png]

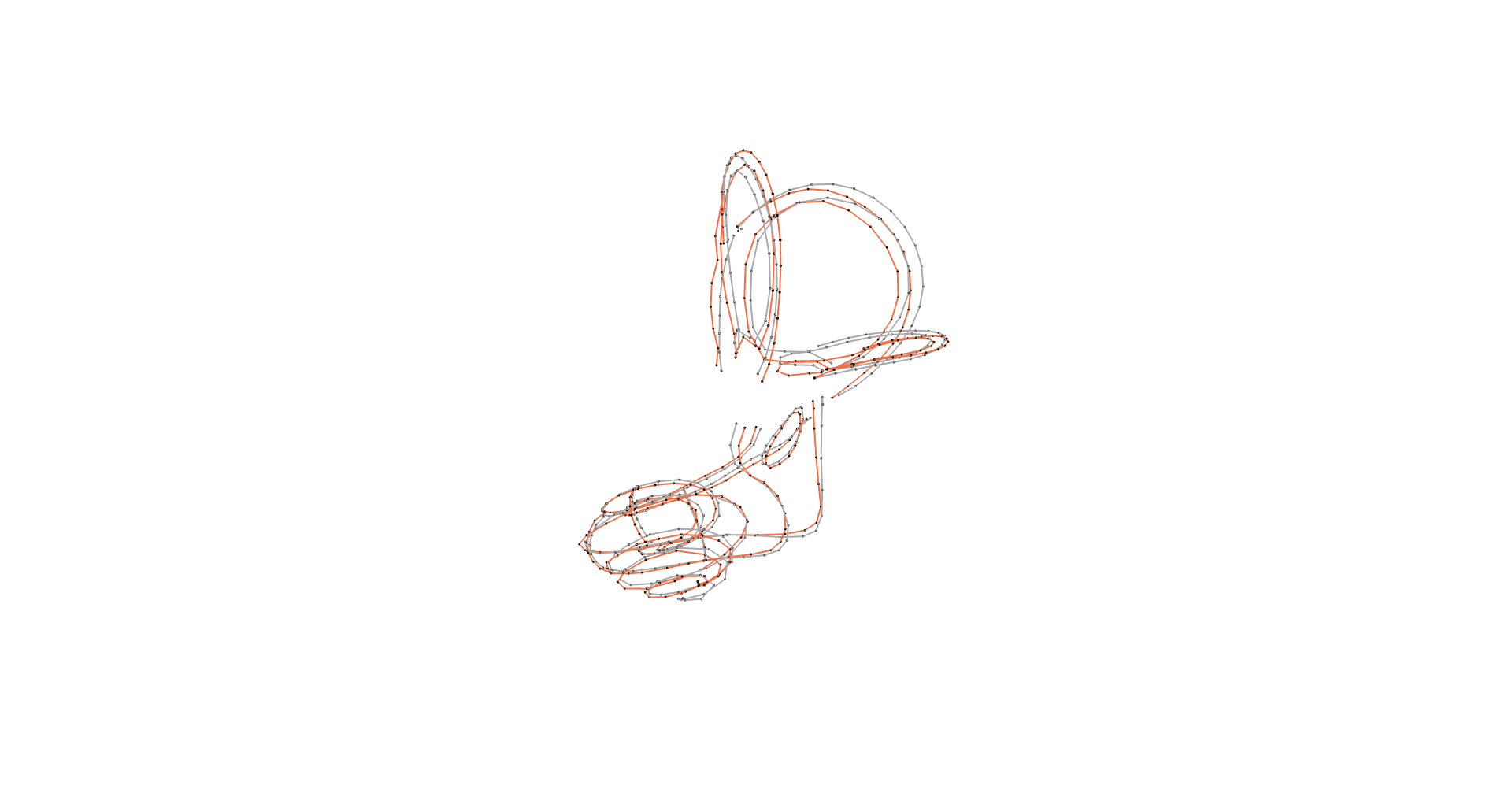

Supplement: Supplementary file 3 — Supplementary Data 1 [file 41467_2022_34656_MOESM3_ESM.zip › Supplementary data_1/Supplementary_material_1-1 Geometric morphometrics/CVA_306/mean_shapes_per_clade_CVA/Dromomerycidae-ro.png]

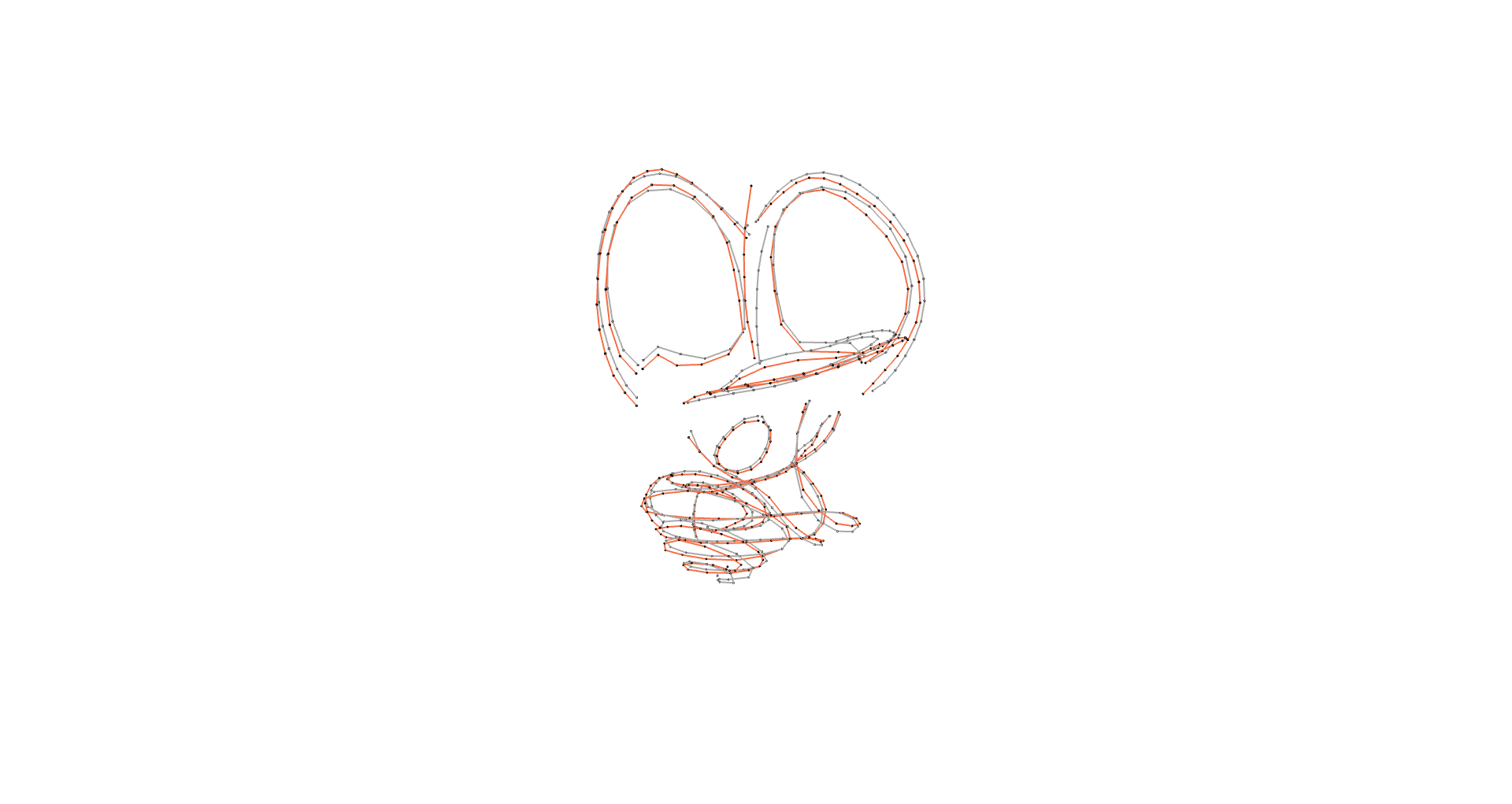

Supplement: Supplementary file 3 — Supplementary Data 1 [file 41467_2022_34656_MOESM3_ESM.zip › Supplementary data_1/Supplementary_material_1-1 Geometric morphometrics/CVA_306/mean_shapes_per_clade_CVA/Dromomerycidae-vl.png]

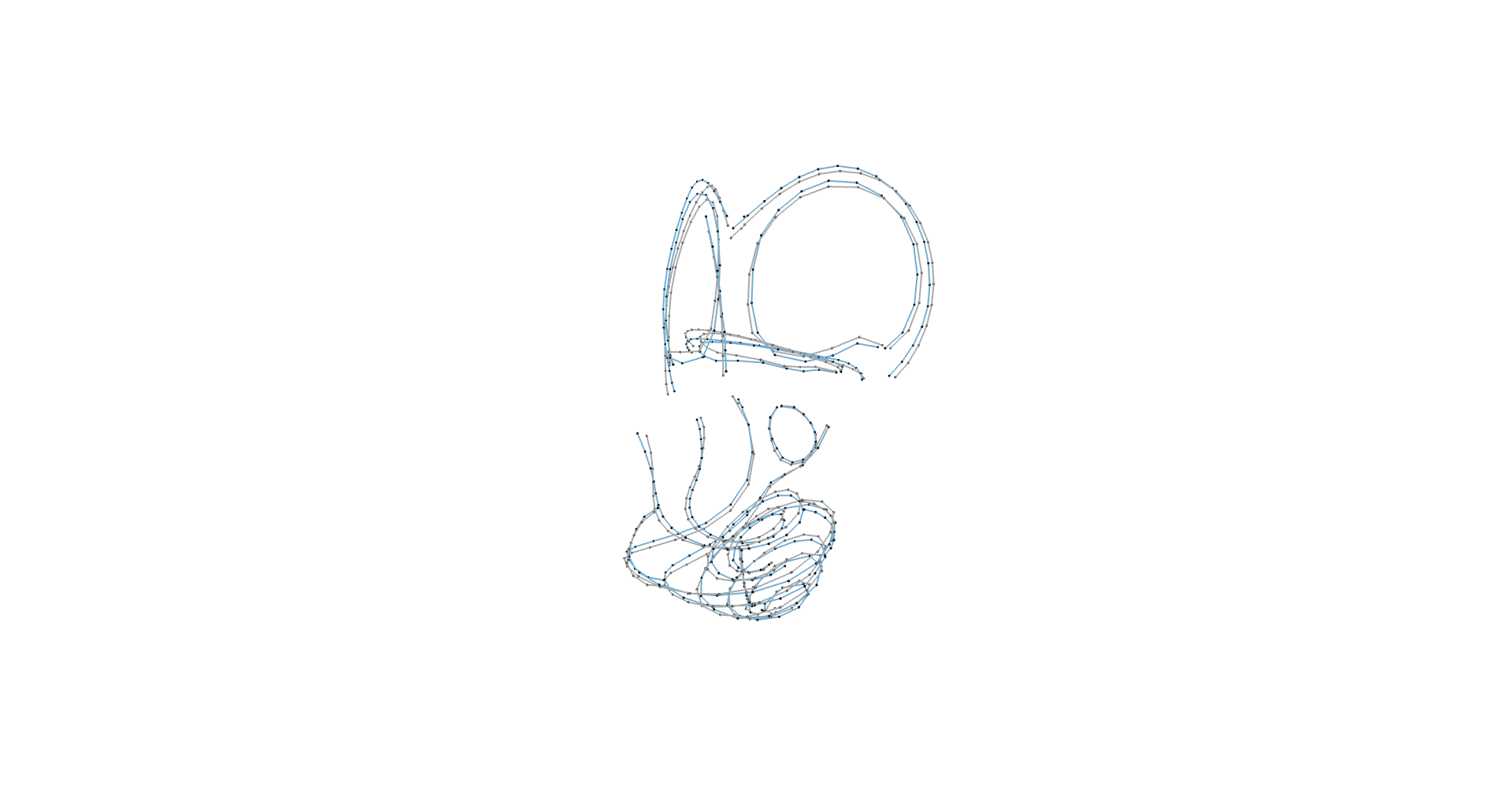

Supplement: Supplementary file 3 — Supplementary Data 1 [file 41467_2022_34656_MOESM3_ESM.zip › Supplementary data_1/Supplementary_material_1-1 Geometric morphometrics/CVA_306/mean_shapes_per_clade_CVA/Giraffidae-dl.png]

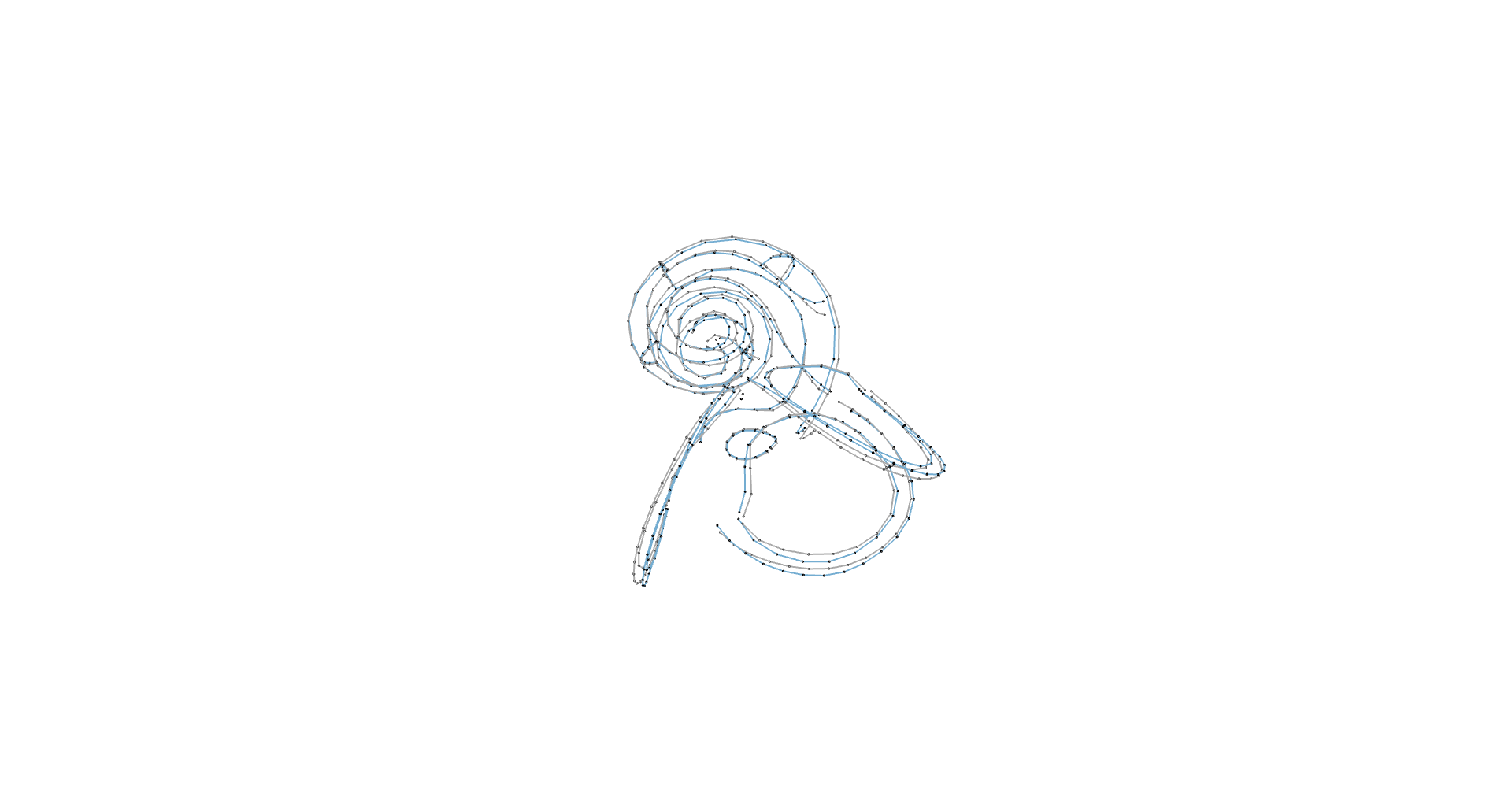

Supplement: Supplementary file 3 — Supplementary Data 1 [file 41467_2022_34656_MOESM3_ESM.zip › Supplementary data_1/Supplementary_material_1-1 Geometric morphometrics/CVA_306/mean_shapes_per_clade_CVA/Giraffidae-do.png]

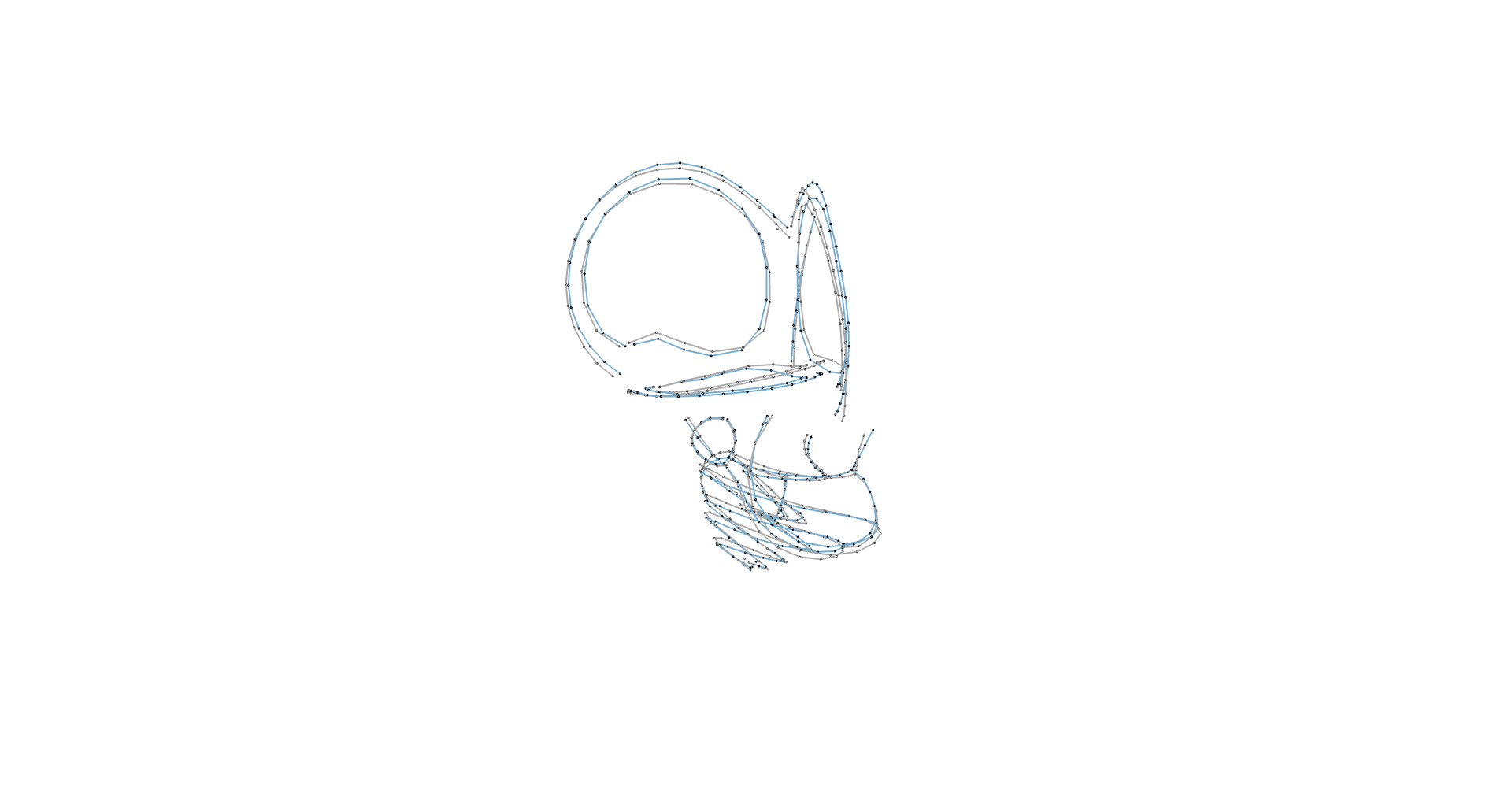

Supplement: Supplementary file 3 — Supplementary Data 1 [file 41467_2022_34656_MOESM3_ESM.zip › Supplementary data_1/Supplementary_material_1-1 Geometric morphometrics/CVA_306/mean_shapes_per_clade_CVA/Giraffidae-la.png]

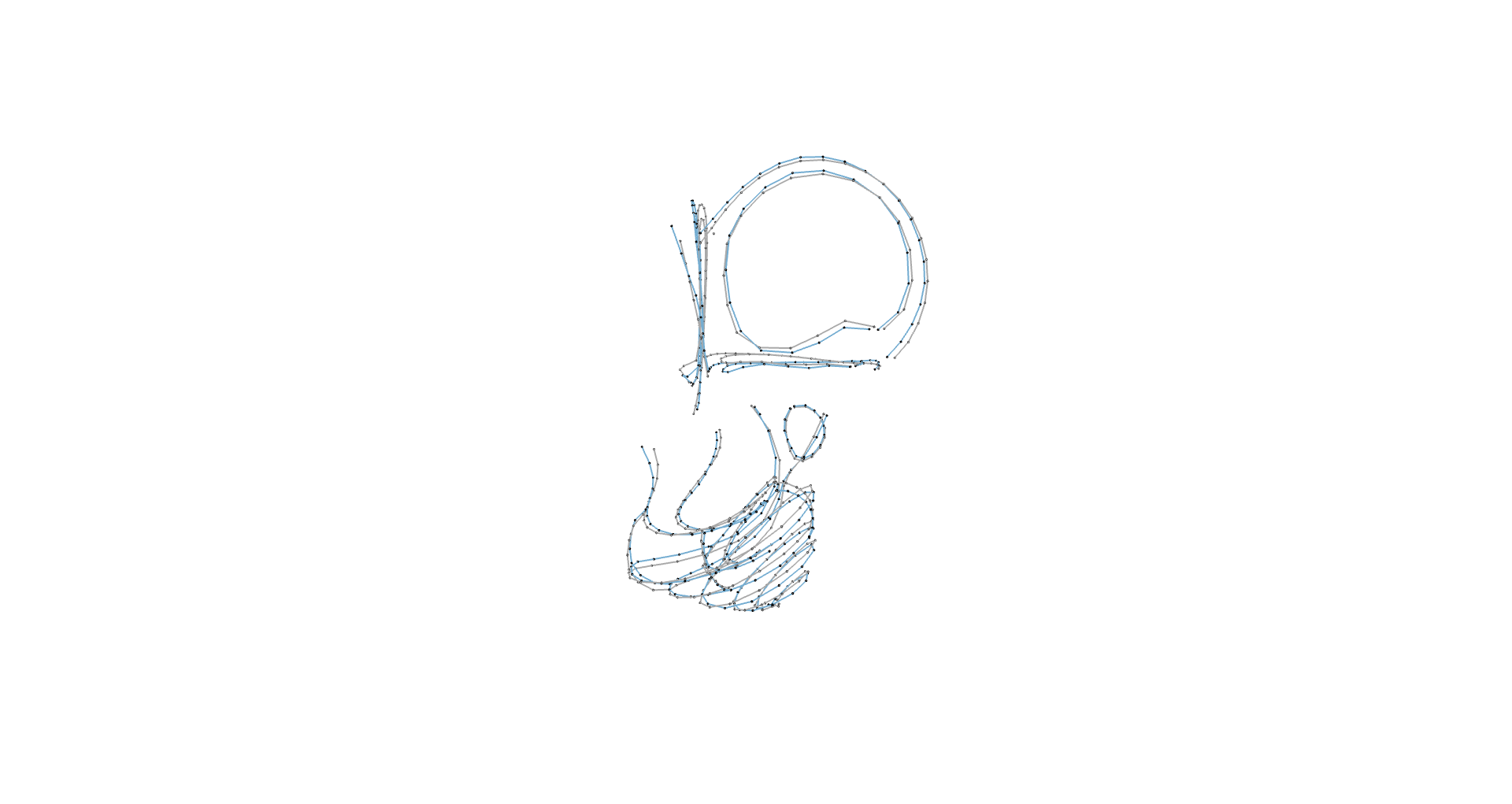

Supplement: Supplementary file 3 — Supplementary Data 1 [file 41467_2022_34656_MOESM3_ESM.zip › Supplementary data_1/Supplementary_material_1-1 Geometric morphometrics/CVA_306/mean_shapes_per_clade_CVA/Giraffidae-me.png]

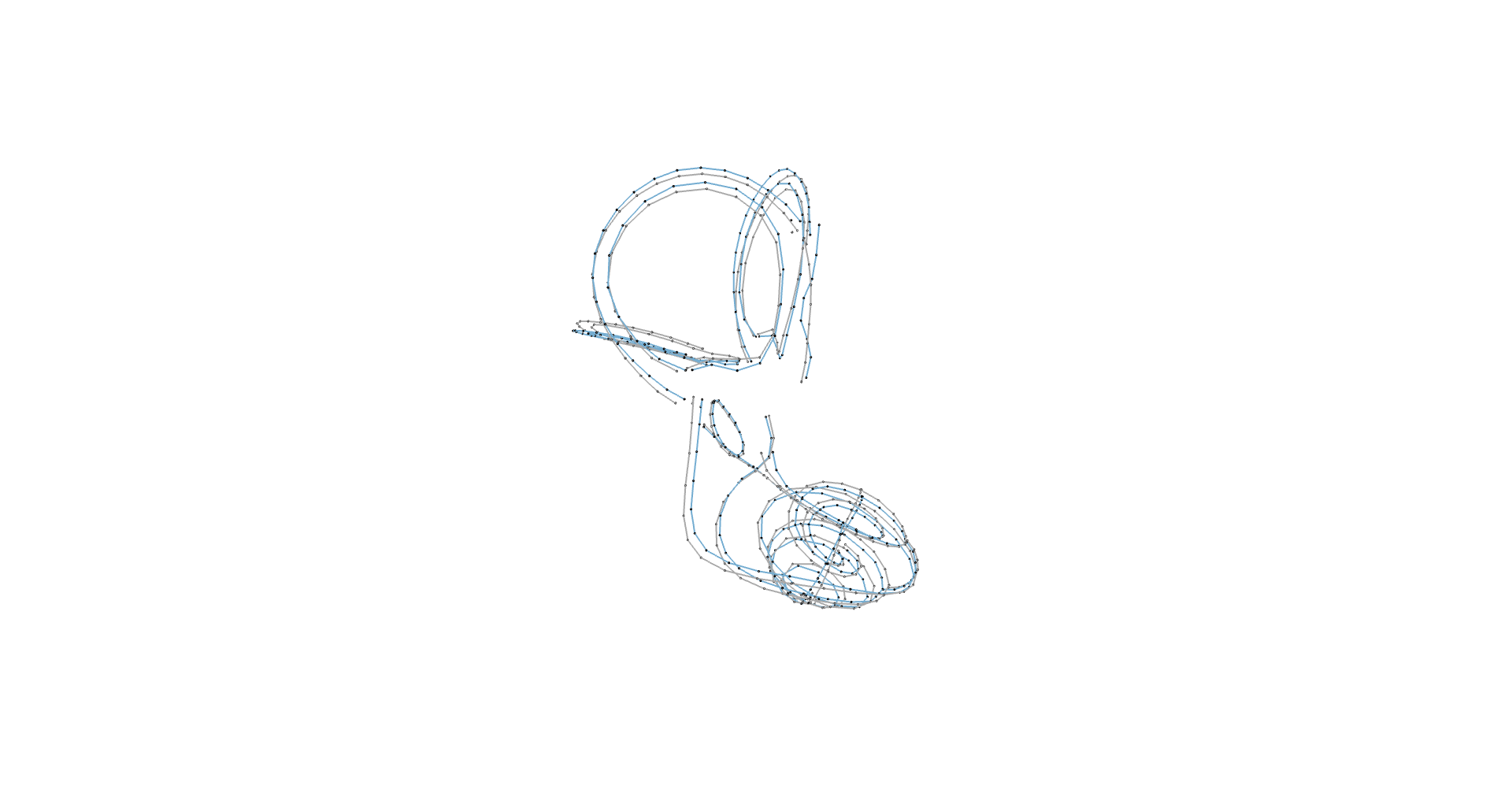

Supplement: Supplementary file 3 — Supplementary Data 1 [file 41467_2022_34656_MOESM3_ESM.zip › Supplementary data_1/Supplementary_material_1-1 Geometric morphometrics/CVA_306/mean_shapes_per_clade_CVA/Giraffidae-oc.png]

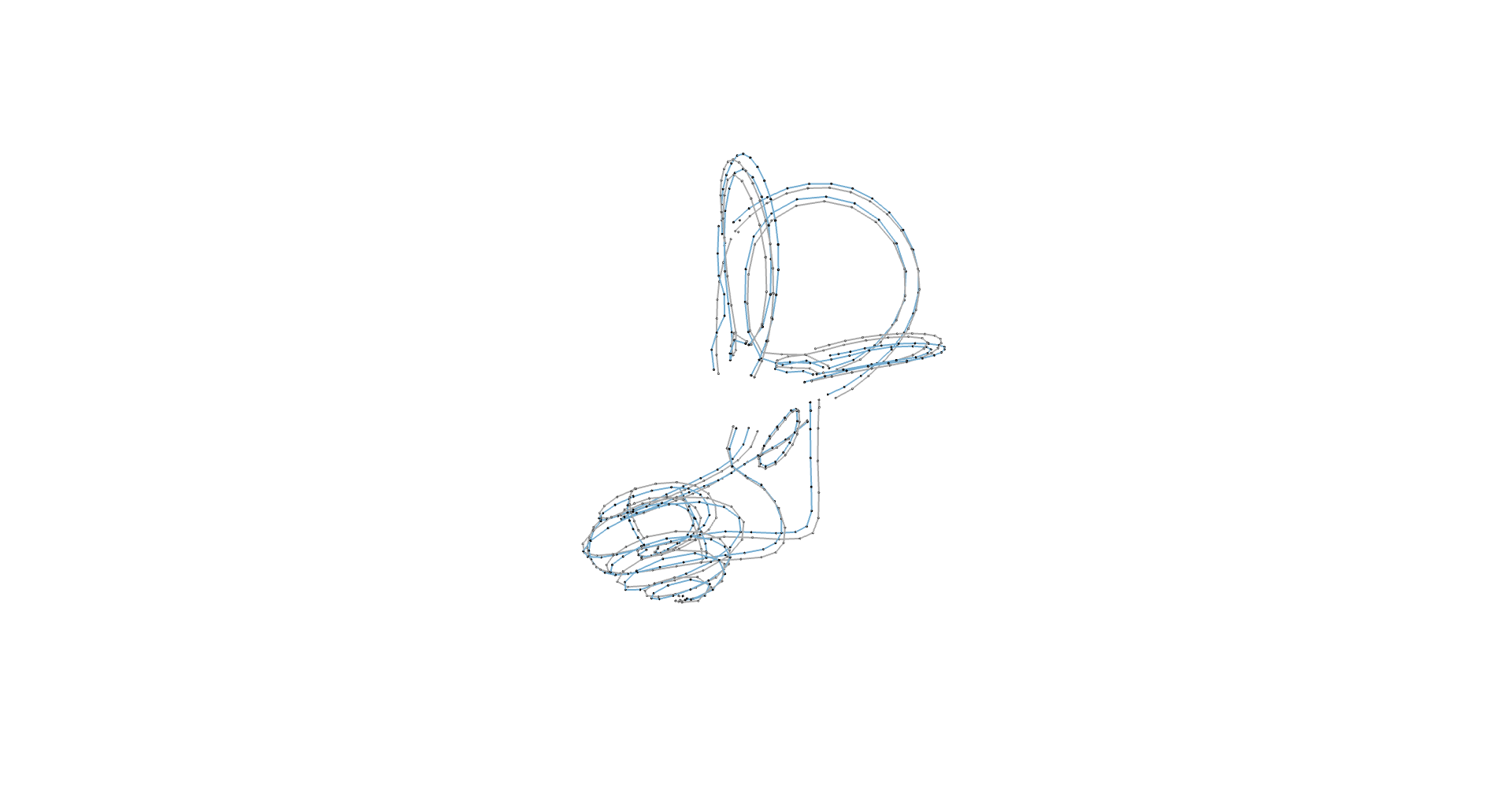

Supplement: Supplementary file 3 — Supplementary Data 1 [file 41467_2022_34656_MOESM3_ESM.zip › Supplementary data_1/Supplementary_material_1-1 Geometric morphometrics/CVA_306/mean_shapes_per_clade_CVA/Giraffidae-ro.png]

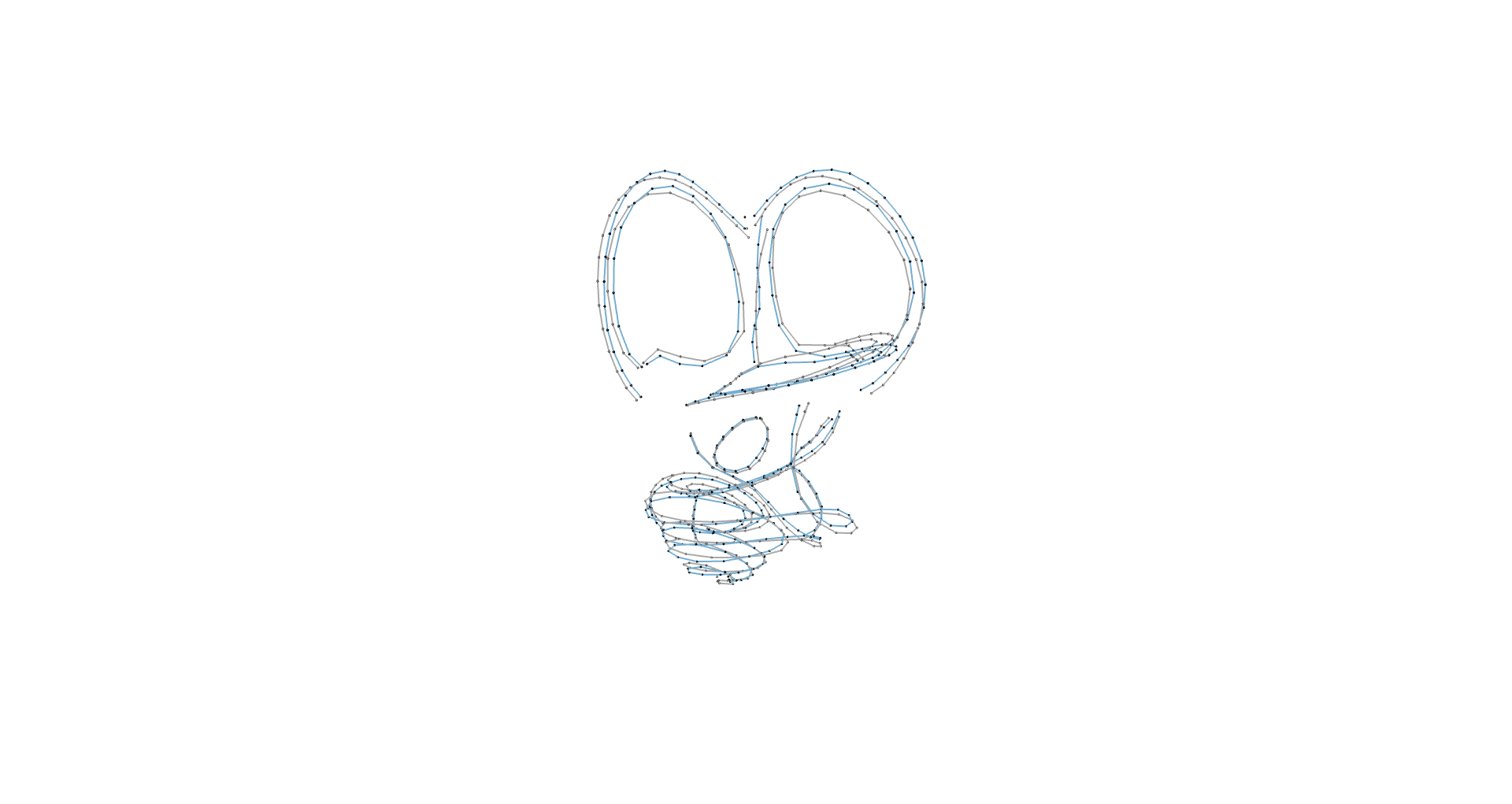

Supplement: Supplementary file 3 — Supplementary Data 1 [file 41467_2022_34656_MOESM3_ESM.zip › Supplementary data_1/Supplementary_material_1-1 Geometric morphometrics/CVA_306/mean_shapes_per_clade_CVA/Giraffidae-vl.png]

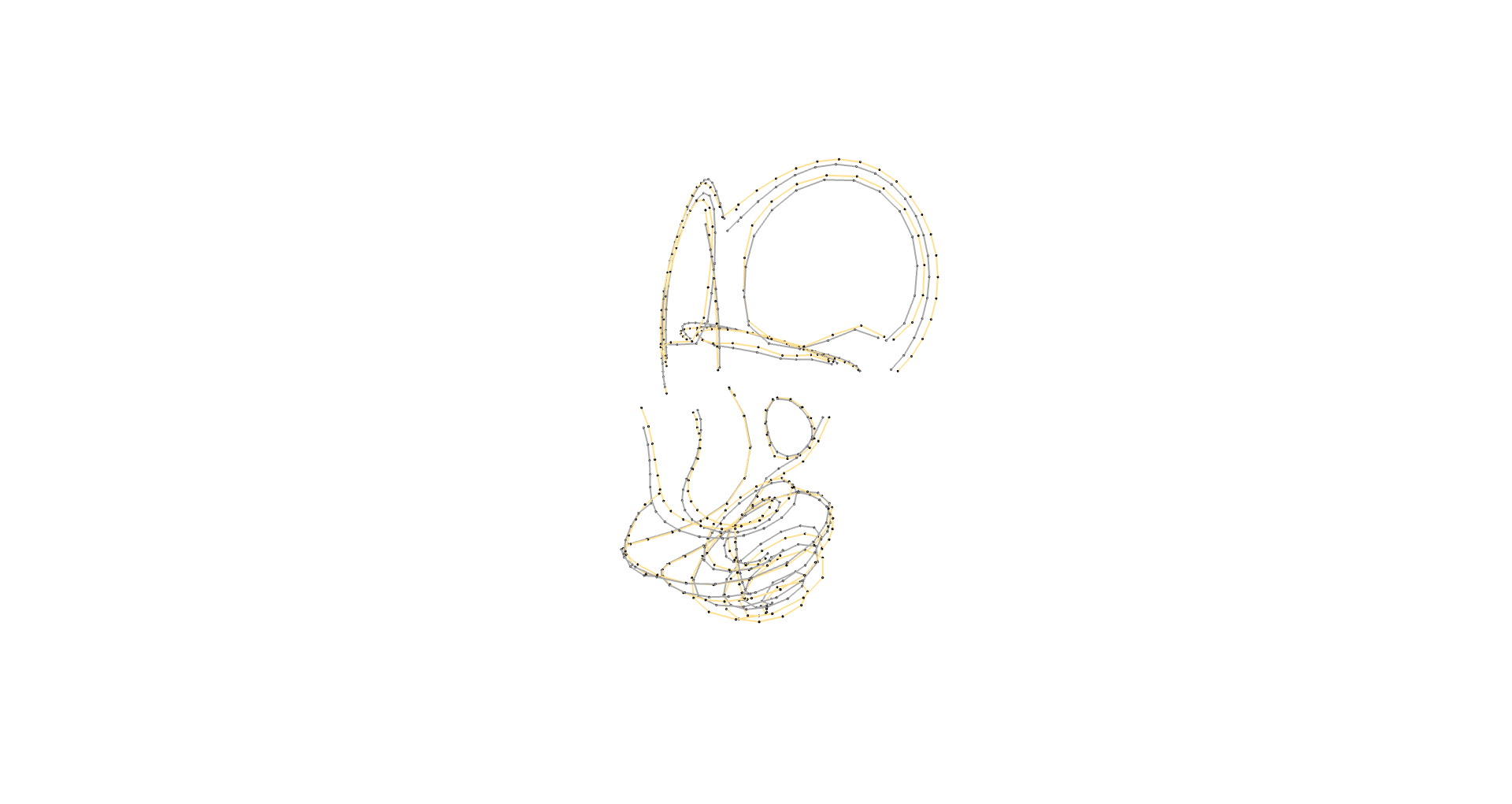

Supplement: Supplementary file 3 — Supplementary Data 1 [file 41467_2022_34656_MOESM3_ESM.zip › Supplementary data_1/Supplementary_material_1-1 Geometric morphometrics/CVA_306/mean_shapes_per_clade_CVA/Moschidae-dl.png]

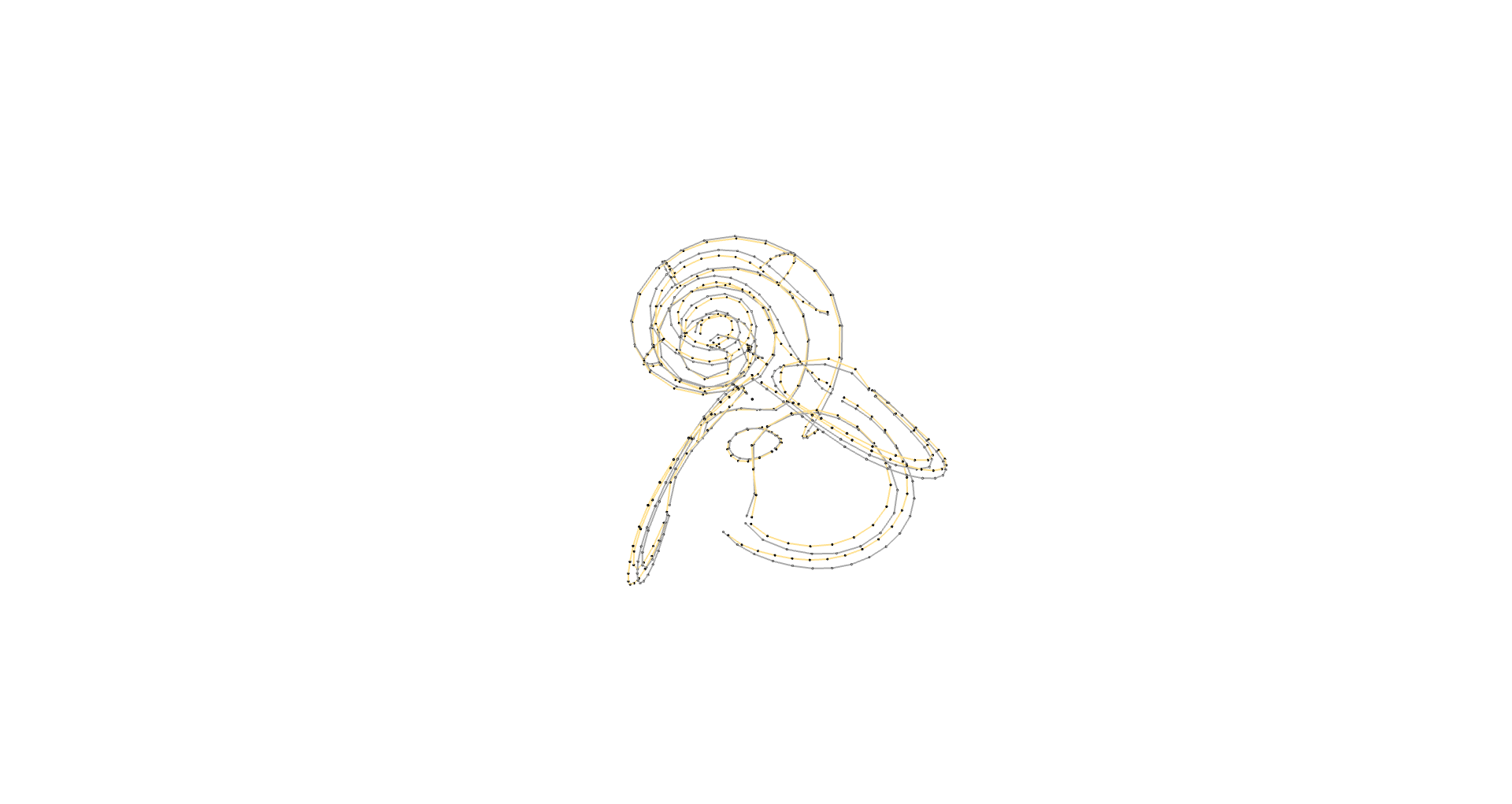

Supplement: Supplementary file 3 — Supplementary Data 1 [file 41467_2022_34656_MOESM3_ESM.zip › Supplementary data_1/Supplementary_material_1-1 Geometric morphometrics/CVA_306/mean_shapes_per_clade_CVA/Moschidae-do.png]

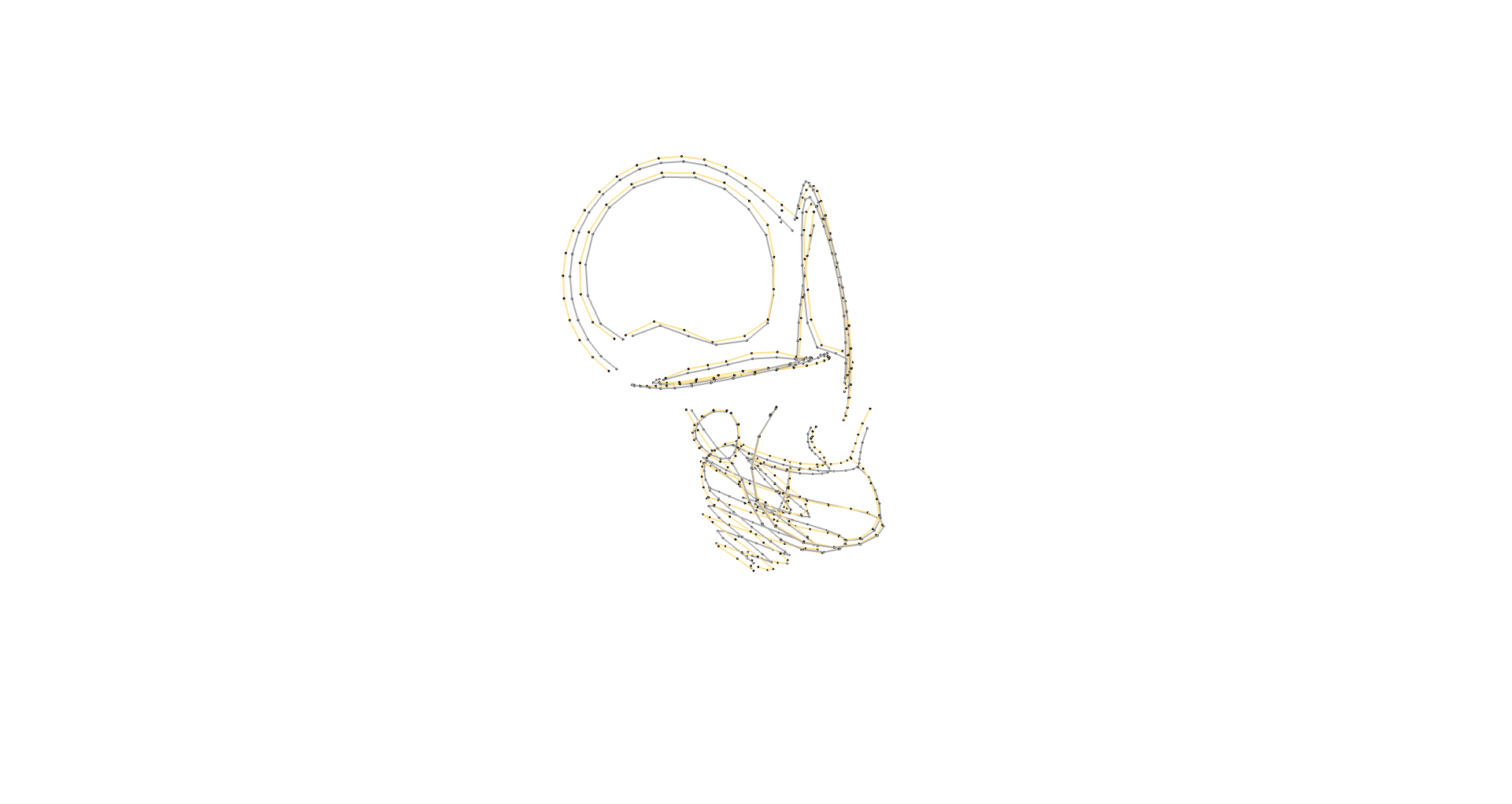

Supplement: Supplementary file 3 — Supplementary Data 1 [file 41467_2022_34656_MOESM3_ESM.zip › Supplementary data_1/Supplementary_material_1-1 Geometric morphometrics/CVA_306/mean_shapes_per_clade_CVA/Moschidae-la.png]

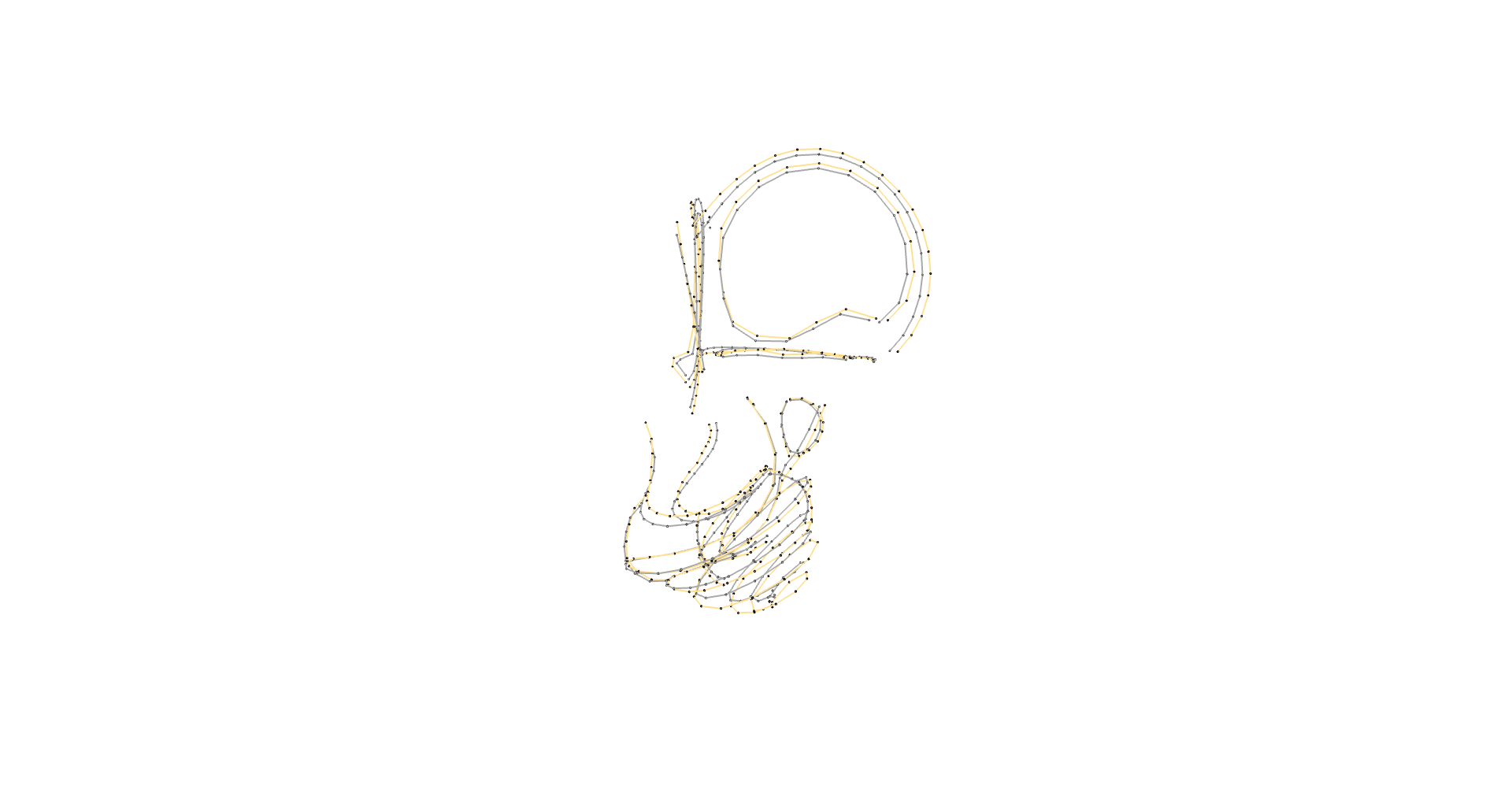

Supplement: Supplementary file 3 — Supplementary Data 1 [file 41467_2022_34656_MOESM3_ESM.zip › Supplementary data_1/Supplementary_material_1-1 Geometric morphometrics/CVA_306/mean_shapes_per_clade_CVA/Moschidae-me.png]

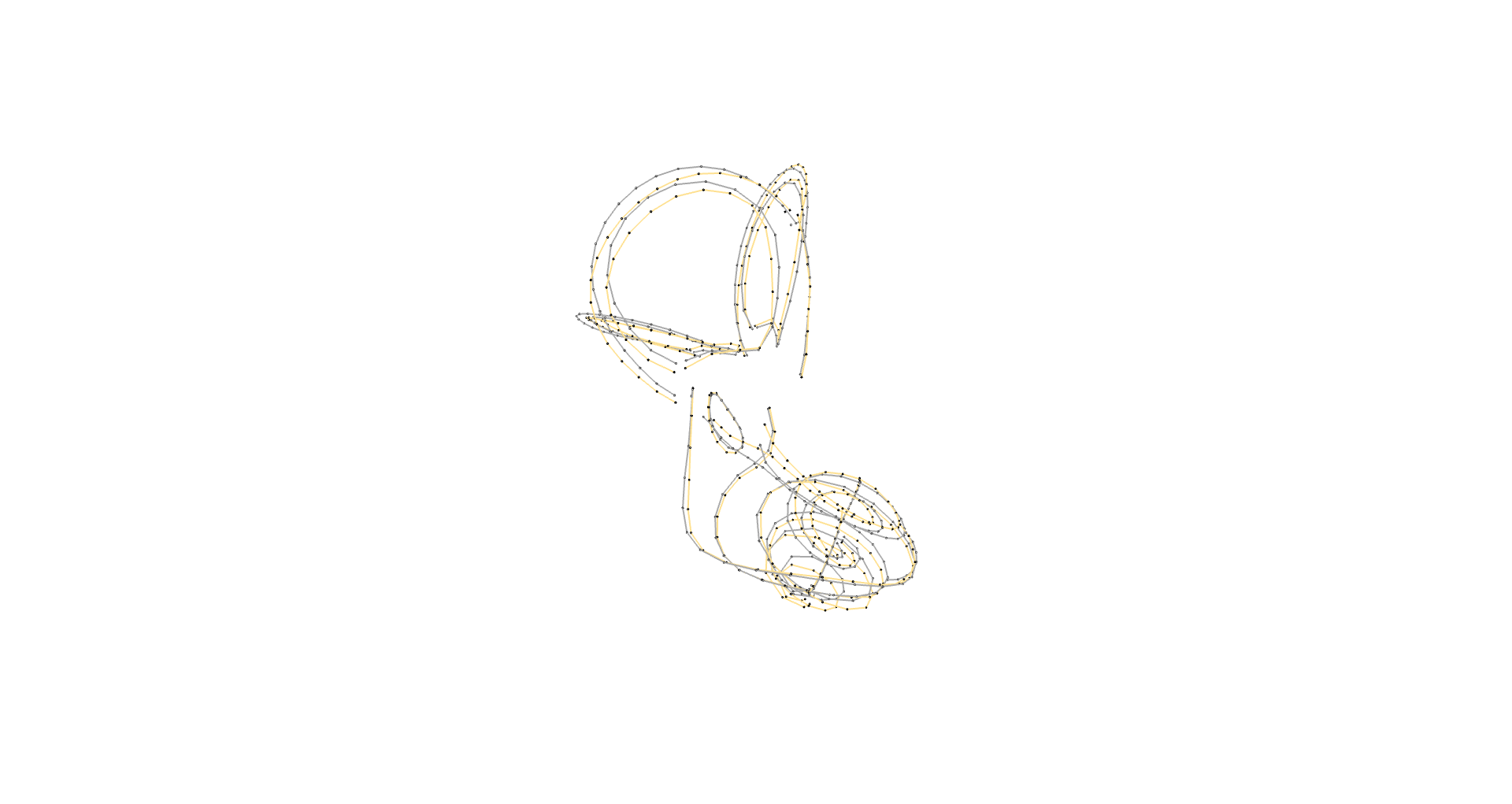

Supplement: Supplementary file 3 — Supplementary Data 1 [file 41467_2022_34656_MOESM3_ESM.zip › Supplementary data_1/Supplementary_material_1-1 Geometric morphometrics/CVA_306/mean_shapes_per_clade_CVA/Moschidae-oc.png]

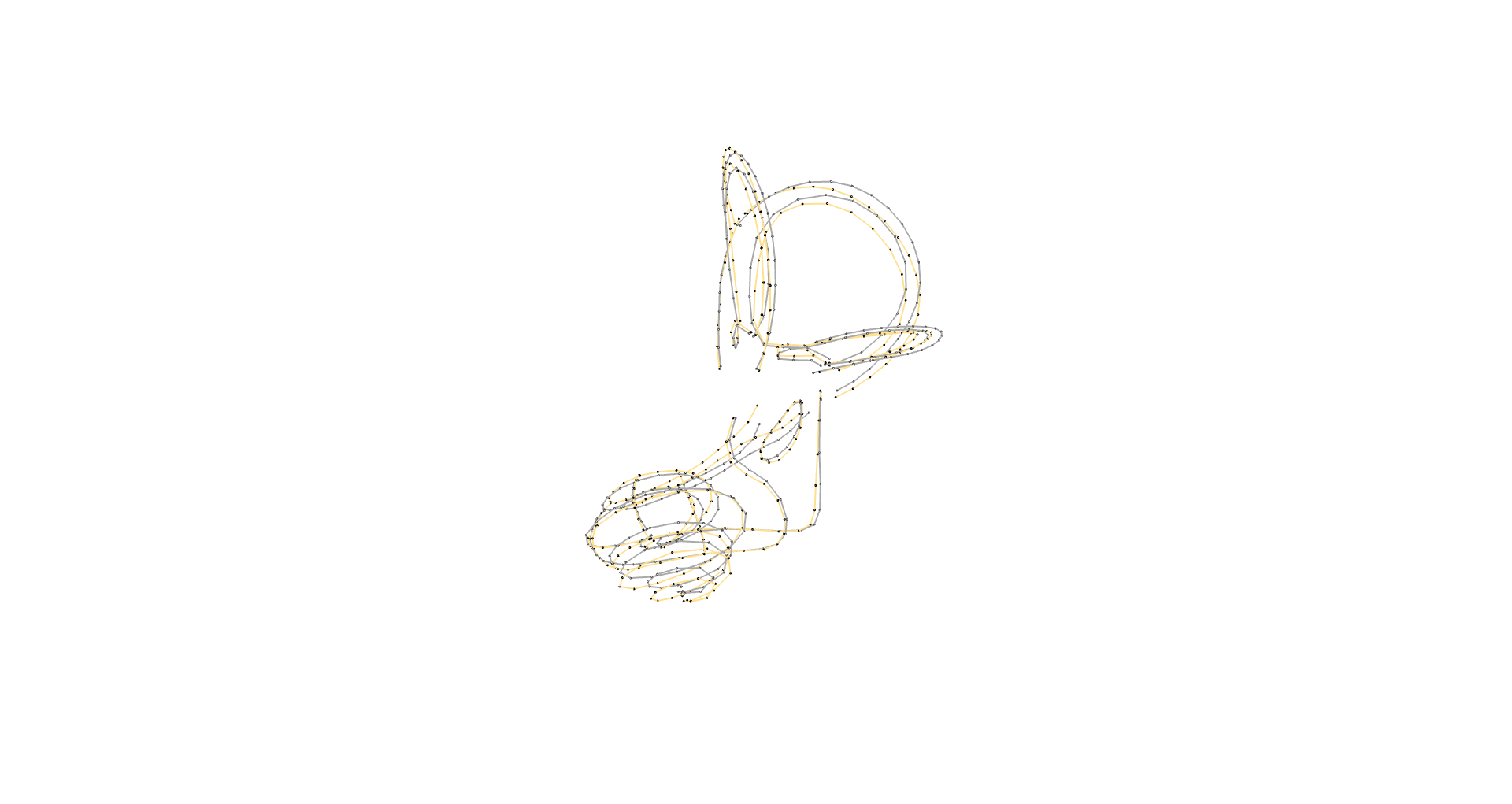

Supplement: Supplementary file 3 — Supplementary Data 1 [file 41467_2022_34656_MOESM3_ESM.zip › Supplementary data_1/Supplementary_material_1-1 Geometric morphometrics/CVA_306/mean_shapes_per_clade_CVA/Moschidae-ro.png]

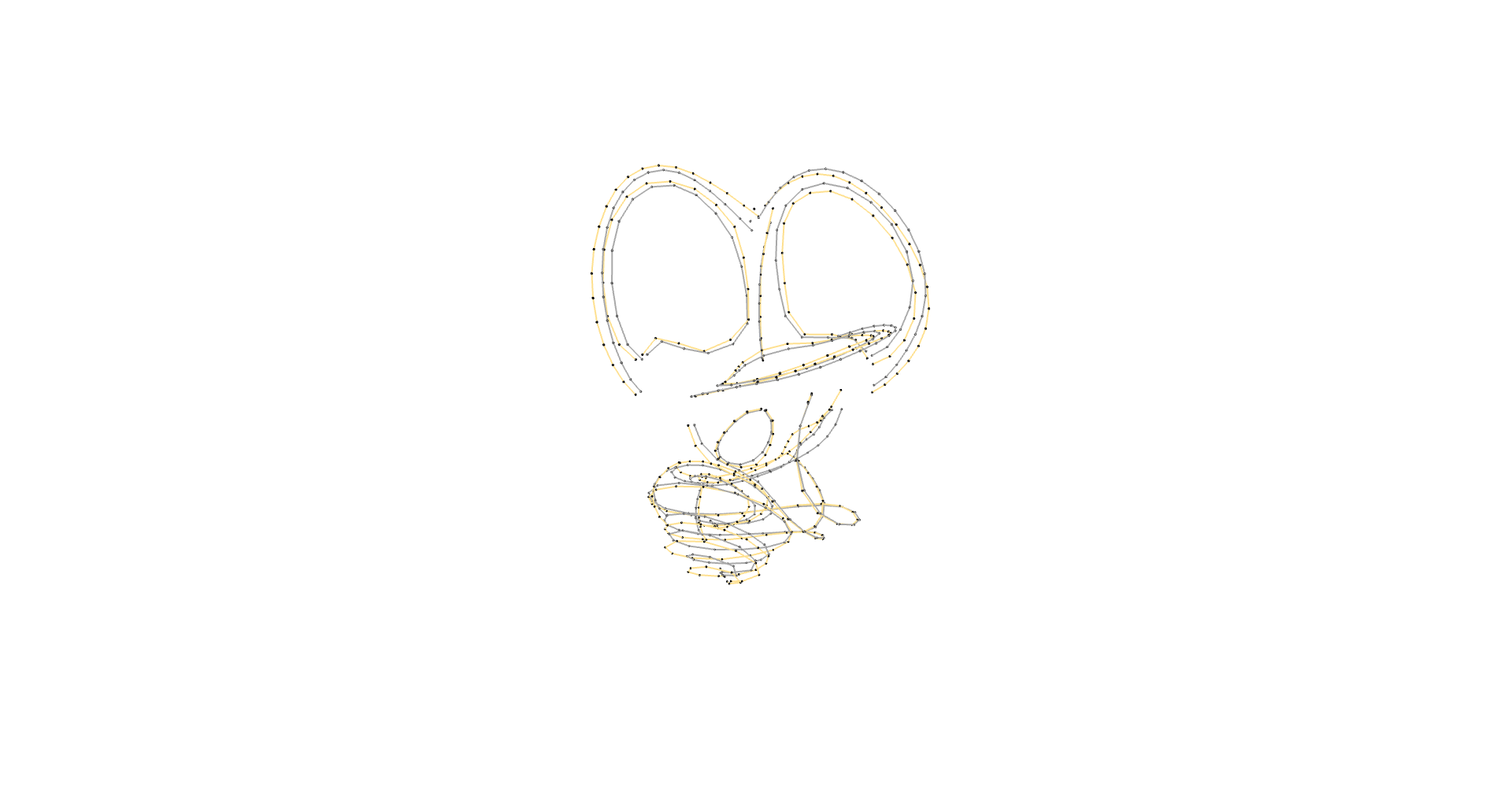

Supplement: Supplementary file 3 — Supplementary Data 1 [file 41467_2022_34656_MOESM3_ESM.zip › Supplementary data_1/Supplementary_material_1-1 Geometric morphometrics/CVA_306/mean_shapes_per_clade_CVA/Moschidae-vl.png]

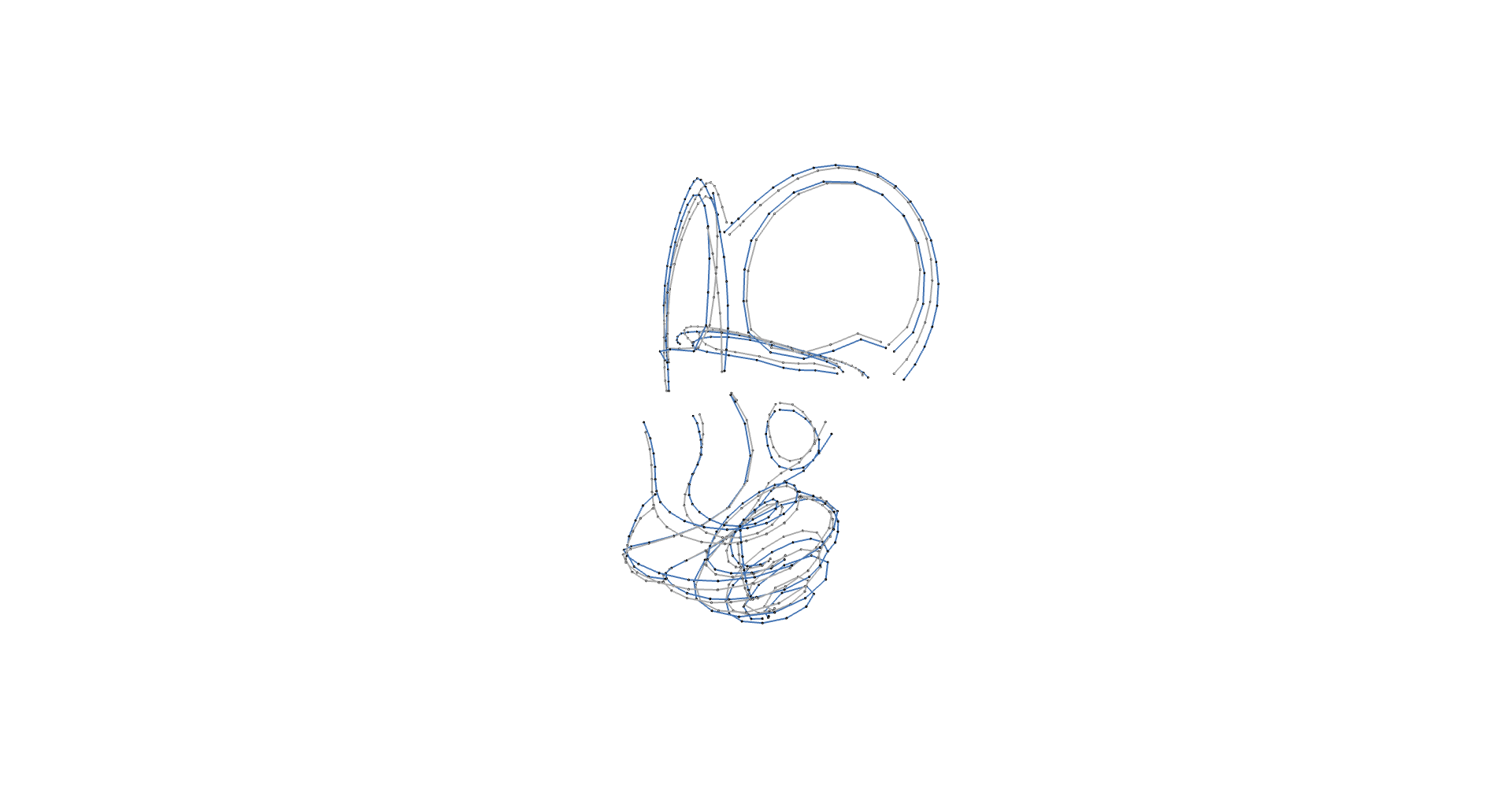

Supplement: Supplementary file 3 — Supplementary Data 1 [file 41467_2022_34656_MOESM3_ESM.zip › Supplementary data_1/Supplementary_material_1-1 Geometric morphometrics/CVA_306/mean_shapes_per_clade_CVA/Stem_Pecora-dl.png]

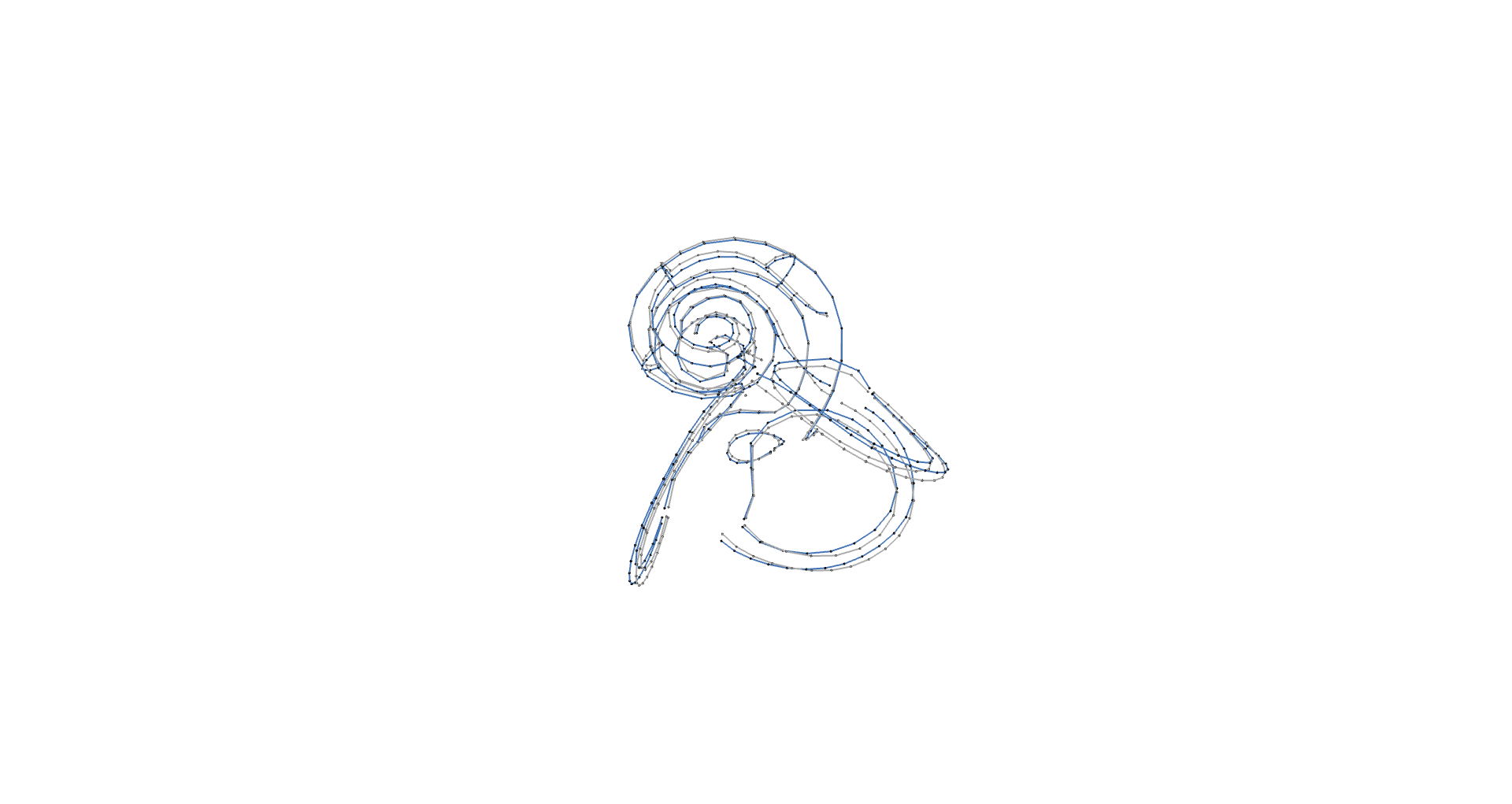

Supplement: Supplementary file 3 — Supplementary Data 1 [file 41467_2022_34656_MOESM3_ESM.zip › Supplementary data_1/Supplementary_material_1-1 Geometric morphometrics/CVA_306/mean_shapes_per_clade_CVA/Stem_Pecora-do.png]

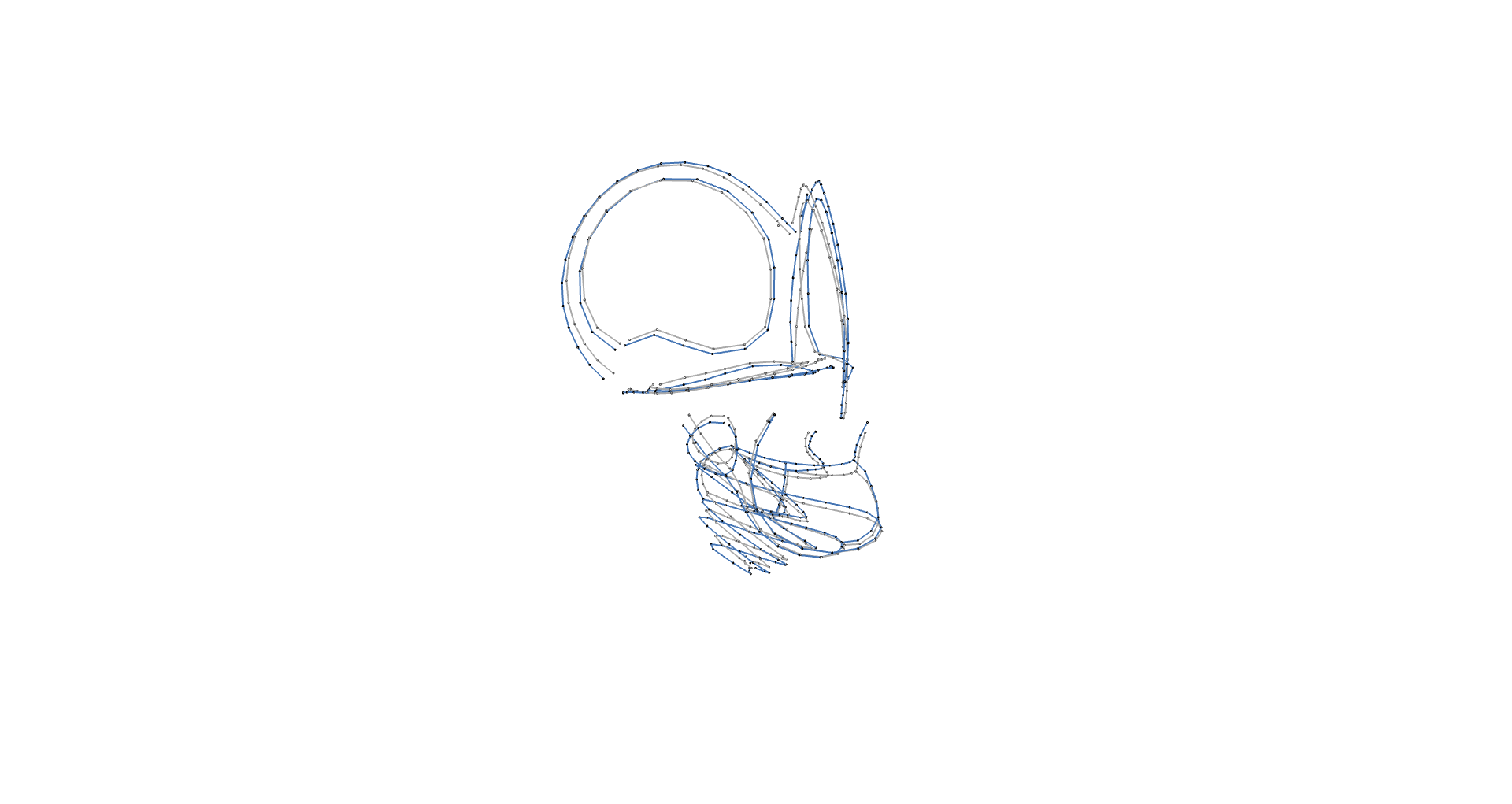

Supplement: Supplementary file 3 — Supplementary Data 1 [file 41467_2022_34656_MOESM3_ESM.zip › Supplementary data_1/Supplementary_material_1-1 Geometric morphometrics/CVA_306/mean_shapes_per_clade_CVA/Stem_Pecora-la.png]

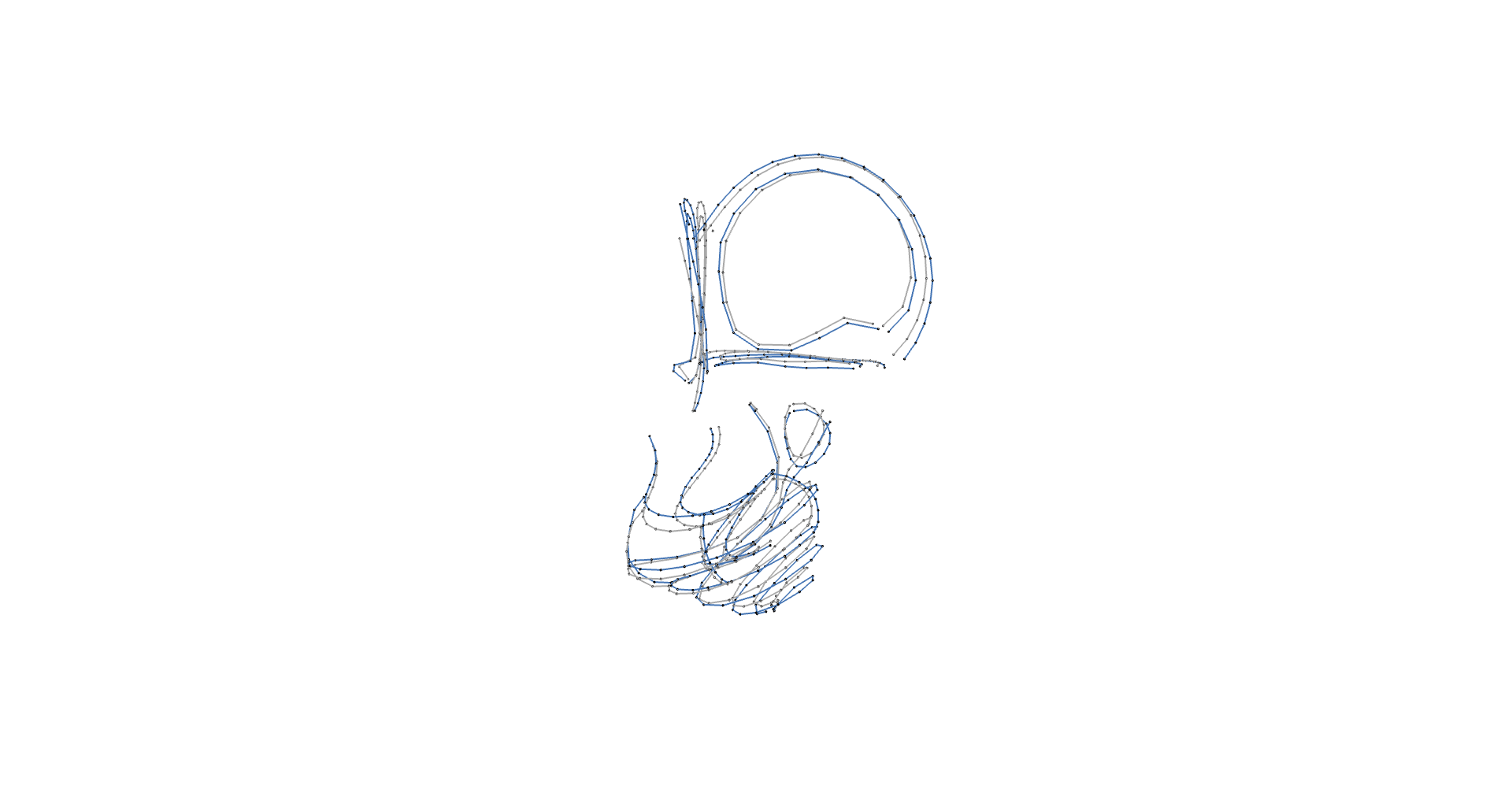

Supplement: Supplementary file 3 — Supplementary Data 1 [file 41467_2022_34656_MOESM3_ESM.zip › Supplementary data_1/Supplementary_material_1-1 Geometric morphometrics/CVA_306/mean_shapes_per_clade_CVA/Stem_Pecora-me.png]

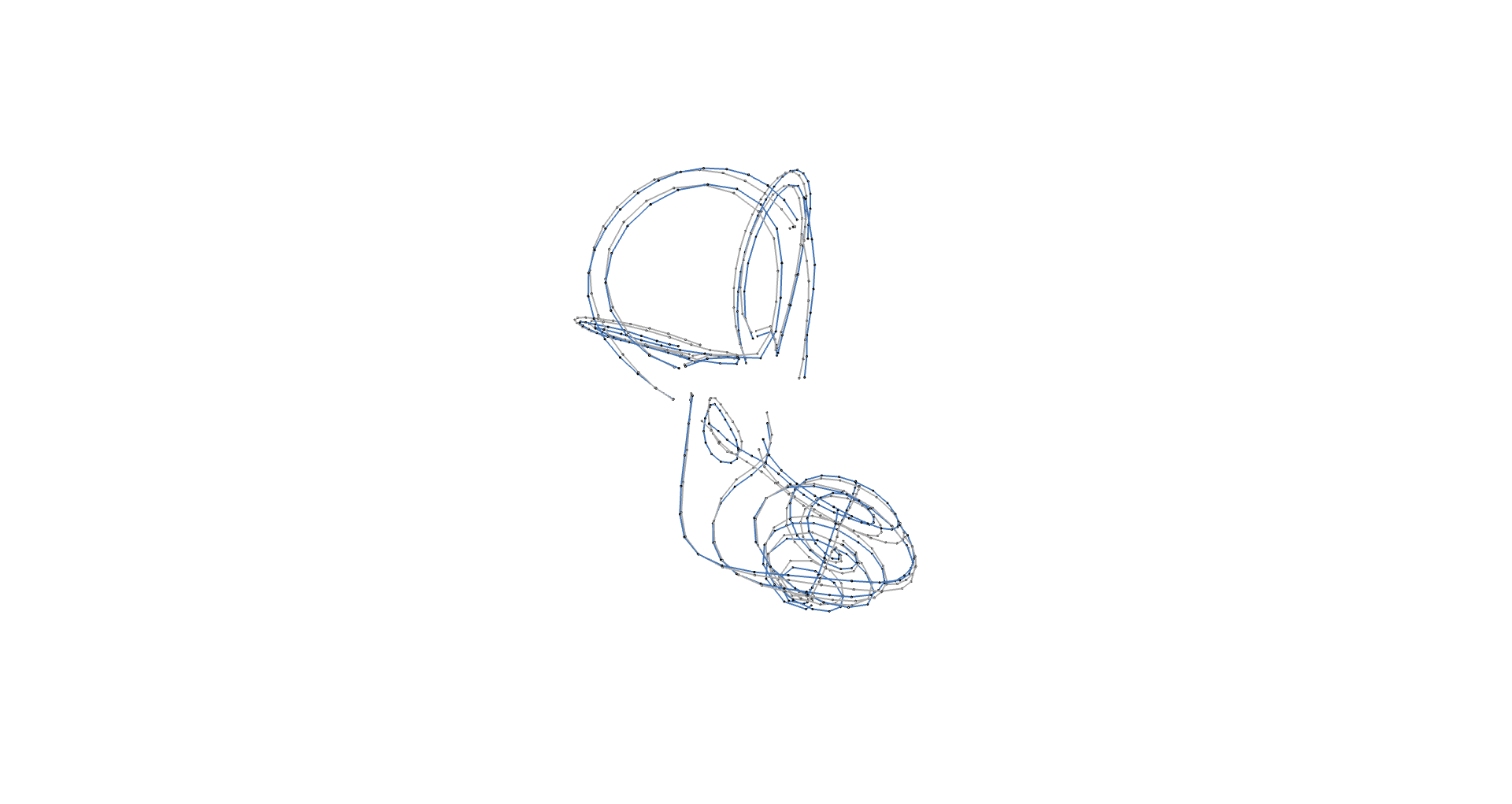

Supplement: Supplementary file 3 — Supplementary Data 1 [file 41467_2022_34656_MOESM3_ESM.zip › Supplementary data_1/Supplementary_material_1-1 Geometric morphometrics/CVA_306/mean_shapes_per_clade_CVA/Stem_Pecora-oc.png]

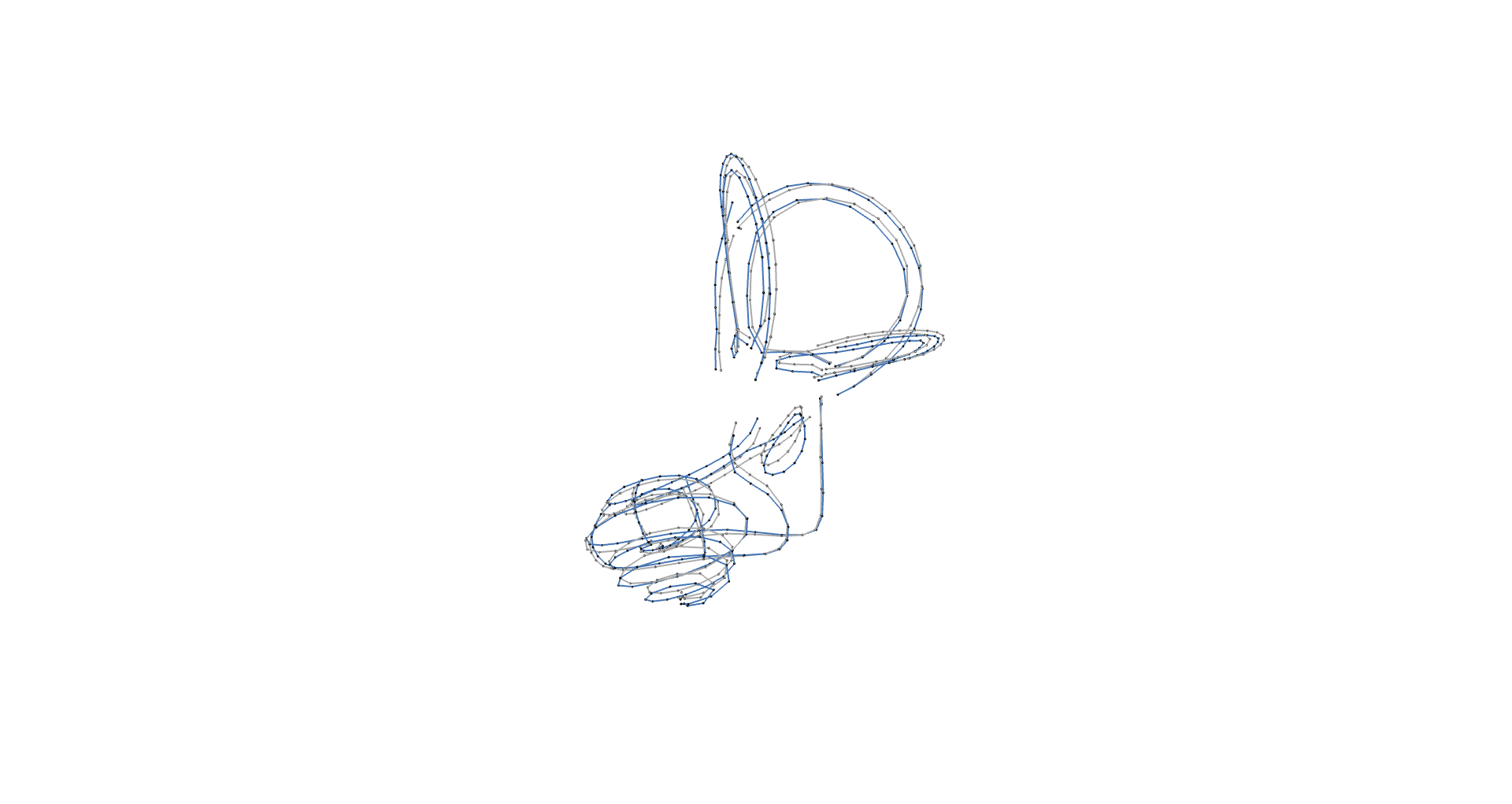

Supplement: Supplementary file 3 — Supplementary Data 1 [file 41467_2022_34656_MOESM3_ESM.zip › Supplementary data_1/Supplementary_material_1-1 Geometric morphometrics/CVA_306/mean_shapes_per_clade_CVA/Stem_Pecora-ro.png]

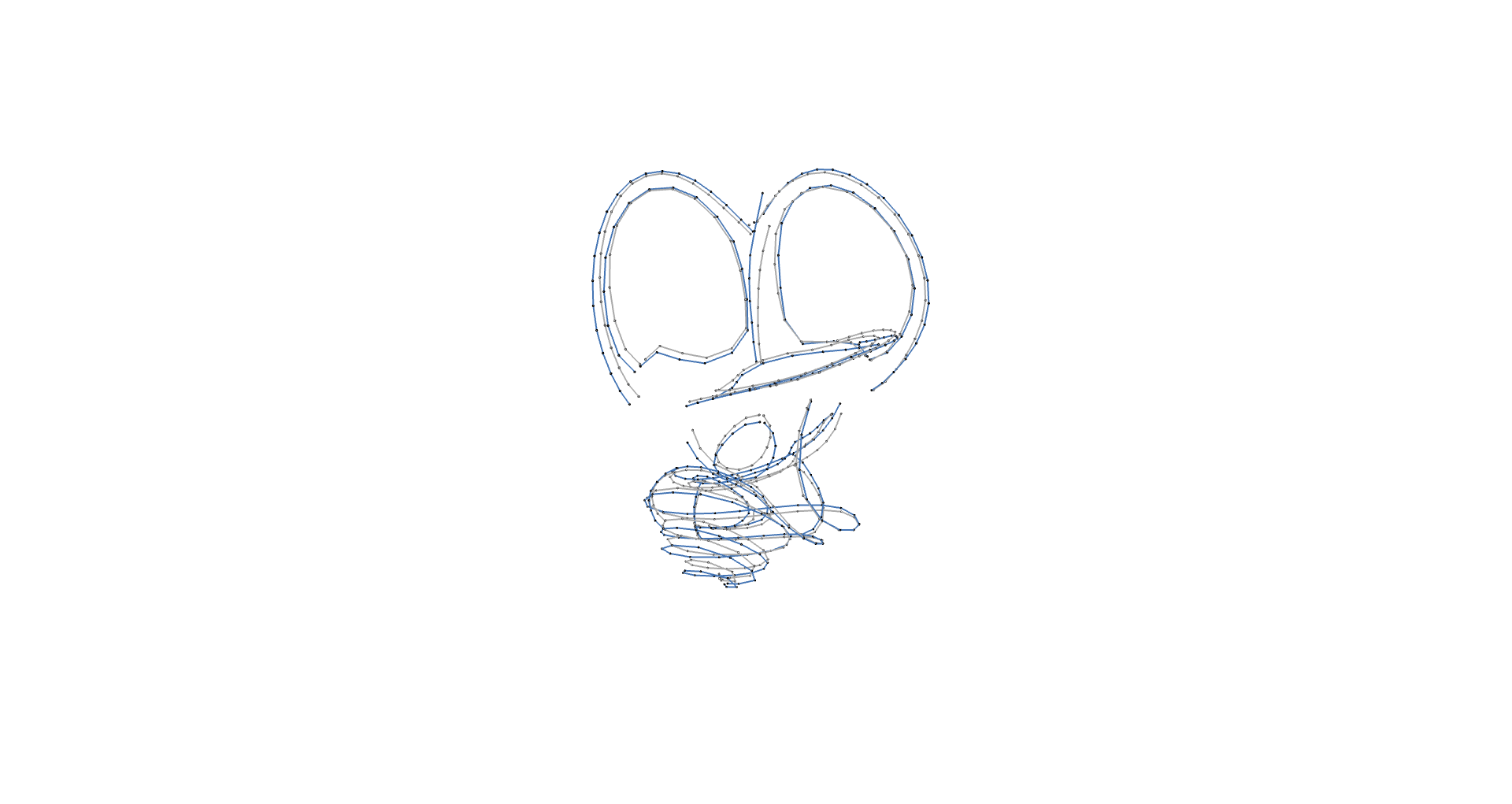

Supplement: Supplementary file 3 — Supplementary Data 1 [file 41467_2022_34656_MOESM3_ESM.zip › Supplementary data_1/Supplementary_material_1-1 Geometric morphometrics/CVA_306/mean_shapes_per_clade_CVA/Stem_Pecora-vl.png]

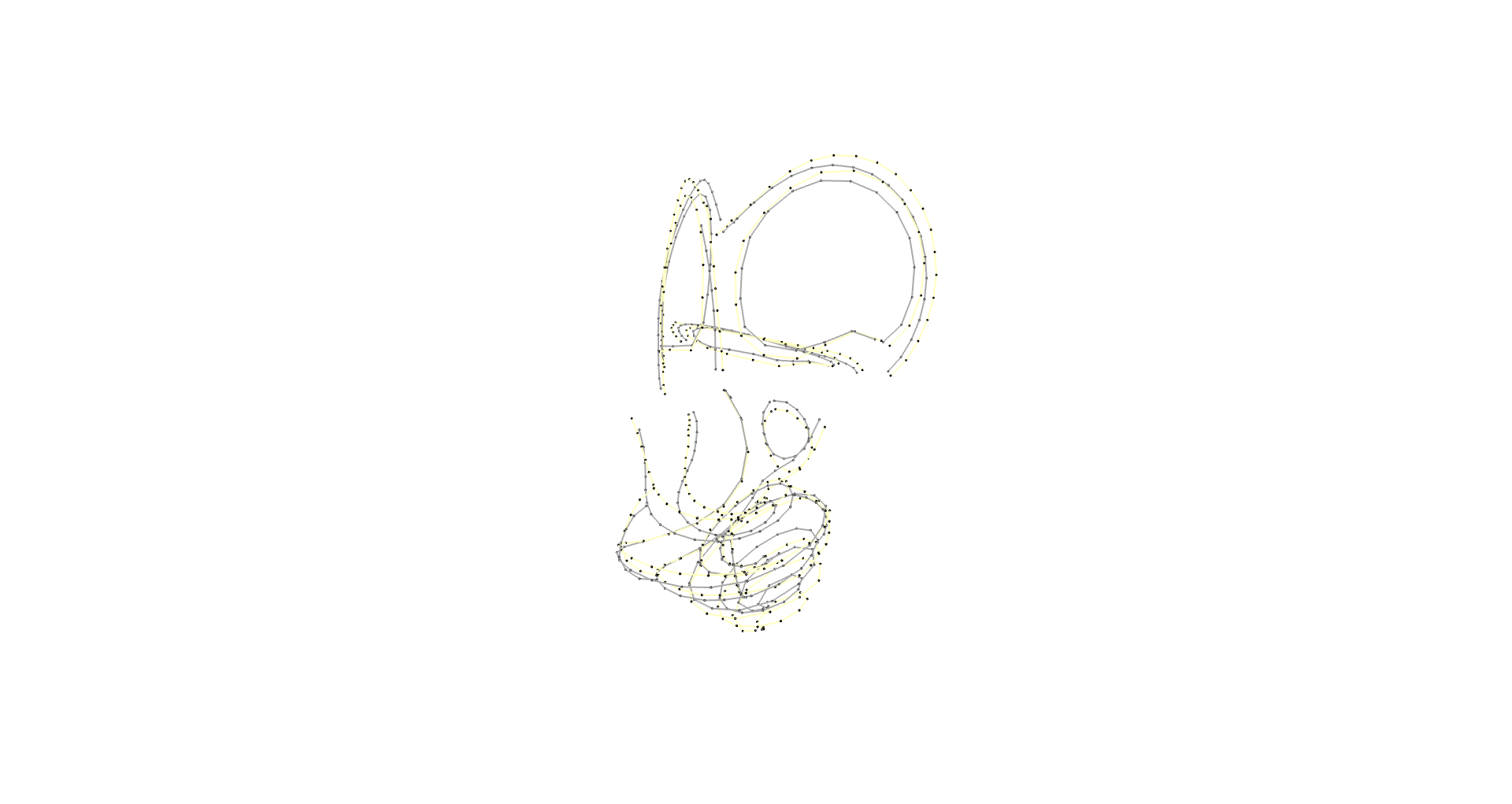

Supplement: Supplementary file 3 — Supplementary Data 1 [file 41467_2022_34656_MOESM3_ESM.zip › Supplementary data_1/Supplementary_material_1-1 Geometric morphometrics/CVA_306/mean_shapes_per_clade_CVA/Stem_Ruminantia-dl.png]

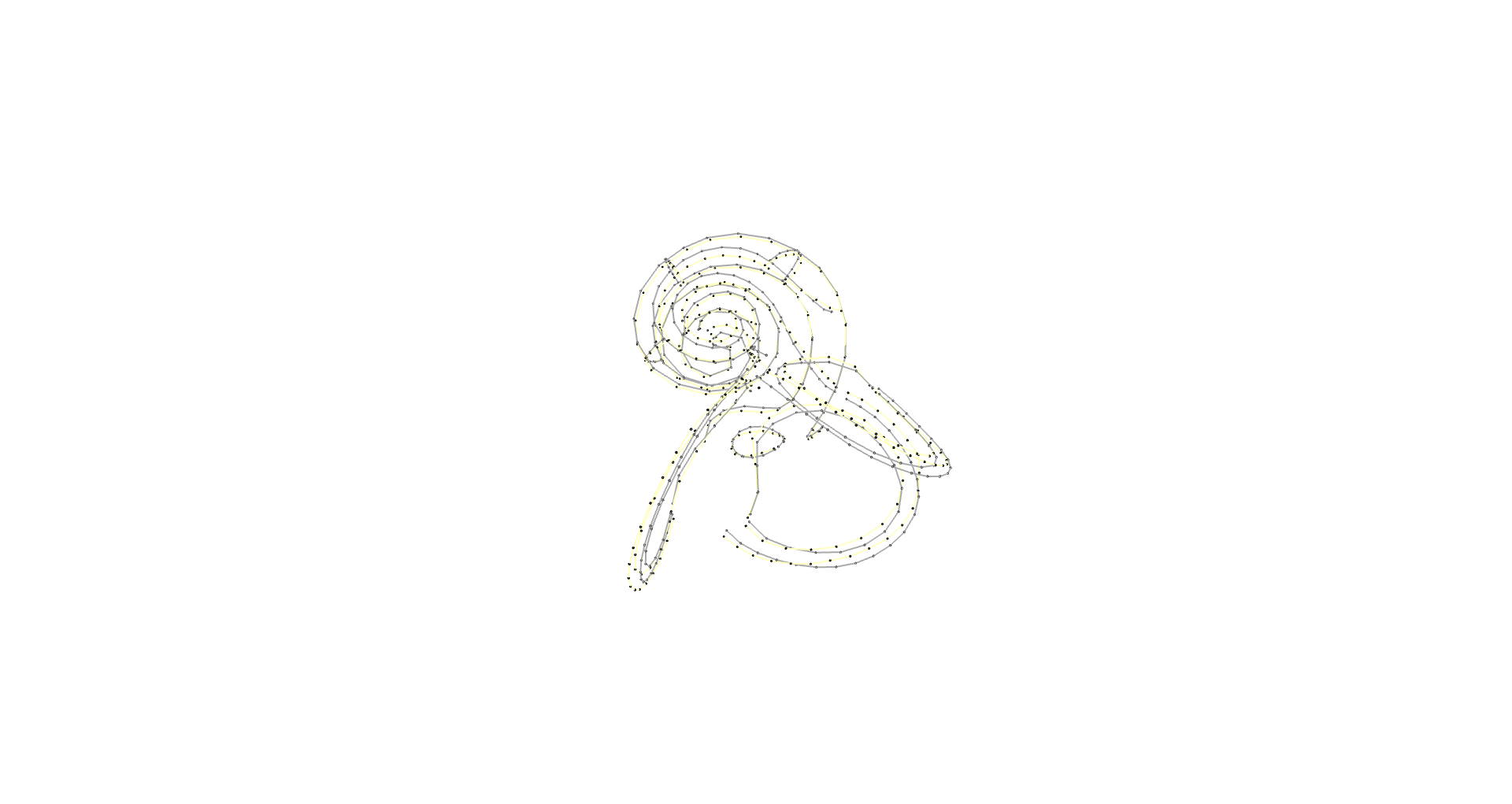

Supplement: Supplementary file 3 — Supplementary Data 1 [file 41467_2022_34656_MOESM3_ESM.zip › Supplementary data_1/Supplementary_material_1-1 Geometric morphometrics/CVA_306/mean_shapes_per_clade_CVA/Stem_Ruminantia-do.png]

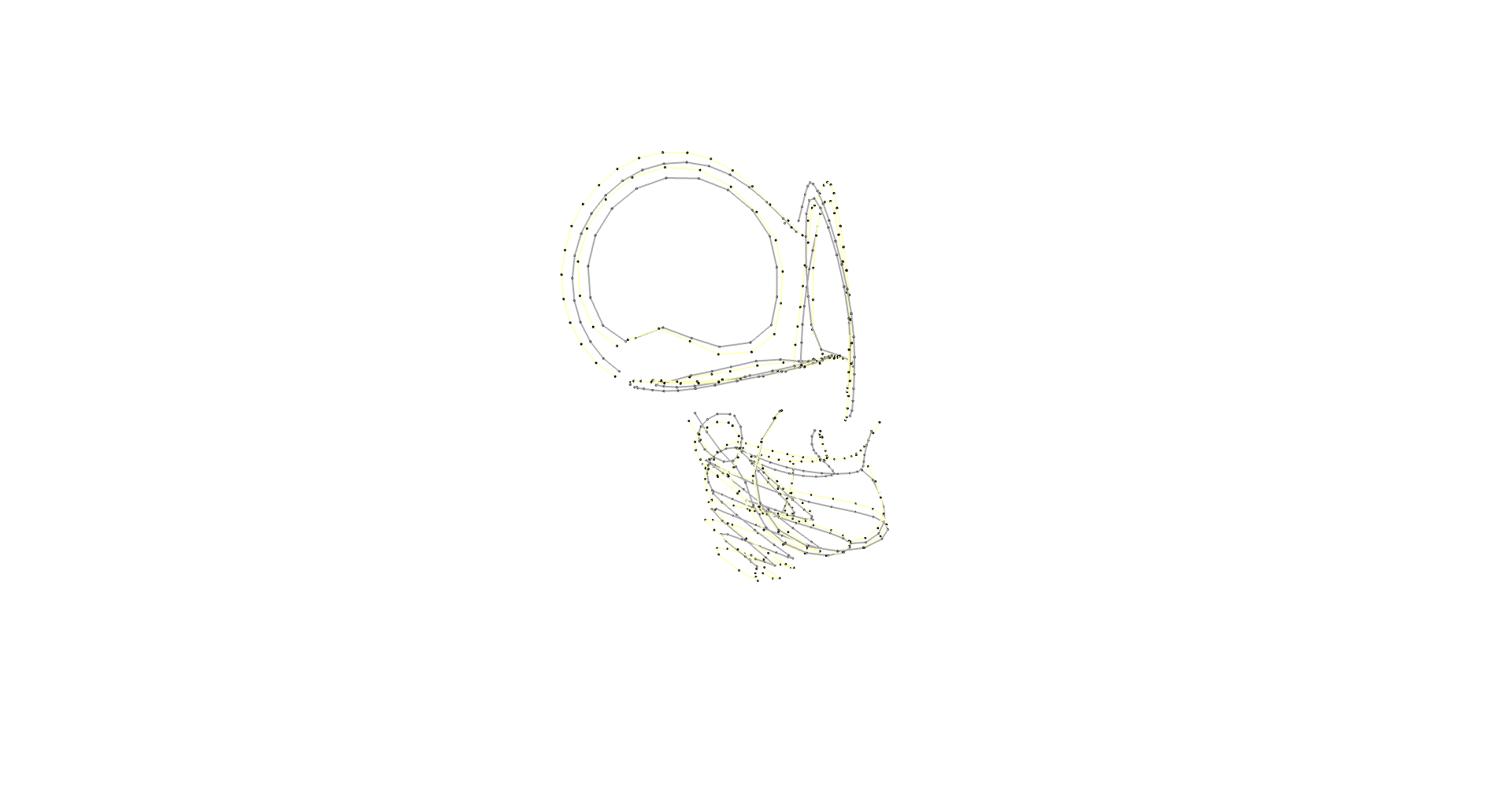

Supplement: Supplementary file 3 — Supplementary Data 1 [file 41467_2022_34656_MOESM3_ESM.zip › Supplementary data_1/Supplementary_material_1-1 Geometric morphometrics/CVA_306/mean_shapes_per_clade_CVA/Stem_Ruminantia-la.png]

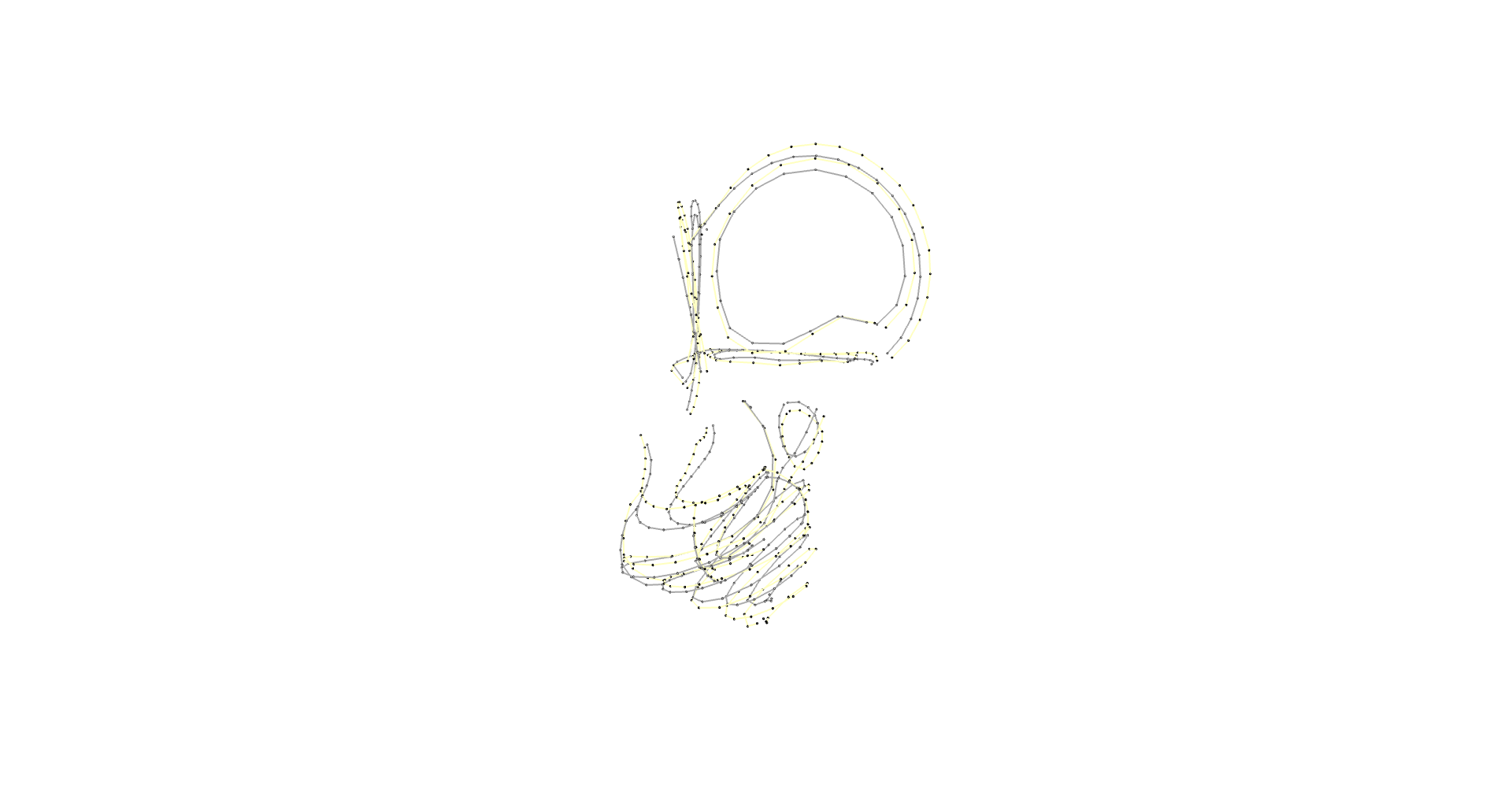

Supplement: Supplementary file 3 — Supplementary Data 1 [file 41467_2022_34656_MOESM3_ESM.zip › Supplementary data_1/Supplementary_material_1-1 Geometric morphometrics/CVA_306/mean_shapes_per_clade_CVA/Stem_Ruminantia-me.png]

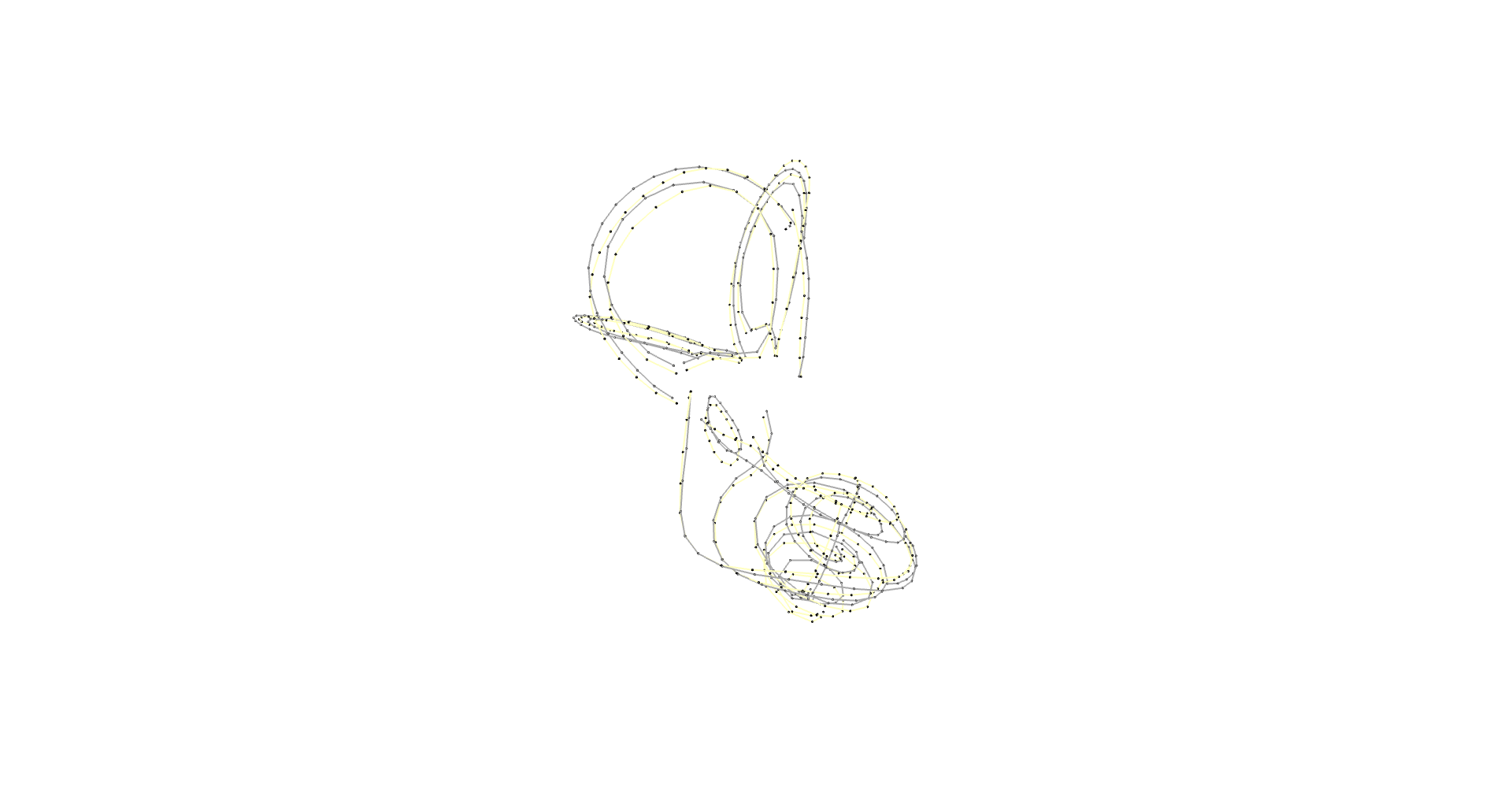

Supplement: Supplementary file 3 — Supplementary Data 1 [file 41467_2022_34656_MOESM3_ESM.zip › Supplementary data_1/Supplementary_material_1-1 Geometric morphometrics/CVA_306/mean_shapes_per_clade_CVA/Stem_Ruminantia-oc.png]

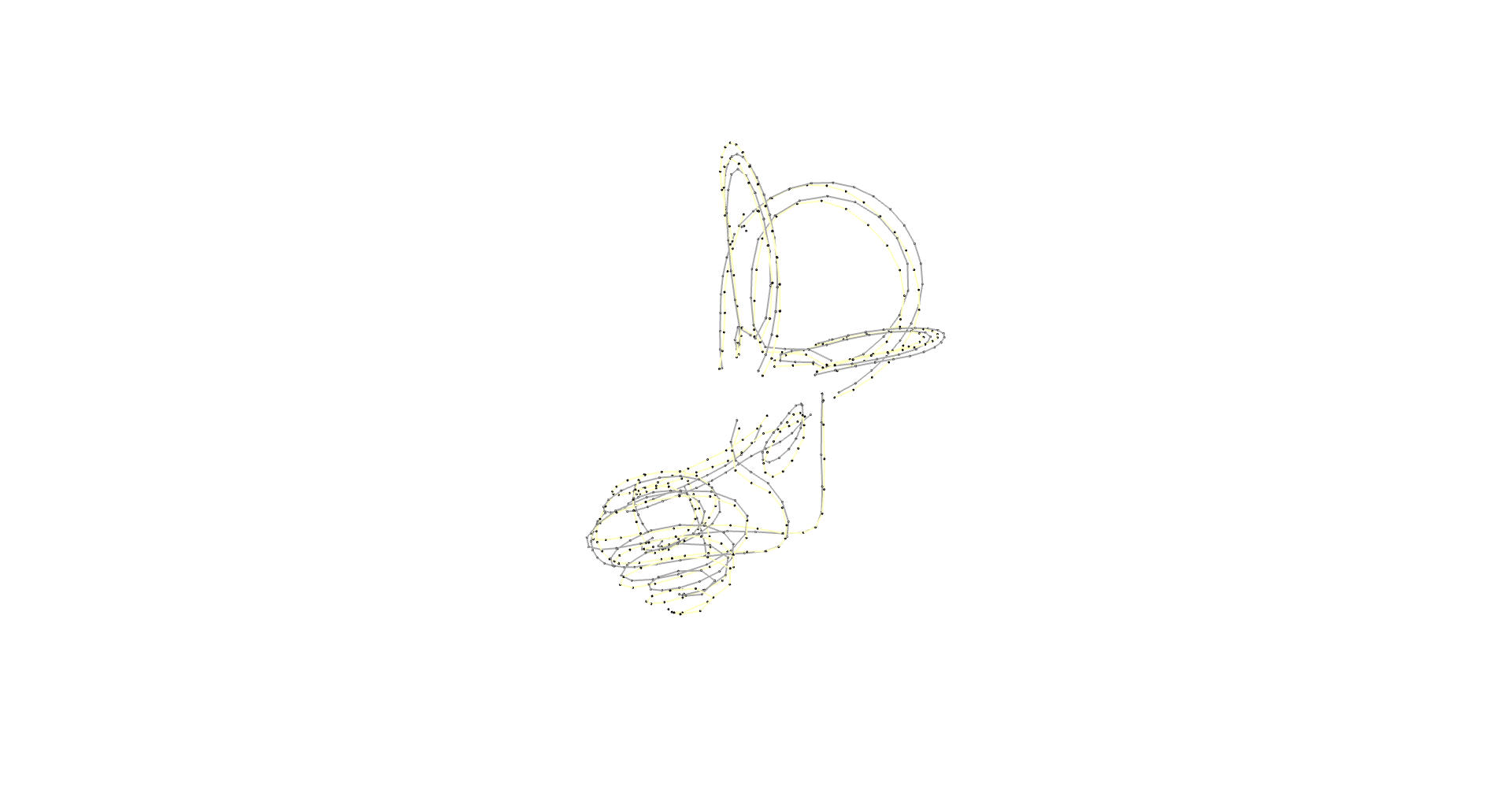

Supplement: Supplementary file 3 — Supplementary Data 1 [file 41467_2022_34656_MOESM3_ESM.zip › Supplementary data_1/Supplementary_material_1-1 Geometric morphometrics/CVA_306/mean_shapes_per_clade_CVA/Stem_Ruminantia-ro.png]

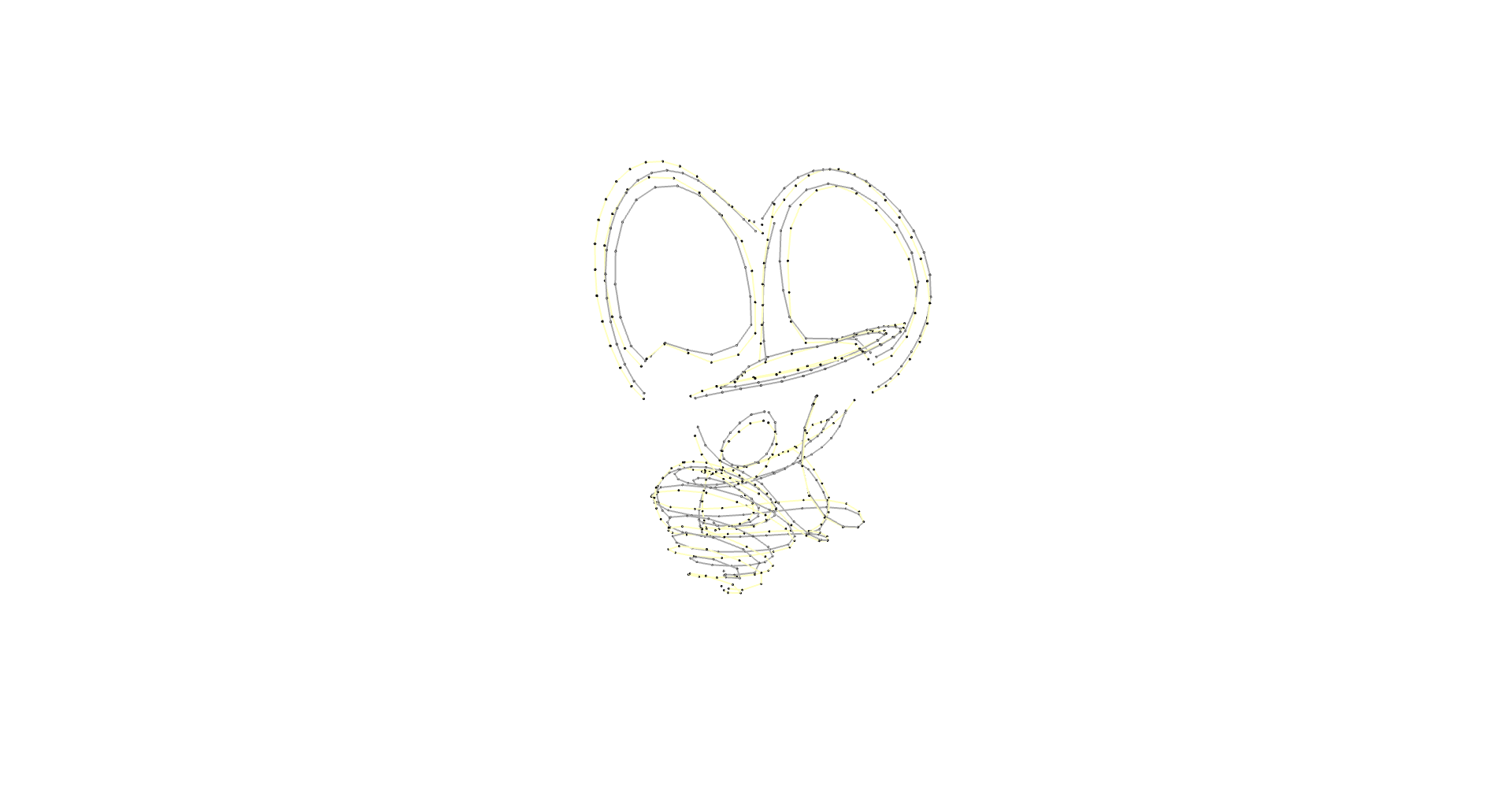

Supplement: Supplementary file 3 — Supplementary Data 1 [file 41467_2022_34656_MOESM3_ESM.zip › Supplementary data_1/Supplementary_material_1-1 Geometric morphometrics/CVA_306/mean_shapes_per_clade_CVA/Stem_Ruminantia-vl.png]

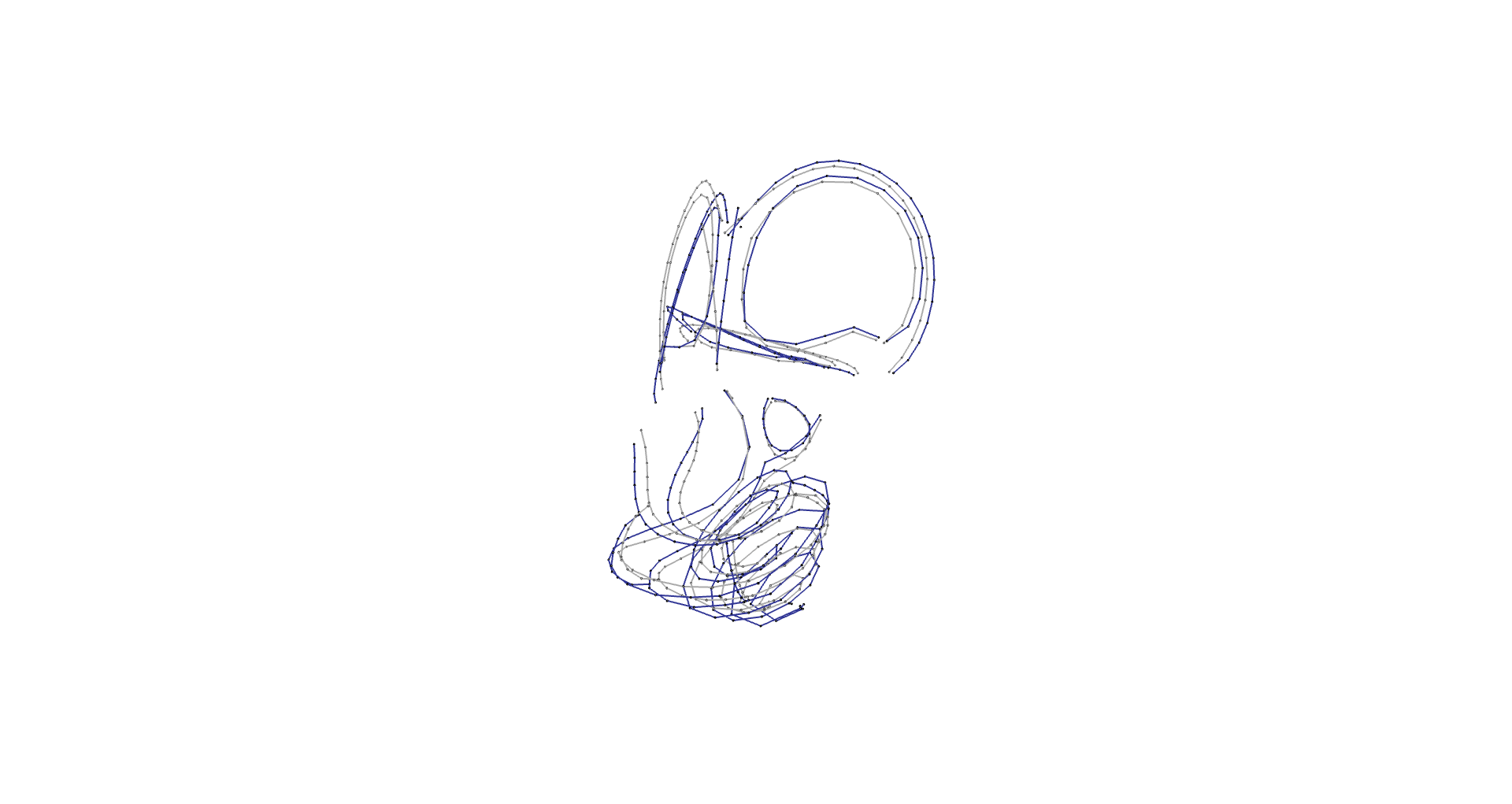

Supplement: Supplementary file 3 — Supplementary Data 1 [file 41467_2022_34656_MOESM3_ESM.zip › Supplementary data_1/Supplementary_material_1-1 Geometric morphometrics/CVA_306/mean_shapes_per_clade_CVA/Tragulidae-dl.png]

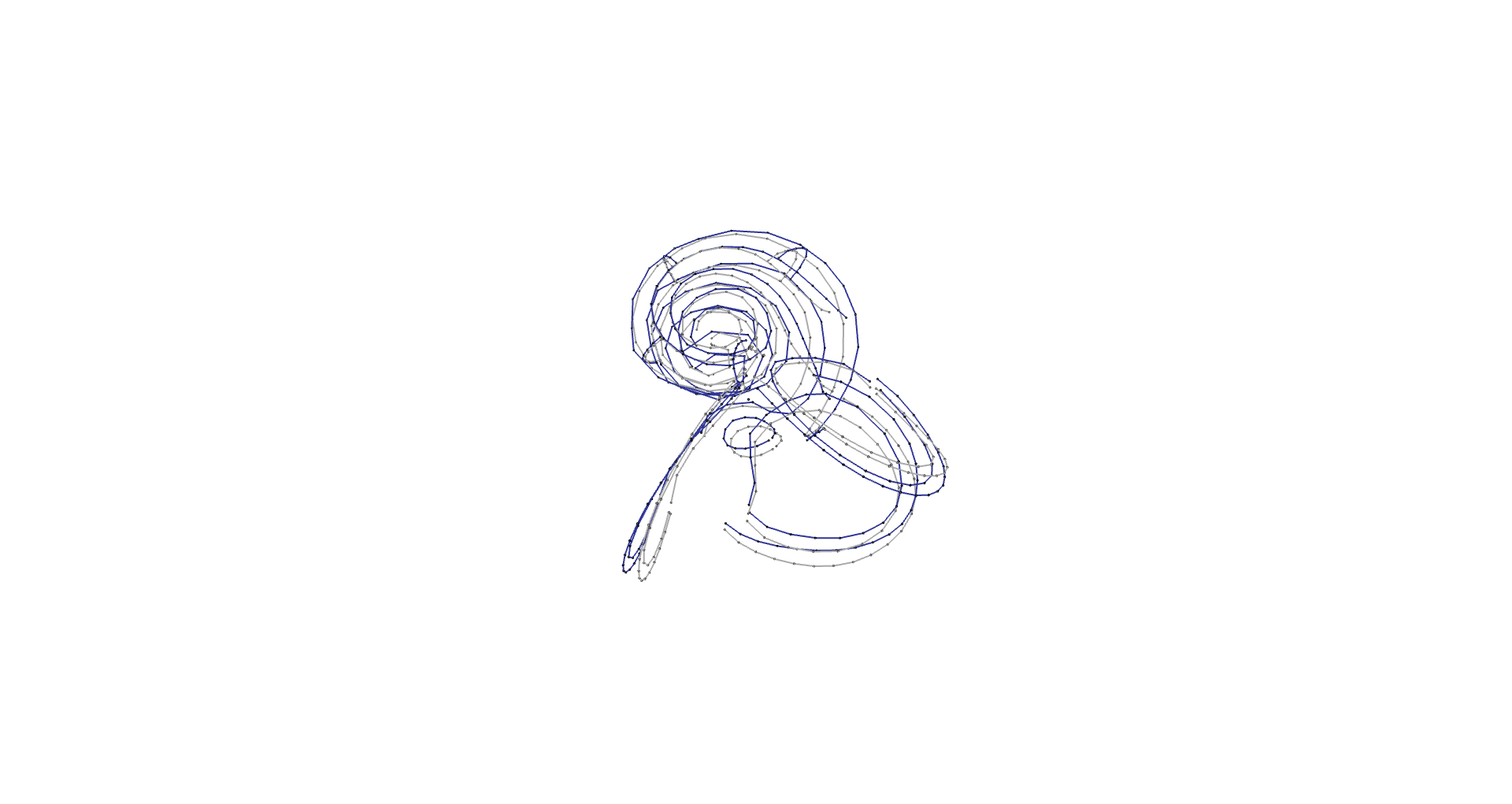

Supplement: Supplementary file 3 — Supplementary Data 1 [file 41467_2022_34656_MOESM3_ESM.zip › Supplementary data_1/Supplementary_material_1-1 Geometric morphometrics/CVA_306/mean_shapes_per_clade_CVA/Tragulidae-do.png]

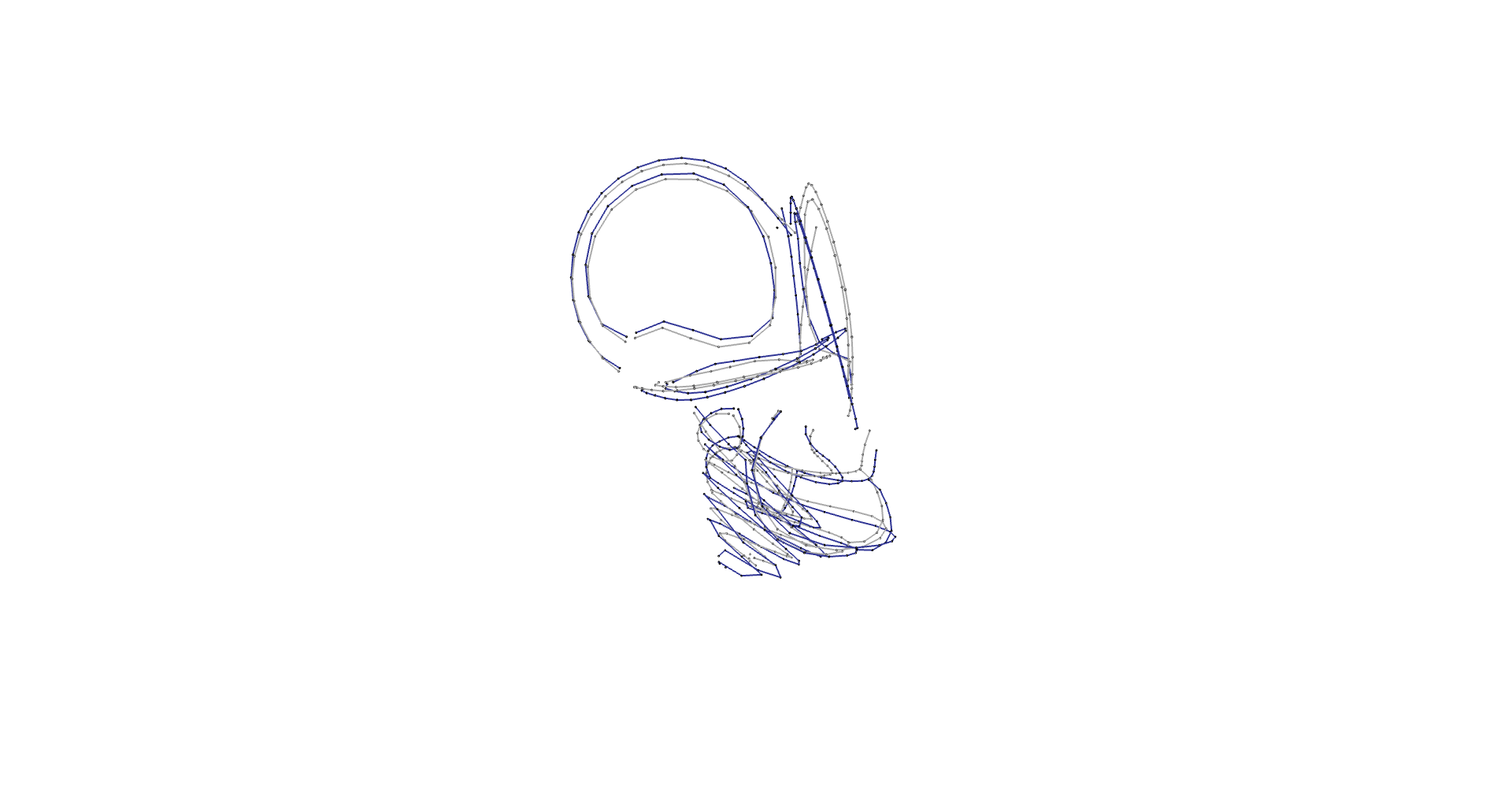

Supplement: Supplementary file 3 — Supplementary Data 1 [file 41467_2022_34656_MOESM3_ESM.zip › Supplementary data_1/Supplementary_material_1-1 Geometric morphometrics/CVA_306/mean_shapes_per_clade_CVA/Tragulidae-la.png]

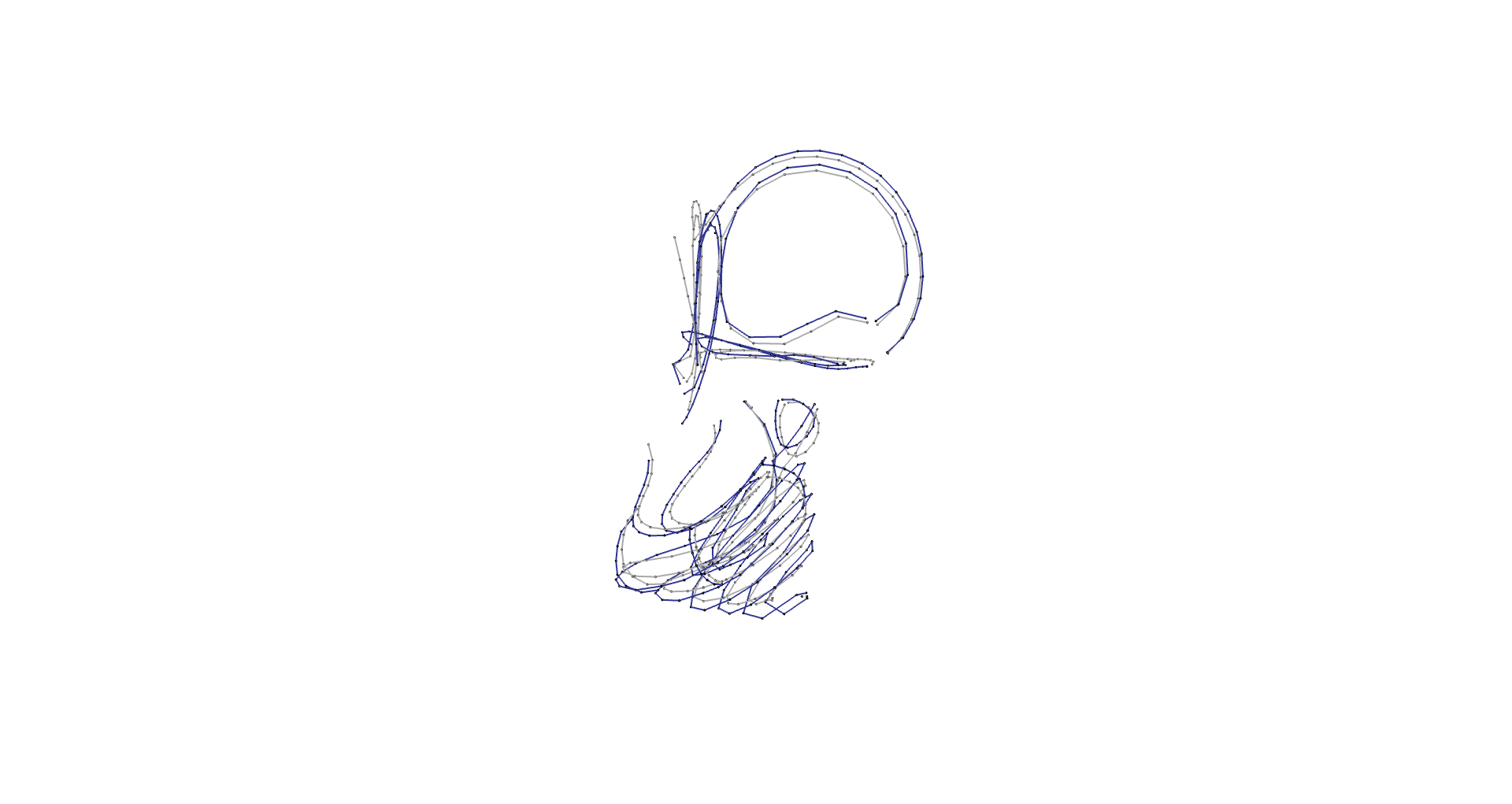

Supplement: Supplementary file 3 — Supplementary Data 1 [file 41467_2022_34656_MOESM3_ESM.zip › Supplementary data_1/Supplementary_material_1-1 Geometric morphometrics/CVA_306/mean_shapes_per_clade_CVA/Tragulidae-me.png]

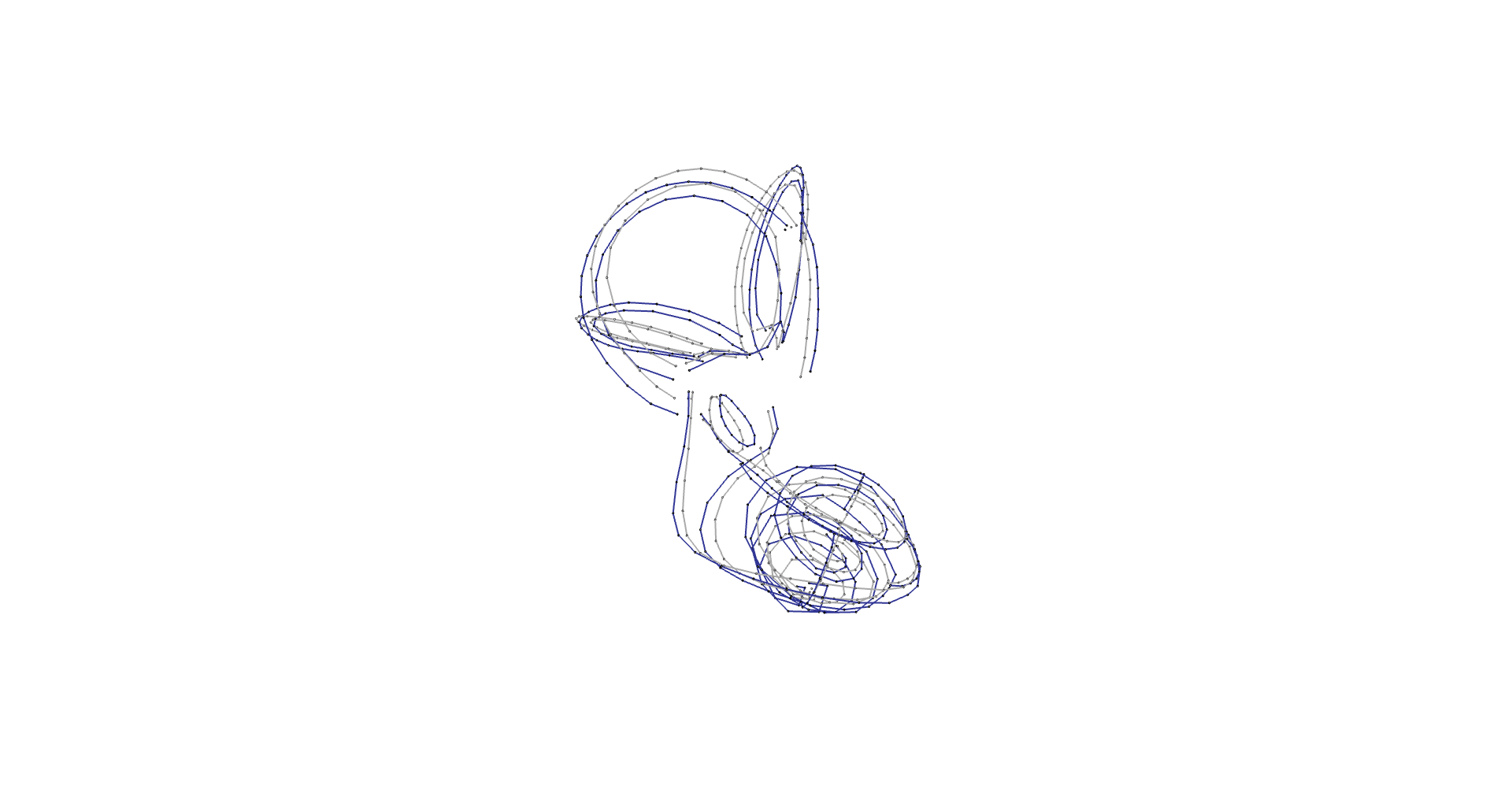

Supplement: Supplementary file 3 — Supplementary Data 1 [file 41467_2022_34656_MOESM3_ESM.zip › Supplementary data_1/Supplementary_material_1-1 Geometric morphometrics/CVA_306/mean_shapes_per_clade_CVA/Tragulidae-oc.png]

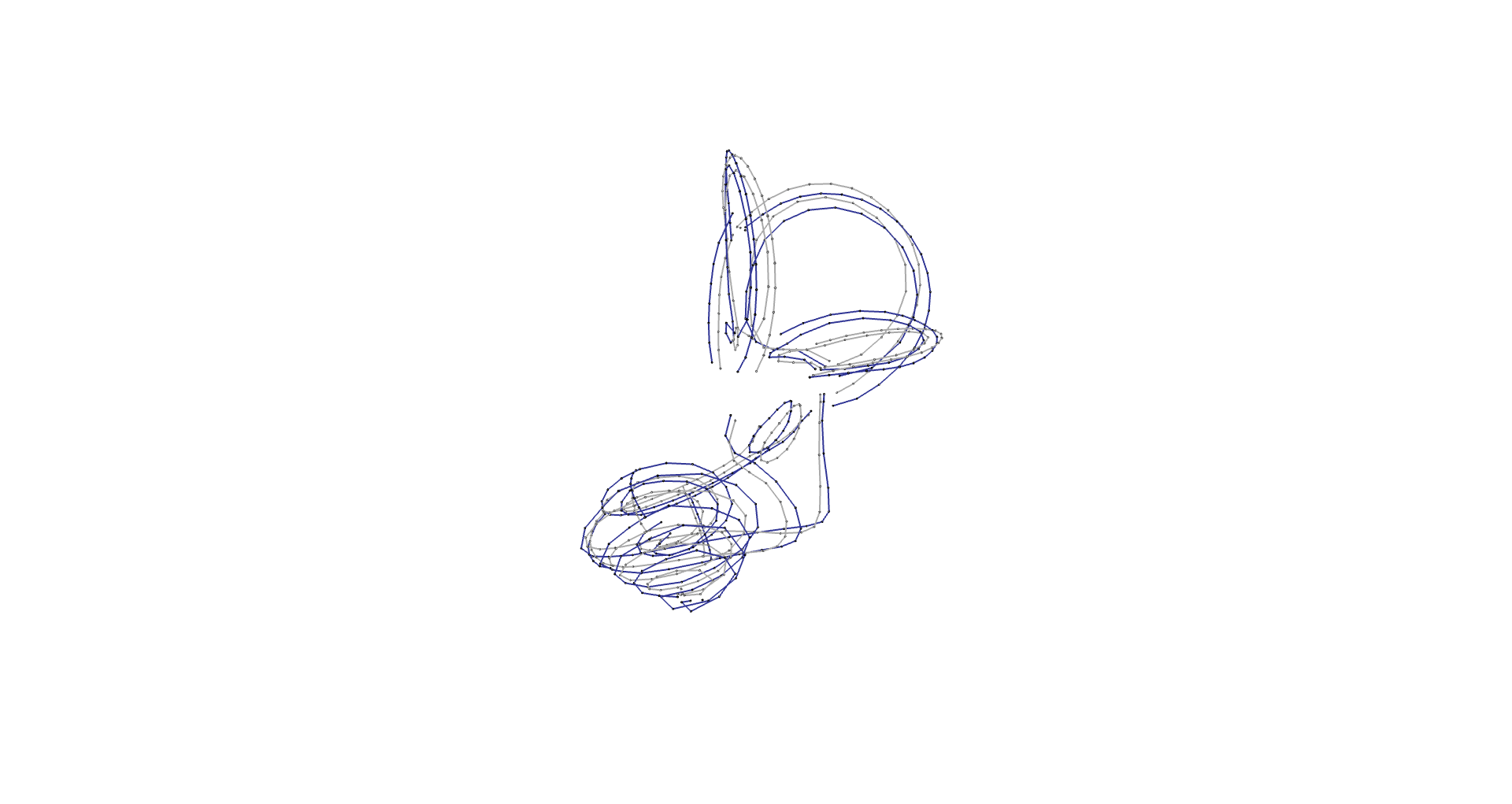

Supplement: Supplementary file 3 — Supplementary Data 1 [file 41467_2022_34656_MOESM3_ESM.zip › Supplementary data_1/Supplementary_material_1-1 Geometric morphometrics/CVA_306/mean_shapes_per_clade_CVA/Tragulidae-ro.png]

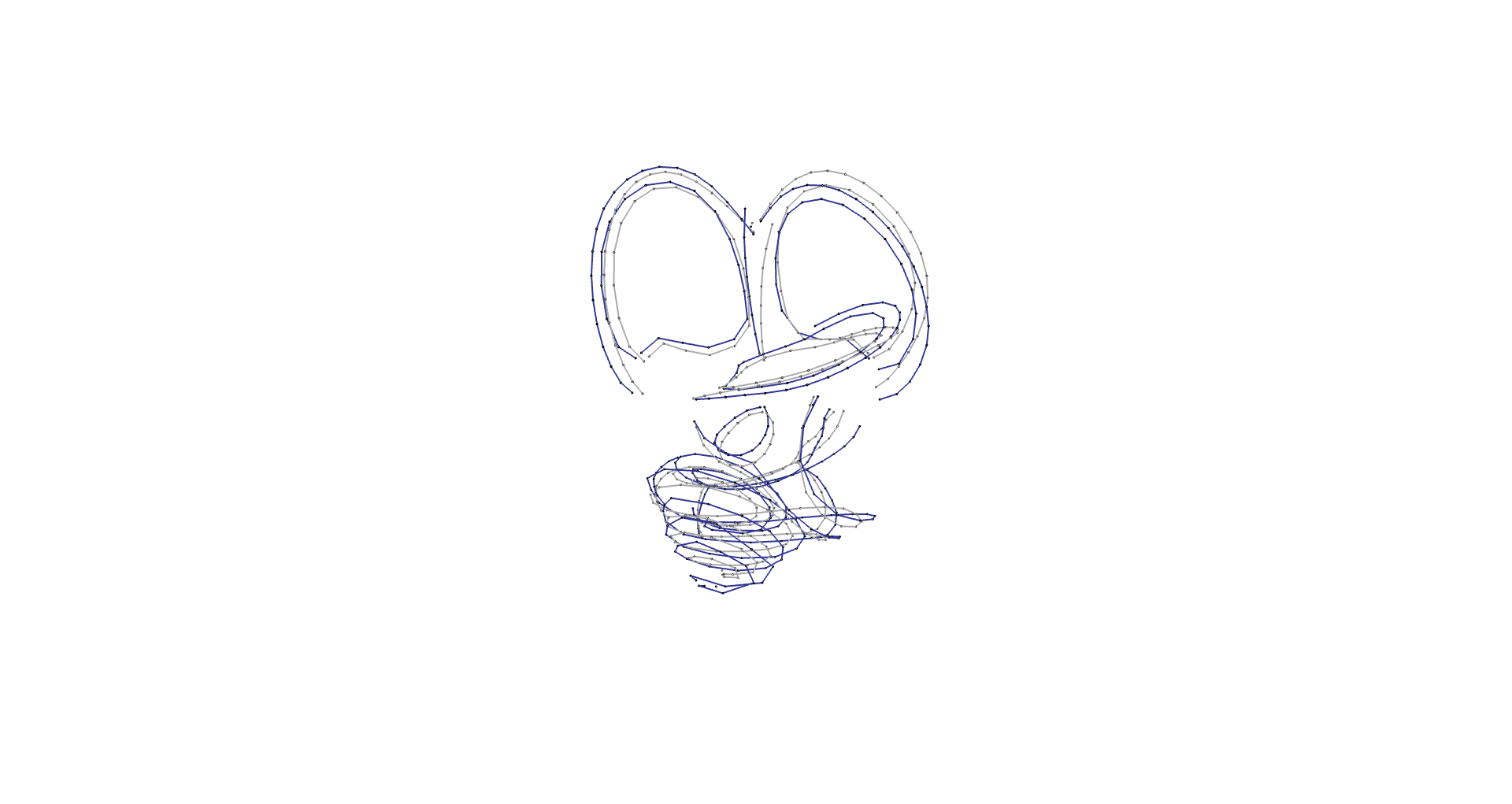

Supplement: Supplementary file 3 — Supplementary Data 1 [file 41467_2022_34656_MOESM3_ESM.zip › Supplementary data_1/Supplementary_material_1-1 Geometric morphometrics/CVA_306/mean_shapes_per_clade_CVA/Tragulidae-vl.png]

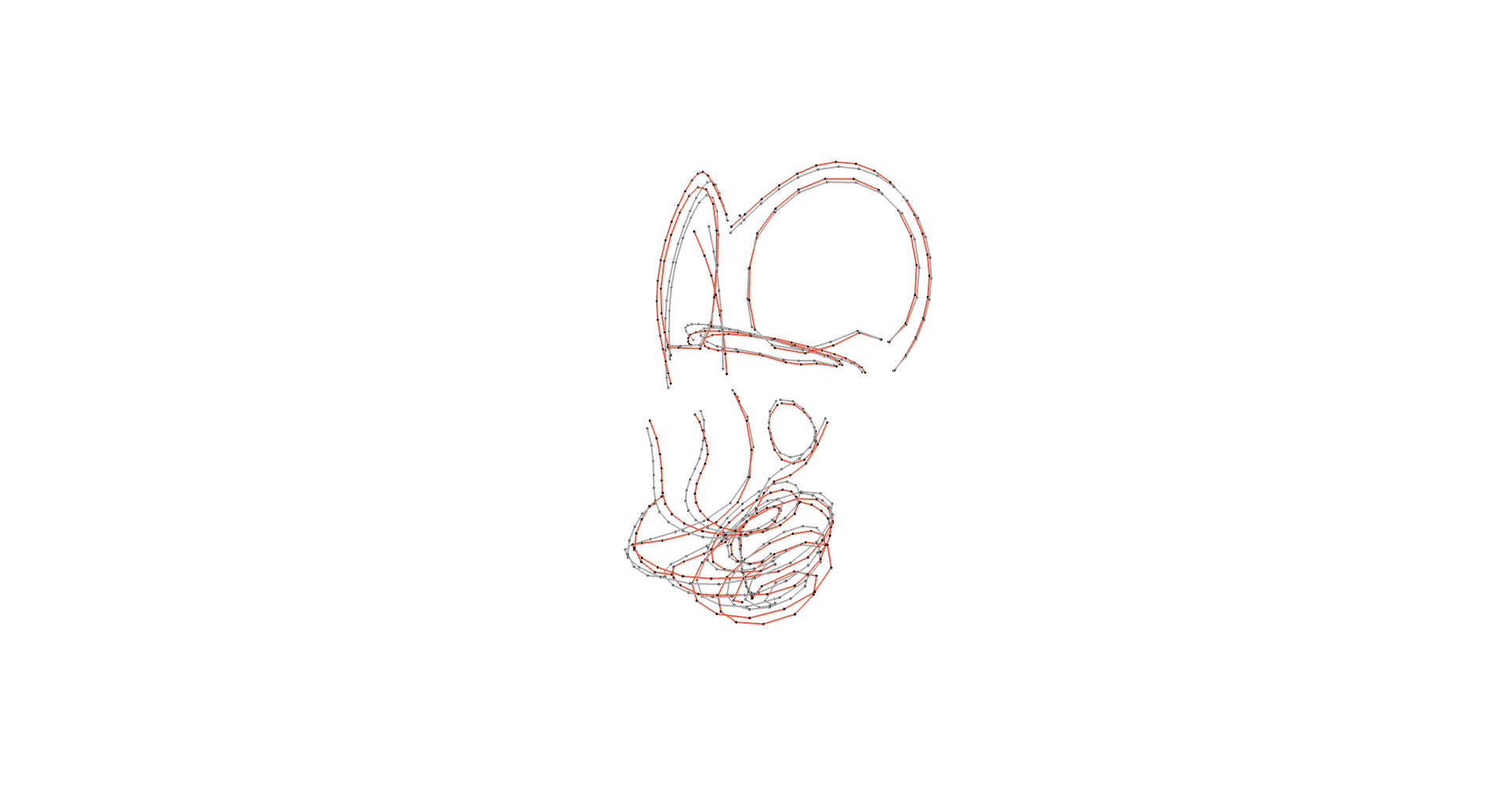

Supplement: Supplementary file 3 — Supplementary Data 1 [file 41467_2022_34656_MOESM3_ESM.zip › Supplementary data_1/Supplementary_material_1-1 Geometric morphometrics/PCA_306/Extreme_shapes_PCA/PC1max-dl.png]

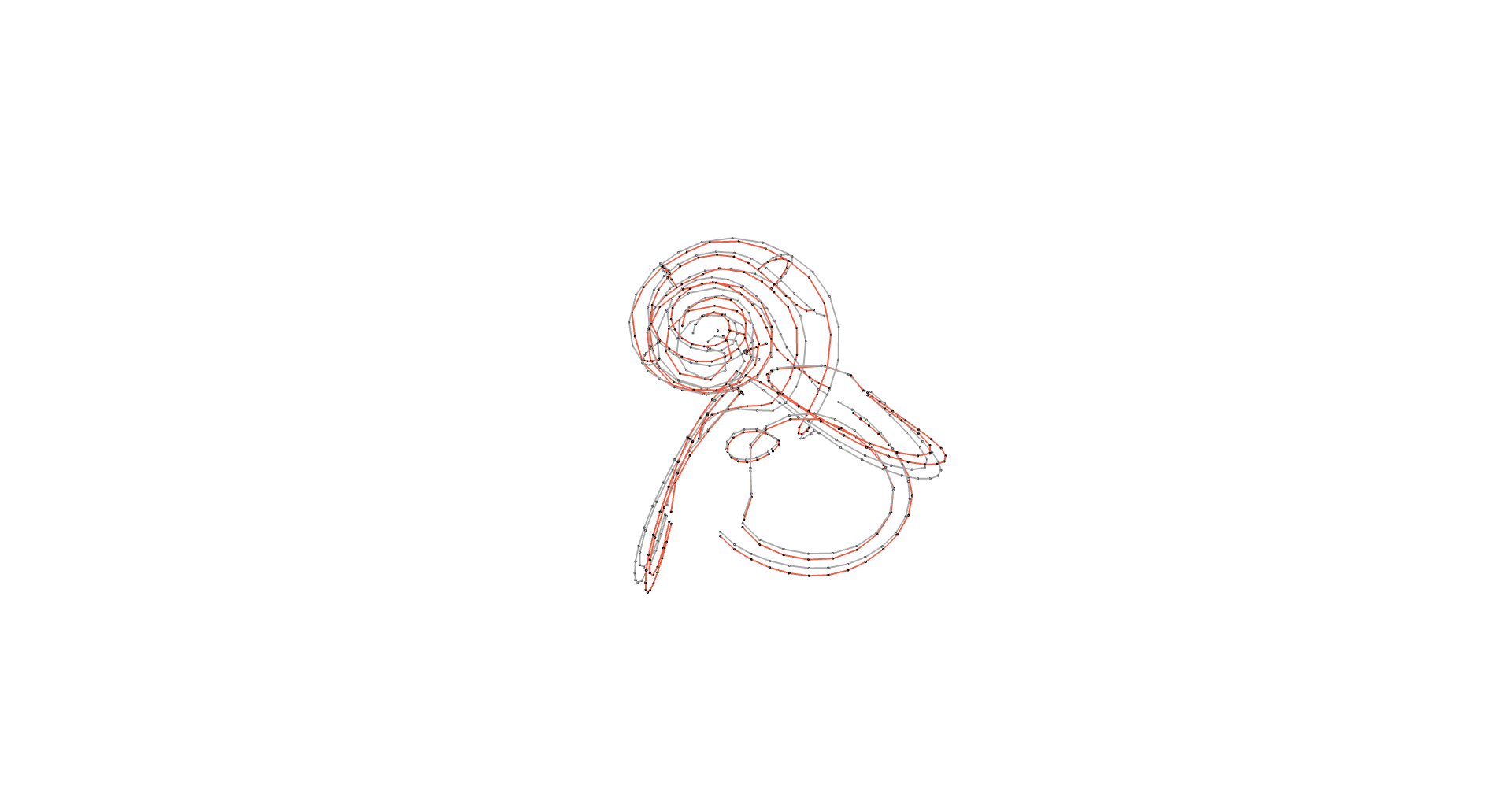

Supplement: Supplementary file 3 — Supplementary Data 1 [file 41467_2022_34656_MOESM3_ESM.zip › Supplementary data_1/Supplementary_material_1-1 Geometric morphometrics/PCA_306/Extreme_shapes_PCA/PC1max-do.png]

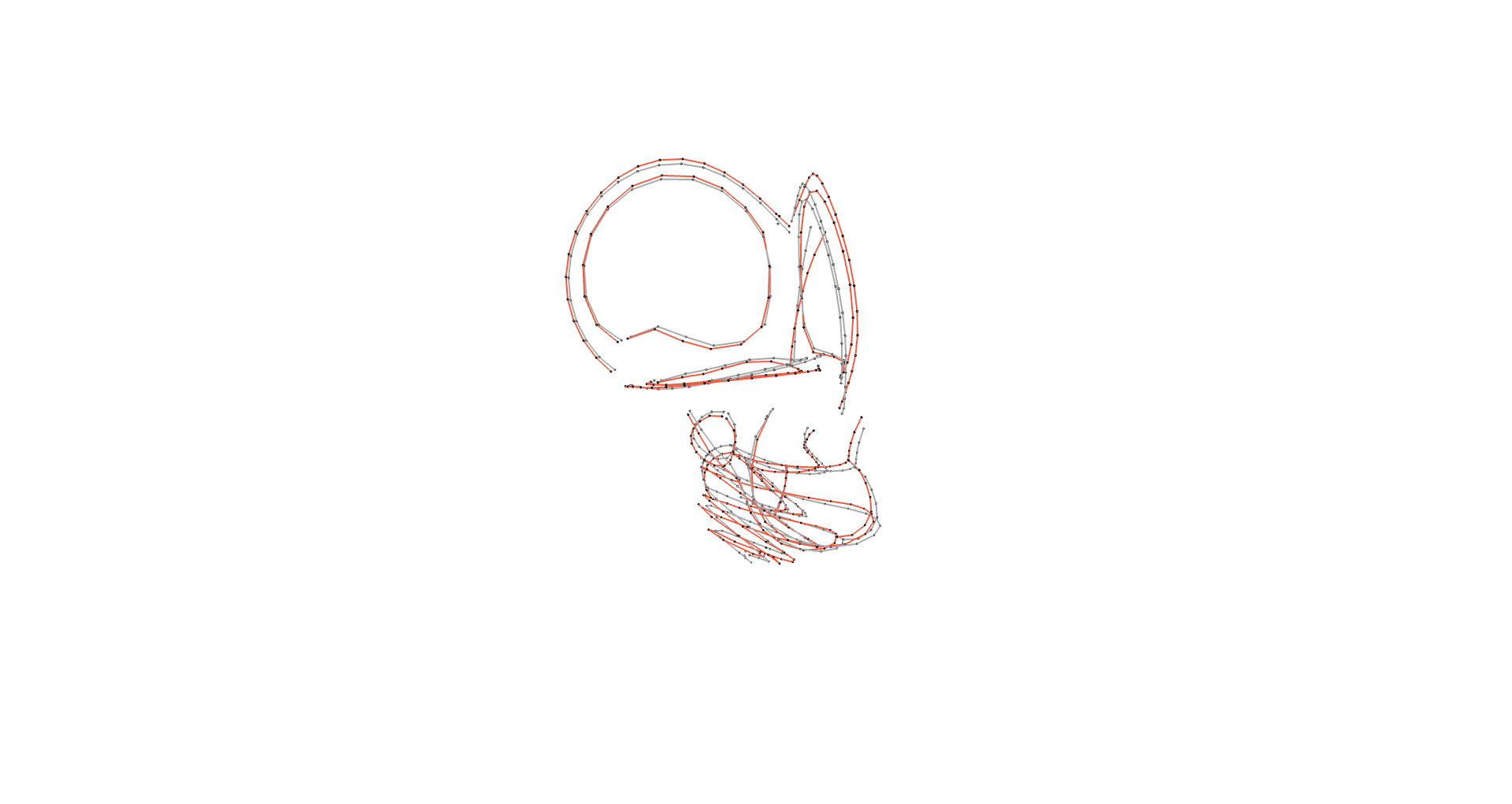

Supplement: Supplementary file 3 — Supplementary Data 1 [file 41467_2022_34656_MOESM3_ESM.zip › Supplementary data_1/Supplementary_material_1-1 Geometric morphometrics/PCA_306/Extreme_shapes_PCA/PC1max-la.png]

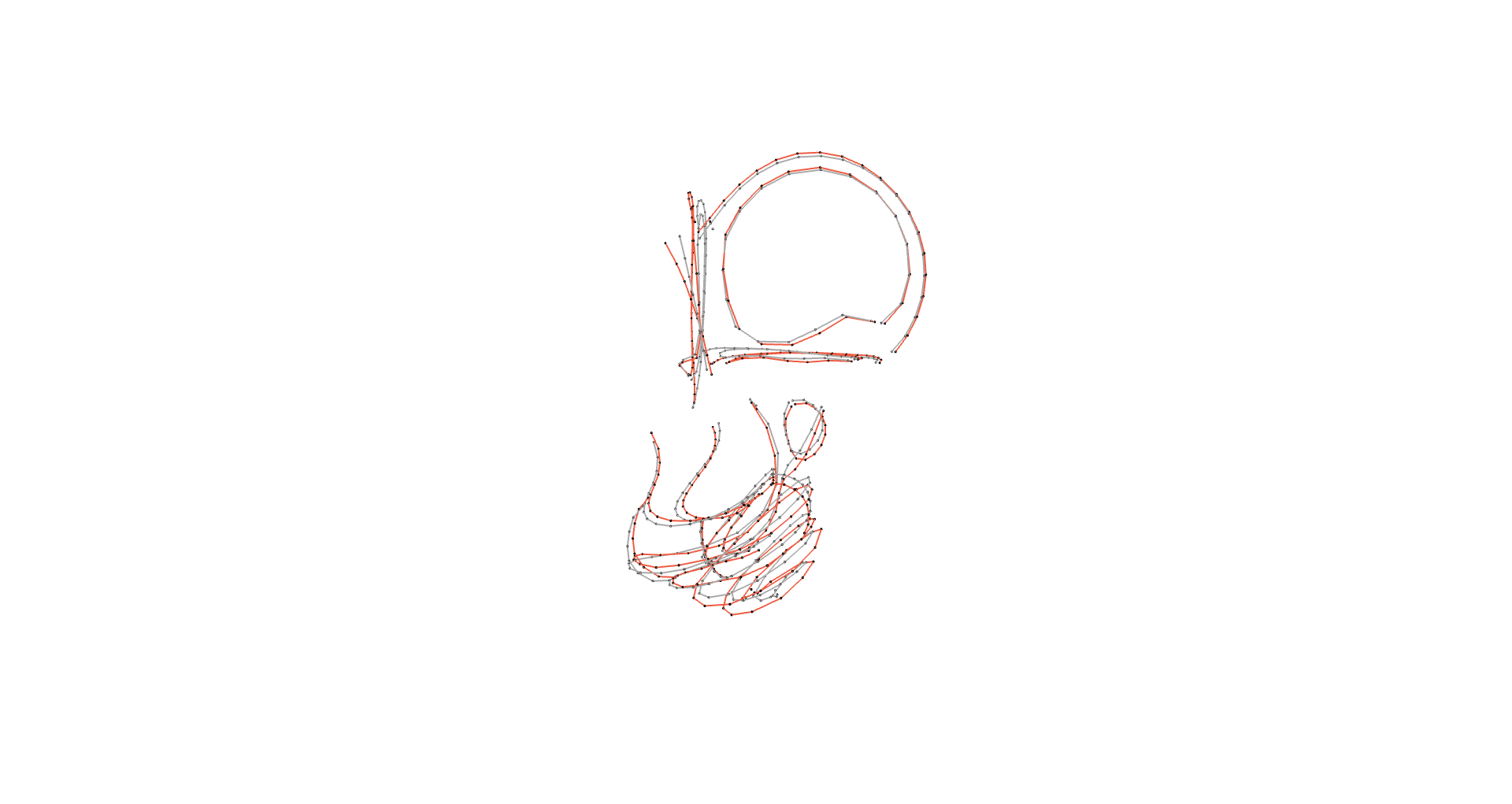

Supplement: Supplementary file 3 — Supplementary Data 1 [file 41467_2022_34656_MOESM3_ESM.zip › Supplementary data_1/Supplementary_material_1-1 Geometric morphometrics/PCA_306/Extreme_shapes_PCA/PC1max-me.png]

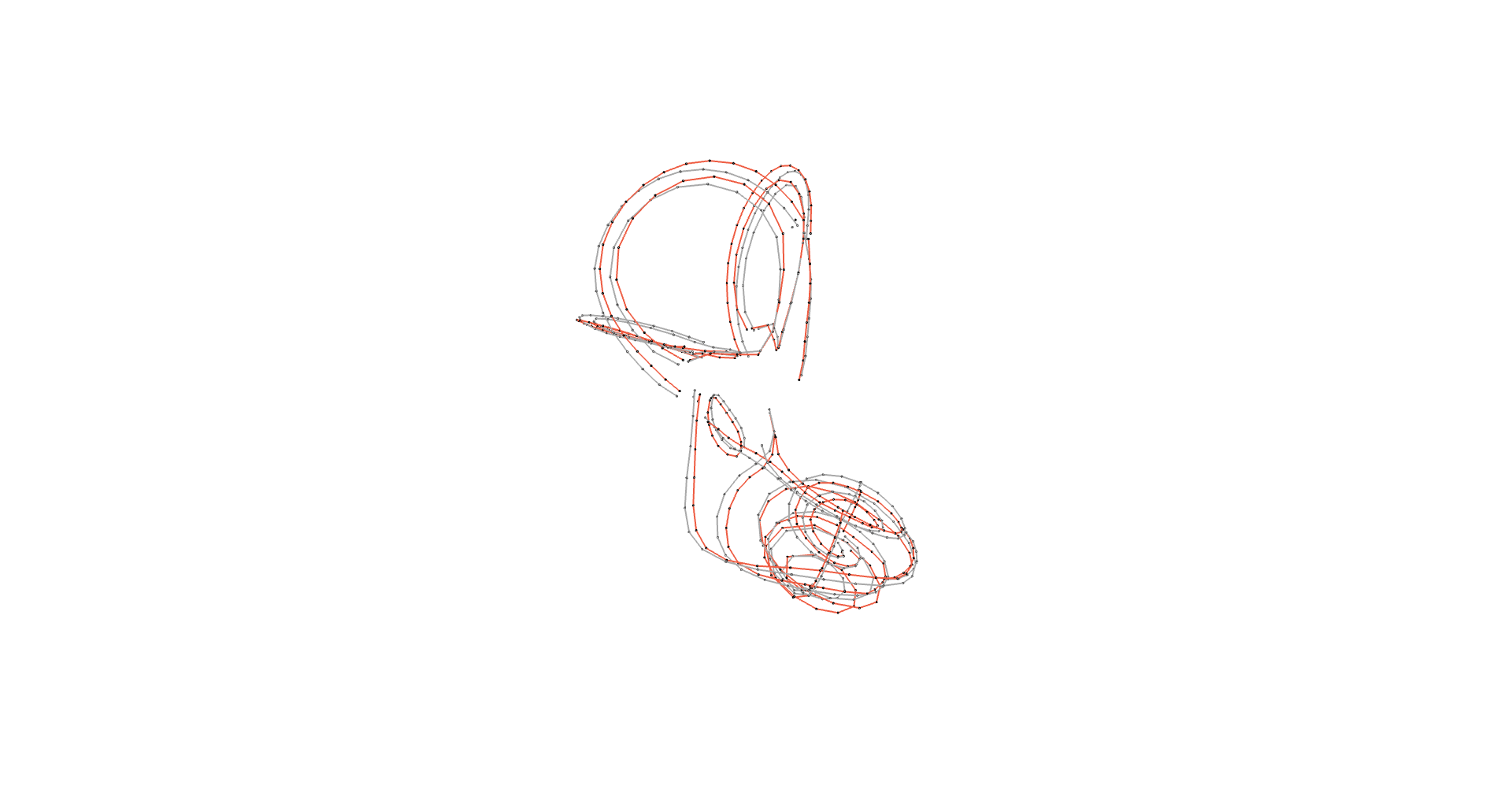

Supplement: Supplementary file 3 — Supplementary Data 1 [file 41467_2022_34656_MOESM3_ESM.zip › Supplementary data_1/Supplementary_material_1-1 Geometric morphometrics/PCA_306/Extreme_shapes_PCA/PC1max-oc.png]

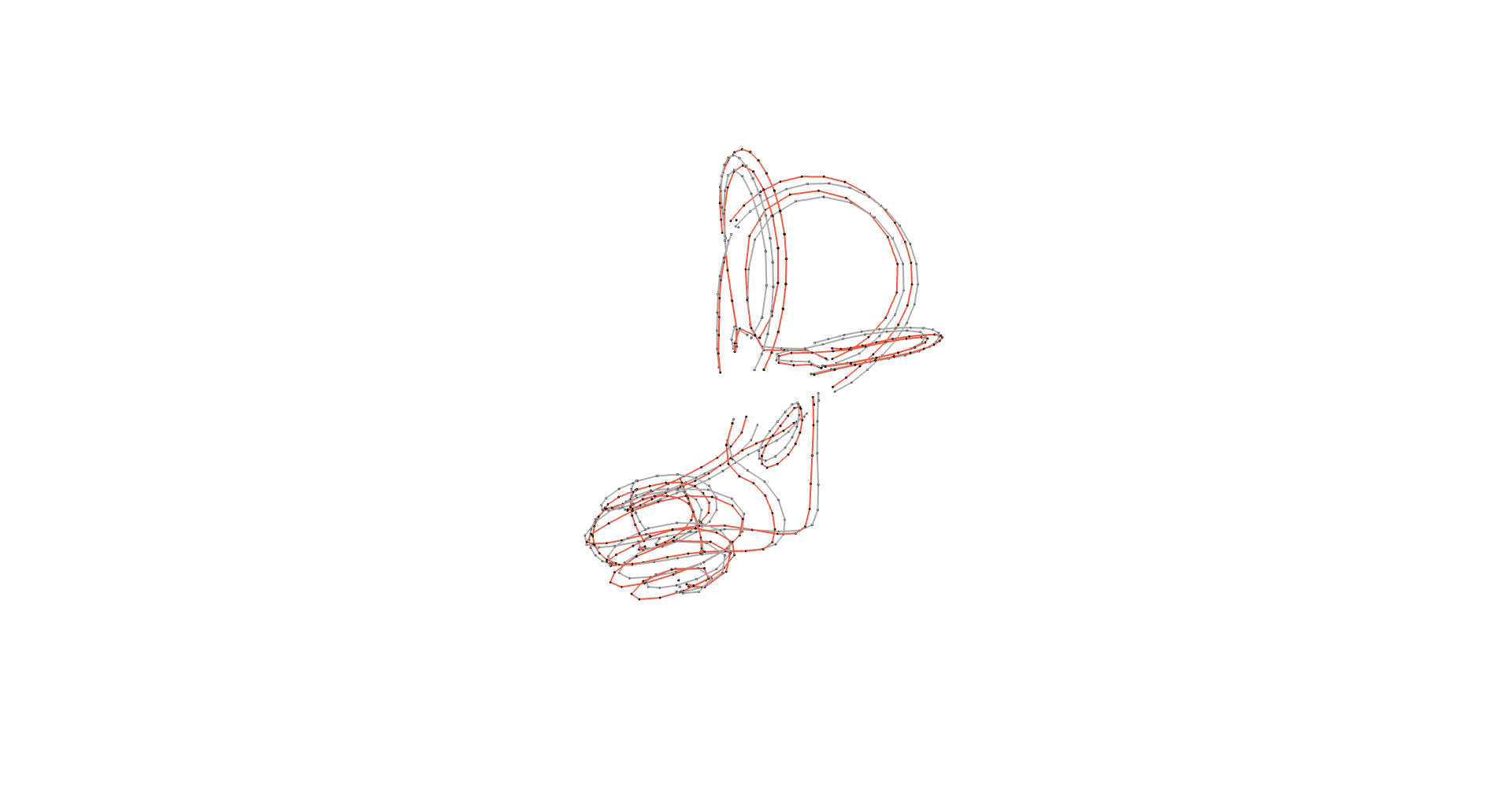

Supplement: Supplementary file 3 — Supplementary Data 1 [file 41467_2022_34656_MOESM3_ESM.zip › Supplementary data_1/Supplementary_material_1-1 Geometric morphometrics/PCA_306/Extreme_shapes_PCA/PC1max-ro.png]

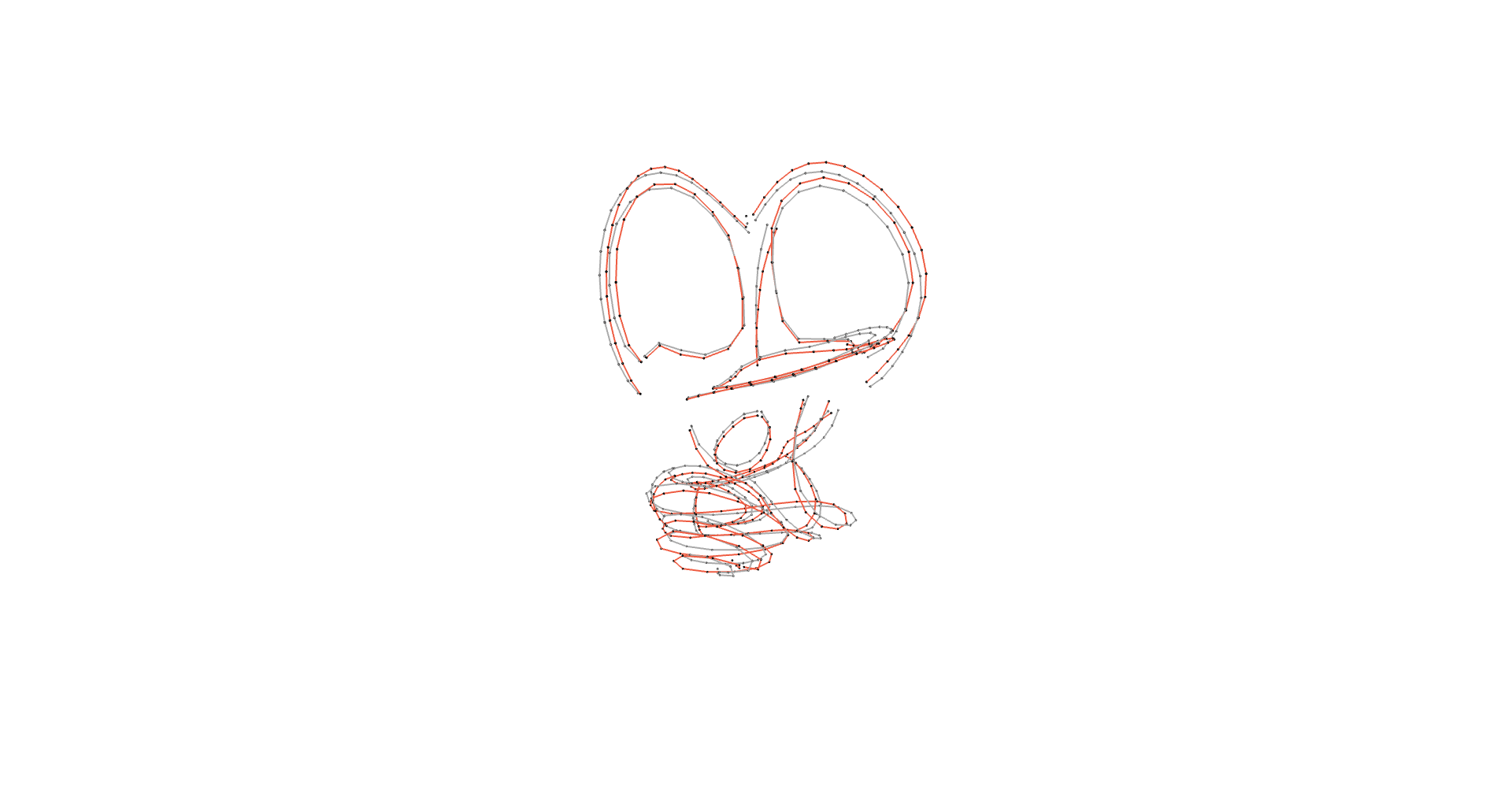

Supplement: Supplementary file 3 — Supplementary Data 1 [file 41467_2022_34656_MOESM3_ESM.zip › Supplementary data_1/Supplementary_material_1-1 Geometric morphometrics/PCA_306/Extreme_shapes_PCA/PC1max-vl.png]

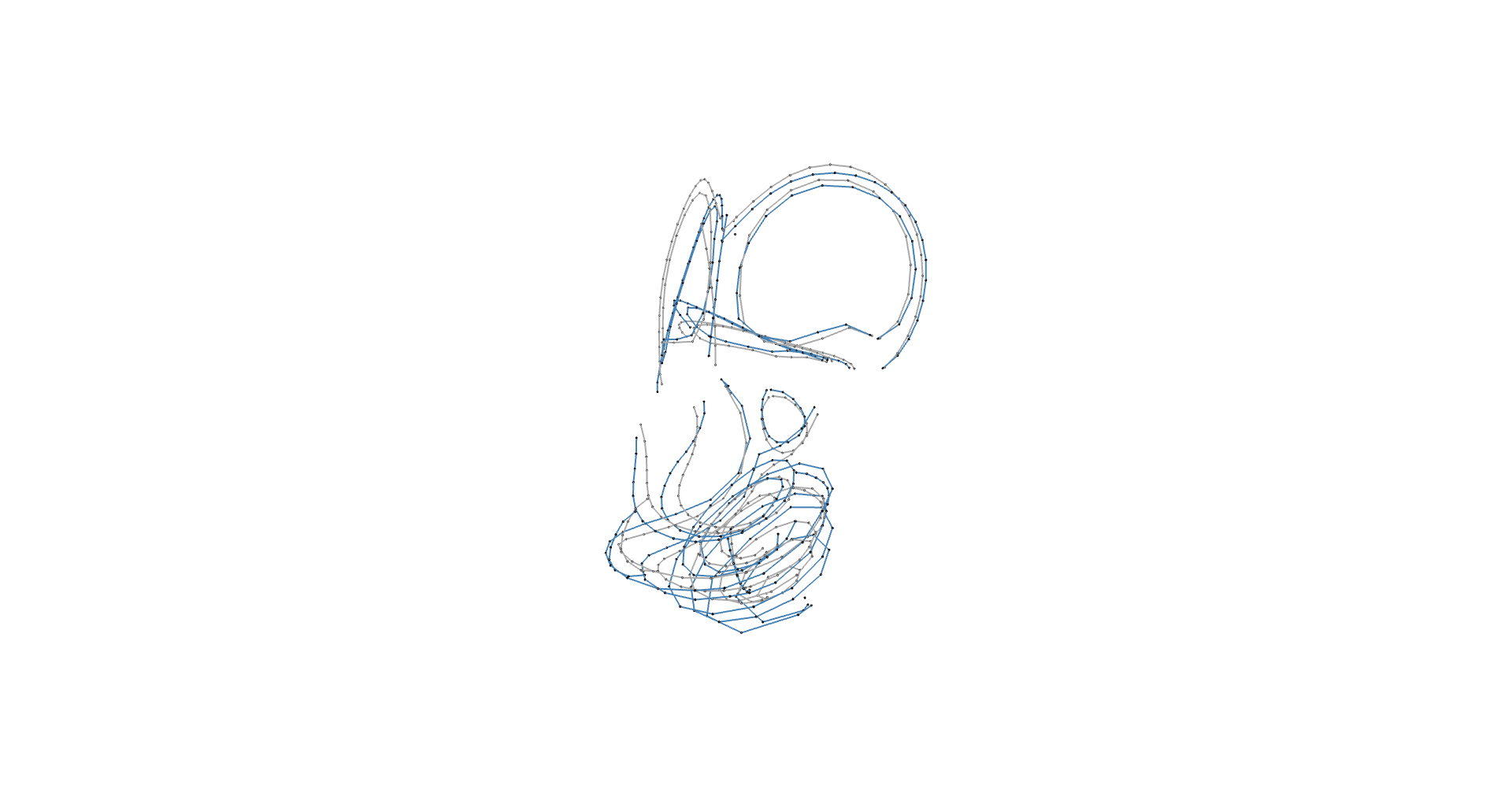

Supplement: Supplementary file 3 — Supplementary Data 1 [file 41467_2022_34656_MOESM3_ESM.zip › Supplementary data_1/Supplementary_material_1-1 Geometric morphometrics/PCA_306/Extreme_shapes_PCA/PC1min-dl.png]

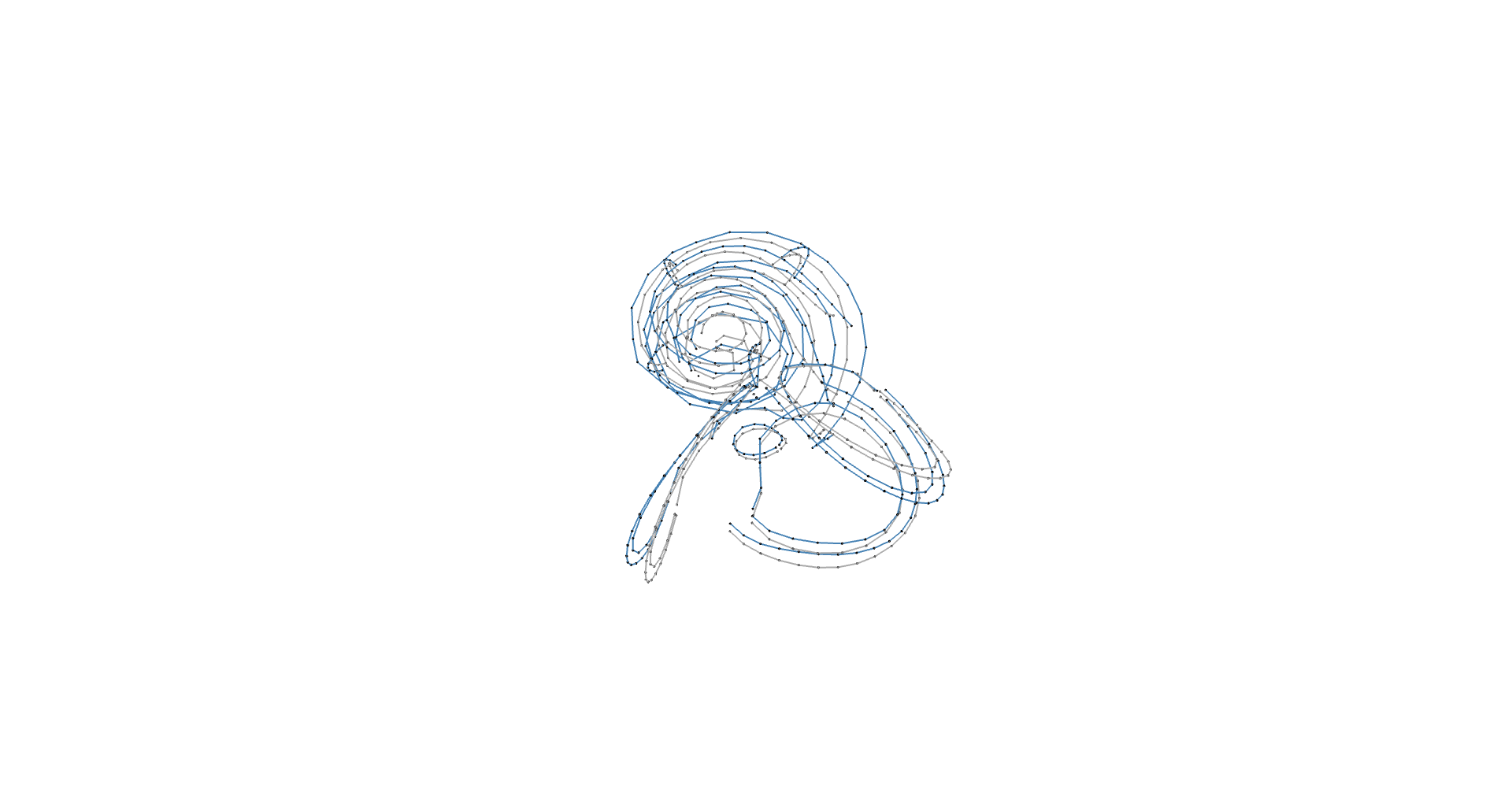

Supplement: Supplementary file 3 — Supplementary Data 1 [file 41467_2022_34656_MOESM3_ESM.zip › Supplementary data_1/Supplementary_material_1-1 Geometric morphometrics/PCA_306/Extreme_shapes_PCA/PC1min-do.png]

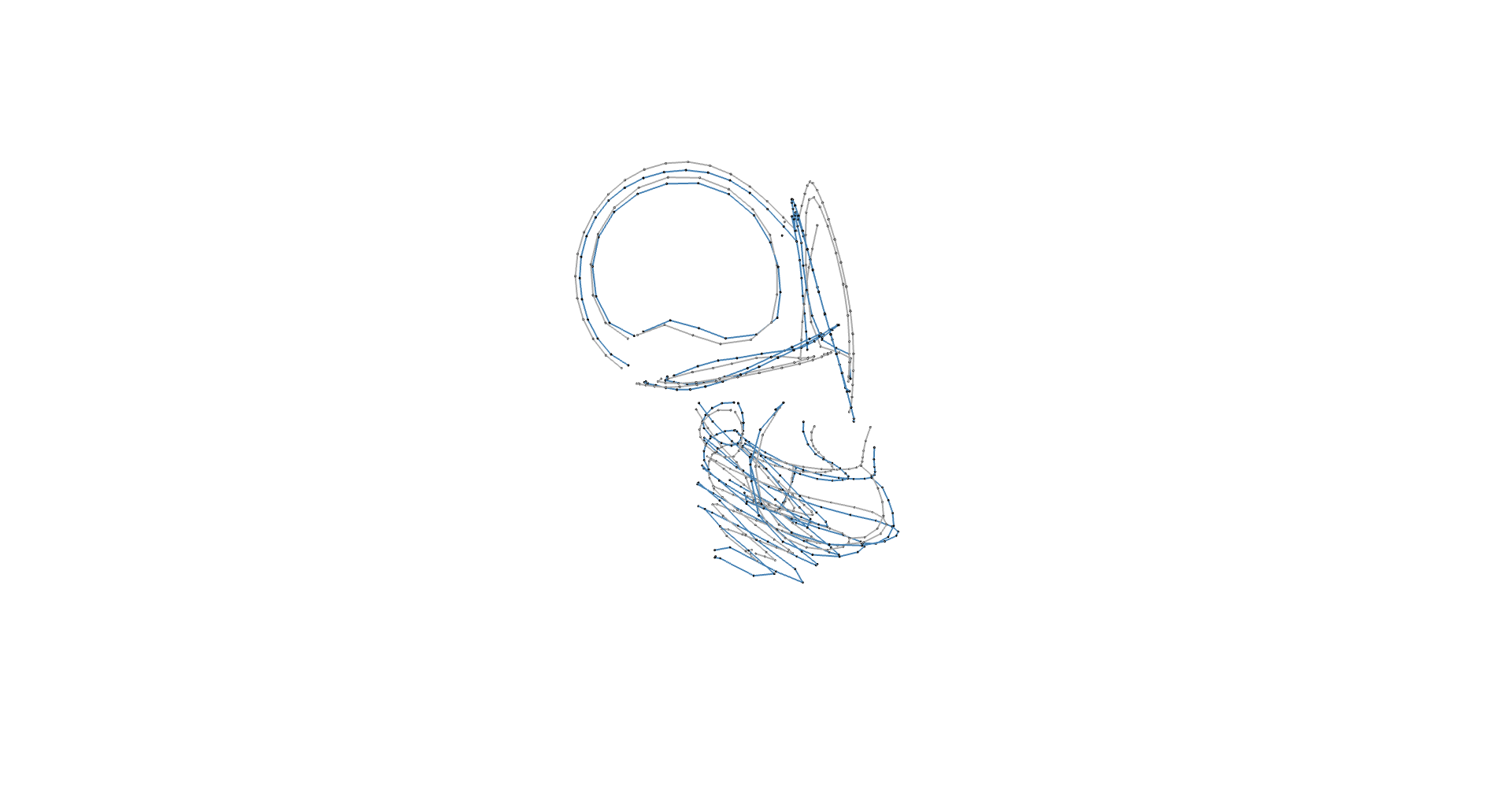

Supplement: Supplementary file 3 — Supplementary Data 1 [file 41467_2022_34656_MOESM3_ESM.zip › Supplementary data_1/Supplementary_material_1-1 Geometric morphometrics/PCA_306/Extreme_shapes_PCA/PC1min-la.png]

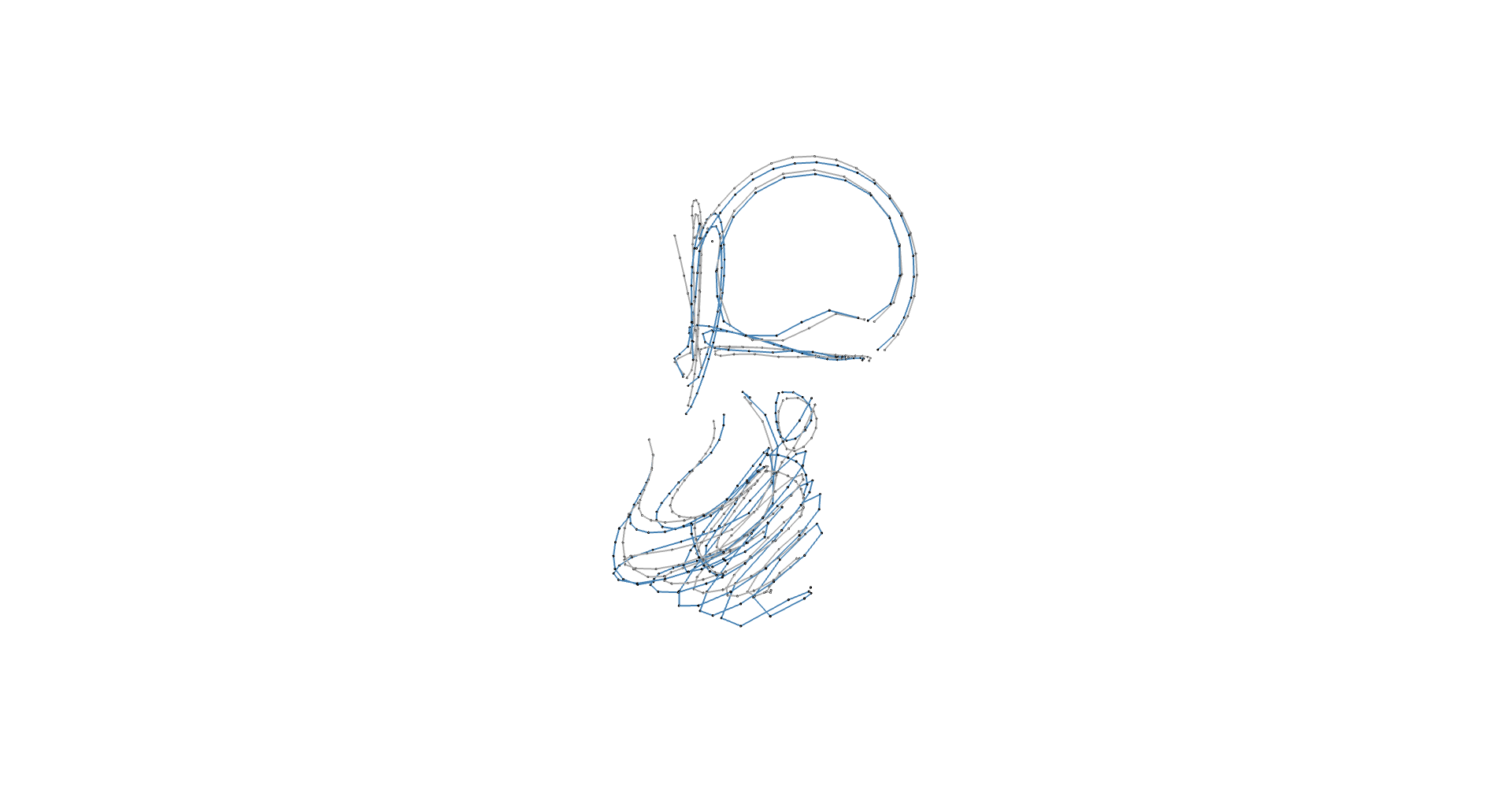

Supplement: Supplementary file 3 — Supplementary Data 1 [file 41467_2022_34656_MOESM3_ESM.zip › Supplementary data_1/Supplementary_material_1-1 Geometric morphometrics/PCA_306/Extreme_shapes_PCA/PC1min-me.png]

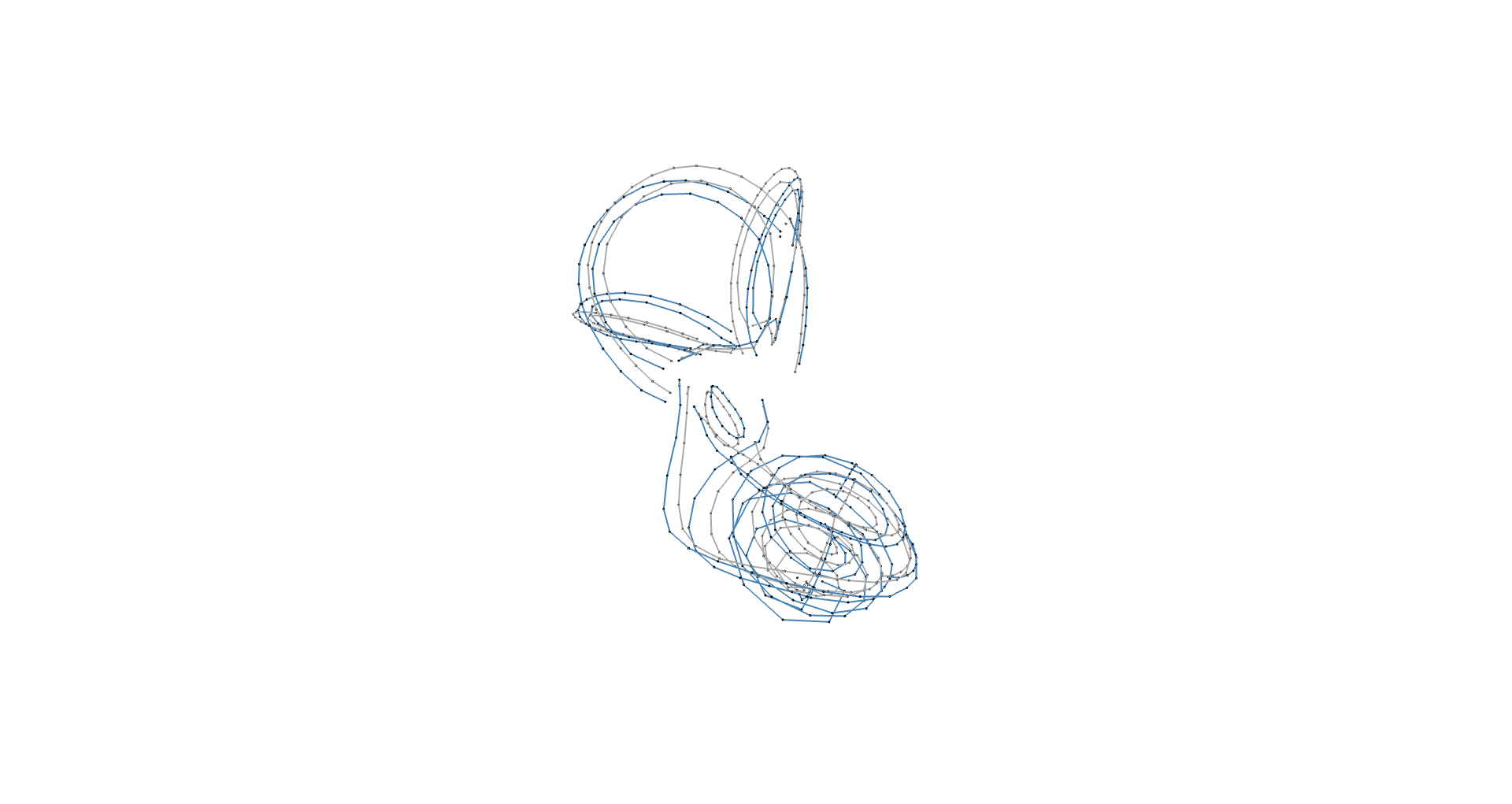

Supplement: Supplementary file 3 — Supplementary Data 1 [file 41467_2022_34656_MOESM3_ESM.zip › Supplementary data_1/Supplementary_material_1-1 Geometric morphometrics/PCA_306/Extreme_shapes_PCA/PC1min-oc.png]

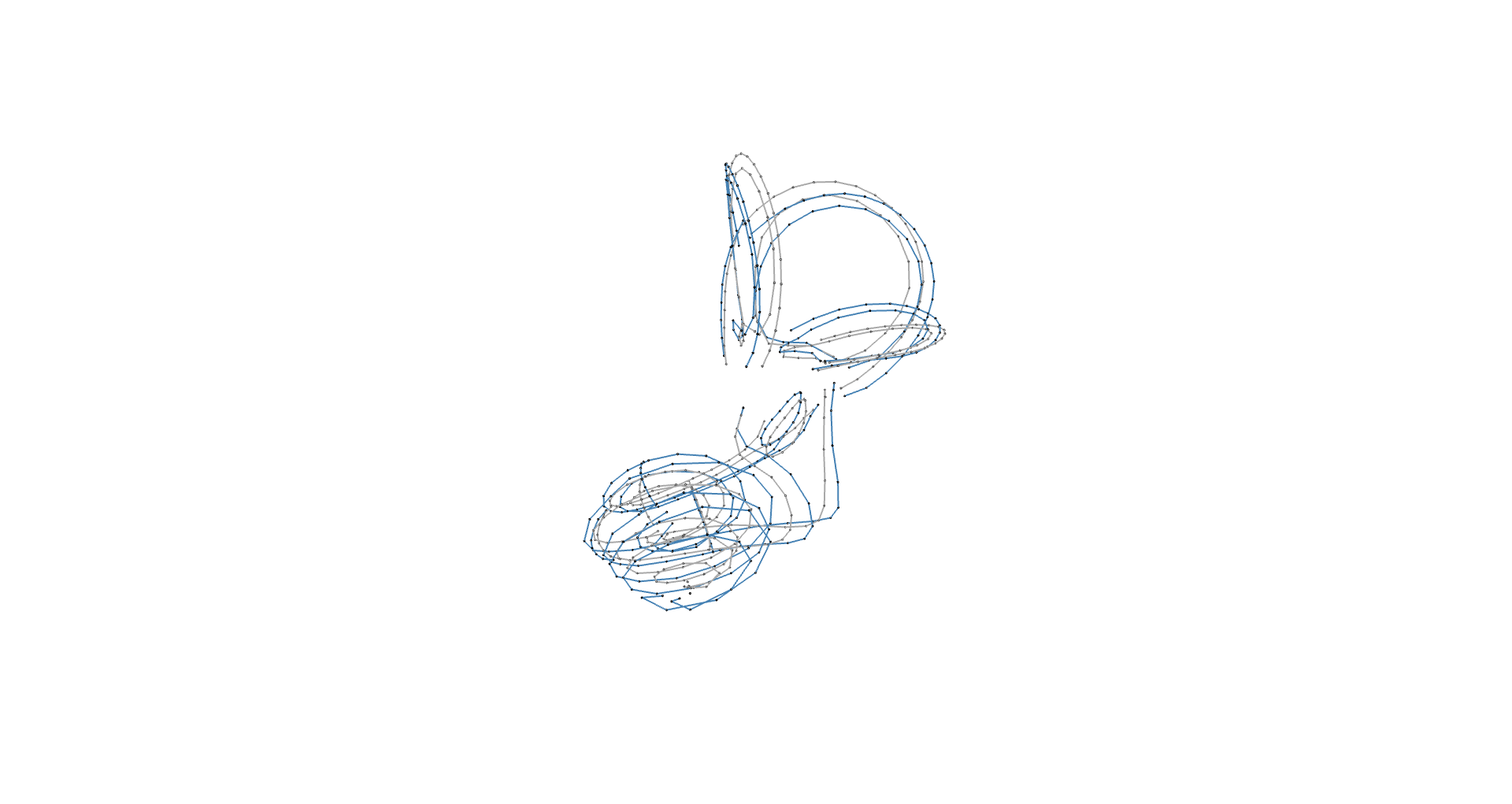

Supplement: Supplementary file 3 — Supplementary Data 1 [file 41467_2022_34656_MOESM3_ESM.zip › Supplementary data_1/Supplementary_material_1-1 Geometric morphometrics/PCA_306/Extreme_shapes_PCA/PC1min-ro.png]

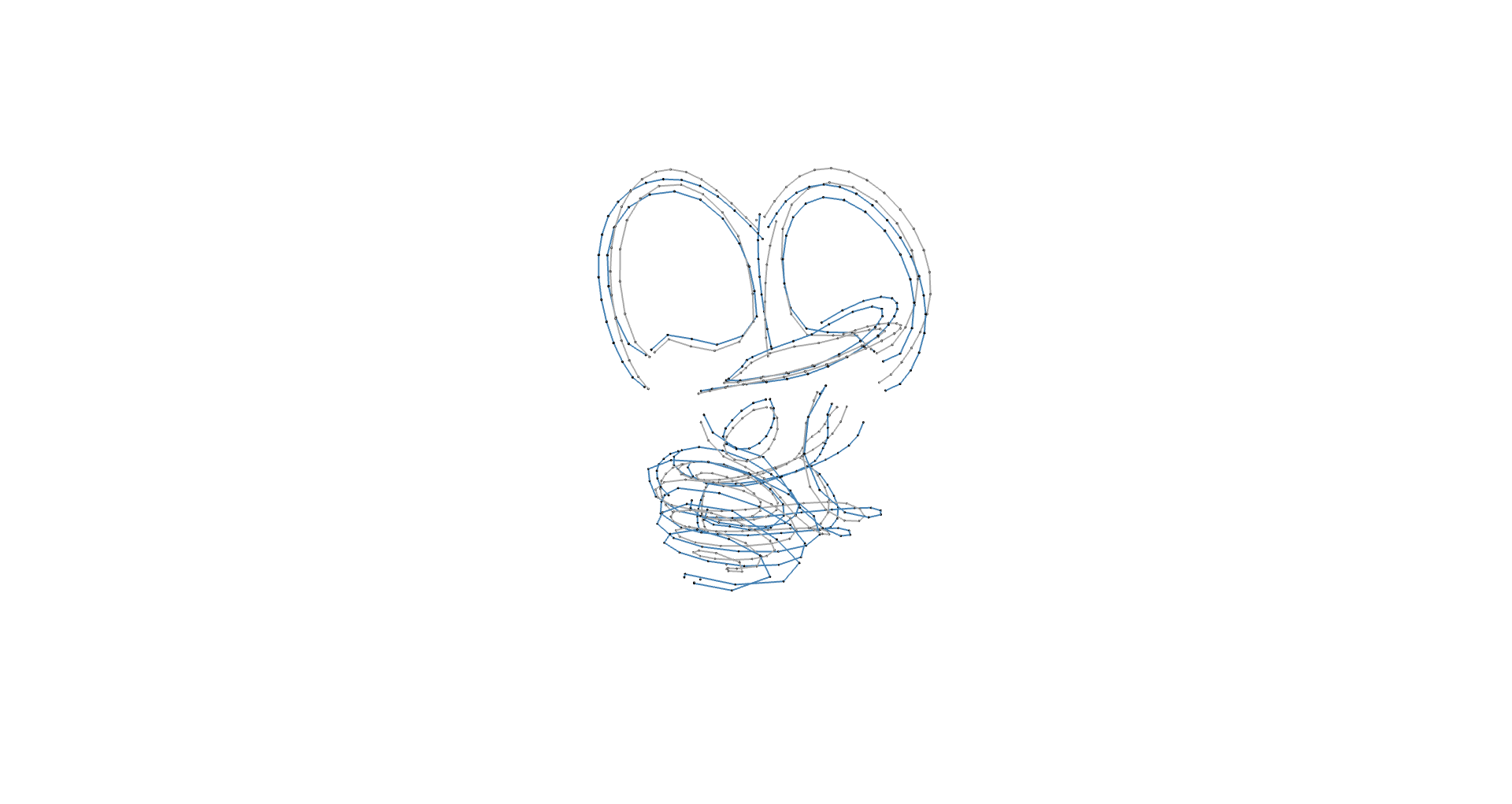

Supplement: Supplementary file 3 — Supplementary Data 1 [file 41467_2022_34656_MOESM3_ESM.zip › Supplementary data_1/Supplementary_material_1-1 Geometric morphometrics/PCA_306/Extreme_shapes_PCA/PC1min-vl.png]

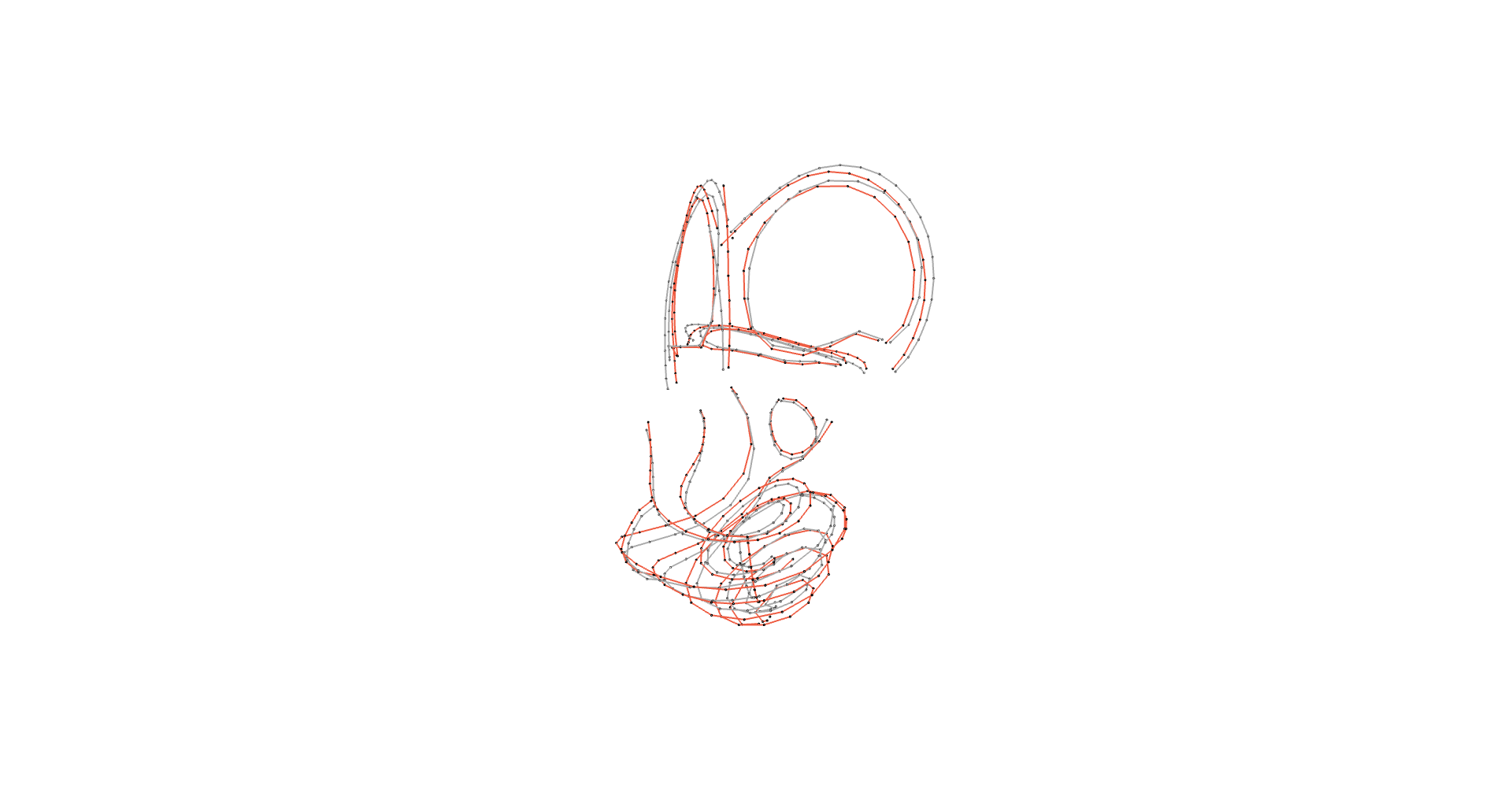

Supplement: Supplementary file 3 — Supplementary Data 1 [file 41467_2022_34656_MOESM3_ESM.zip › Supplementary data_1/Supplementary_material_1-1 Geometric morphometrics/PCA_306/Extreme_shapes_PCA/PC2max-dl.png]

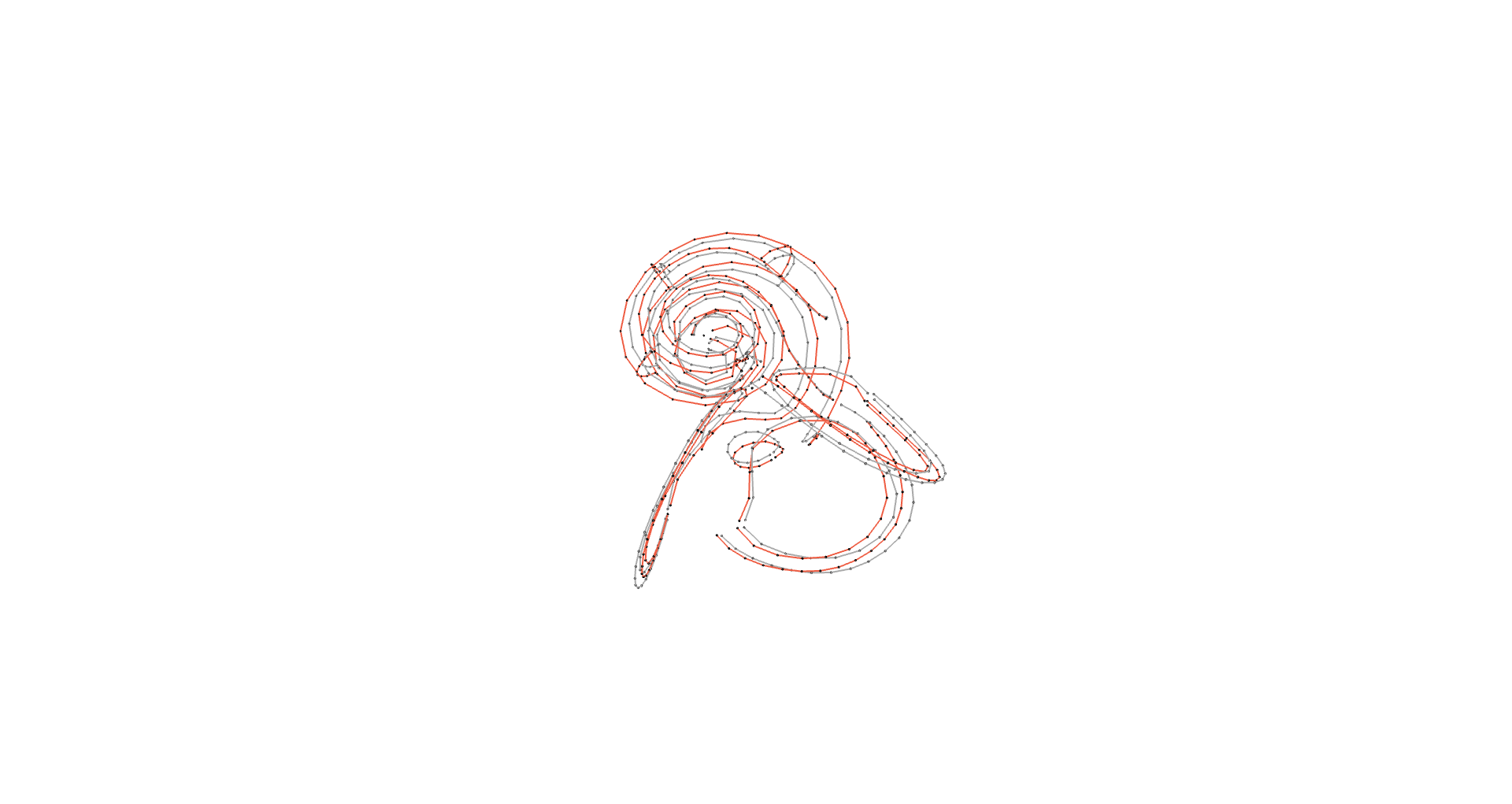

Supplement: Supplementary file 3 — Supplementary Data 1 [file 41467_2022_34656_MOESM3_ESM.zip › Supplementary data_1/Supplementary_material_1-1 Geometric morphometrics/PCA_306/Extreme_shapes_PCA/PC2max-do.png]

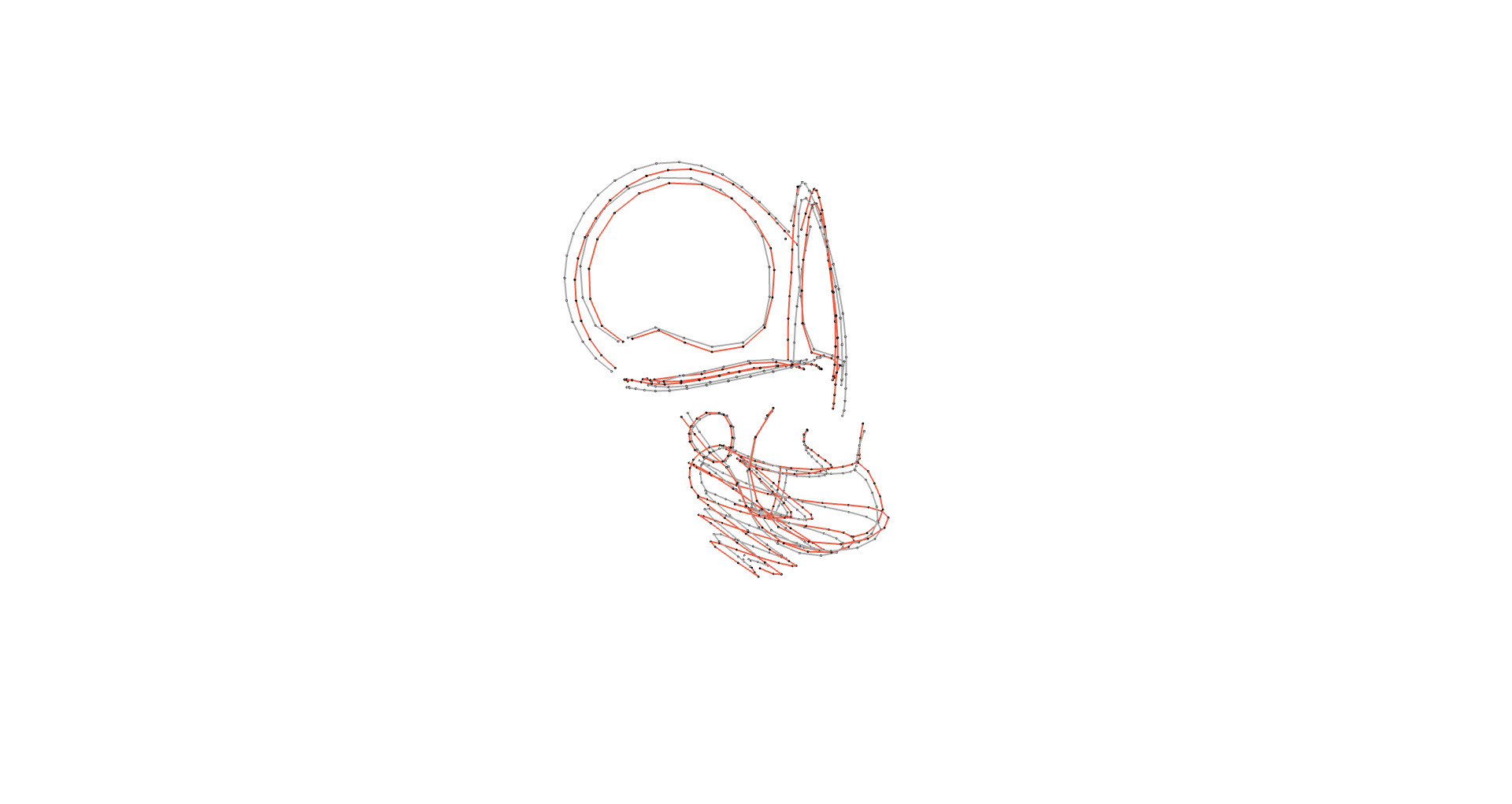

Supplement: Supplementary file 3 — Supplementary Data 1 [file 41467_2022_34656_MOESM3_ESM.zip › Supplementary data_1/Supplementary_material_1-1 Geometric morphometrics/PCA_306/Extreme_shapes_PCA/PC2max-la.png]

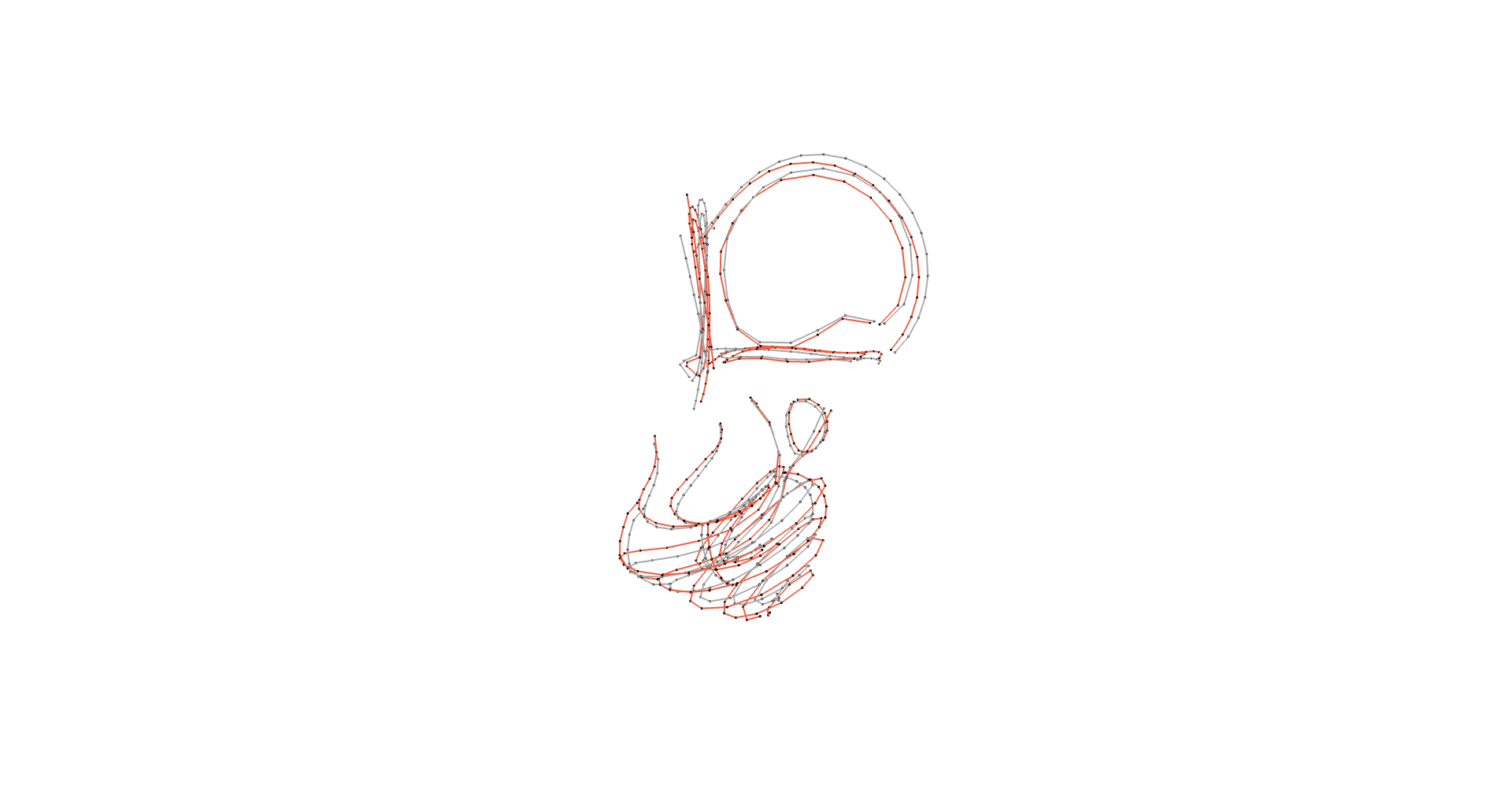

Supplement: Supplementary file 3 — Supplementary Data 1 [file 41467_2022_34656_MOESM3_ESM.zip › Supplementary data_1/Supplementary_material_1-1 Geometric morphometrics/PCA_306/Extreme_shapes_PCA/PC2max-me.png]

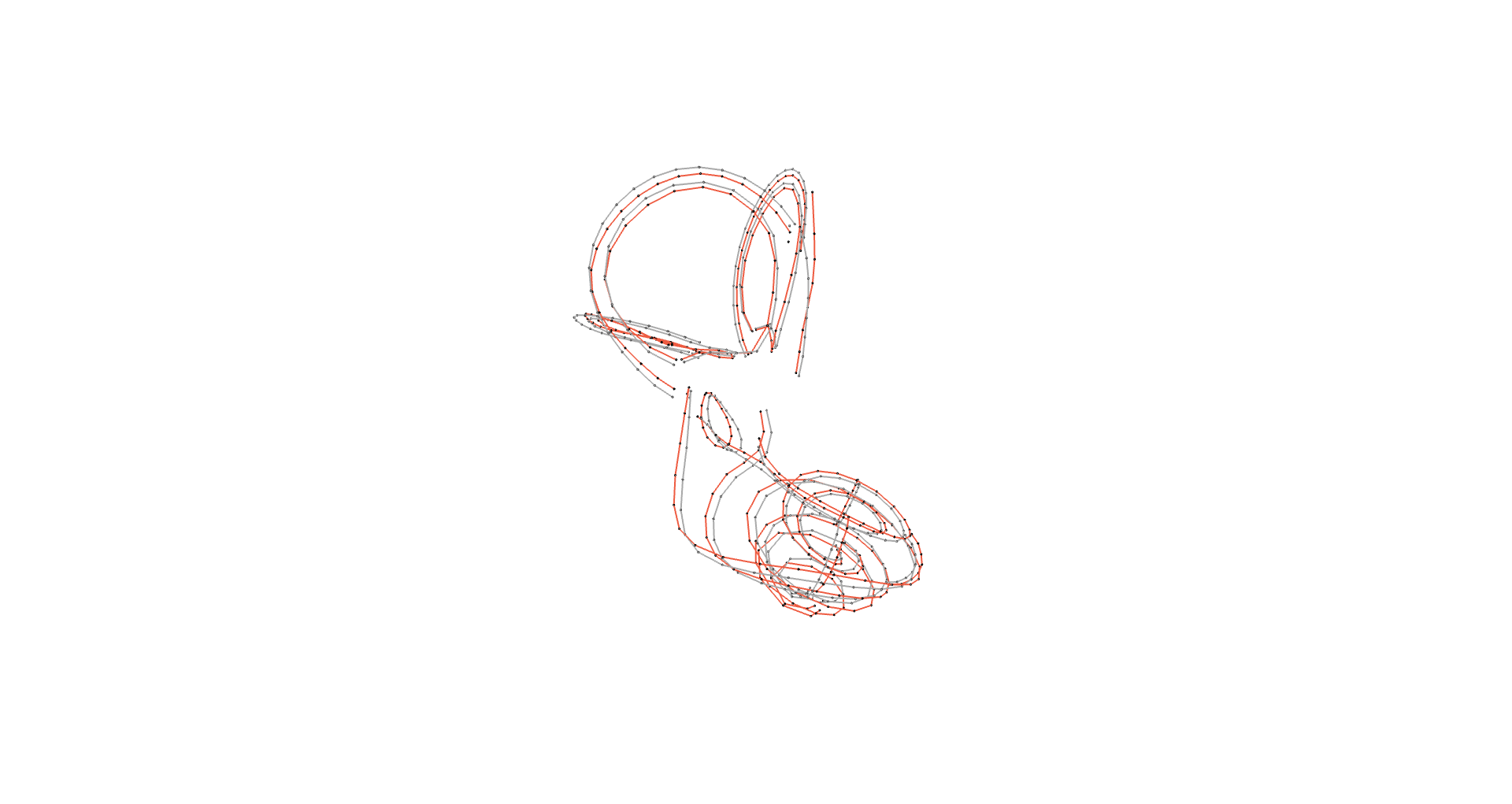

Supplement: Supplementary file 3 — Supplementary Data 1 [file 41467_2022_34656_MOESM3_ESM.zip › Supplementary data_1/Supplementary_material_1-1 Geometric morphometrics/PCA_306/Extreme_shapes_PCA/PC2max-oc.png]

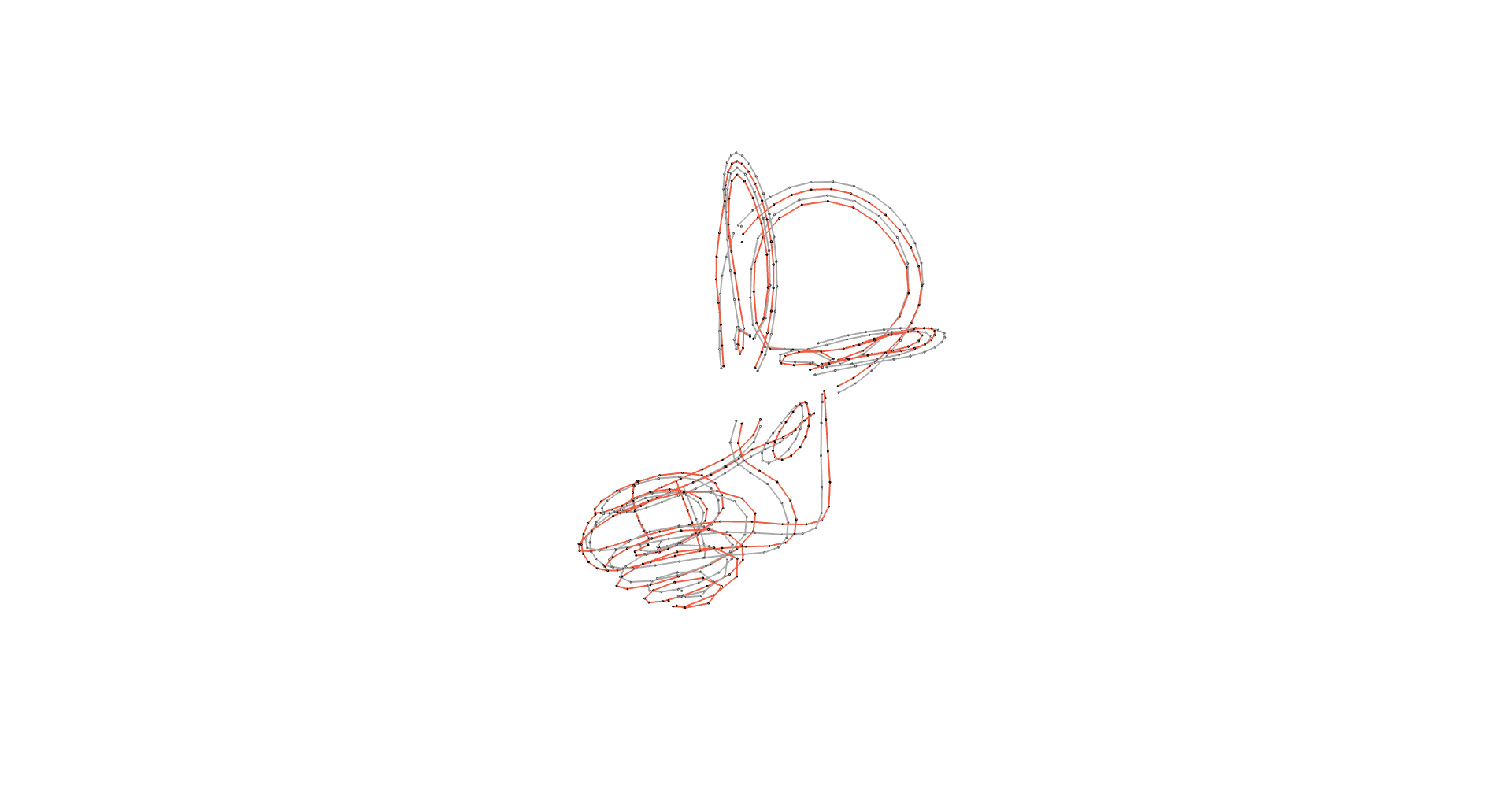

Supplement: Supplementary file 3 — Supplementary Data 1 [file 41467_2022_34656_MOESM3_ESM.zip › Supplementary data_1/Supplementary_material_1-1 Geometric morphometrics/PCA_306/Extreme_shapes_PCA/PC2max-ro.png]

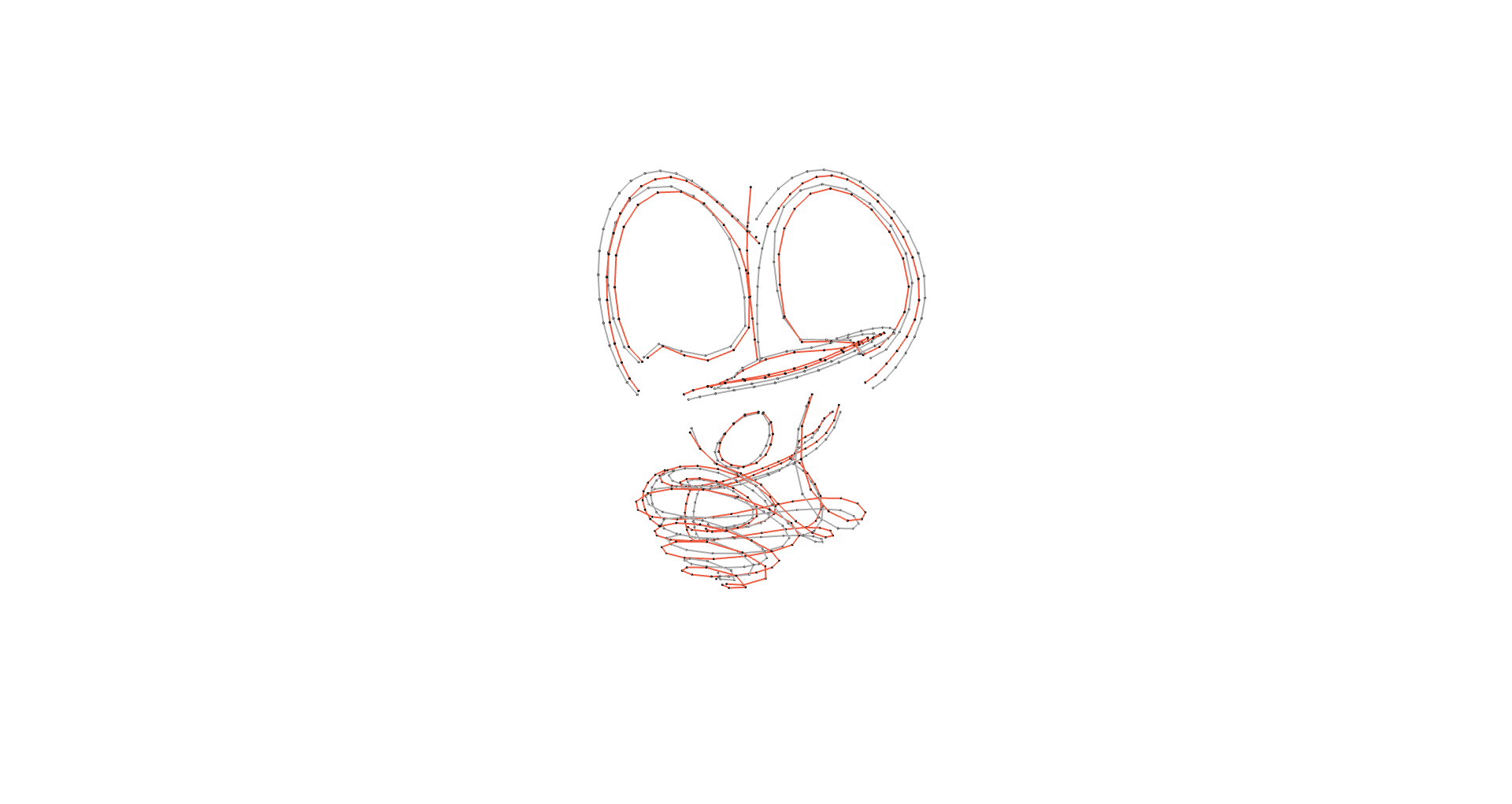

Supplement: Supplementary file 3 — Supplementary Data 1 [file 41467_2022_34656_MOESM3_ESM.zip › Supplementary data_1/Supplementary_material_1-1 Geometric morphometrics/PCA_306/Extreme_shapes_PCA/PC2max-vl.png]

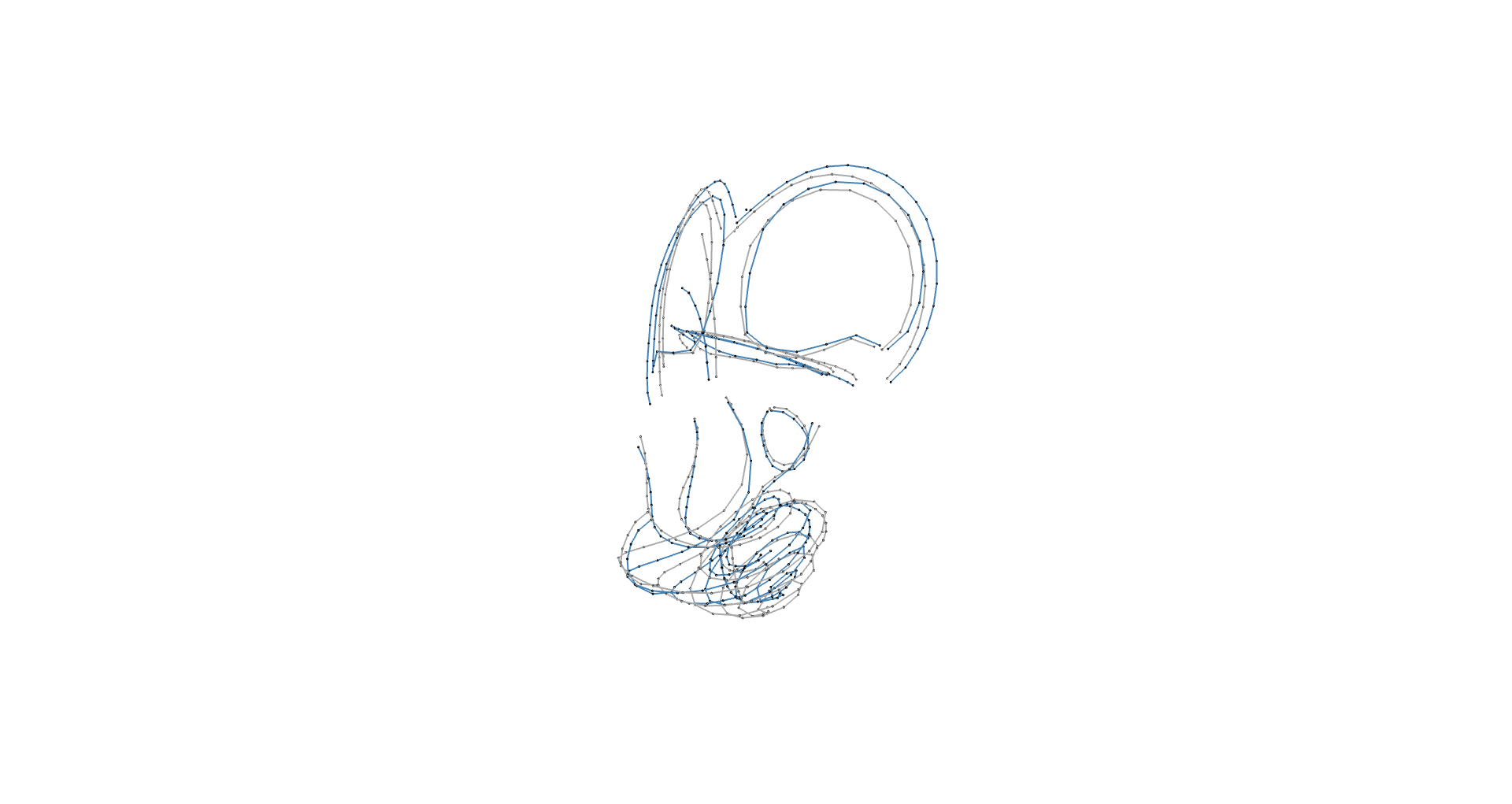

Supplement: Supplementary file 3 — Supplementary Data 1 [file 41467_2022_34656_MOESM3_ESM.zip › Supplementary data_1/Supplementary_material_1-1 Geometric morphometrics/PCA_306/Extreme_shapes_PCA/PC2min-dl.png]

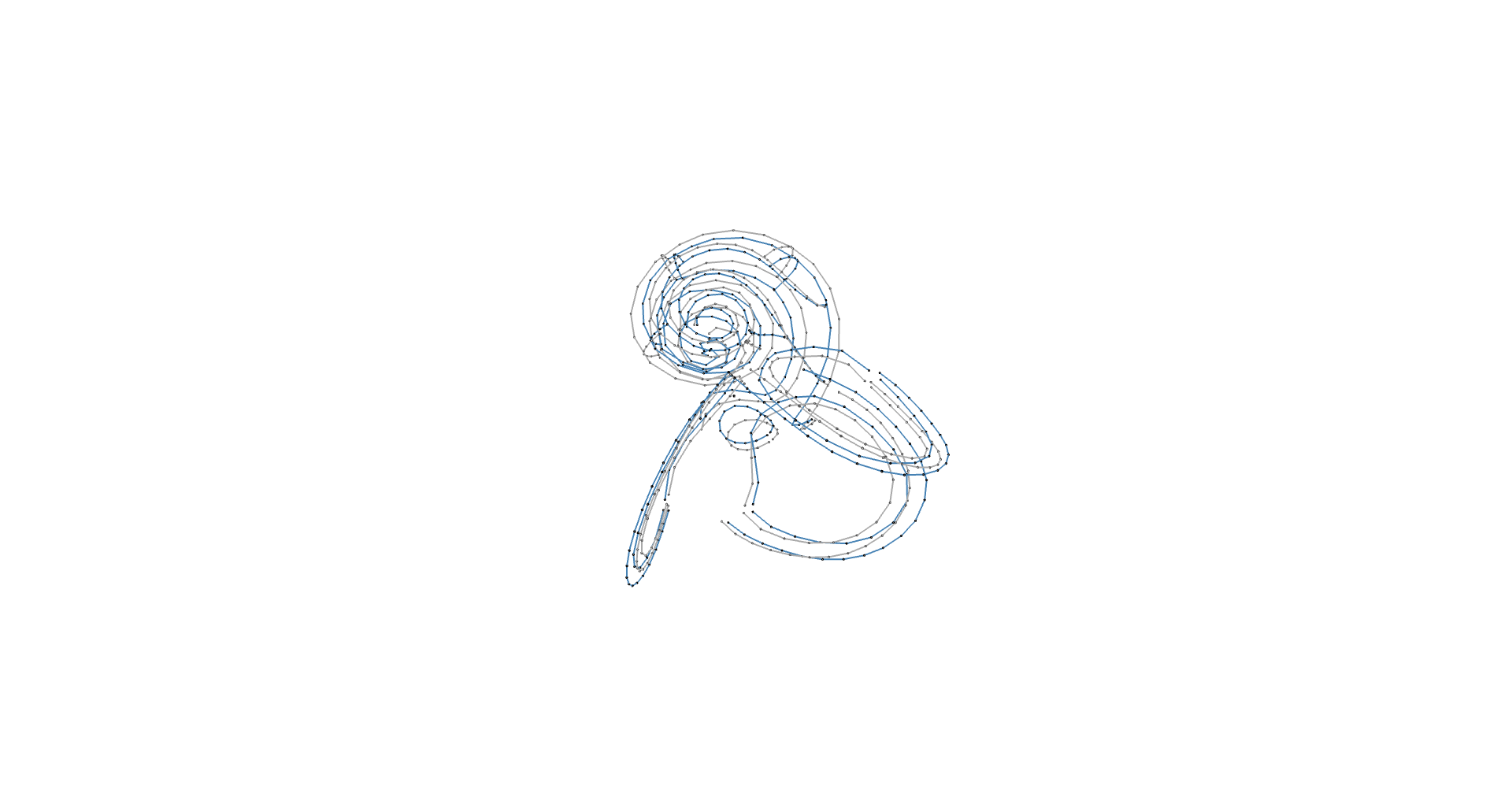

Supplement: Supplementary file 3 — Supplementary Data 1 [file 41467_2022_34656_MOESM3_ESM.zip › Supplementary data_1/Supplementary_material_1-1 Geometric morphometrics/PCA_306/Extreme_shapes_PCA/PC2min-do.png]

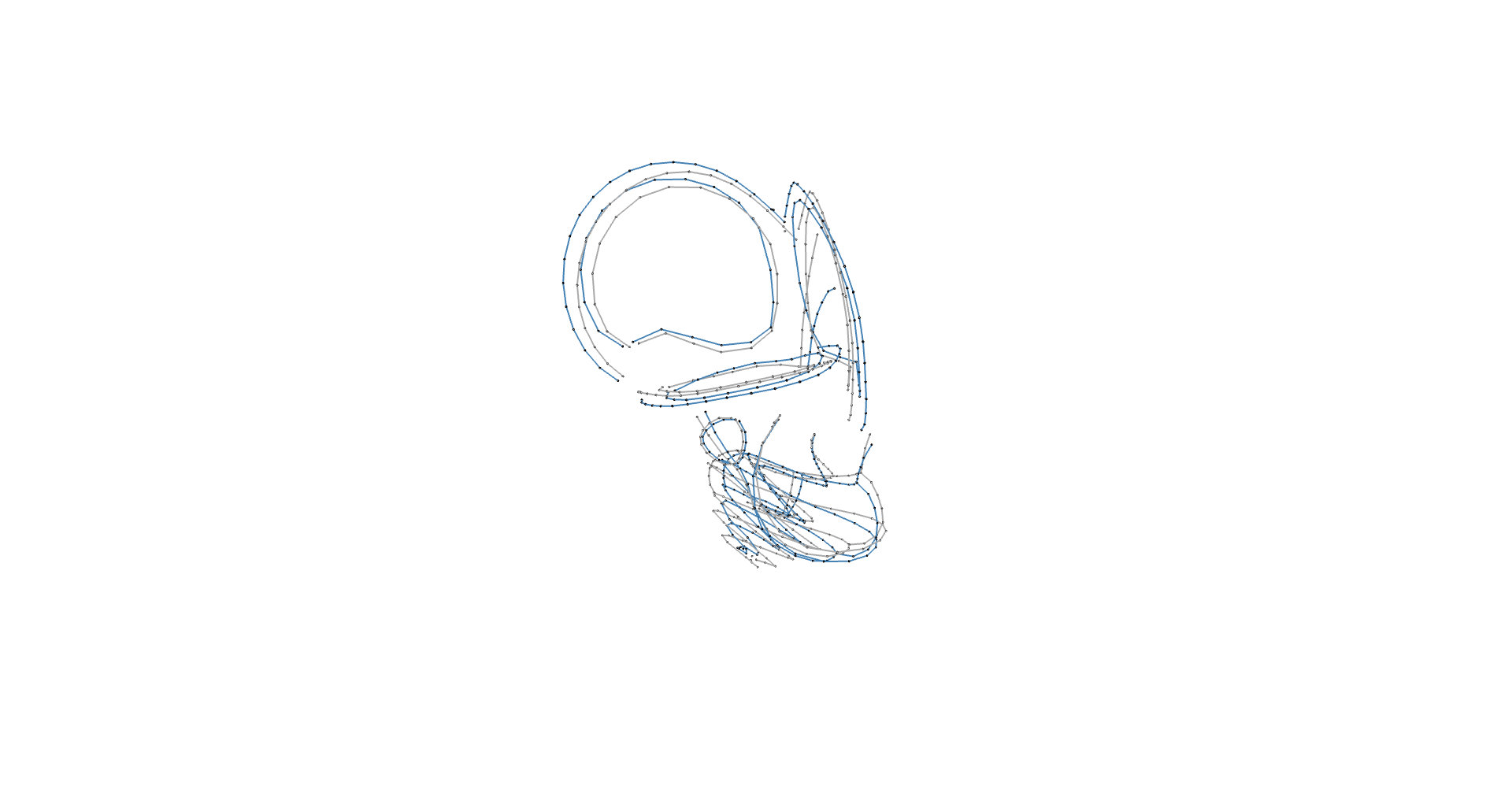

Supplement: Supplementary file 3 — Supplementary Data 1 [file 41467_2022_34656_MOESM3_ESM.zip › Supplementary data_1/Supplementary_material_1-1 Geometric morphometrics/PCA_306/Extreme_shapes_PCA/PC2min-la.png]

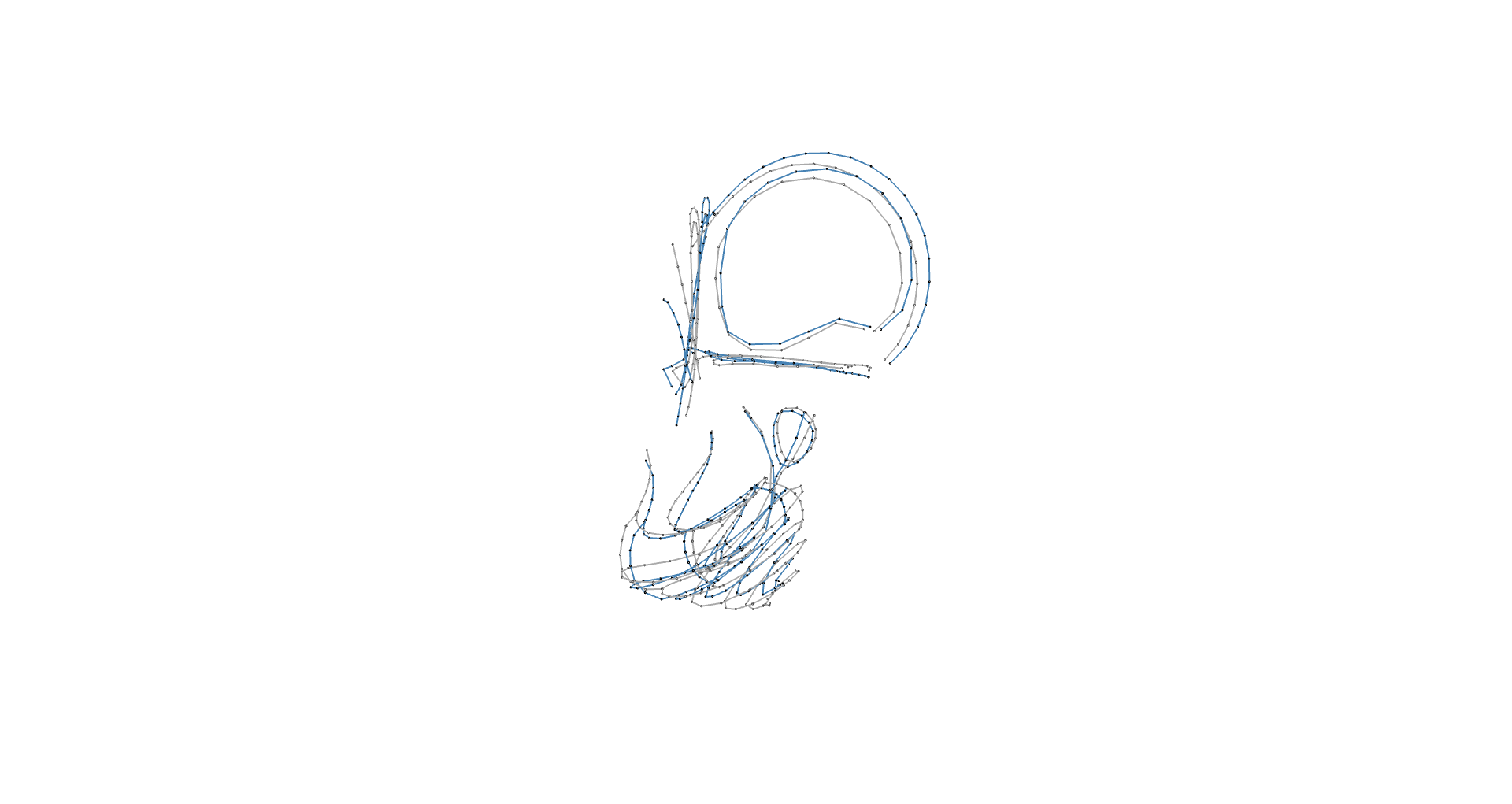

Supplement: Supplementary file 3 — Supplementary Data 1 [file 41467_2022_34656_MOESM3_ESM.zip › Supplementary data_1/Supplementary_material_1-1 Geometric morphometrics/PCA_306/Extreme_shapes_PCA/PC2min-me.png]

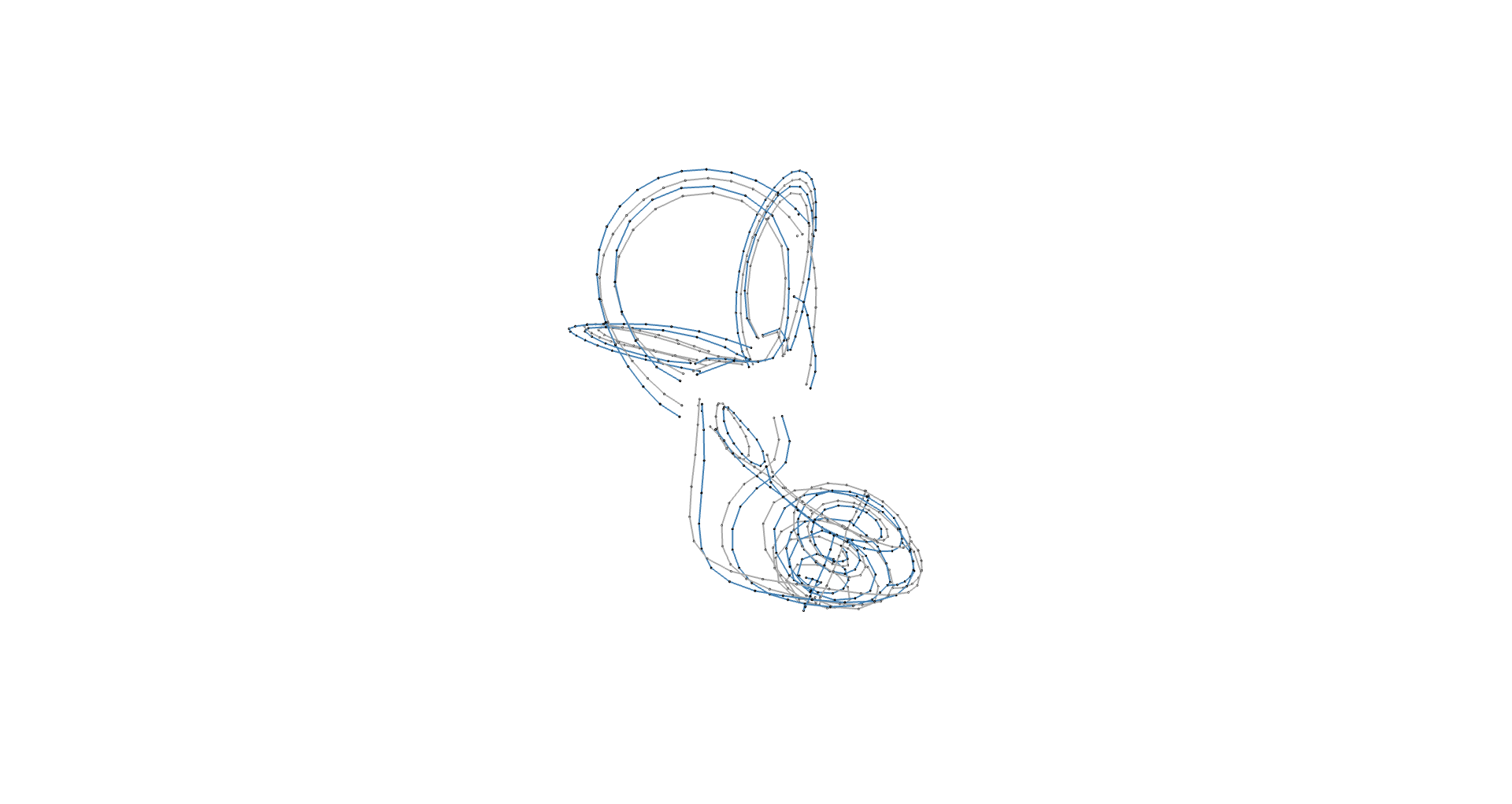

Supplement: Supplementary file 3 — Supplementary Data 1 [file 41467_2022_34656_MOESM3_ESM.zip › Supplementary data_1/Supplementary_material_1-1 Geometric morphometrics/PCA_306/Extreme_shapes_PCA/PC2min-oc.png]

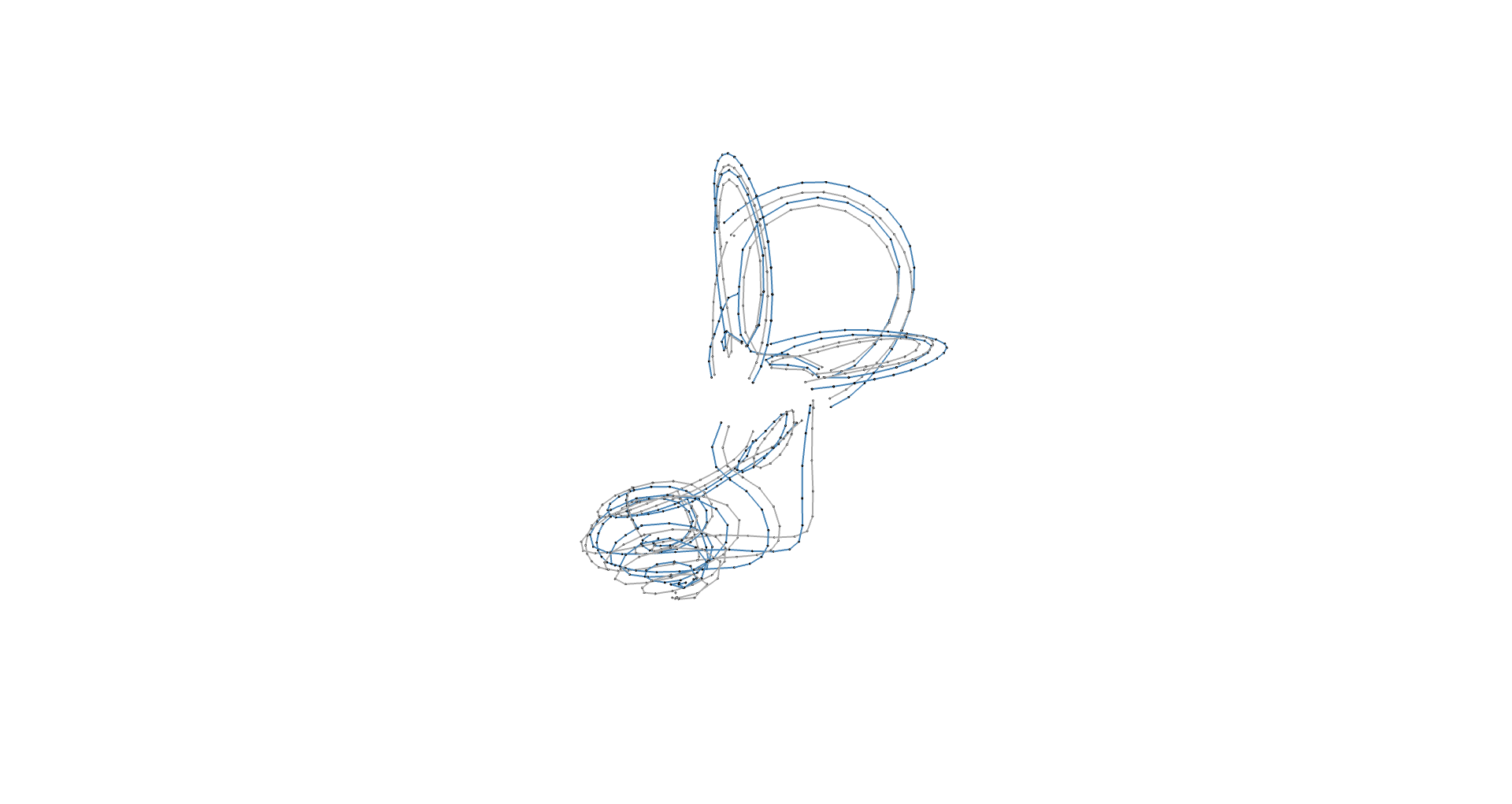

Supplement: Supplementary file 3 — Supplementary Data 1 [file 41467_2022_34656_MOESM3_ESM.zip › Supplementary data_1/Supplementary_material_1-1 Geometric morphometrics/PCA_306/Extreme_shapes_PCA/PC2min-ro.png]

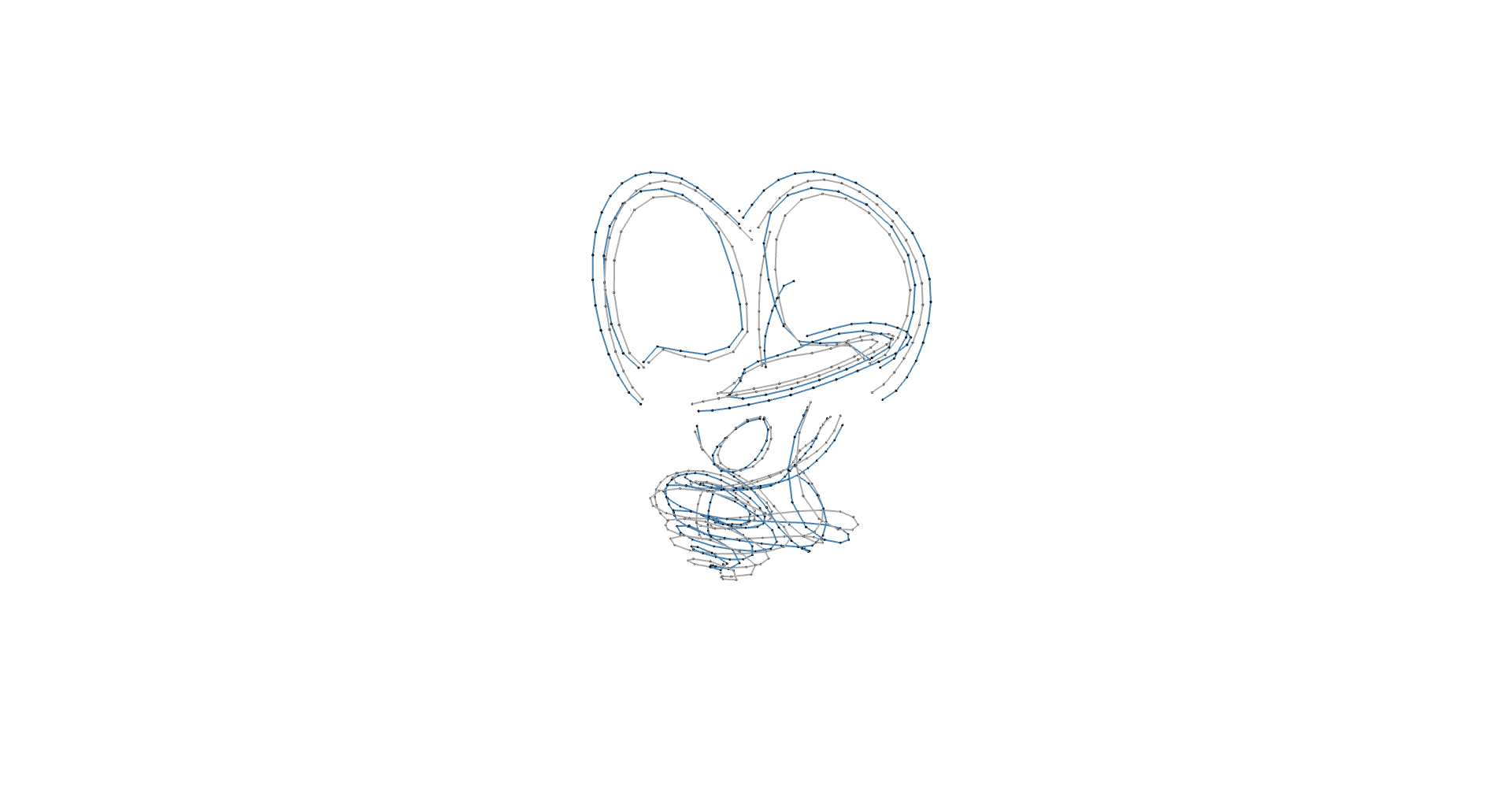

Supplement: Supplementary file 3 — Supplementary Data 1 [file 41467_2022_34656_MOESM3_ESM.zip › Supplementary data_1/Supplementary_material_1-1 Geometric morphometrics/PCA_306/Extreme_shapes_PCA/PC2min-vl.png]

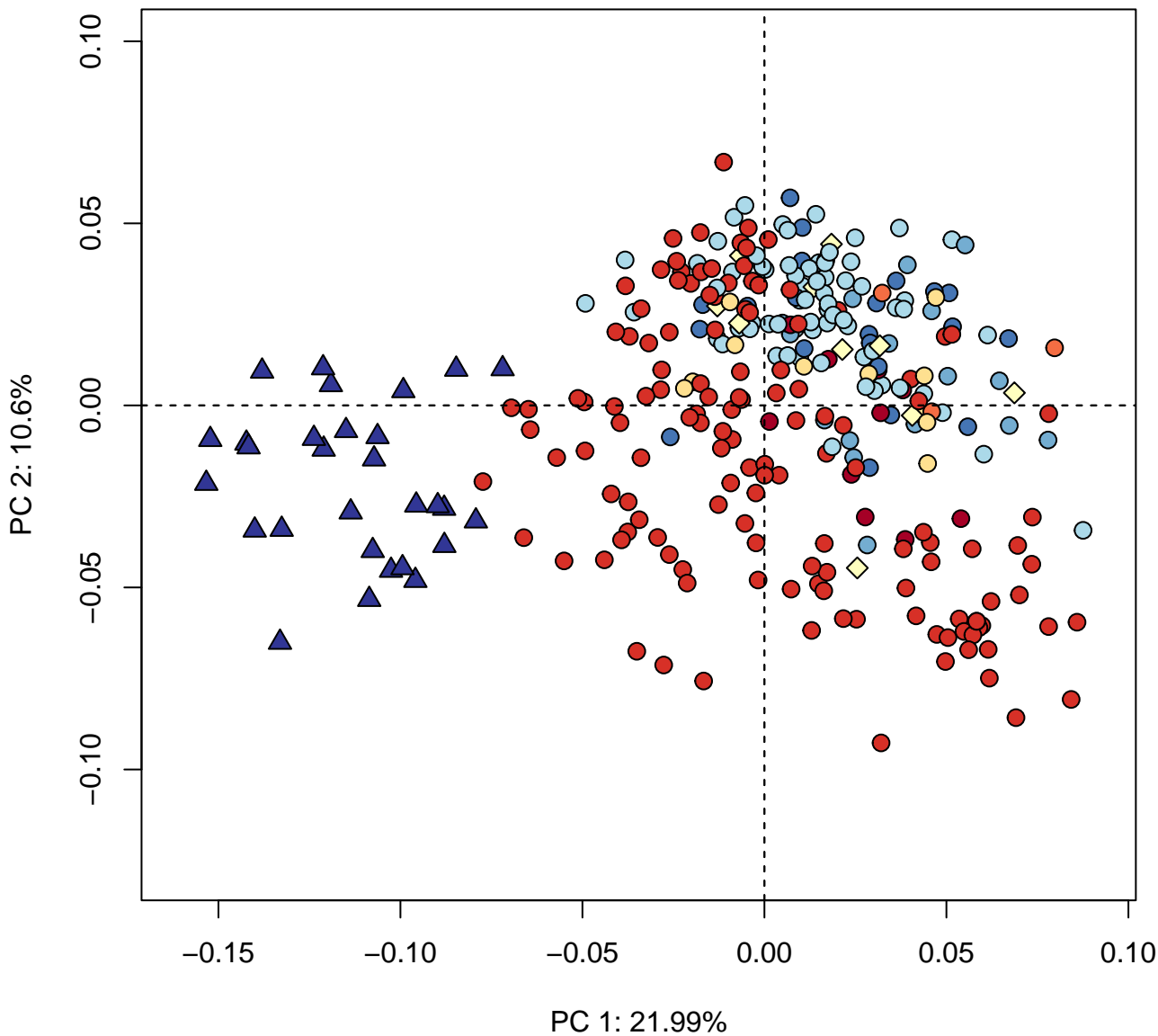

Supplement: Supplementary file 3 — Supplementary Data 1 [file 41467_2022_34656_MOESM3_ESM.zip › Supplementary data_1/Supplementary_material_1-1 Geometric morphometrics/PCA_306/PCA_306_rum.pdf]

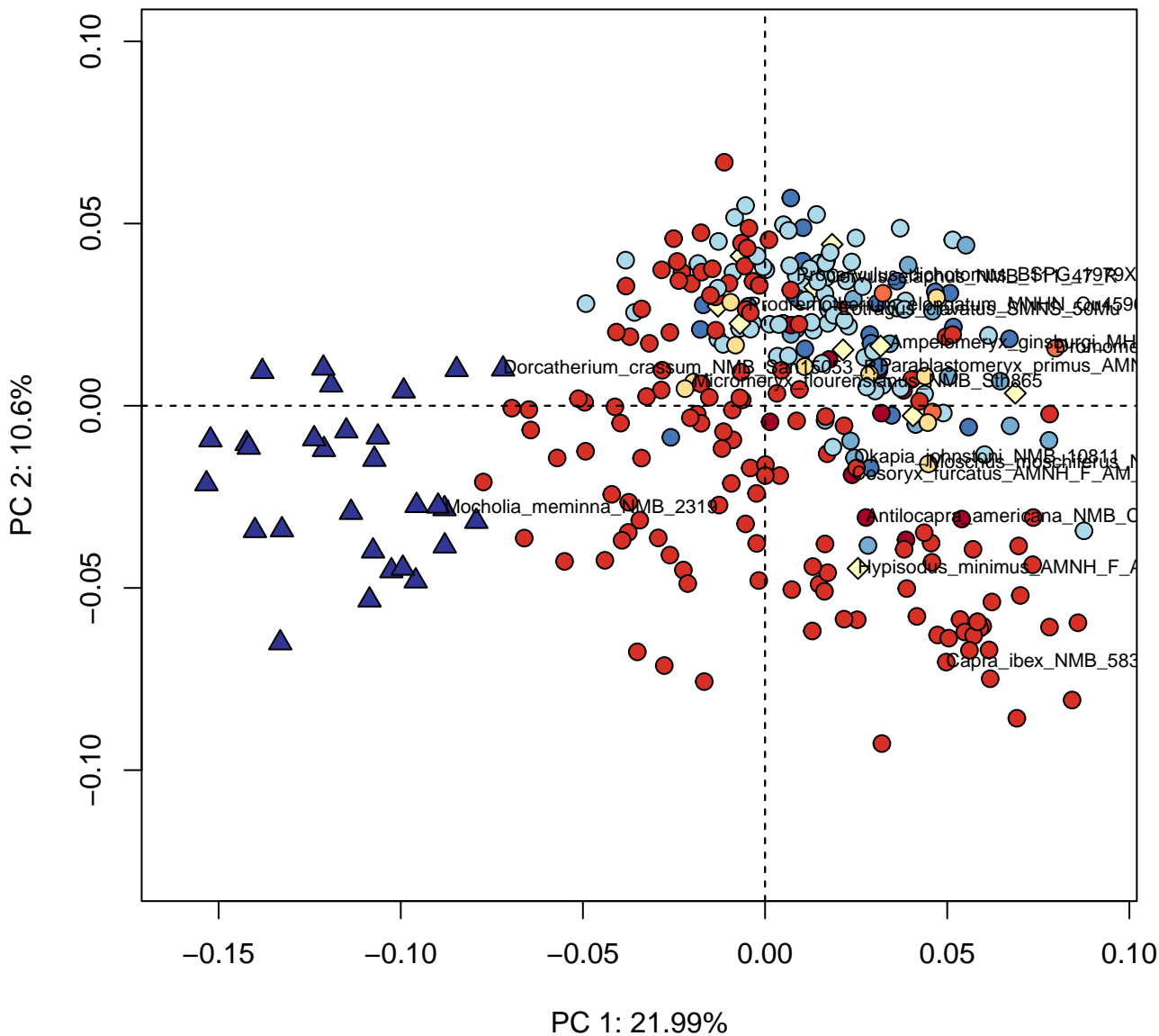

Supplement: Supplementary file 3 — Supplementary Data 1 [file 41467_2022_34656_MOESM3_ESM.zip › Supplementary data_1/Supplementary_material_1-1 Geometric morphometrics/PCA_306/PCA_306_rum_names.pdf]

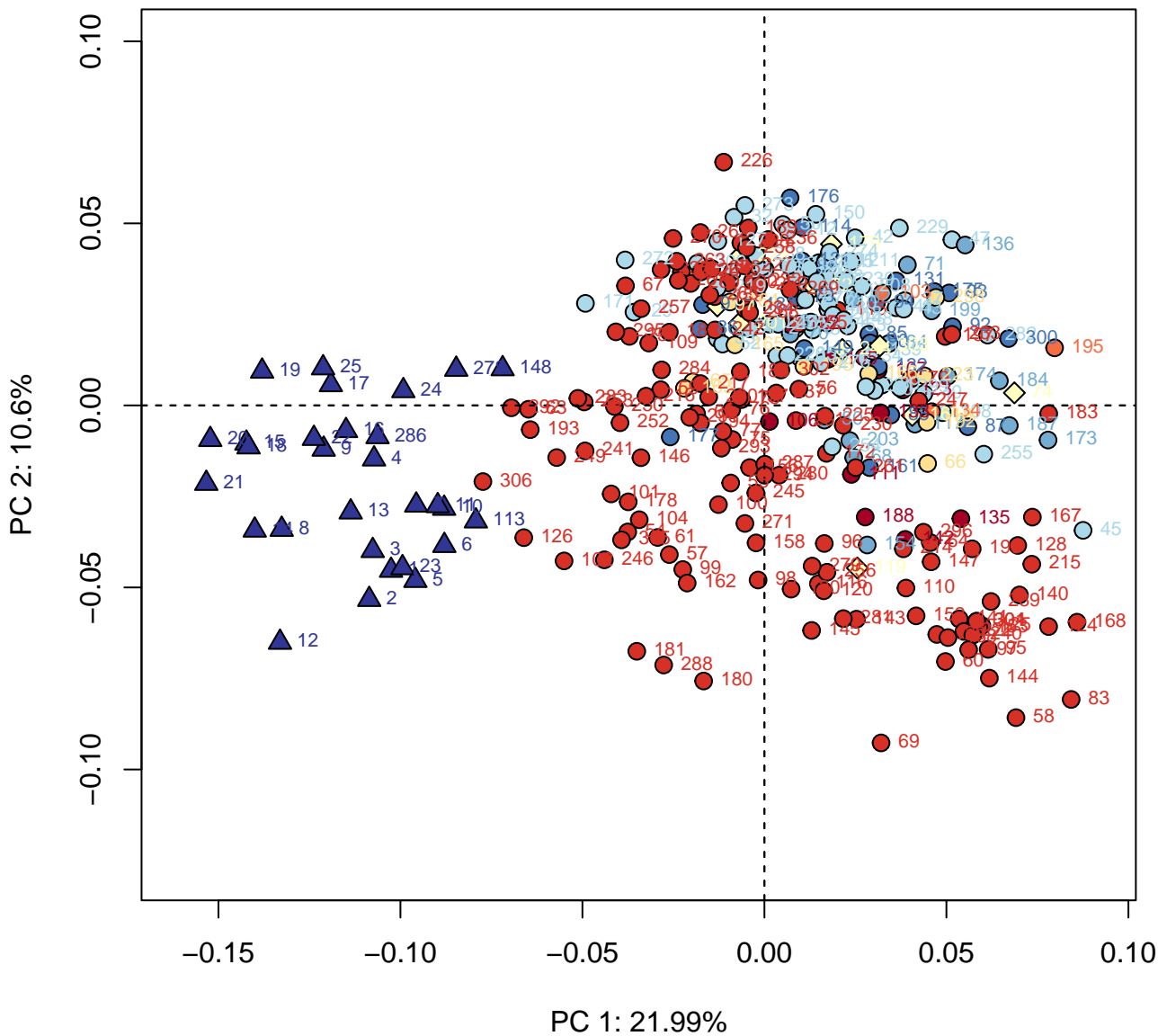

Supplement: Supplementary file 3 — Supplementary Data 1 [file 41467_2022_34656_MOESM3_ESM.zip › Supplementary data_1/Supplementary_material_1-1 Geometric morphometrics/PCA_306/PCA_306_rum_numbers.pdf]

0 trait value 0.6  
length=22.5

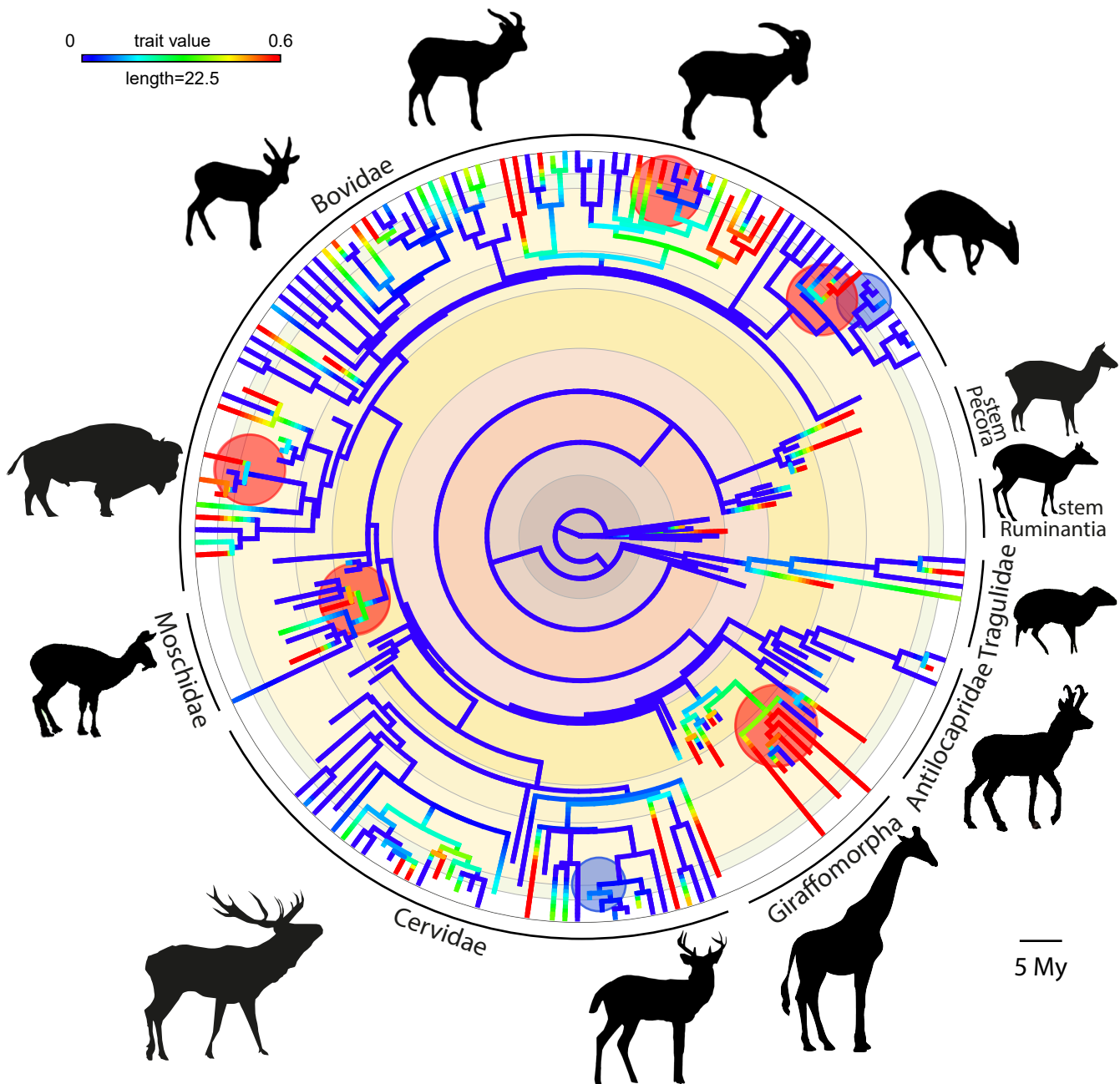

Supplement: Supplementary file 3 — Supplementary Data 1 [file 41467_2022_34656_MOESM3_ESM.zip › Supplementary data_1/Supplementary_material_1-2 RRphylo/centroid/CENTROID_evorates.pdf]

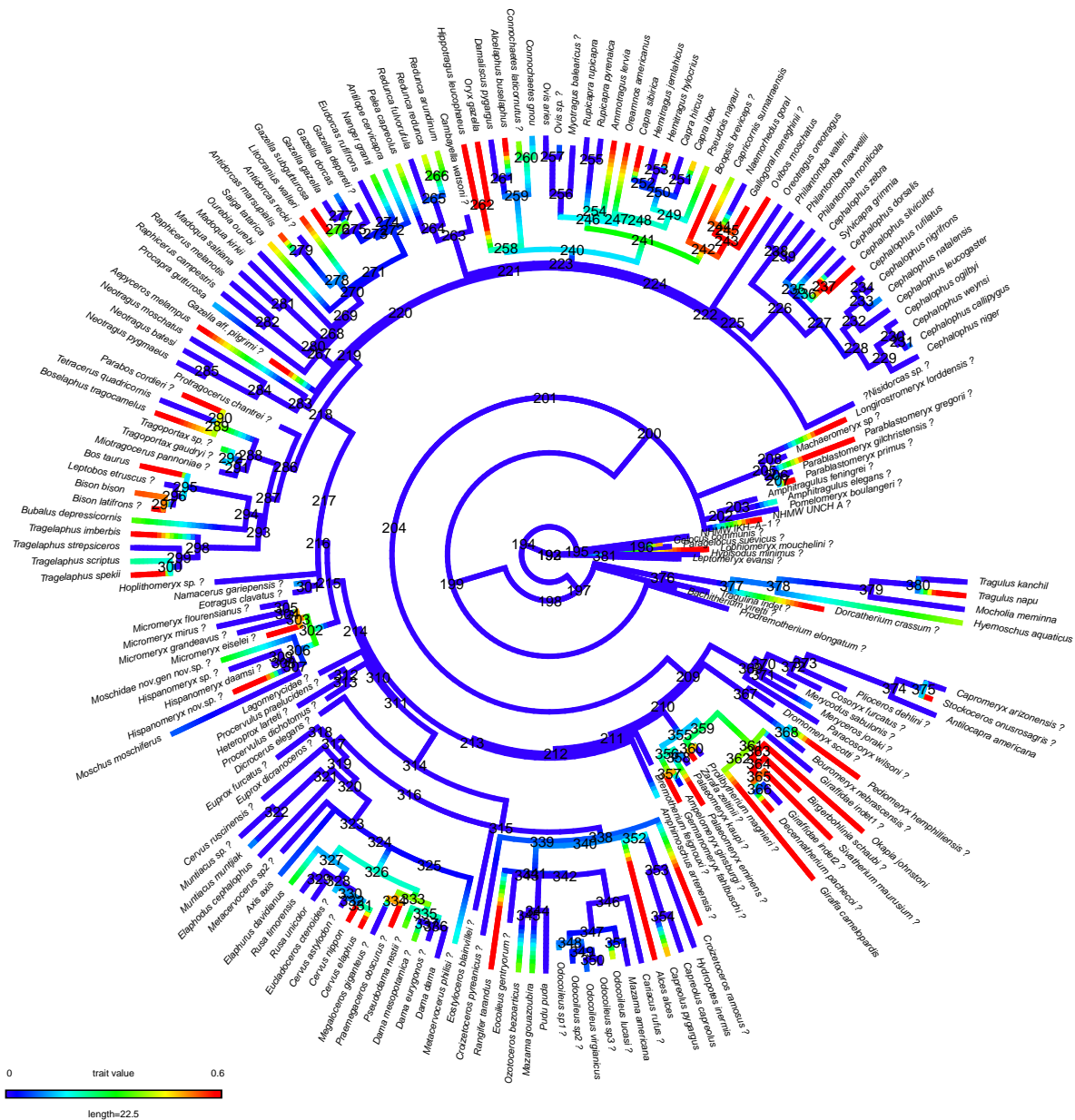

Supplement: Supplementary file 3 — Supplementary Data 1 [file 41467_2022_34656_MOESM3_ESM.zip › Supplementary data_1/Supplementary_material_1-2 RRphylo/centroid/CENTROID_evorates_nodes.pdf]

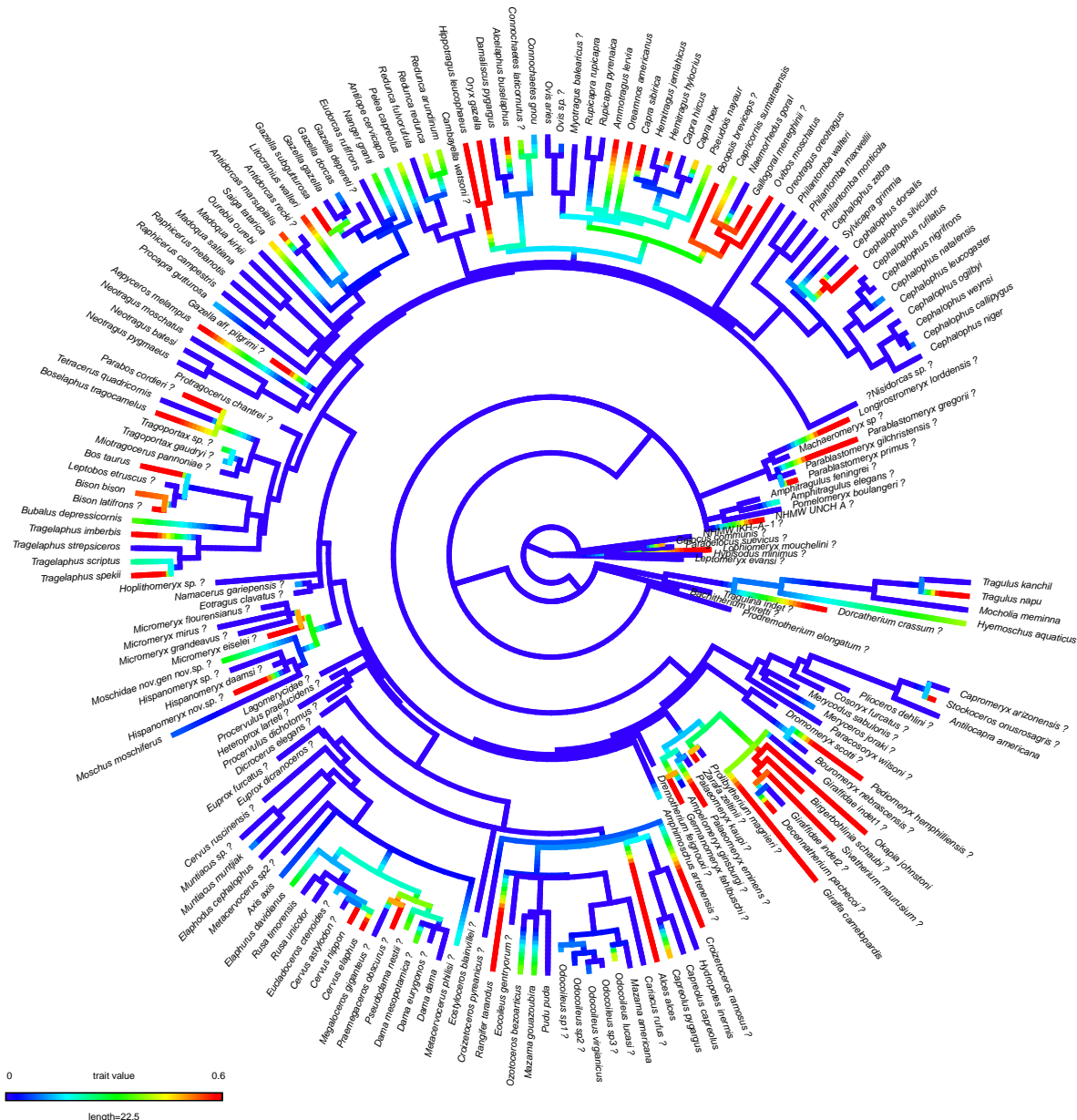

Supplement: Supplementary file 3 — Supplementary Data 1 [file 41467_2022_34656_MOESM3_ESM.zip › Supplementary data_1/Supplementary_material_1-2 RRphylo/centroid/CENTROID_evorates_no_ancestral_states.pdf]

60      trait value      130  
length=22.5

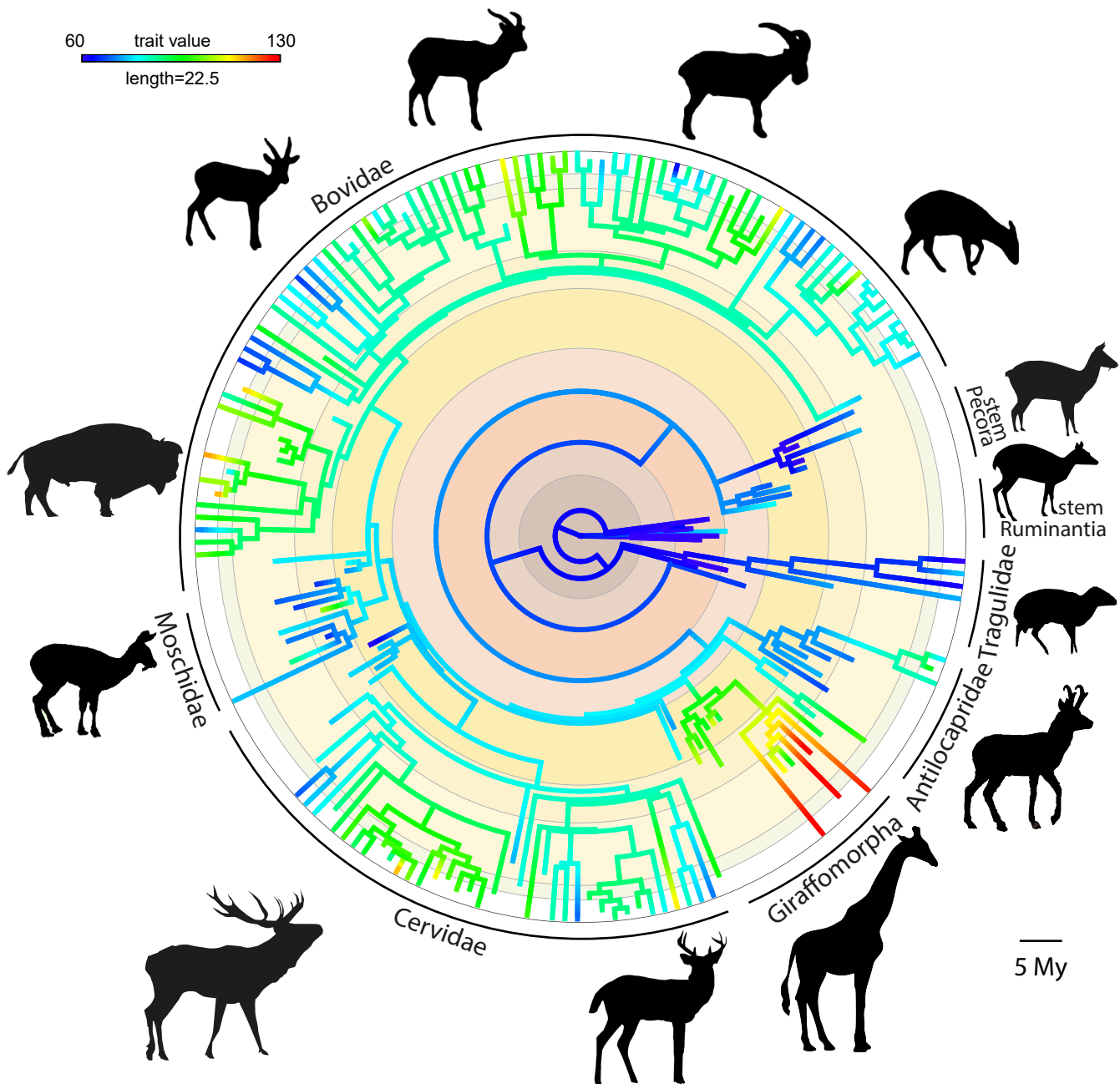

Supplement: Supplementary file 3 — Supplementary Data 1 [file 41467_2022_34656_MOESM3_ESM.zip › Supplementary data_1/Supplementary_material_1-2 RRphylo/centroid/centroid_size_phylo.pdf]
