# Supplementary figures and images for: Ruminant inner ear shape records 35 million years of neutral evolution (part 1 of 2)
Source: Nat Commun. 2022 Dec 6;13:7222. doi: 10.1038/s41467-022-34656-0 (PMC9726890; doi:10.1038/s41467-022-34656-0)

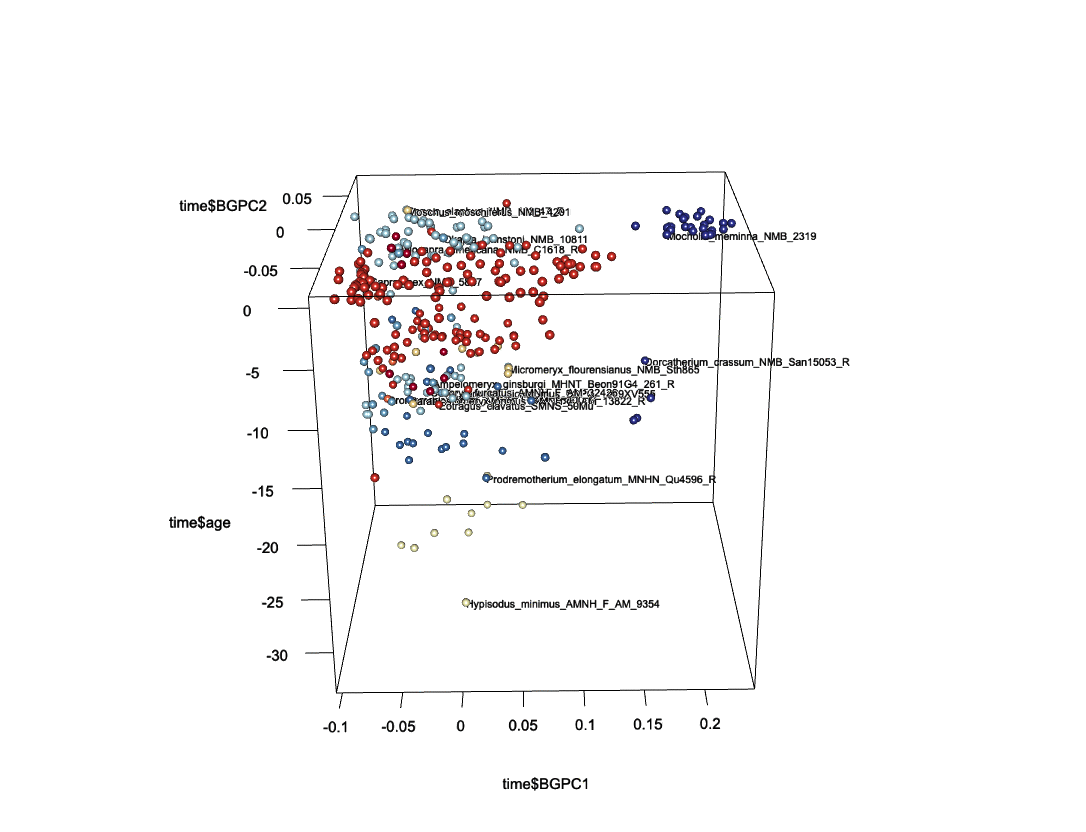

Supplement: Supplementary file 3 — Supplementary Data 1 [file 41467_2022_34656_MOESM3_ESM.zip › Supplementary data_1/Supplementary_material_1-1 Geometric morphometrics/Animated 3Dgraphs_gif/3Dgraphs_view_1_top/BGPCA-3dAnimatedScatterplot.gif]

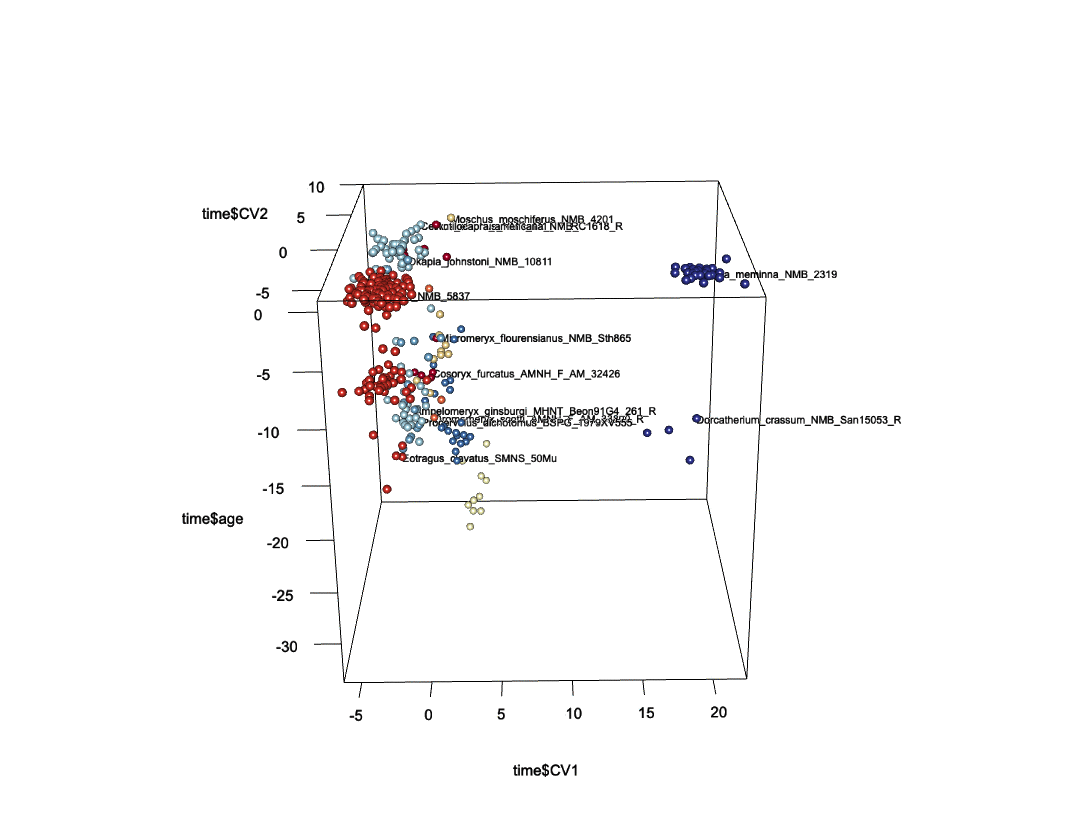

Supplement: Supplementary file 3 — Supplementary Data 1 [file 41467_2022_34656_MOESM3_ESM.zip › Supplementary data_1/Supplementary_material_1-1 Geometric morphometrics/Animated 3Dgraphs_gif/3Dgraphs_view_1_top/CVA-3dAnimatedScatterplot.gif]

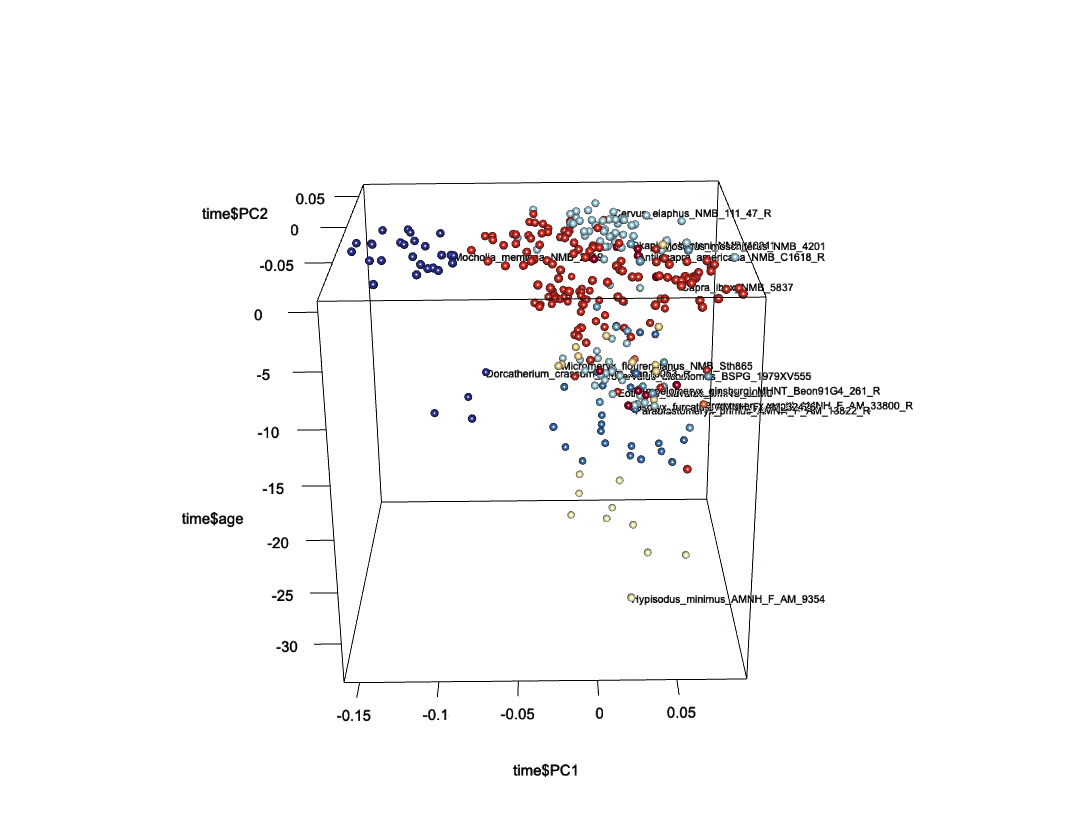

Supplement: Supplementary file 3 — Supplementary Data 1 [file 41467_2022_34656_MOESM3_ESM.zip › Supplementary data_1/Supplementary_material_1-1 Geometric morphometrics/Animated 3Dgraphs_gif/3Dgraphs_view_1_top/PCA-3dAnimatedScatterplot.gif]

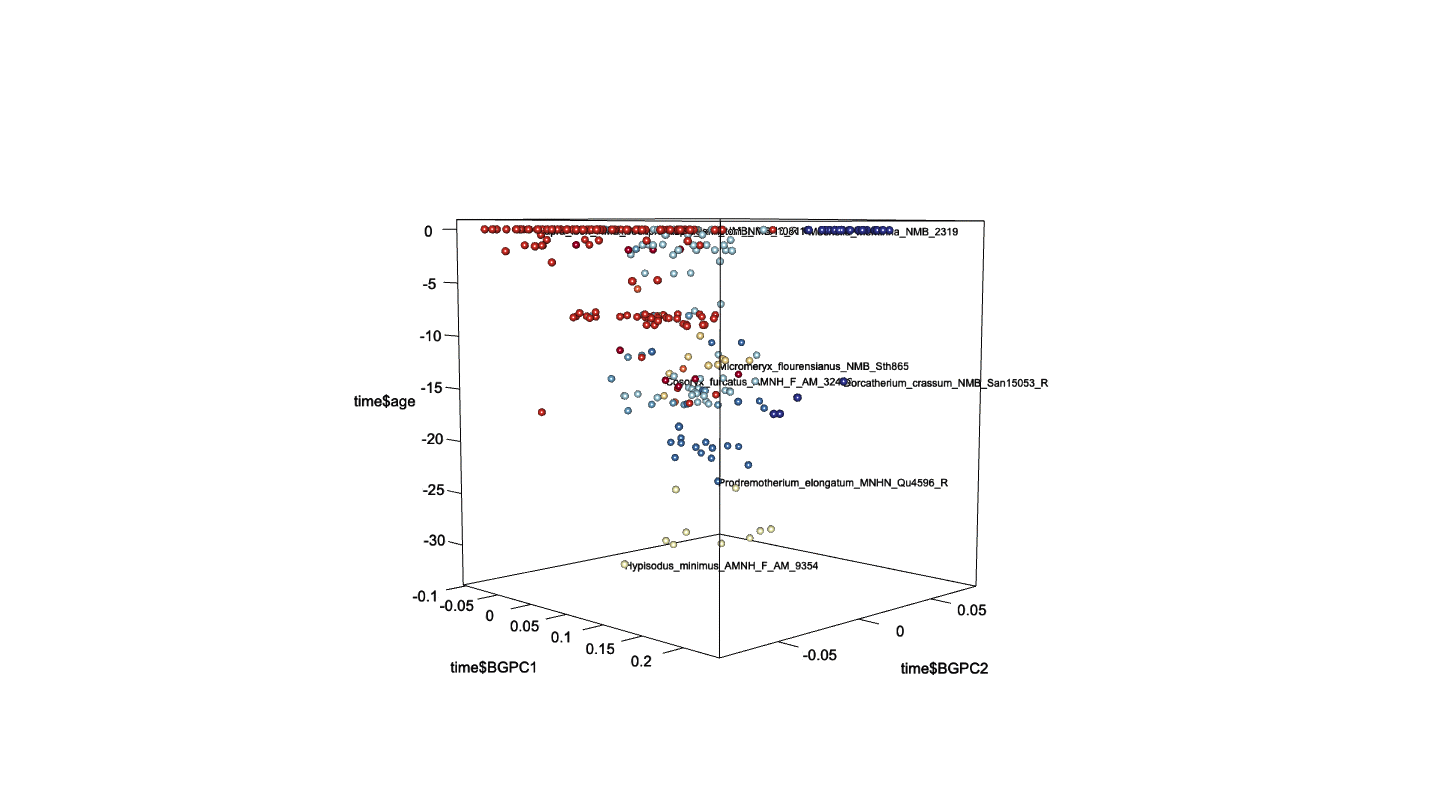

Supplement: Supplementary file 3 — Supplementary Data 1 [file 41467_2022_34656_MOESM3_ESM.zip › Supplementary data_1/Supplementary_material_1-1 Geometric morphometrics/Animated 3Dgraphs_gif/3Dgraphs_view_2_lateral/BGPCA-3dAnimatedScatterplot.gif]

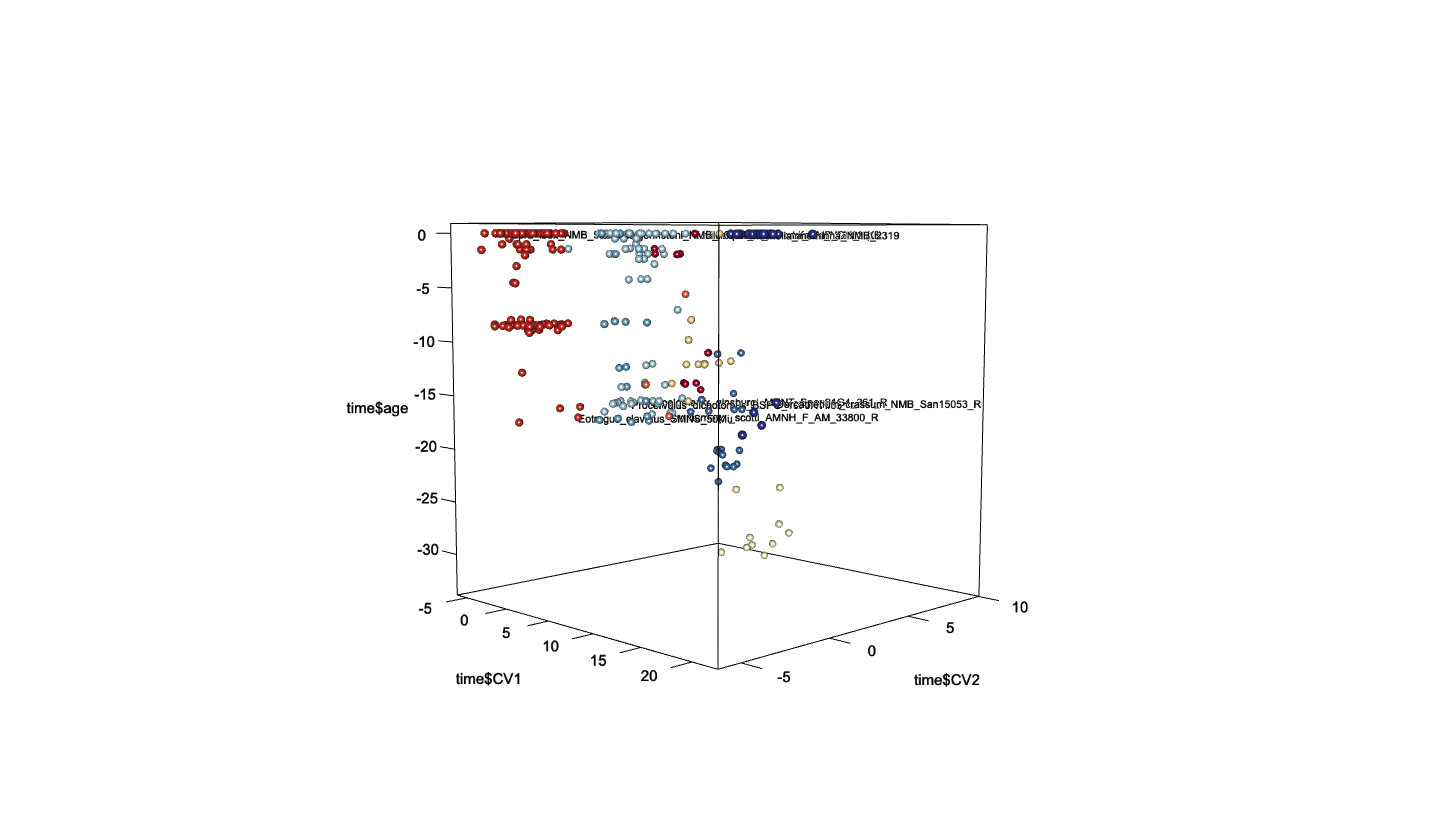

Supplement: Supplementary file 3 — Supplementary Data 1 [file 41467_2022_34656_MOESM3_ESM.zip › Supplementary data_1/Supplementary_material_1-1 Geometric morphometrics/Animated 3Dgraphs_gif/3Dgraphs_view_2_lateral/CVA-3dAnimatedScatterplot.gif]

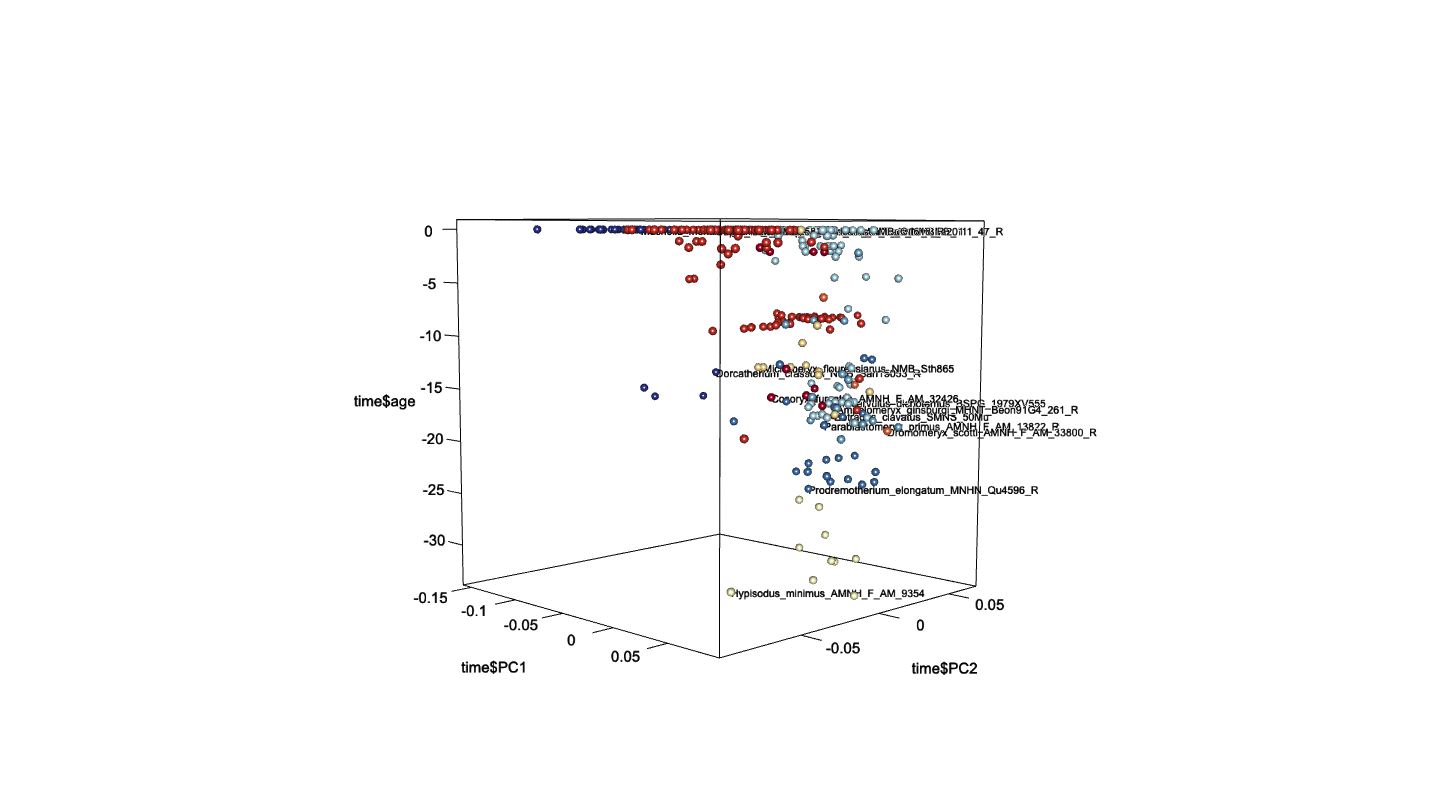

Supplement: Supplementary file 3 — Supplementary Data 1 [file 41467_2022_34656_MOESM3_ESM.zip › Supplementary data_1/Supplementary_material_1-1 Geometric morphometrics/Animated 3Dgraphs_gif/3Dgraphs_view_2_lateral/PCA-3dAnimatedScatterplot.gif]

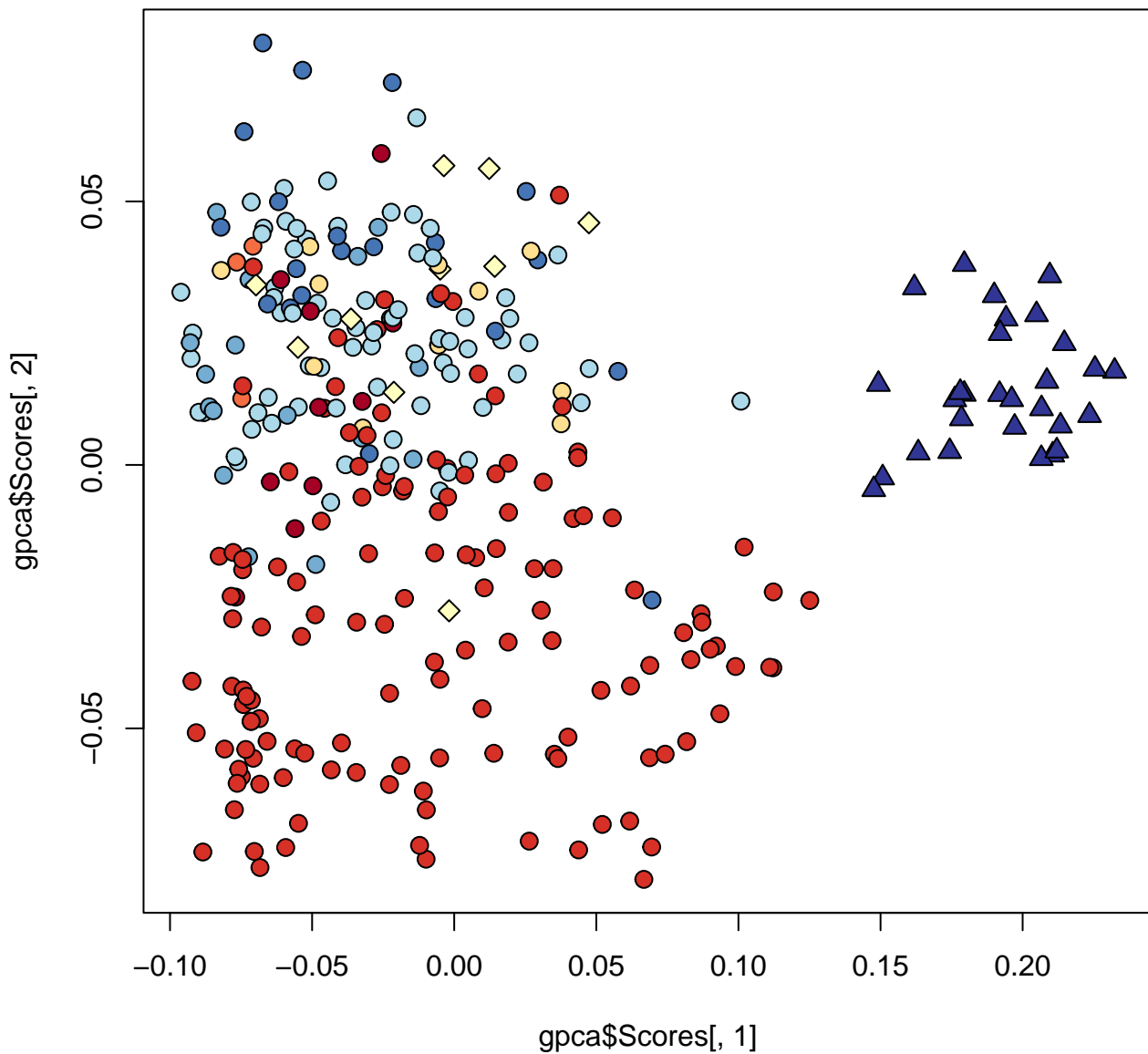

Supplement: Supplementary file 3 — Supplementary Data 1 [file 41467_2022_34656_MOESM3_ESM.zip › Supplementary data_1/Supplementary_material_1-1 Geometric morphometrics/bgPCA_306/bgPCA_306_ruminants.pdf]

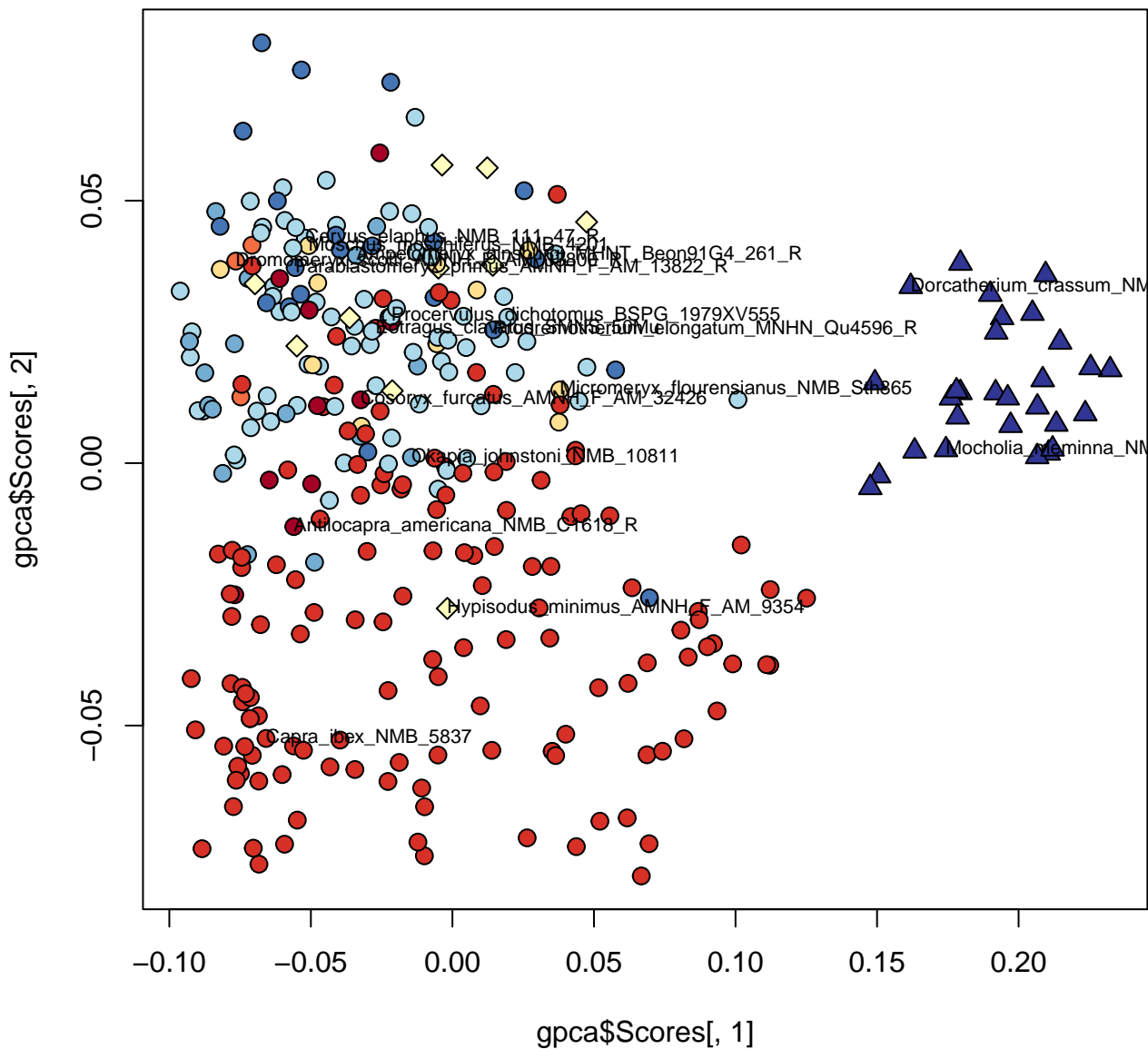

Supplement: Supplementary file 3 — Supplementary Data 1 [file 41467_2022_34656_MOESM3_ESM.zip › Supplementary data_1/Supplementary_material_1-1 Geometric morphometrics/bgPCA_306/bgPCA_306_ruminants_names.pdf]

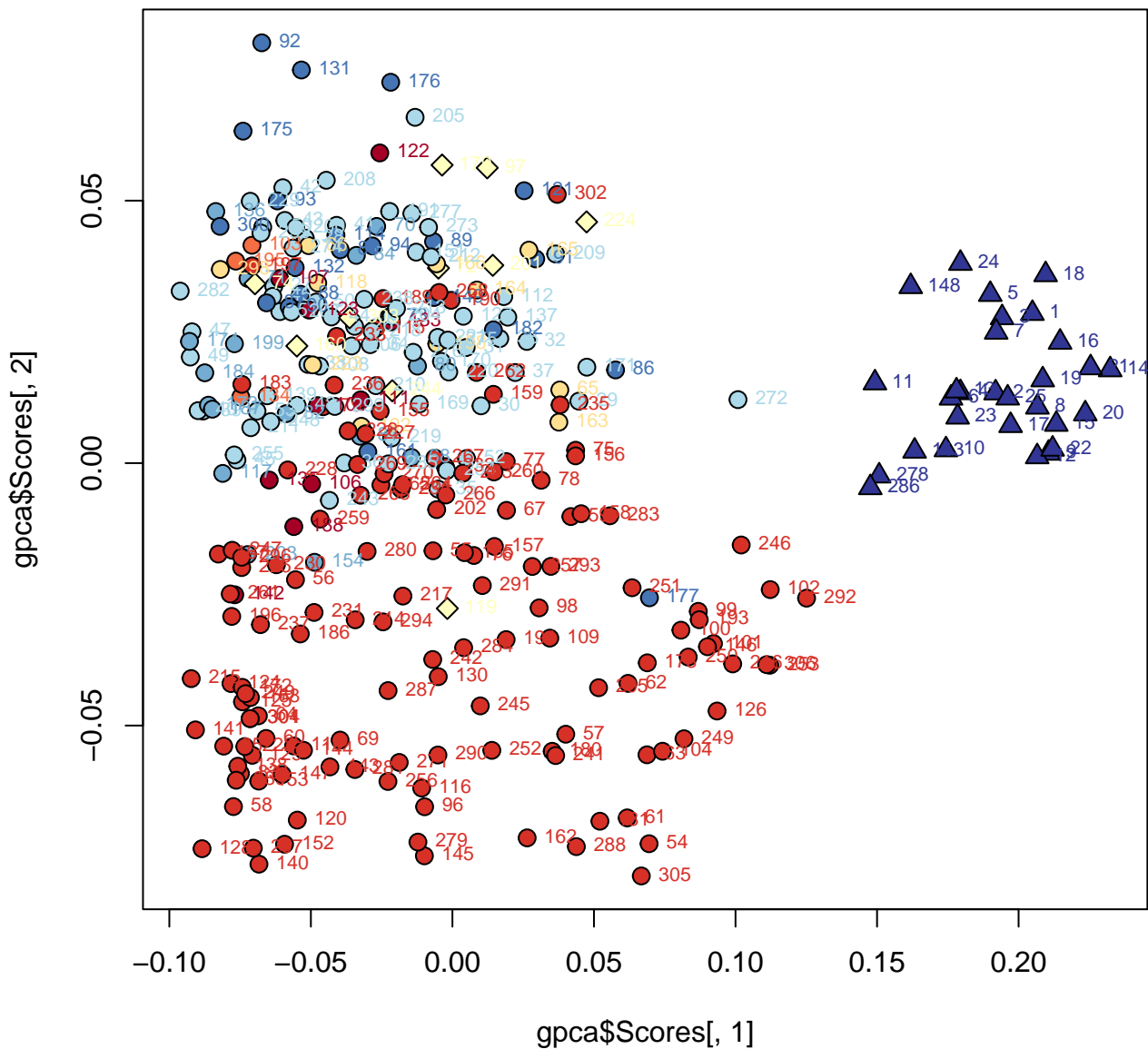

Supplement: Supplementary file 3 — Supplementary Data 1 [file 41467_2022_34656_MOESM3_ESM.zip › Supplementary data_1/Supplementary_material_1-1 Geometric morphometrics/bgPCA_306/bgPCA_306_ruminants_numbers.pdf]

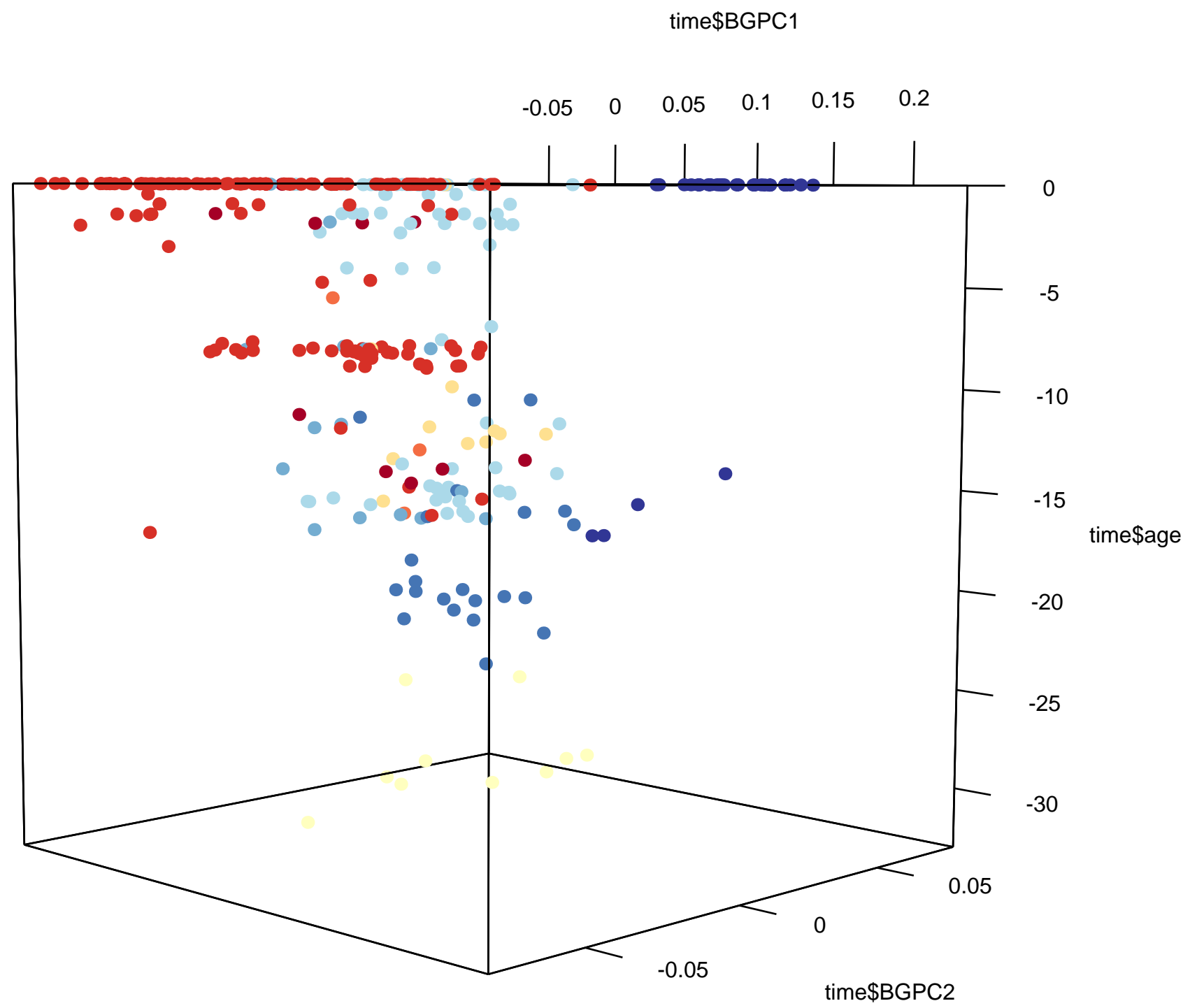

Supplement: Supplementary file 3 — Supplementary Data 1 [file 41467_2022_34656_MOESM3_ESM.zip › Supplementary data_1/Supplementary_material_1-1 Geometric morphometrics/bgPCA_306/BGPCA_3D.pdf]

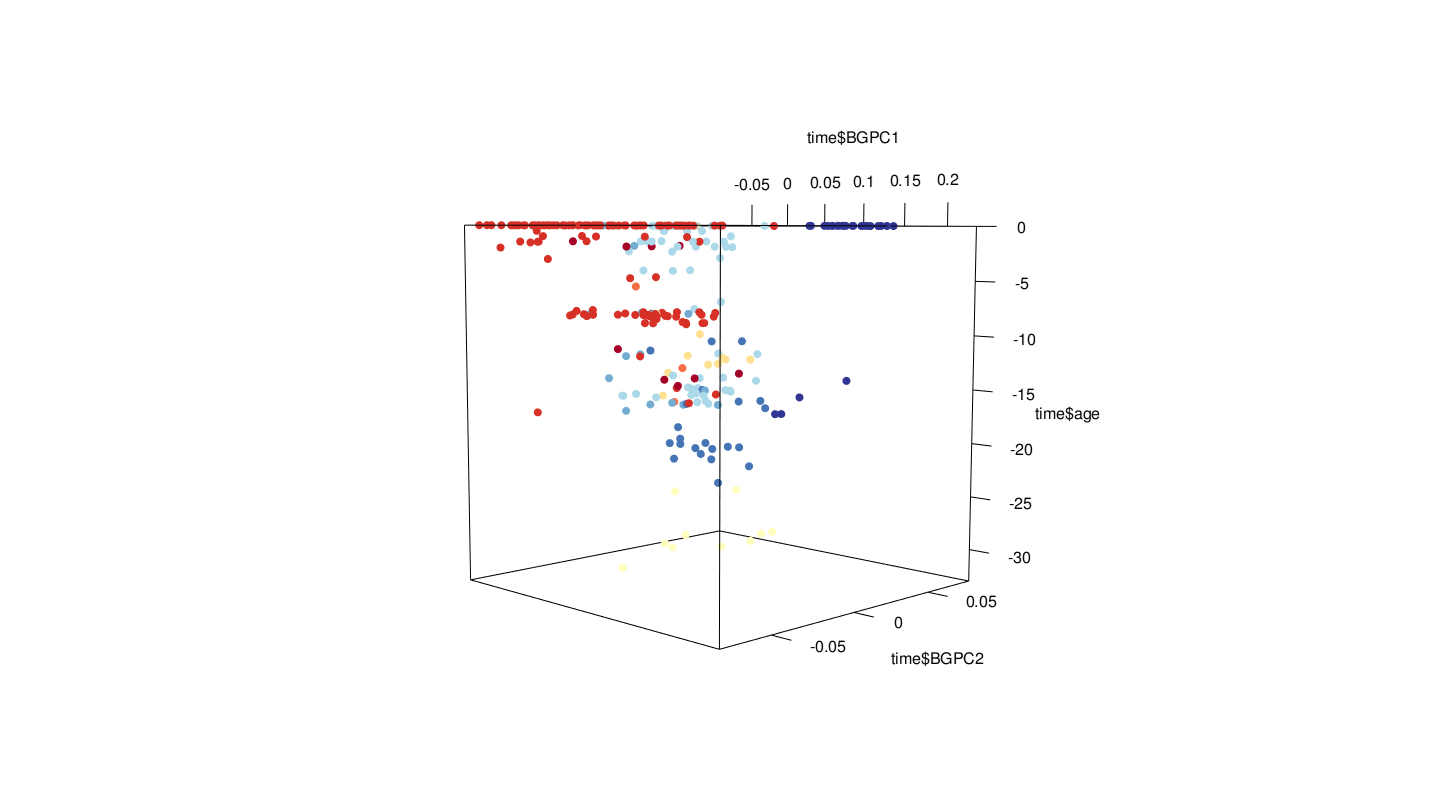

Supplement: Supplementary file 3 — Supplementary Data 1 [file 41467_2022_34656_MOESM3_ESM.zip › Supplementary data_1/Supplementary_material_1-1 Geometric morphometrics/bgPCA_306/BGPCA_3D.png]

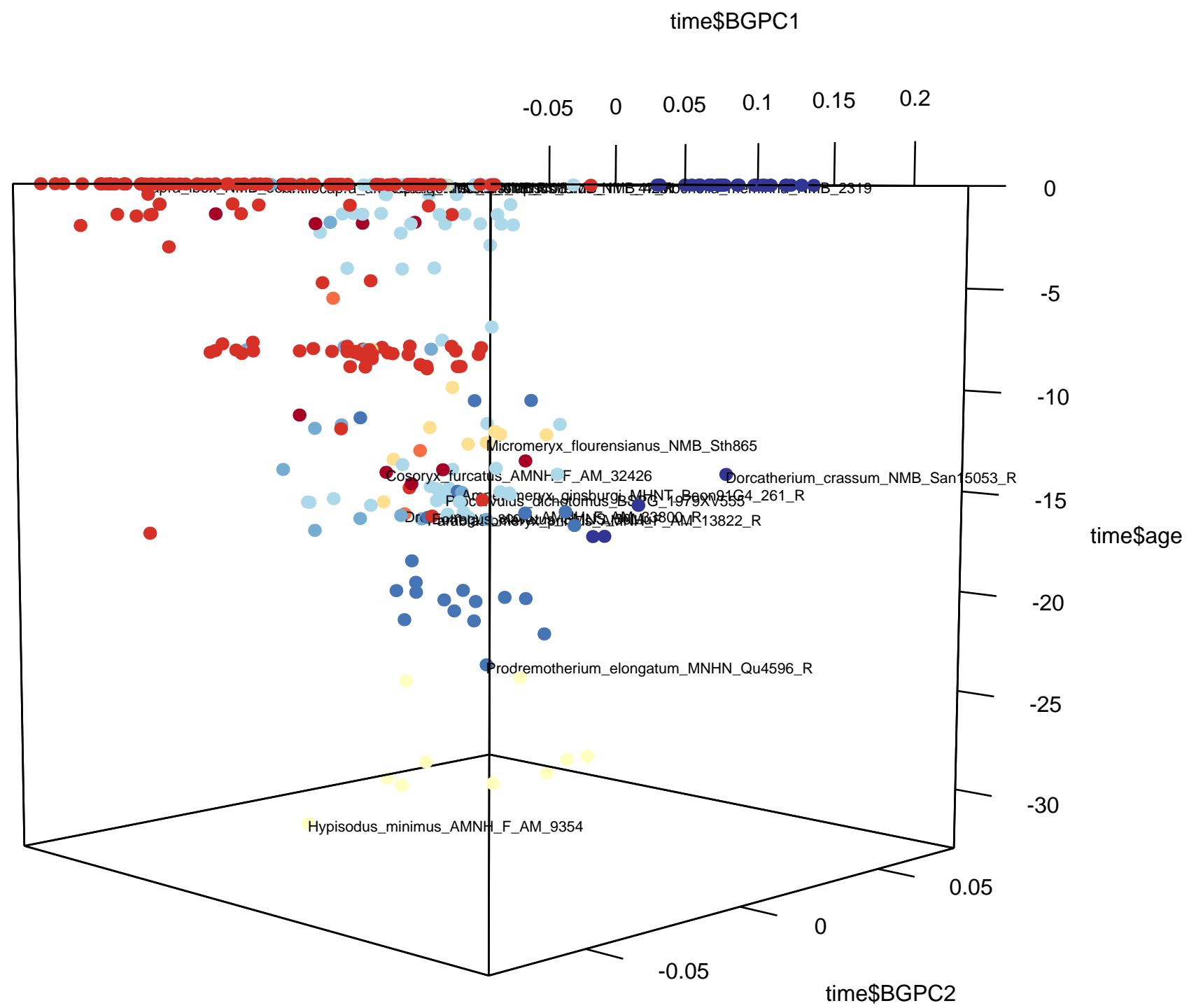

Supplement: Supplementary file 3 — Supplementary Data 1 [file 41467_2022_34656_MOESM3_ESM.zip › Supplementary data_1/Supplementary_material_1-1 Geometric morphometrics/bgPCA_306/BGPCA_3D_names.pdf]

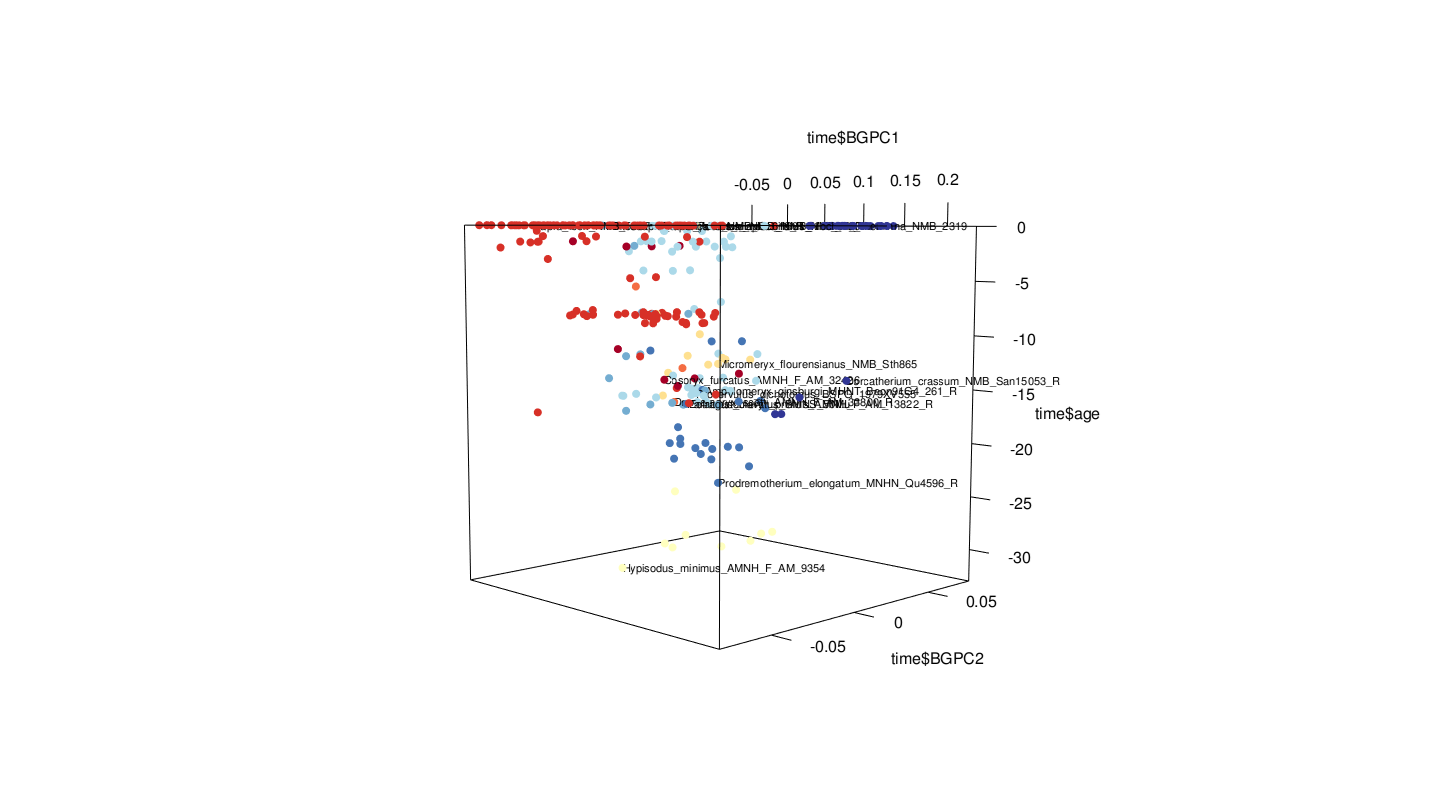

Supplement: Supplementary file 3 — Supplementary Data 1 [file 41467_2022_34656_MOESM3_ESM.zip › Supplementary data_1/Supplementary_material_1-1 Geometric morphometrics/bgPCA_306/BGPCA_3D_names.png]

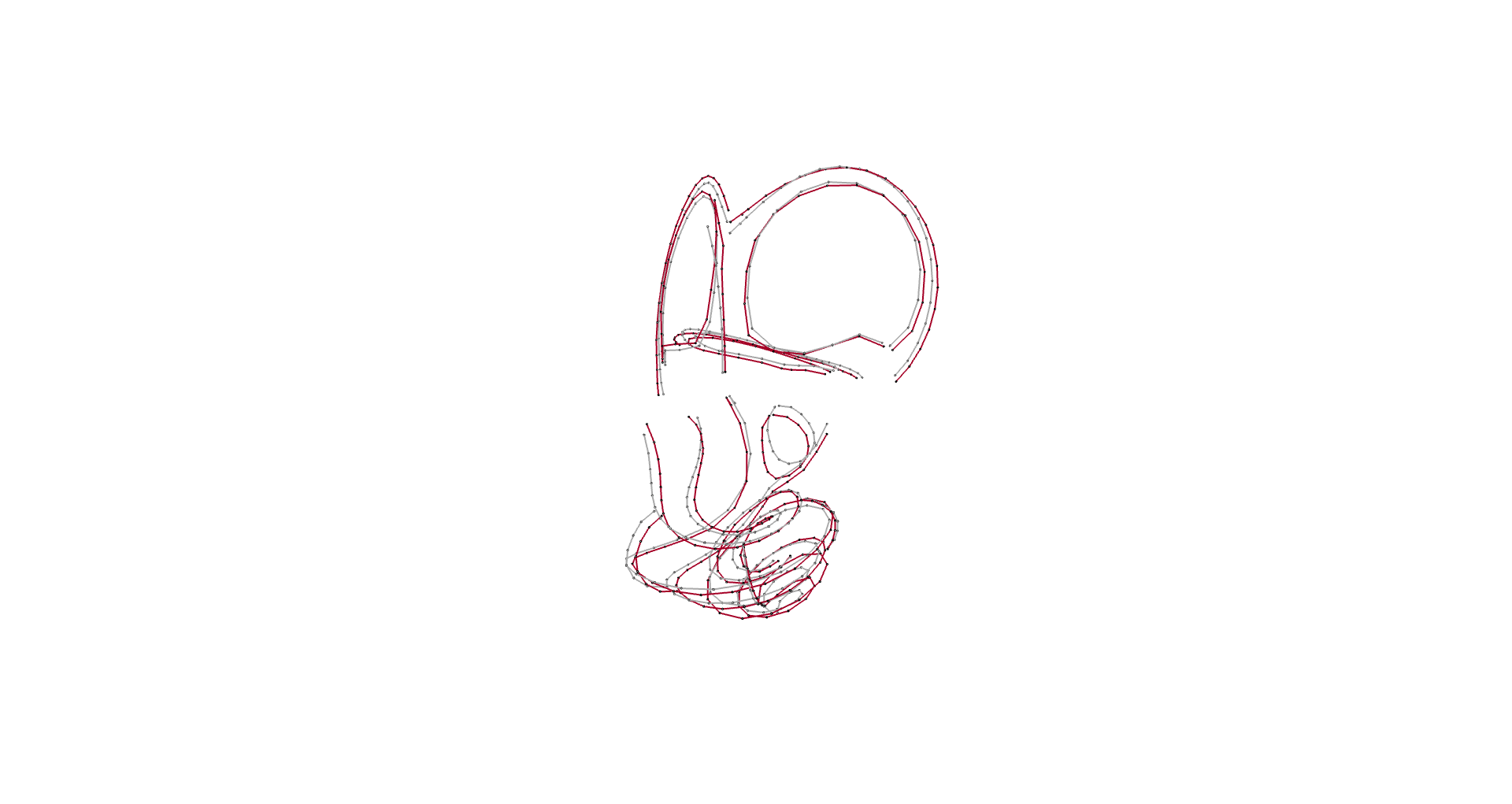

Supplement: Supplementary file 3 — Supplementary Data 1 [file 41467_2022_34656_MOESM3_ESM.zip › Supplementary data_1/Supplementary_material_1-1 Geometric morphometrics/bgPCA_306/mean_shapes_per_clade_bgPCA/Antilo-dl.png]

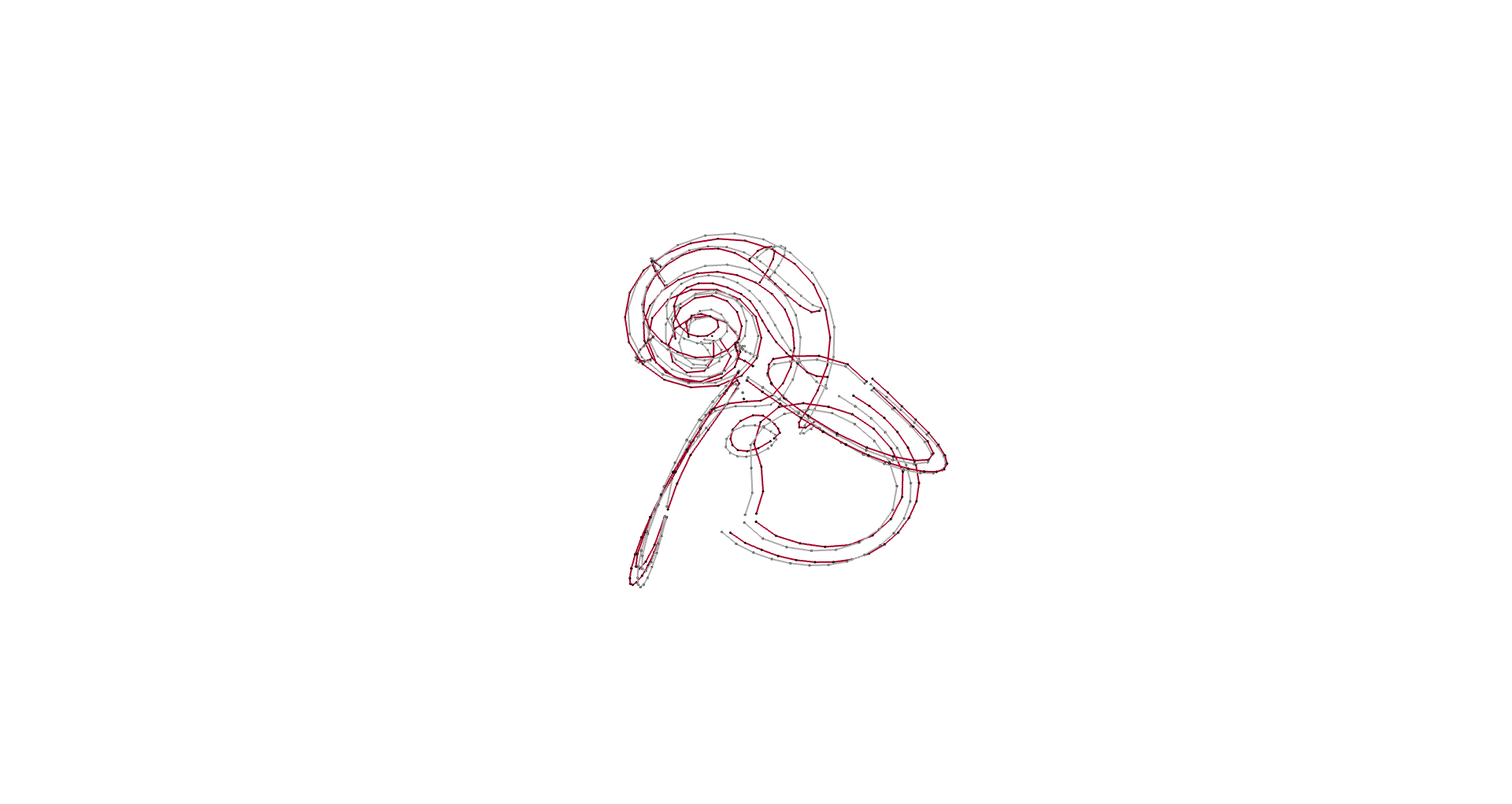

Supplement: Supplementary file 3 — Supplementary Data 1 [file 41467_2022_34656_MOESM3_ESM.zip › Supplementary data_1/Supplementary_material_1-1 Geometric morphometrics/bgPCA_306/mean_shapes_per_clade_bgPCA/Antilo-do.png]

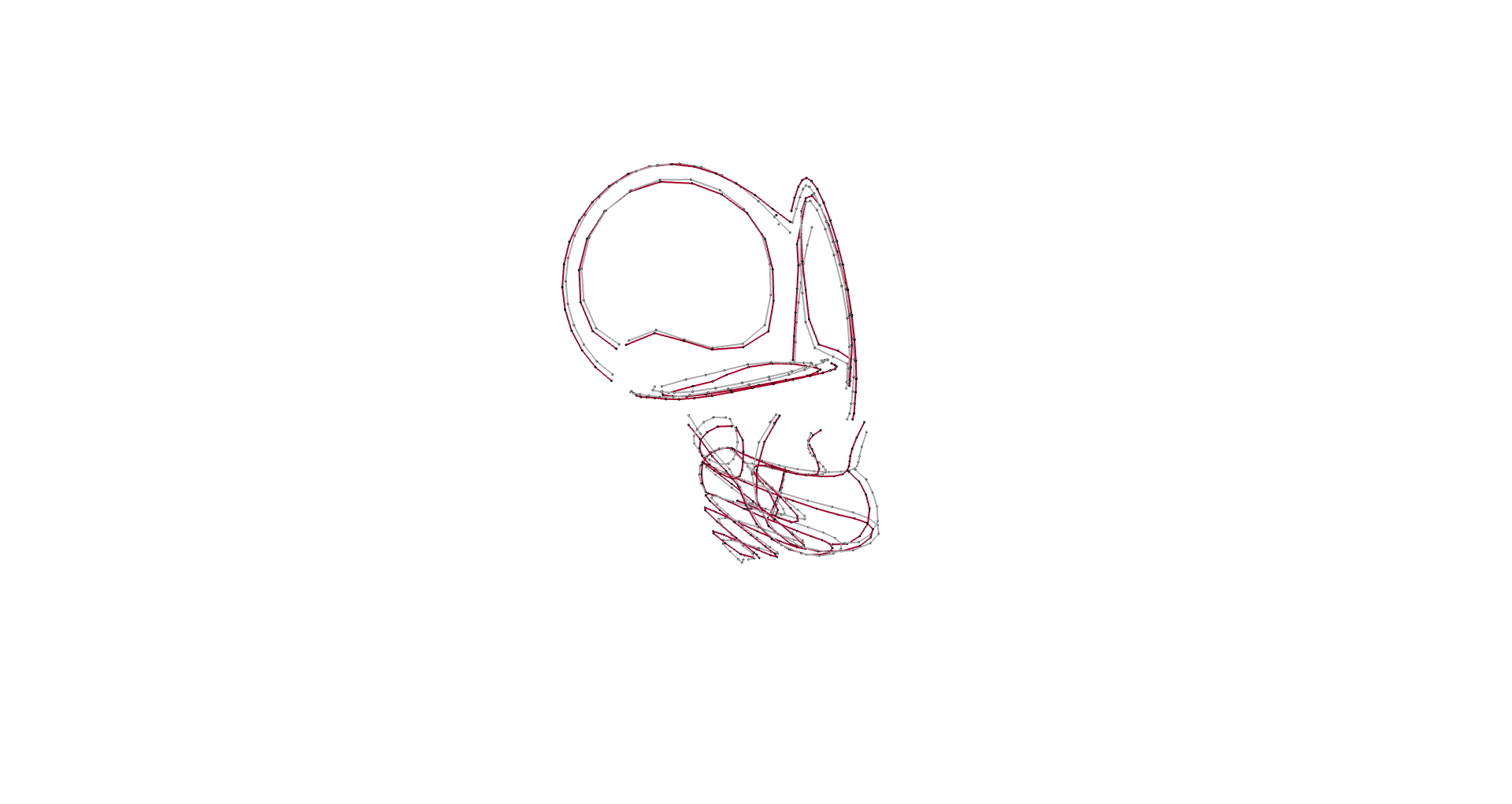

Supplement: Supplementary file 3 — Supplementary Data 1 [file 41467_2022_34656_MOESM3_ESM.zip › Supplementary data_1/Supplementary_material_1-1 Geometric morphometrics/bgPCA_306/mean_shapes_per_clade_bgPCA/Antilo-la.png]

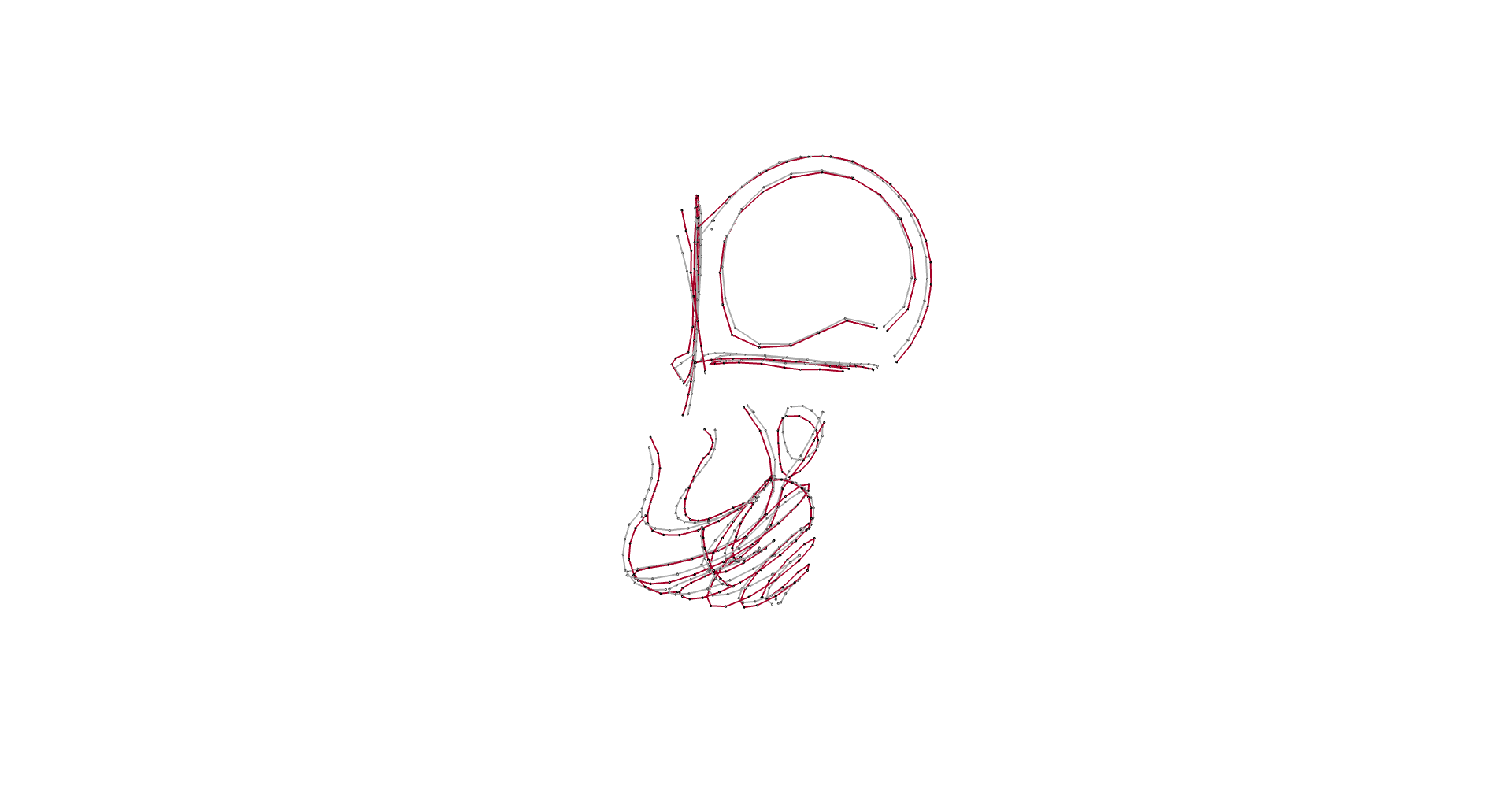

Supplement: Supplementary file 3 — Supplementary Data 1 [file 41467_2022_34656_MOESM3_ESM.zip › Supplementary data_1/Supplementary_material_1-1 Geometric morphometrics/bgPCA_306/mean_shapes_per_clade_bgPCA/Antilo-me.png]

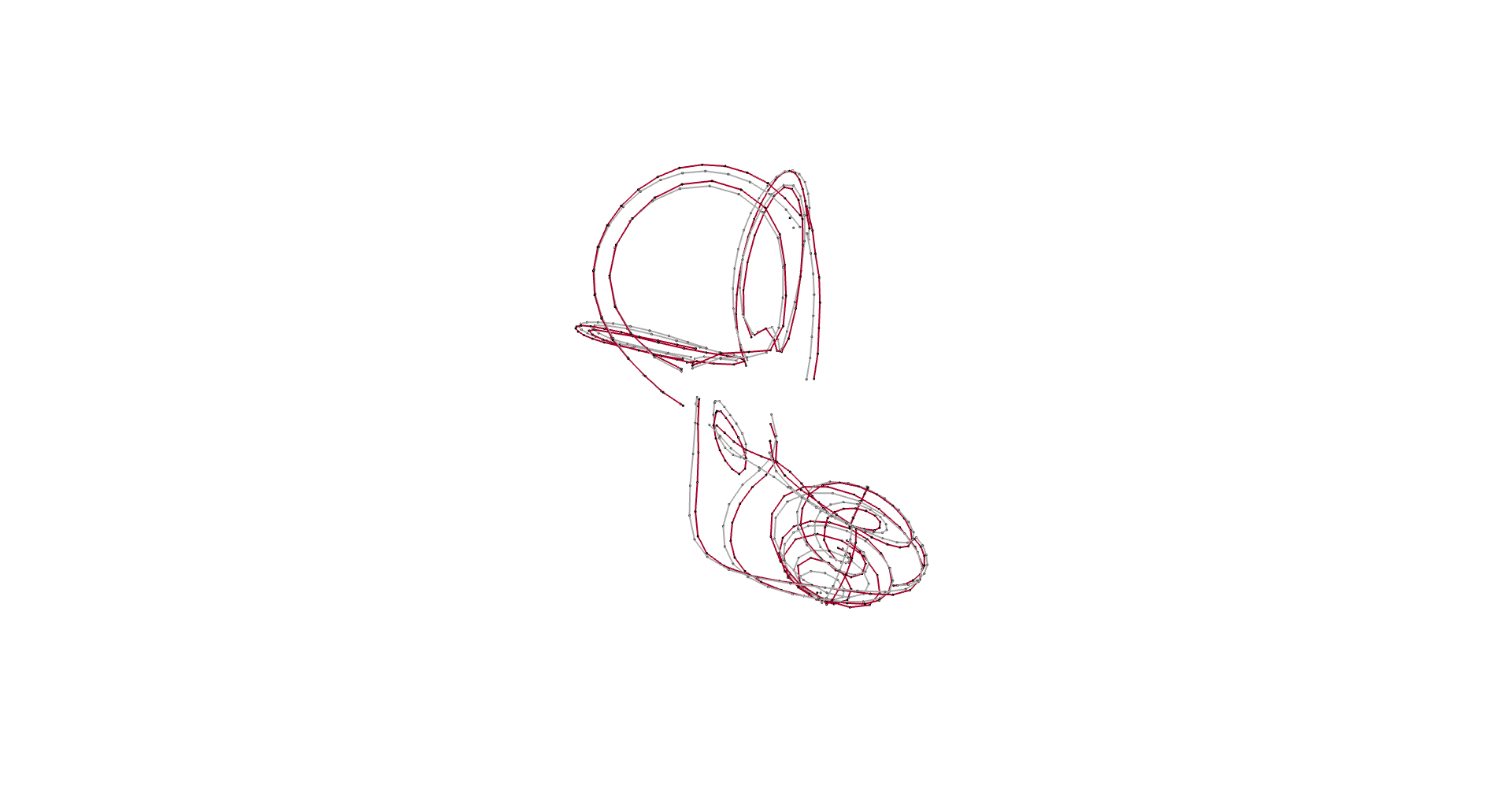

Supplement: Supplementary file 3 — Supplementary Data 1 [file 41467_2022_34656_MOESM3_ESM.zip › Supplementary data_1/Supplementary_material_1-1 Geometric morphometrics/bgPCA_306/mean_shapes_per_clade_bgPCA/Antilo-oc.png]

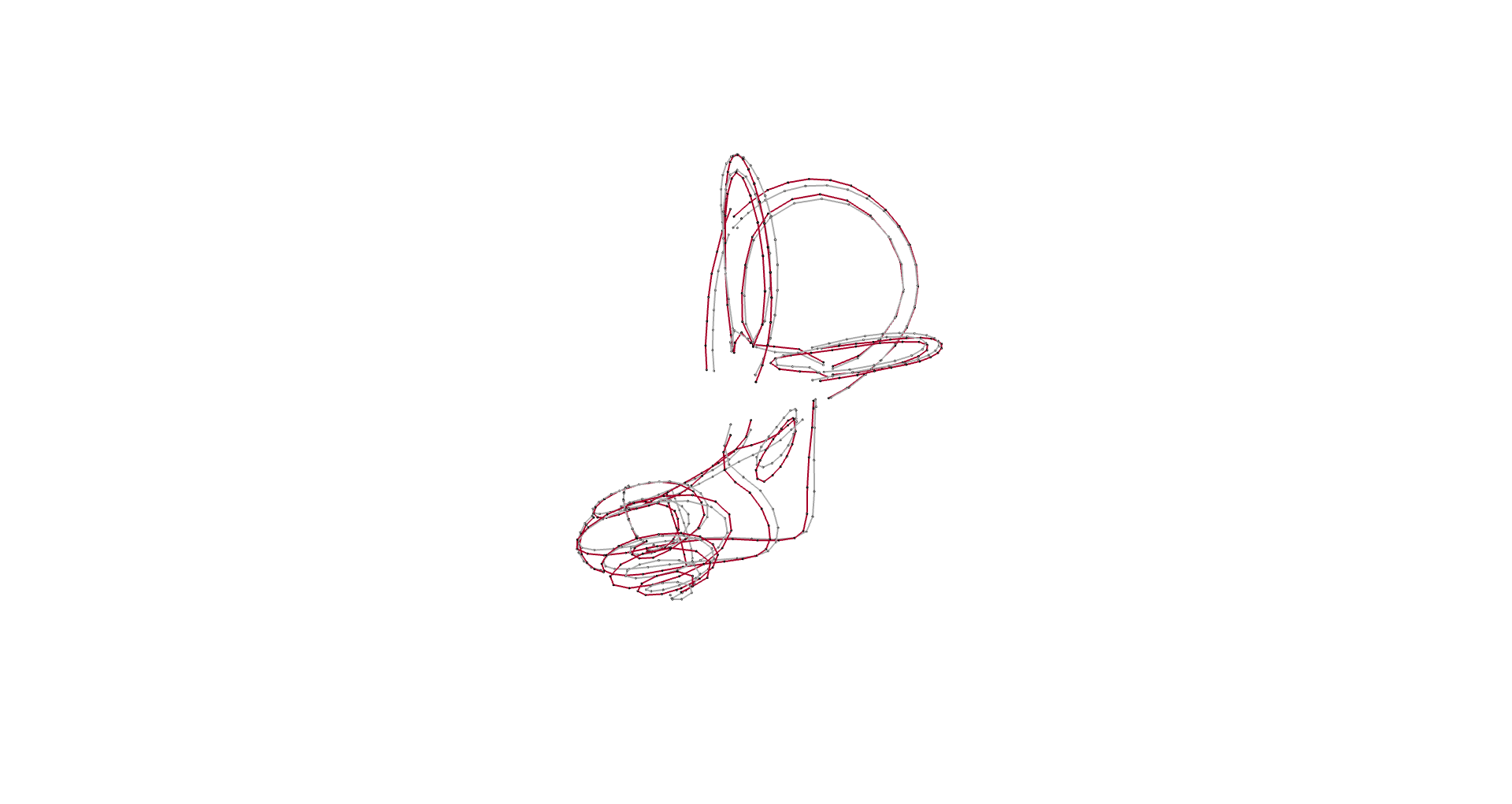

Supplement: Supplementary file 3 — Supplementary Data 1 [file 41467_2022_34656_MOESM3_ESM.zip › Supplementary data_1/Supplementary_material_1-1 Geometric morphometrics/bgPCA_306/mean_shapes_per_clade_bgPCA/Antilo-ro.png]

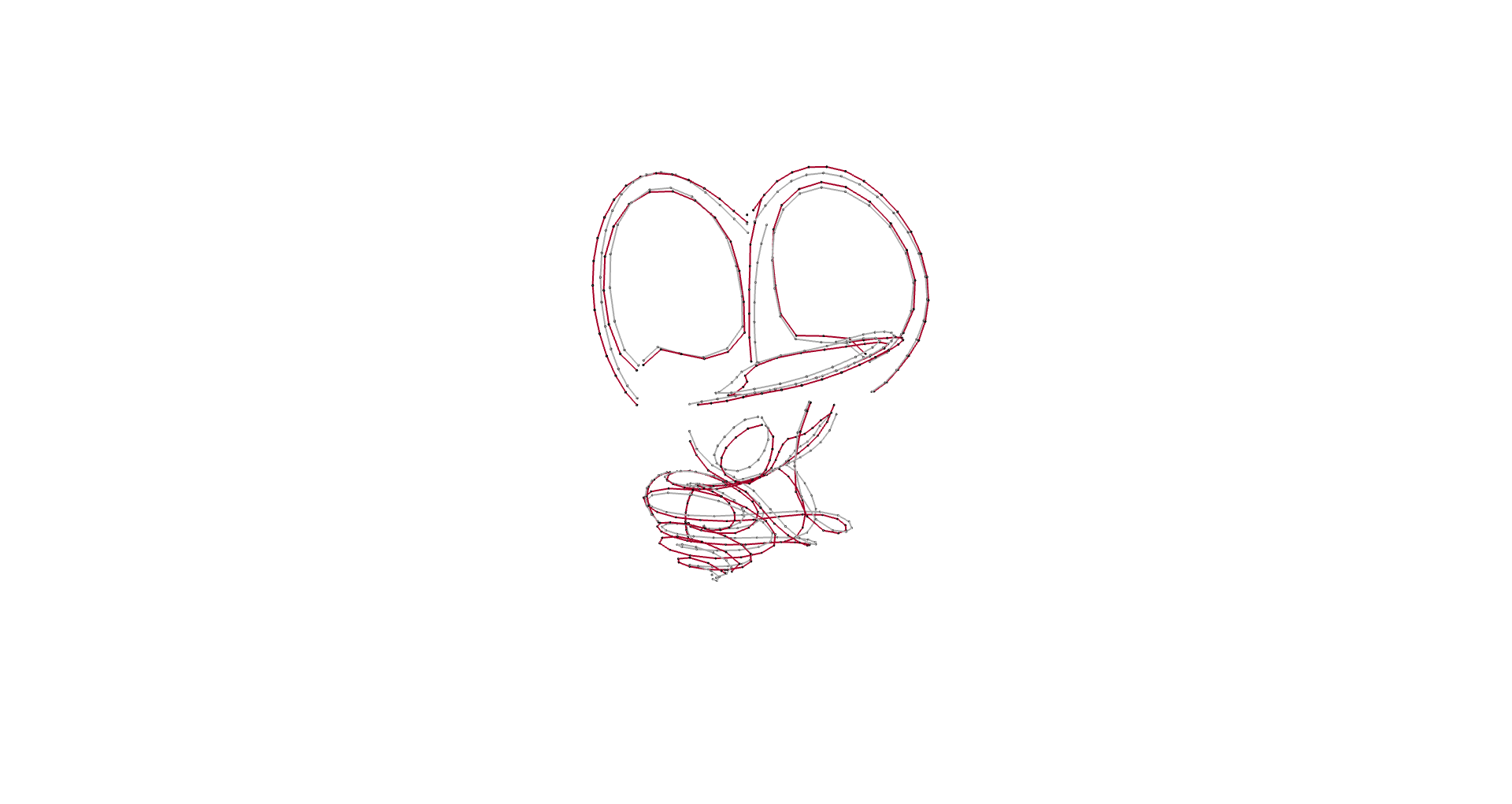

Supplement: Supplementary file 3 — Supplementary Data 1 [file 41467_2022_34656_MOESM3_ESM.zip › Supplementary data_1/Supplementary_material_1-1 Geometric morphometrics/bgPCA_306/mean_shapes_per_clade_bgPCA/Antilo-vl.png]

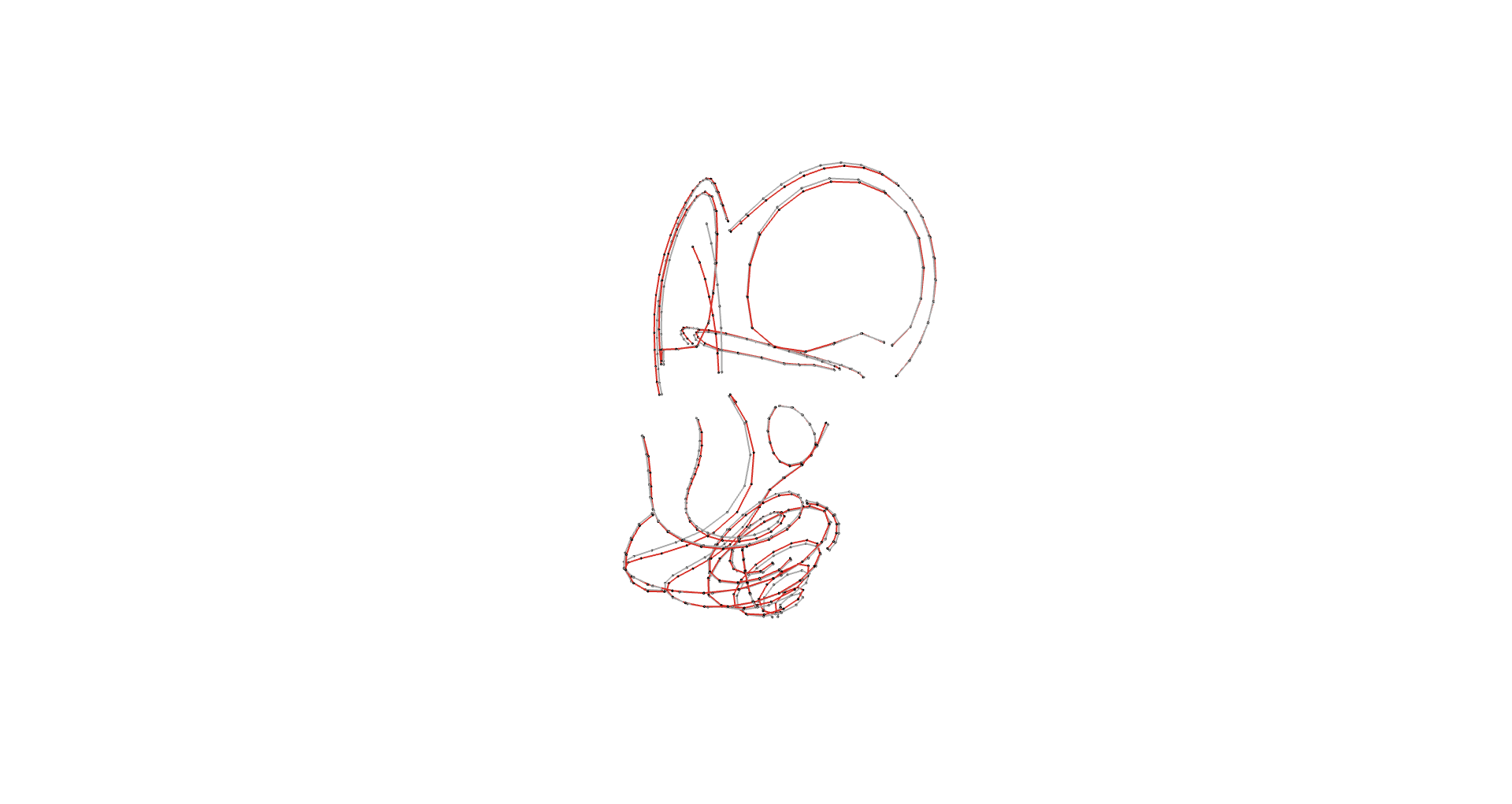

Supplement: Supplementary file 3 — Supplementary Data 1 [file 41467_2022_34656_MOESM3_ESM.zip › Supplementary data_1/Supplementary_material_1-1 Geometric morphometrics/bgPCA_306/mean_shapes_per_clade_bgPCA/Bovidae-dl.png]

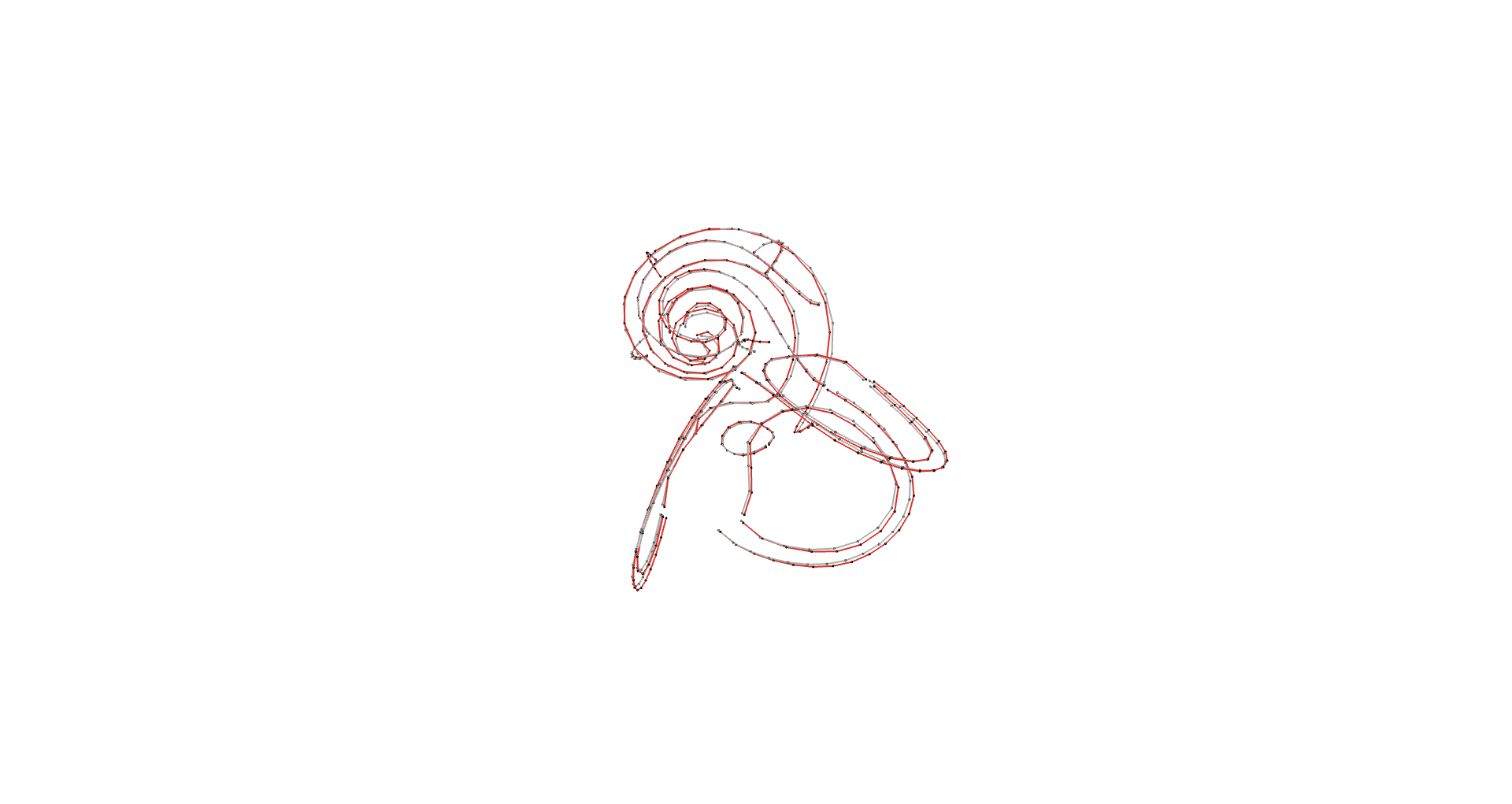

Supplement: Supplementary file 3 — Supplementary Data 1 [file 41467_2022_34656_MOESM3_ESM.zip › Supplementary data_1/Supplementary_material_1-1 Geometric morphometrics/bgPCA_306/mean_shapes_per_clade_bgPCA/Bovidae-do.png]

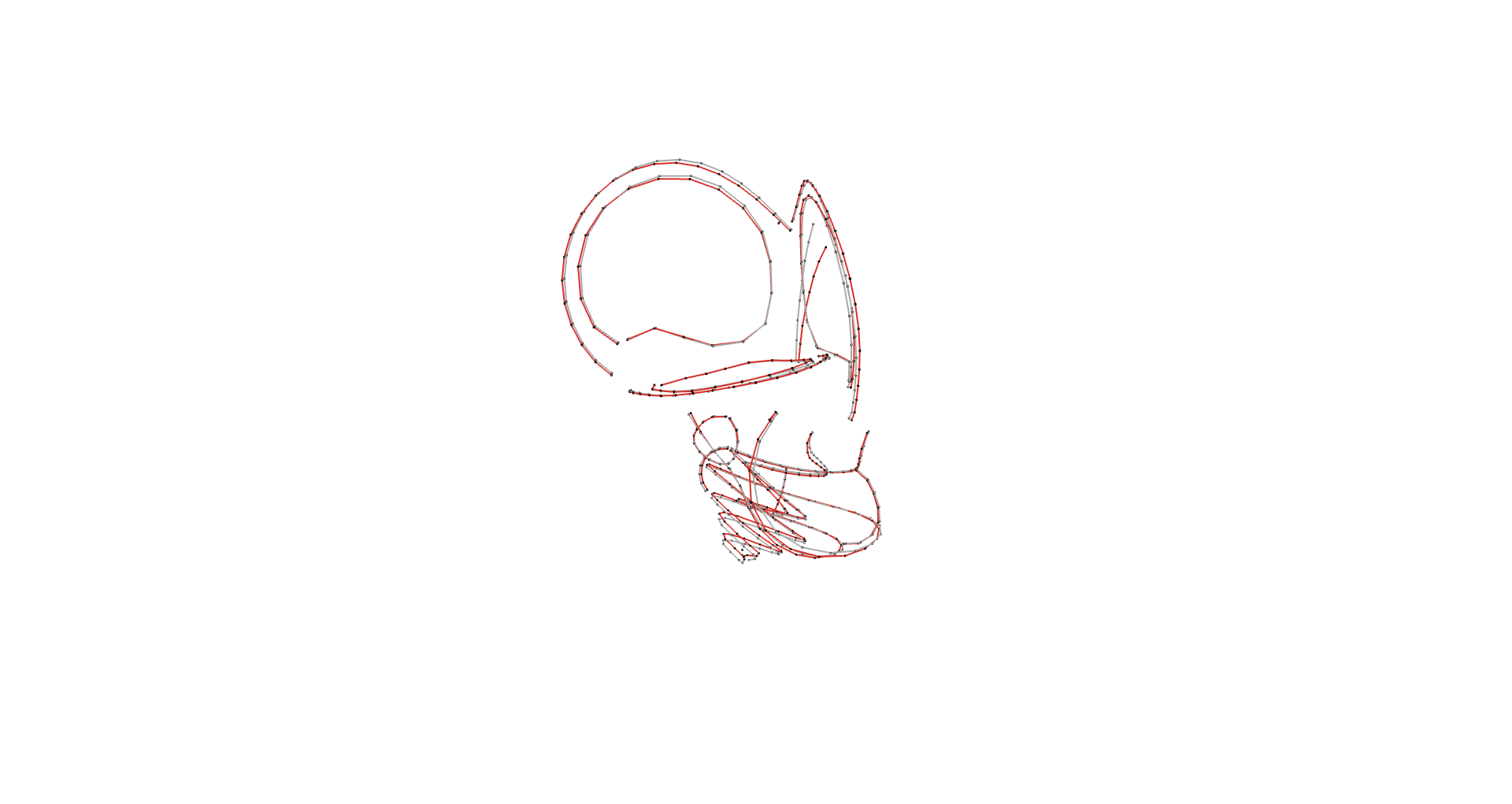

Supplement: Supplementary file 3 — Supplementary Data 1 [file 41467_2022_34656_MOESM3_ESM.zip › Supplementary data_1/Supplementary_material_1-1 Geometric morphometrics/bgPCA_306/mean_shapes_per_clade_bgPCA/Bovidae-la.png]

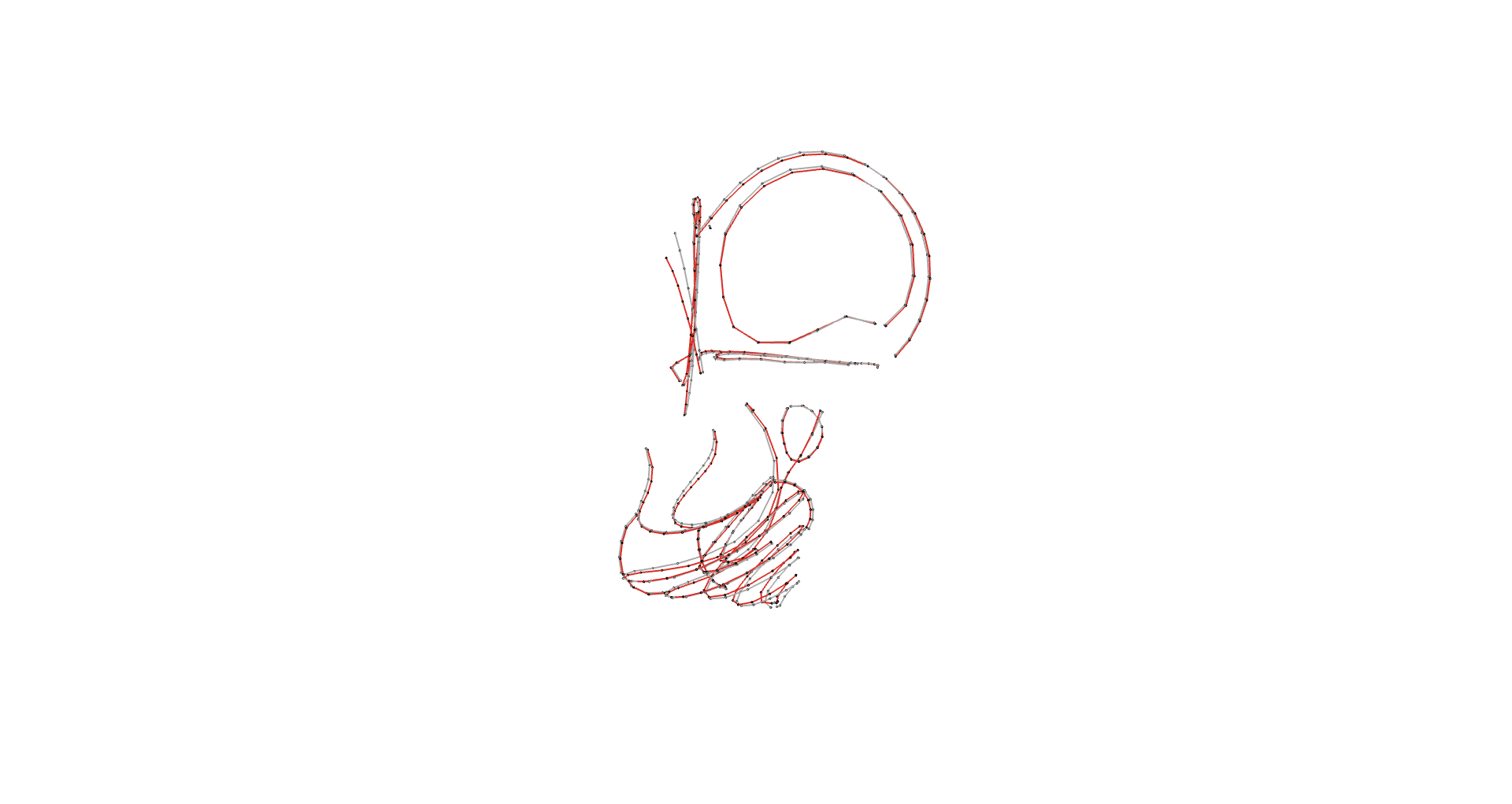

Supplement: Supplementary file 3 — Supplementary Data 1 [file 41467_2022_34656_MOESM3_ESM.zip › Supplementary data_1/Supplementary_material_1-1 Geometric morphometrics/bgPCA_306/mean_shapes_per_clade_bgPCA/Bovidae-me.png]

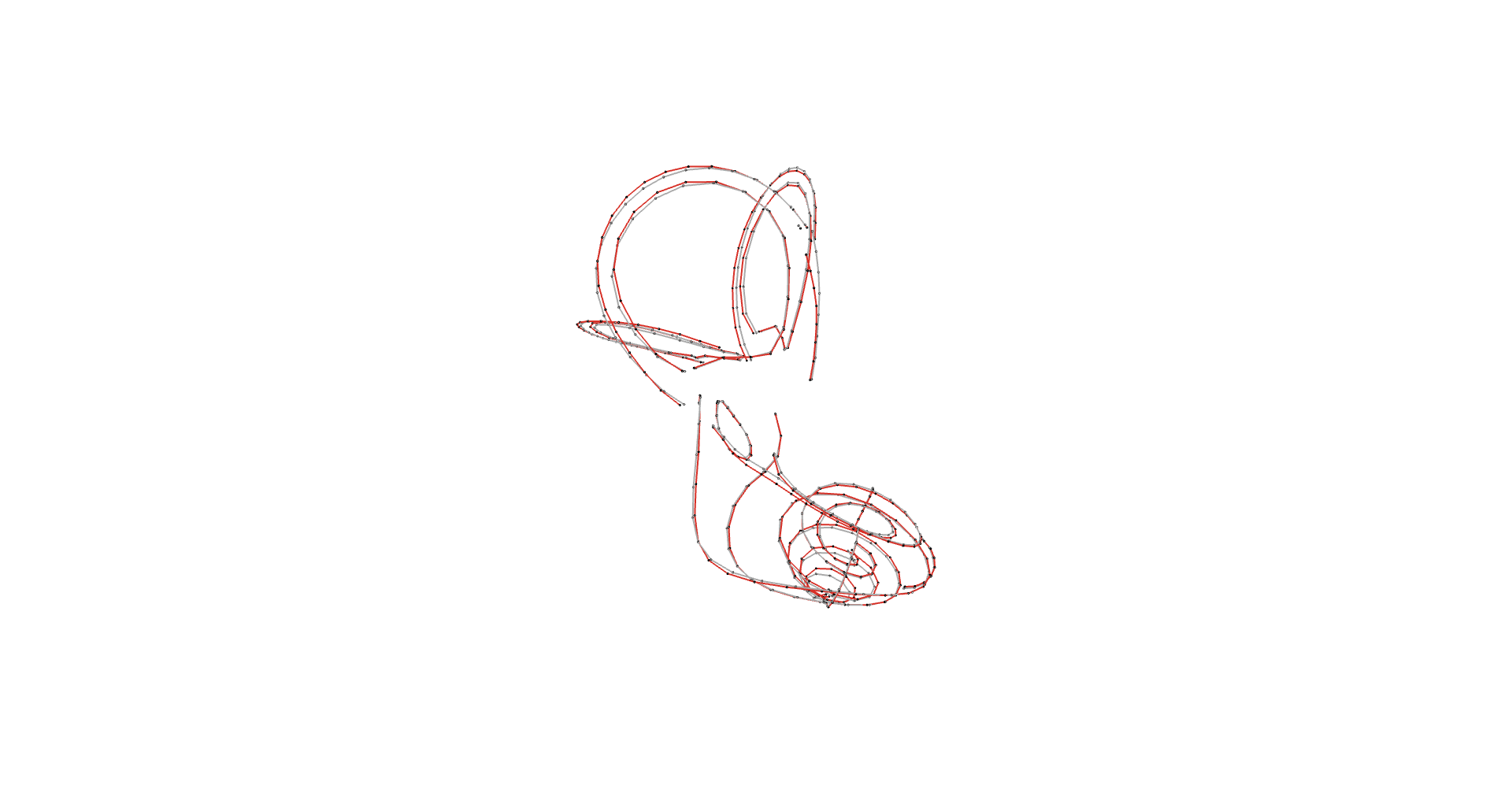

Supplement: Supplementary file 3 — Supplementary Data 1 [file 41467_2022_34656_MOESM3_ESM.zip › Supplementary data_1/Supplementary_material_1-1 Geometric morphometrics/bgPCA_306/mean_shapes_per_clade_bgPCA/Bovidae-oc.png]

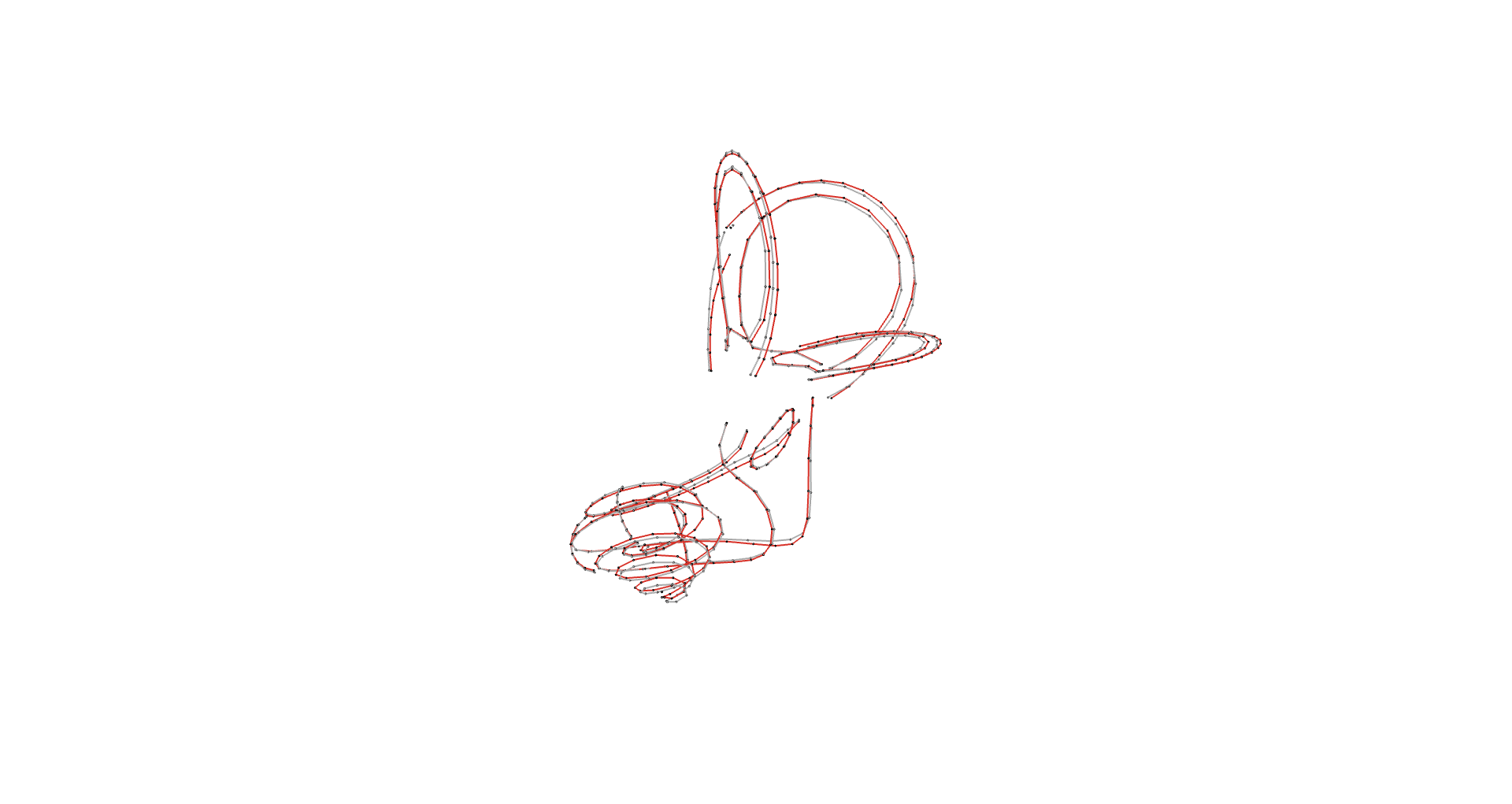

Supplement: Supplementary file 3 — Supplementary Data 1 [file 41467_2022_34656_MOESM3_ESM.zip › Supplementary data_1/Supplementary_material_1-1 Geometric morphometrics/bgPCA_306/mean_shapes_per_clade_bgPCA/Bovidae-ro.png]

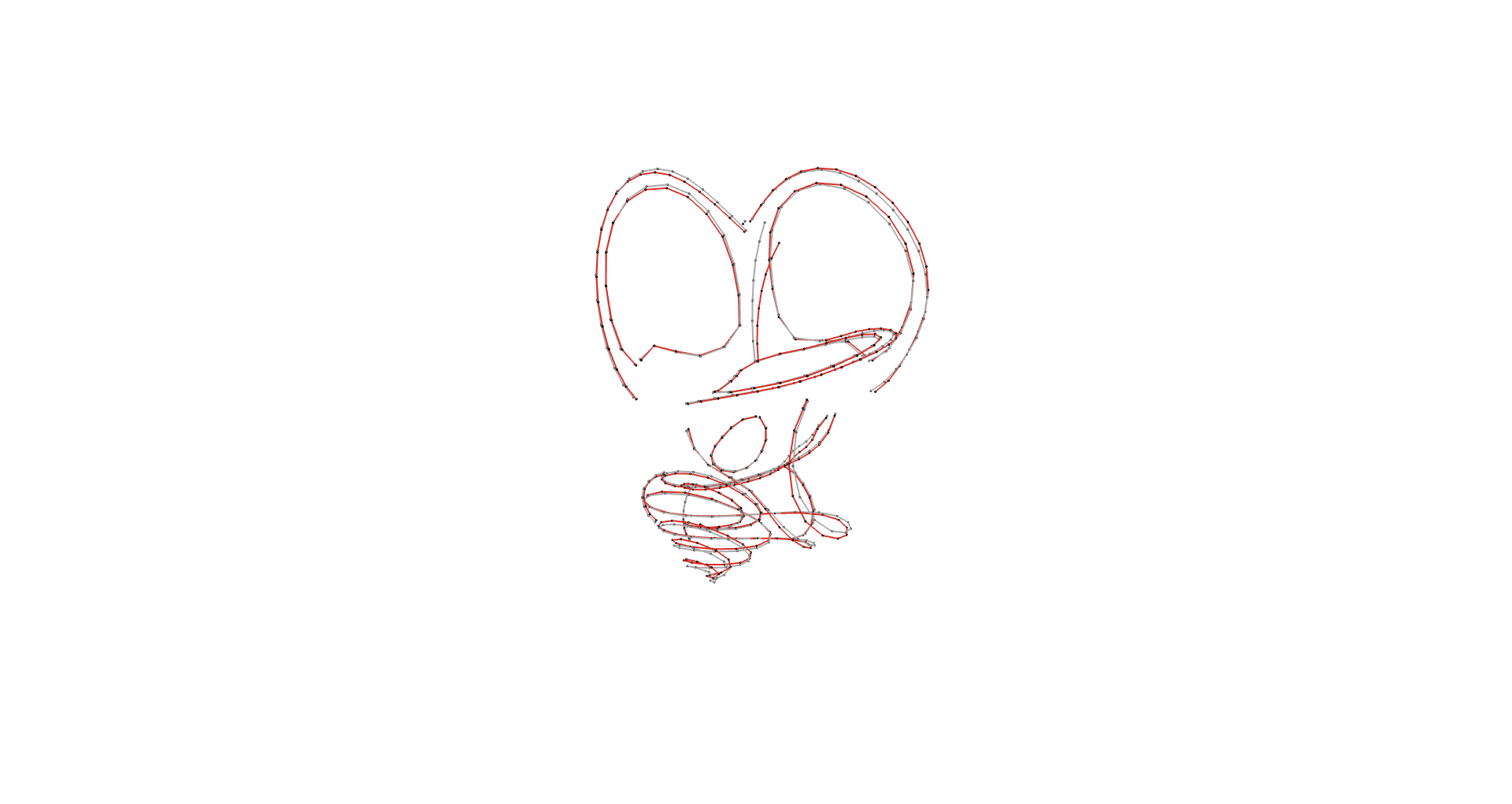

Supplement: Supplementary file 3 — Supplementary Data 1 [file 41467_2022_34656_MOESM3_ESM.zip › Supplementary data_1/Supplementary_material_1-1 Geometric morphometrics/bgPCA_306/mean_shapes_per_clade_bgPCA/Bovidae-vl.png]

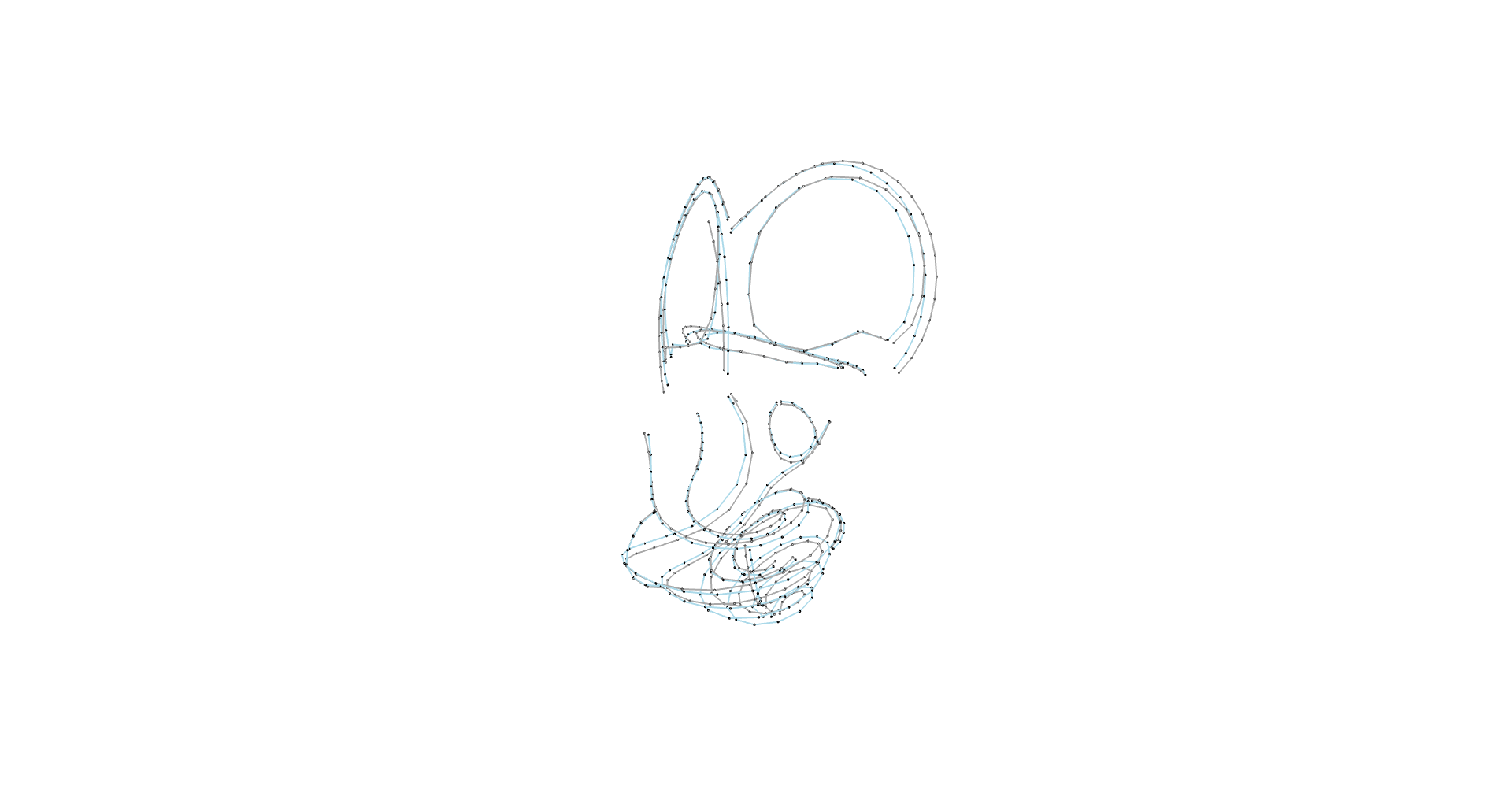

Supplement: Supplementary file 3 — Supplementary Data 1 [file 41467_2022_34656_MOESM3_ESM.zip › Supplementary data_1/Supplementary_material_1-1 Geometric morphometrics/bgPCA_306/mean_shapes_per_clade_bgPCA/Cervidae-dl.png]

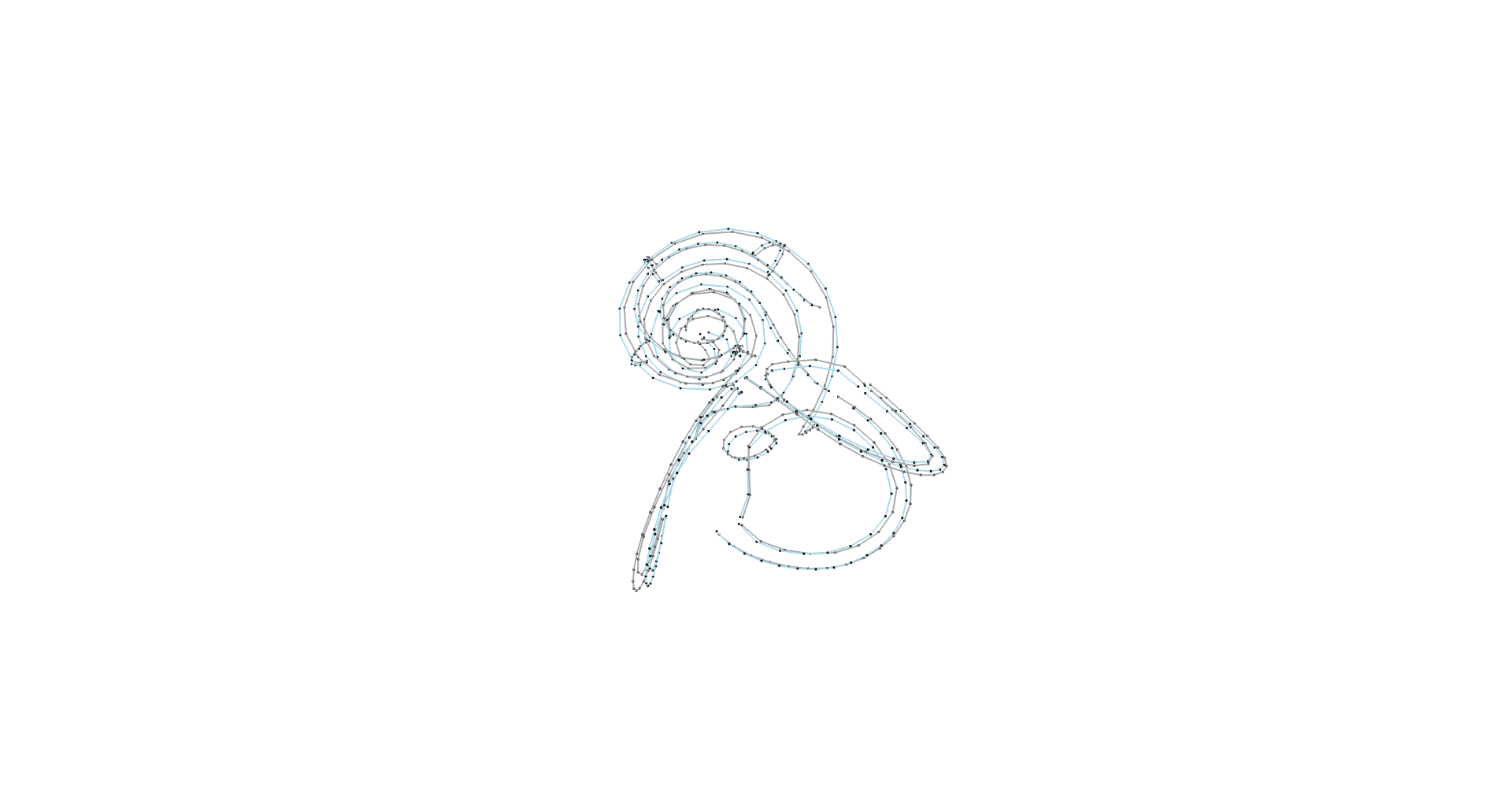

Supplement: Supplementary file 3 — Supplementary Data 1 [file 41467_2022_34656_MOESM3_ESM.zip › Supplementary data_1/Supplementary_material_1-1 Geometric morphometrics/bgPCA_306/mean_shapes_per_clade_bgPCA/Cervidae-do.png]

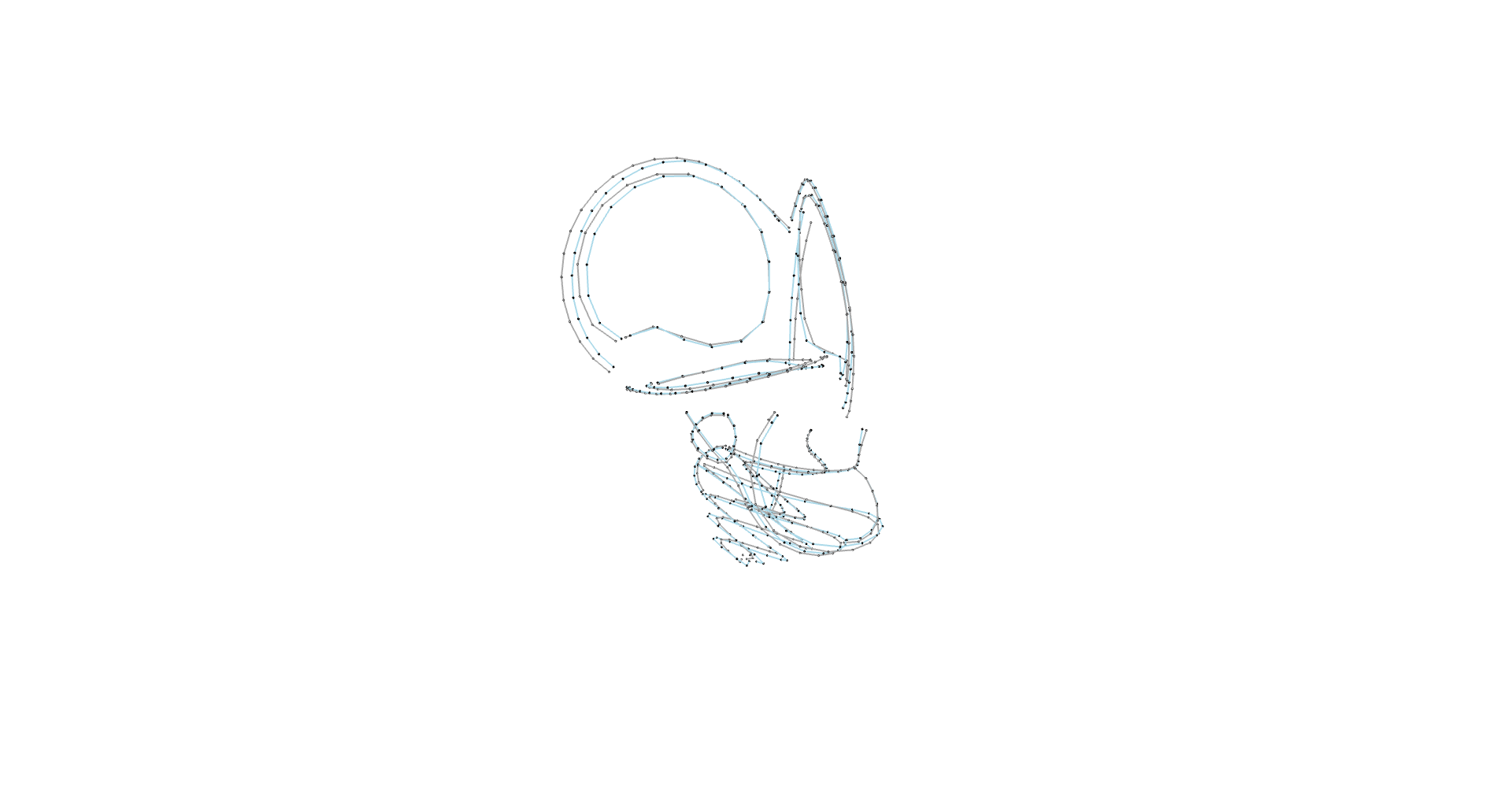

Supplement: Supplementary file 3 — Supplementary Data 1 [file 41467_2022_34656_MOESM3_ESM.zip › Supplementary data_1/Supplementary_material_1-1 Geometric morphometrics/bgPCA_306/mean_shapes_per_clade_bgPCA/Cervidae-la.png]

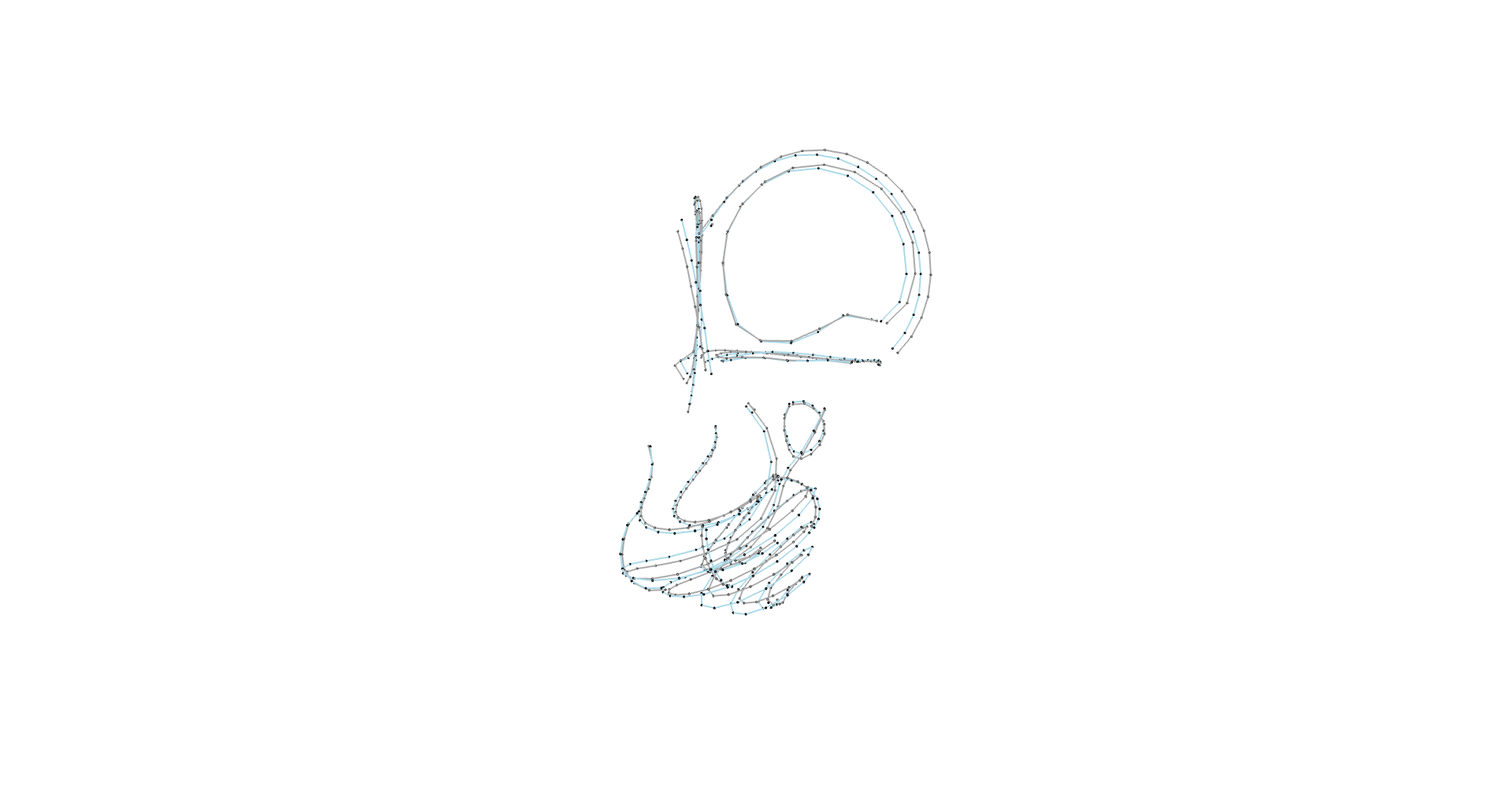

Supplement: Supplementary file 3 — Supplementary Data 1 [file 41467_2022_34656_MOESM3_ESM.zip › Supplementary data_1/Supplementary_material_1-1 Geometric morphometrics/bgPCA_306/mean_shapes_per_clade_bgPCA/Cervidae-me.png]

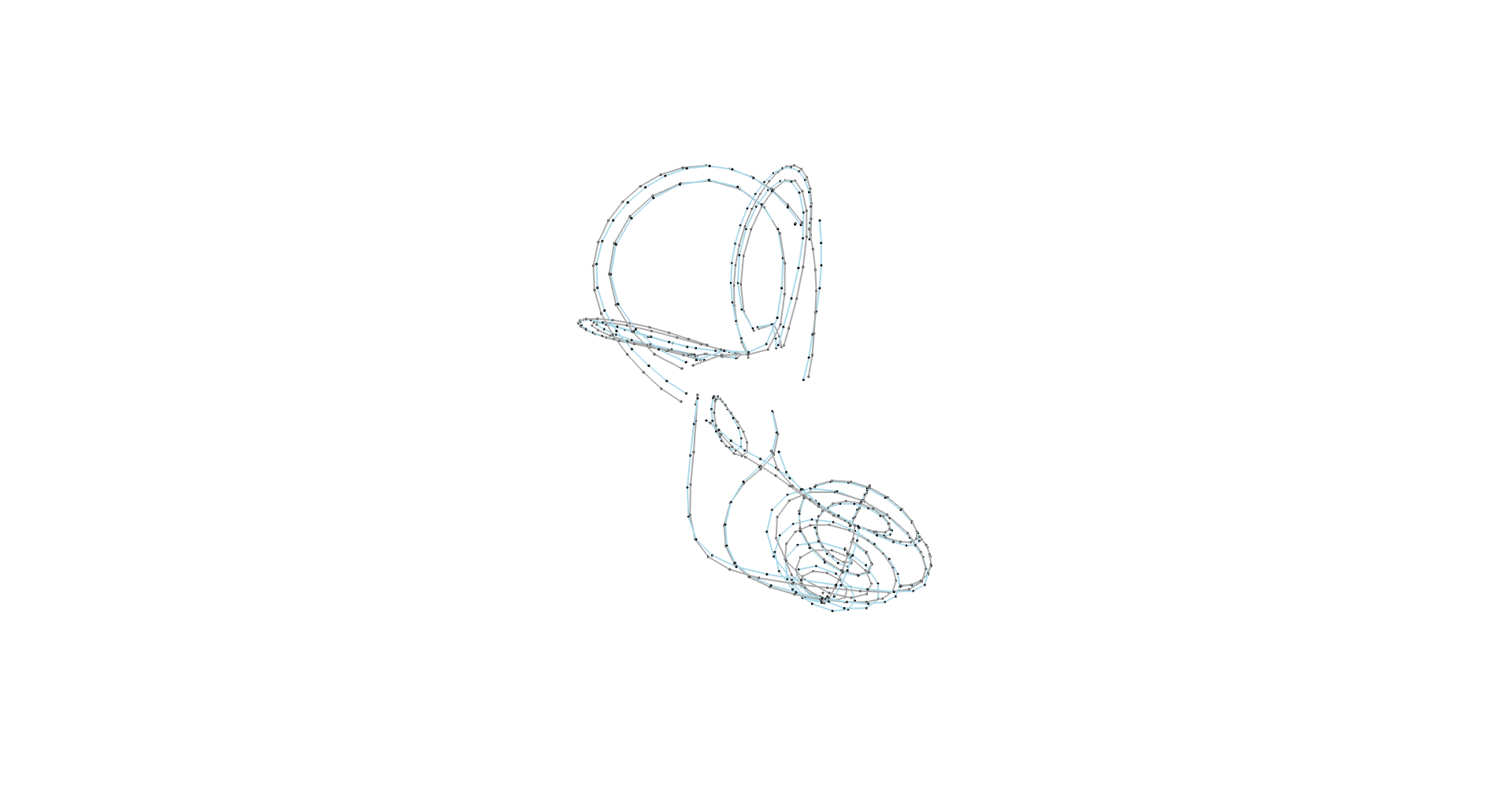

Supplement: Supplementary file 3 — Supplementary Data 1 [file 41467_2022_34656_MOESM3_ESM.zip › Supplementary data_1/Supplementary_material_1-1 Geometric morphometrics/bgPCA_306/mean_shapes_per_clade_bgPCA/Cervidae-oc.png]

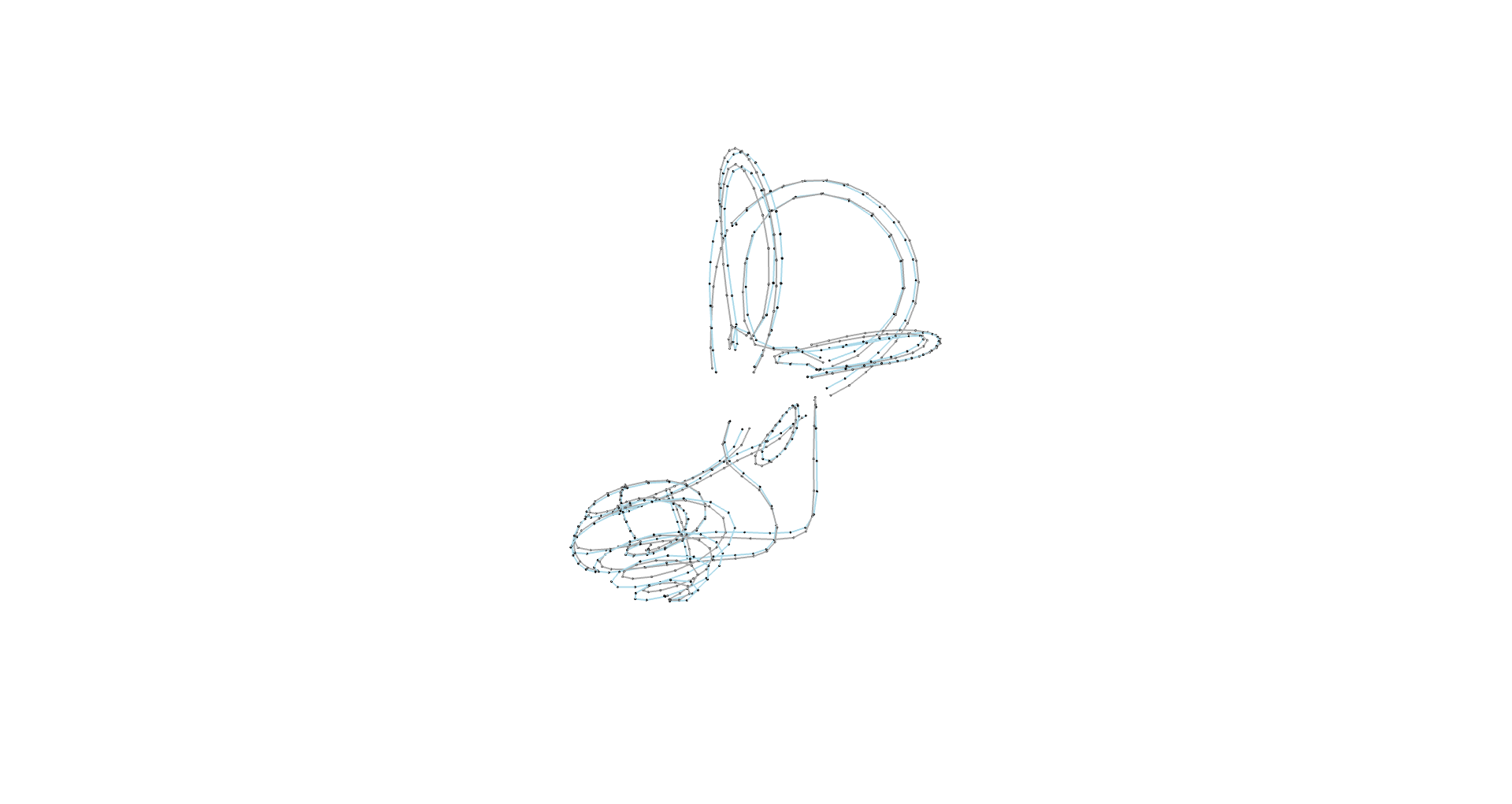

Supplement: Supplementary file 3 — Supplementary Data 1 [file 41467_2022_34656_MOESM3_ESM.zip › Supplementary data_1/Supplementary_material_1-1 Geometric morphometrics/bgPCA_306/mean_shapes_per_clade_bgPCA/Cervidae-ro.png]

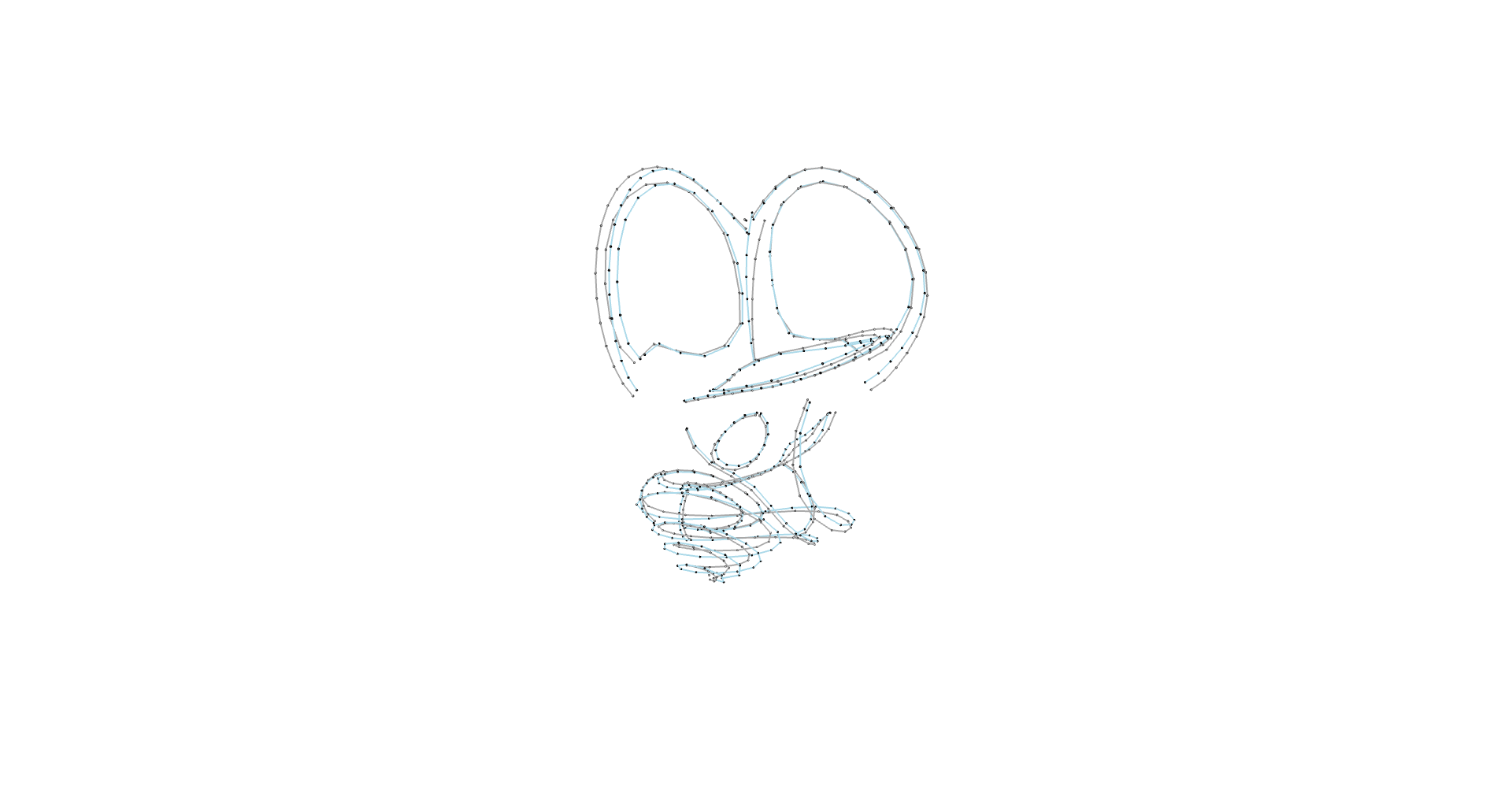

Supplement: Supplementary file 3 — Supplementary Data 1 [file 41467_2022_34656_MOESM3_ESM.zip › Supplementary data_1/Supplementary_material_1-1 Geometric morphometrics/bgPCA_306/mean_shapes_per_clade_bgPCA/Cervidae-vl.png]

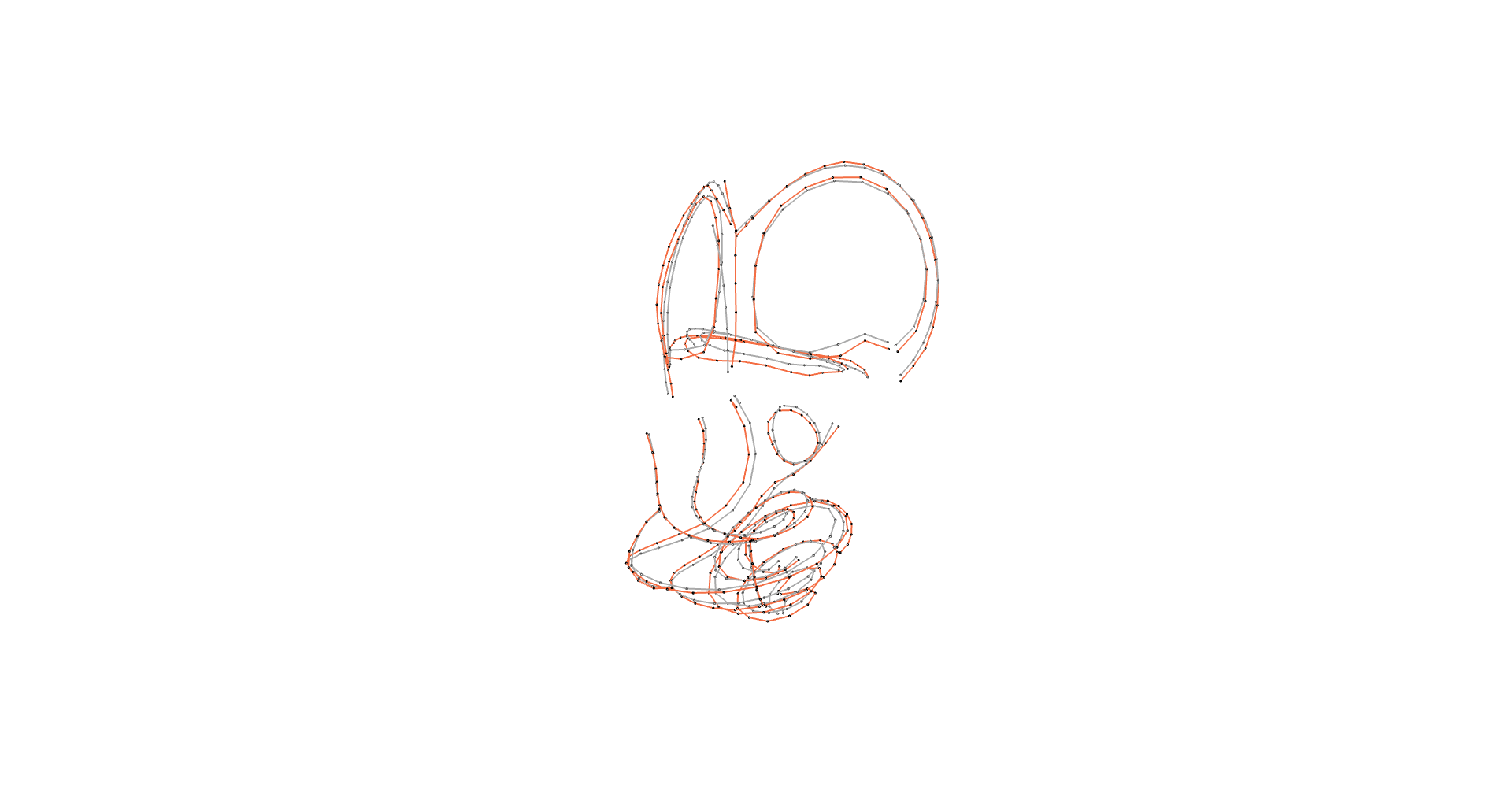

Supplement: Supplementary file 3 — Supplementary Data 1 [file 41467_2022_34656_MOESM3_ESM.zip › Supplementary data_1/Supplementary_material_1-1 Geometric morphometrics/bgPCA_306/mean_shapes_per_clade_bgPCA/Dromomerycidae-dl.png]

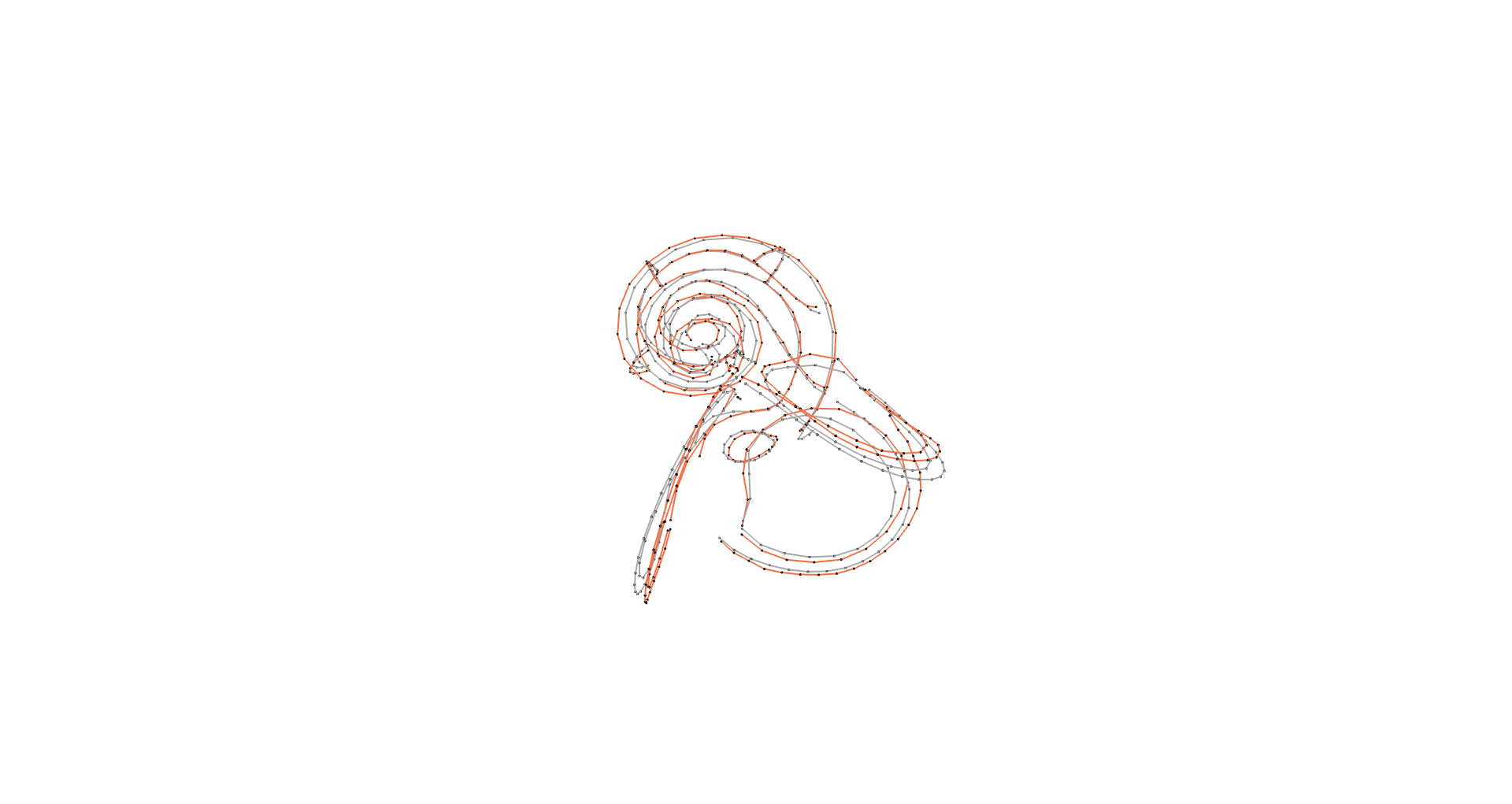

Supplement: Supplementary file 3 — Supplementary Data 1 [file 41467_2022_34656_MOESM3_ESM.zip › Supplementary data_1/Supplementary_material_1-1 Geometric morphometrics/bgPCA_306/mean_shapes_per_clade_bgPCA/Dromomerycidae-do.png]

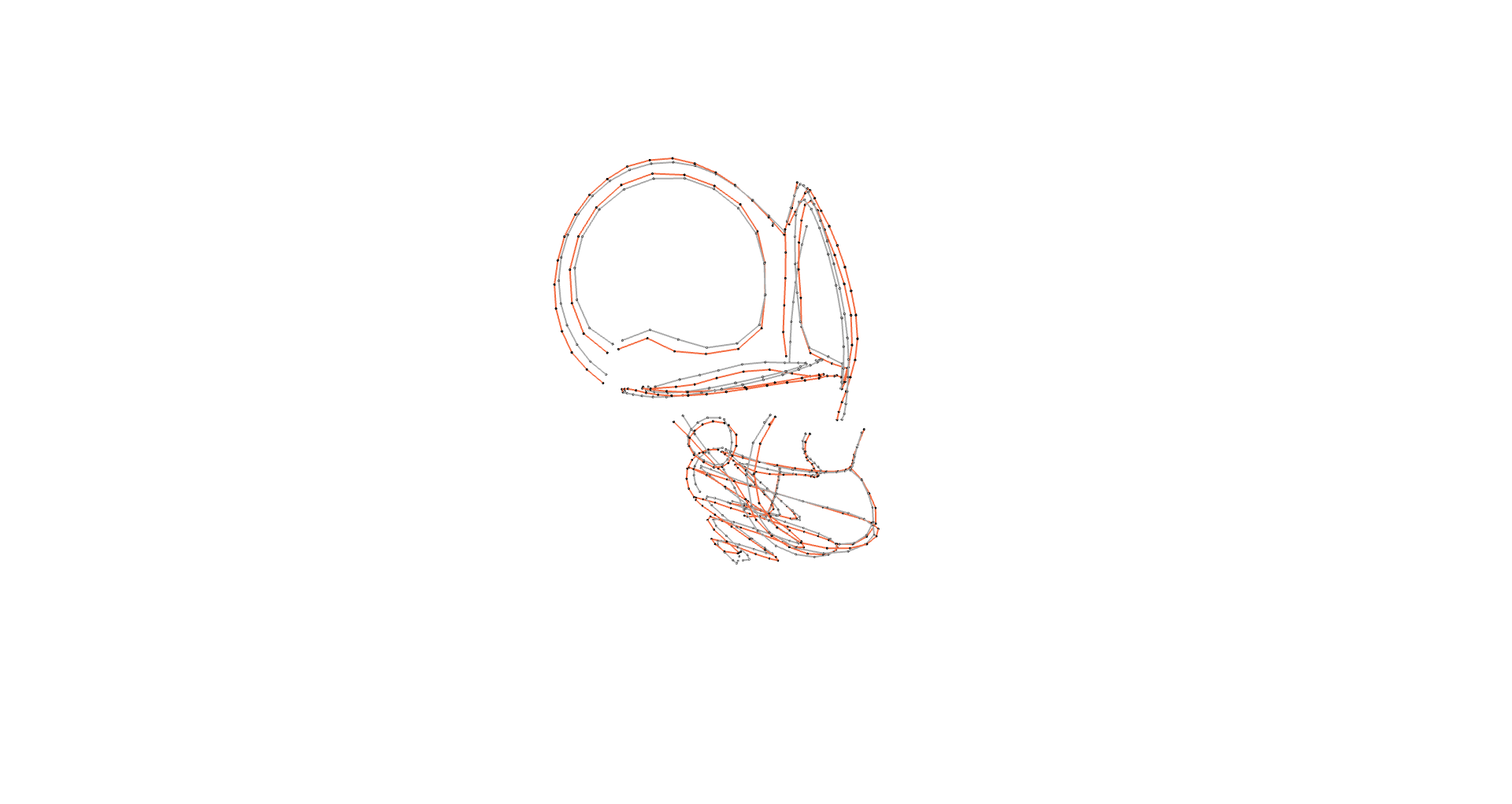

Supplement: Supplementary file 3 — Supplementary Data 1 [file 41467_2022_34656_MOESM3_ESM.zip › Supplementary data_1/Supplementary_material_1-1 Geometric morphometrics/bgPCA_306/mean_shapes_per_clade_bgPCA/Dromomerycidae-la.png]

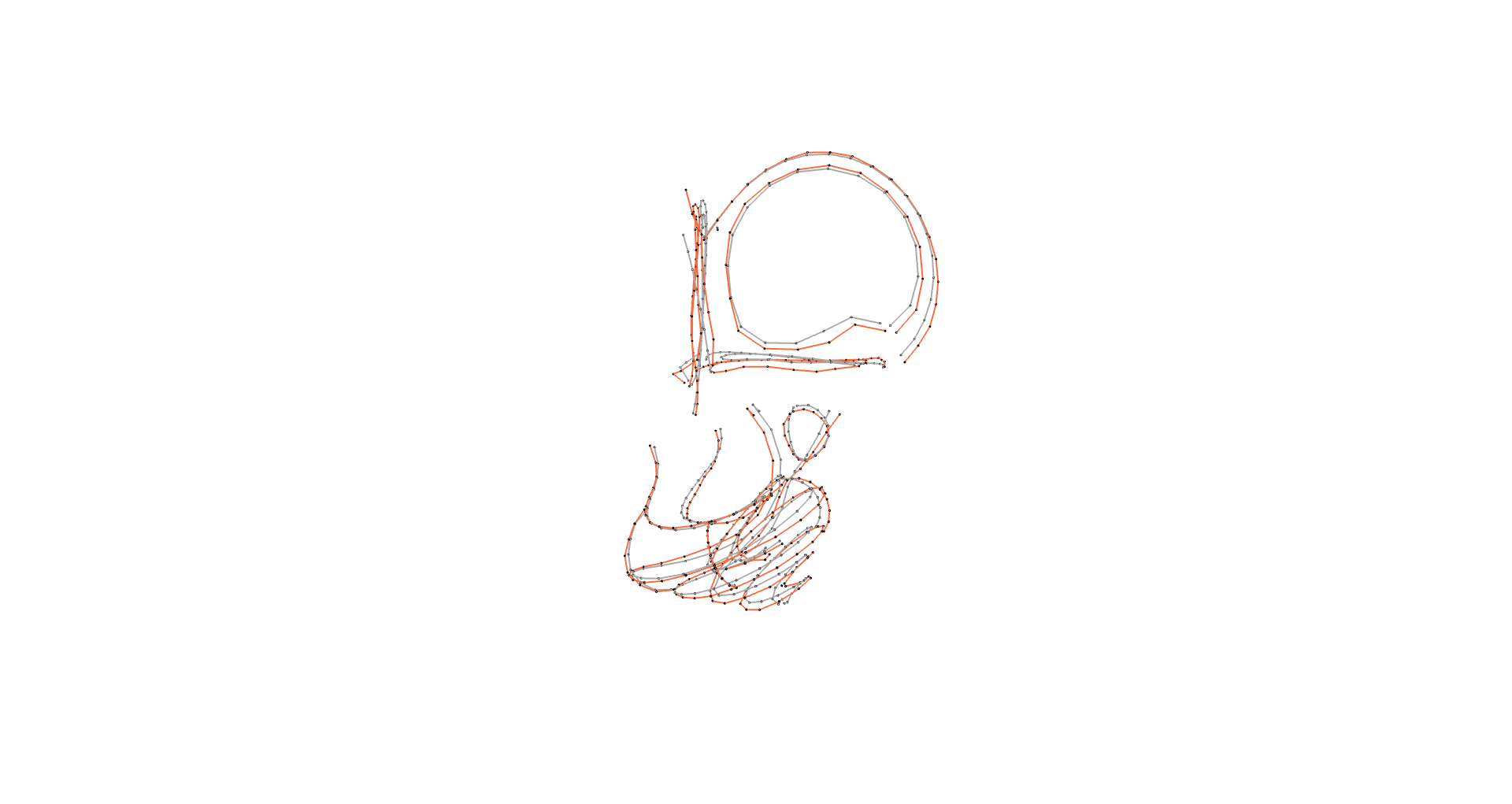

Supplement: Supplementary file 3 — Supplementary Data 1 [file 41467_2022_34656_MOESM3_ESM.zip › Supplementary data_1/Supplementary_material_1-1 Geometric morphometrics/bgPCA_306/mean_shapes_per_clade_bgPCA/Dromomerycidae-me.png]

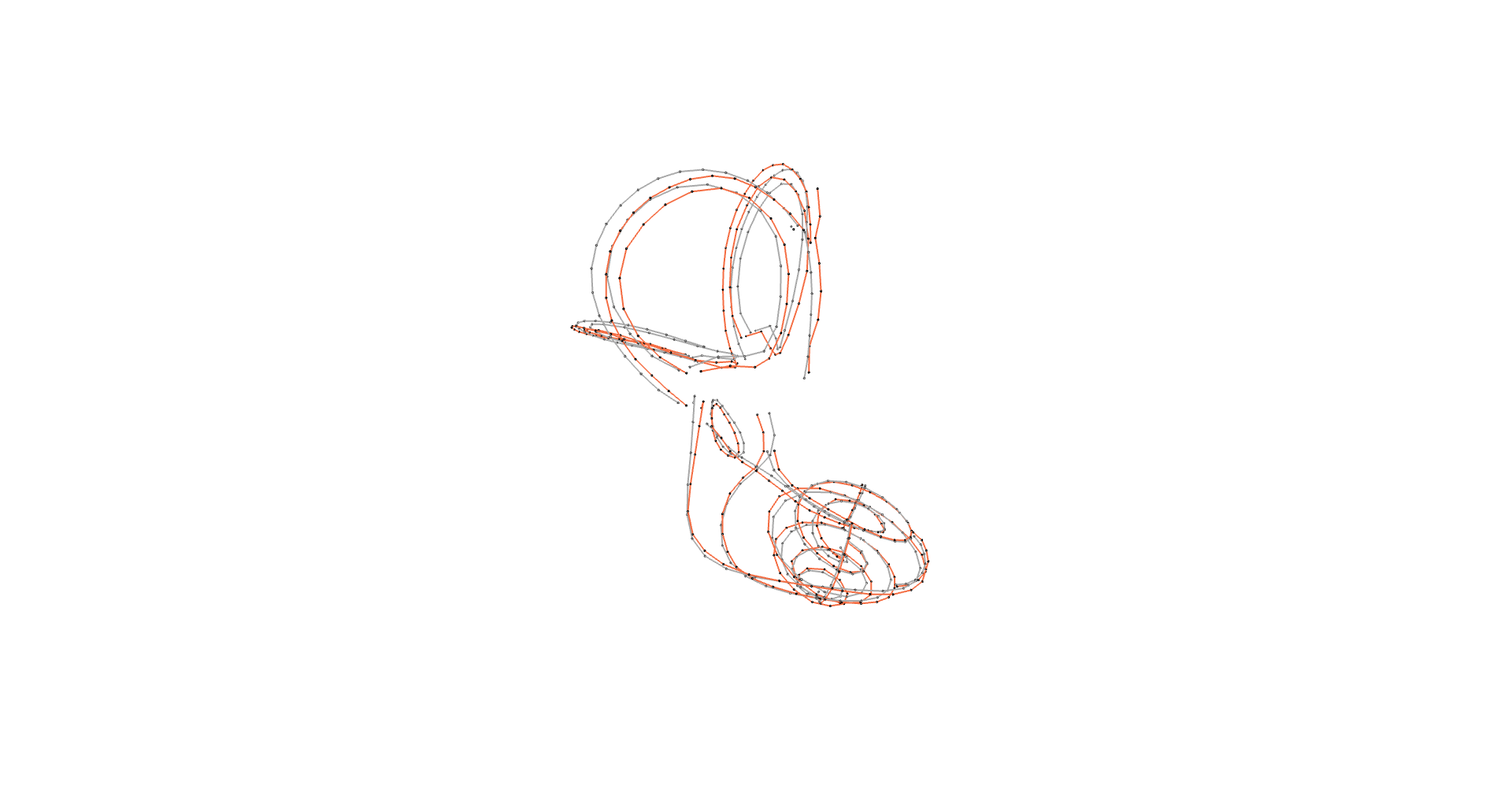

Supplement: Supplementary file 3 — Supplementary Data 1 [file 41467_2022_34656_MOESM3_ESM.zip › Supplementary data_1/Supplementary_material_1-1 Geometric morphometrics/bgPCA_306/mean_shapes_per_clade_bgPCA/Dromomerycidae-oc.png]

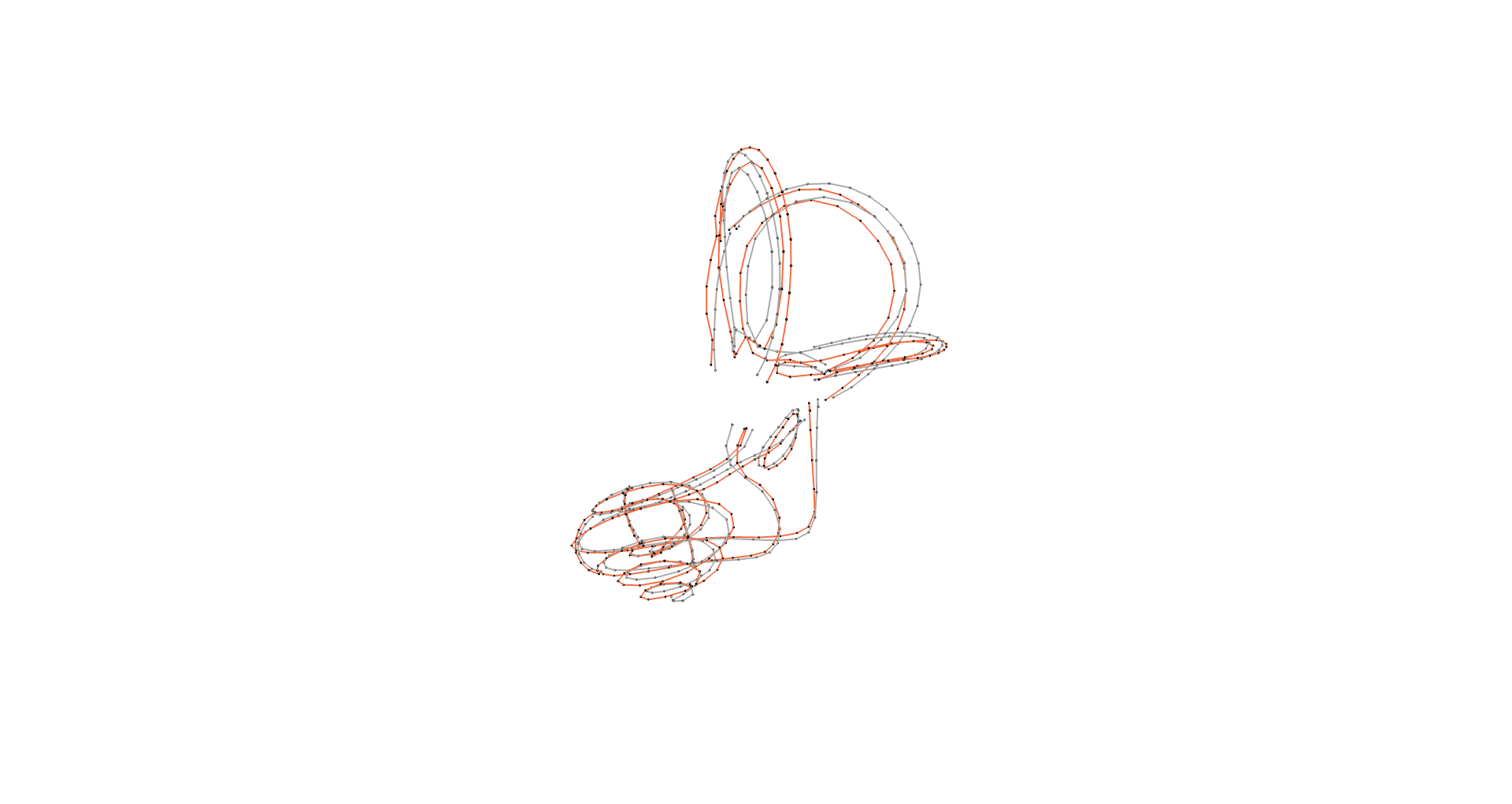

Supplement: Supplementary file 3 — Supplementary Data 1 [file 41467_2022_34656_MOESM3_ESM.zip › Supplementary data_1/Supplementary_material_1-1 Geometric morphometrics/bgPCA_306/mean_shapes_per_clade_bgPCA/Dromomerycidae-ro.png]

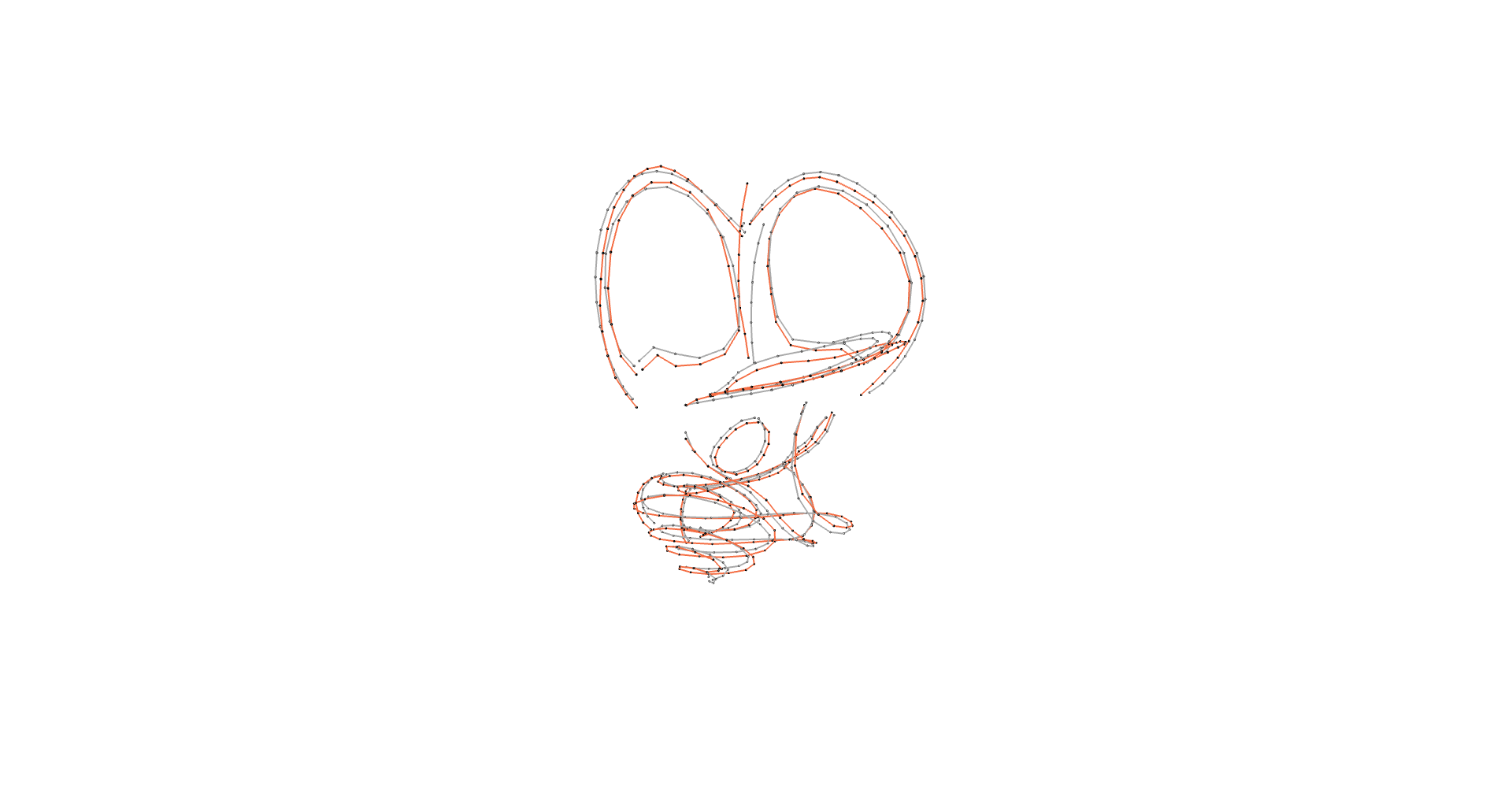

Supplement: Supplementary file 3 — Supplementary Data 1 [file 41467_2022_34656_MOESM3_ESM.zip › Supplementary data_1/Supplementary_material_1-1 Geometric morphometrics/bgPCA_306/mean_shapes_per_clade_bgPCA/Dromomerycidae-vl.png]

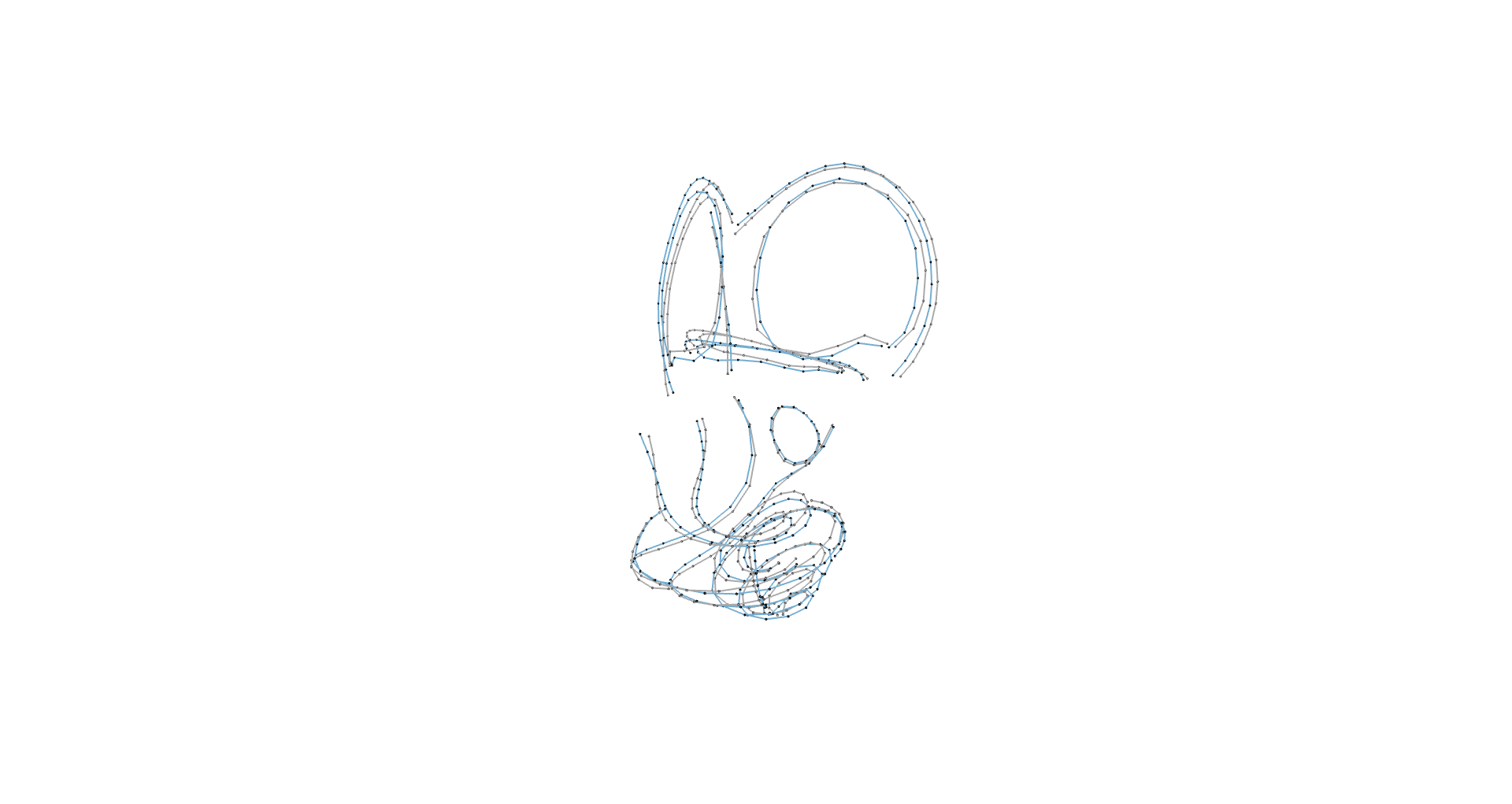

Supplement: Supplementary file 3 — Supplementary Data 1 [file 41467_2022_34656_MOESM3_ESM.zip › Supplementary data_1/Supplementary_material_1-1 Geometric morphometrics/bgPCA_306/mean_shapes_per_clade_bgPCA/Giraffidae-dl.png]

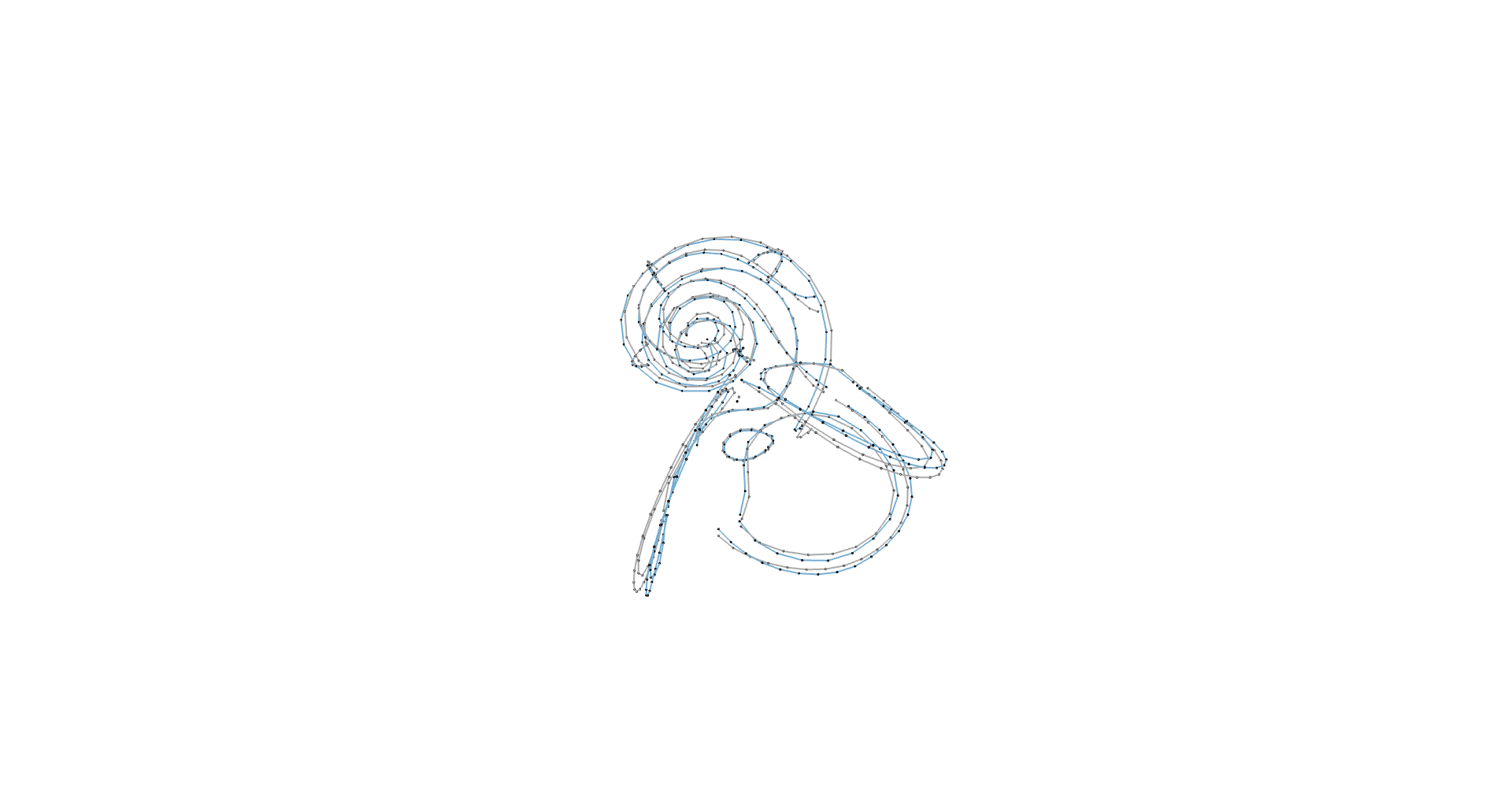

Supplement: Supplementary file 3 — Supplementary Data 1 [file 41467_2022_34656_MOESM3_ESM.zip › Supplementary data_1/Supplementary_material_1-1 Geometric morphometrics/bgPCA_306/mean_shapes_per_clade_bgPCA/Giraffidae-do.png]

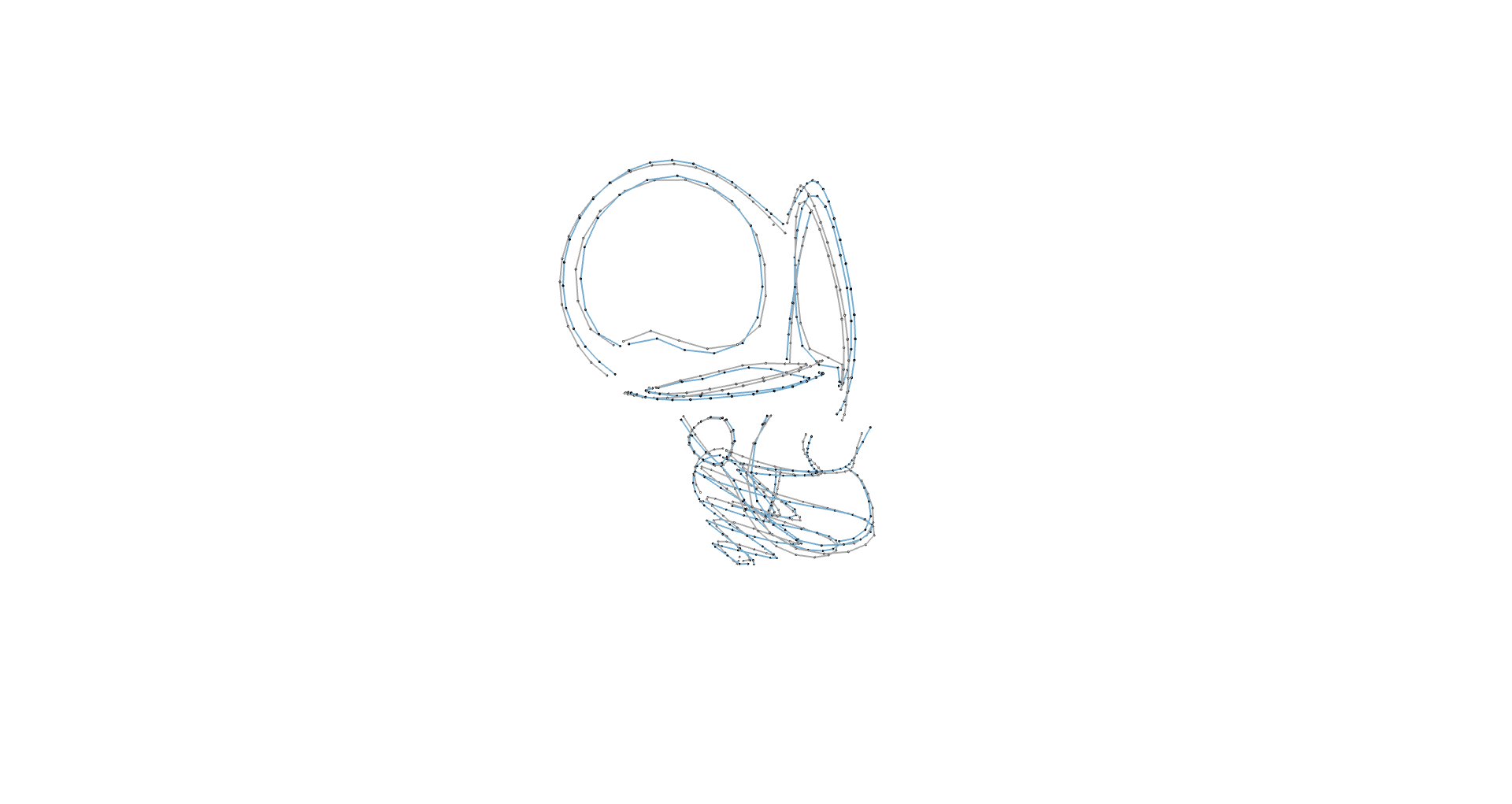

Supplement: Supplementary file 3 — Supplementary Data 1 [file 41467_2022_34656_MOESM3_ESM.zip › Supplementary data_1/Supplementary_material_1-1 Geometric morphometrics/bgPCA_306/mean_shapes_per_clade_bgPCA/Giraffidae-la.png]

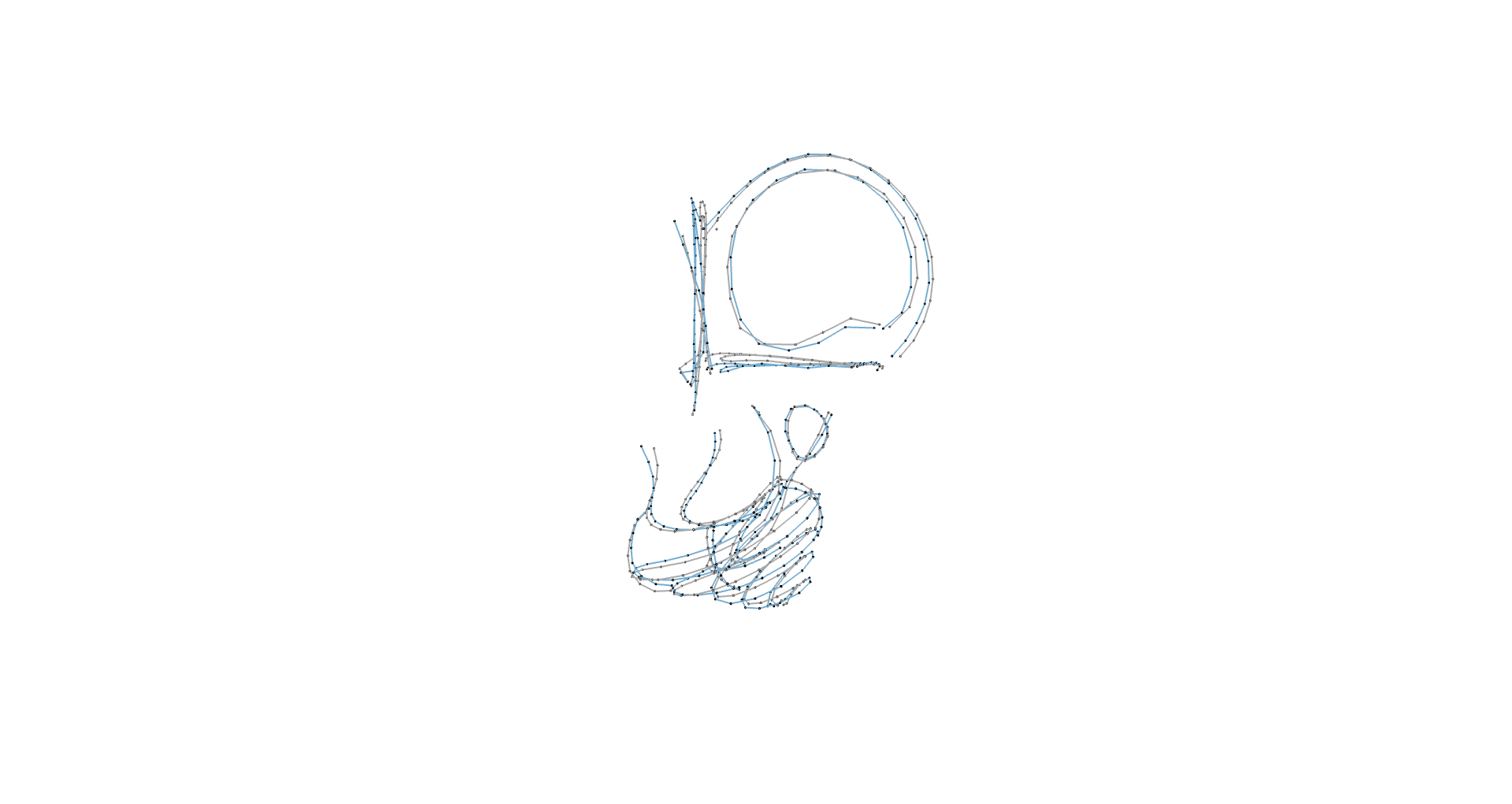

Supplement: Supplementary file 3 — Supplementary Data 1 [file 41467_2022_34656_MOESM3_ESM.zip › Supplementary data_1/Supplementary_material_1-1 Geometric morphometrics/bgPCA_306/mean_shapes_per_clade_bgPCA/Giraffidae-me.png]

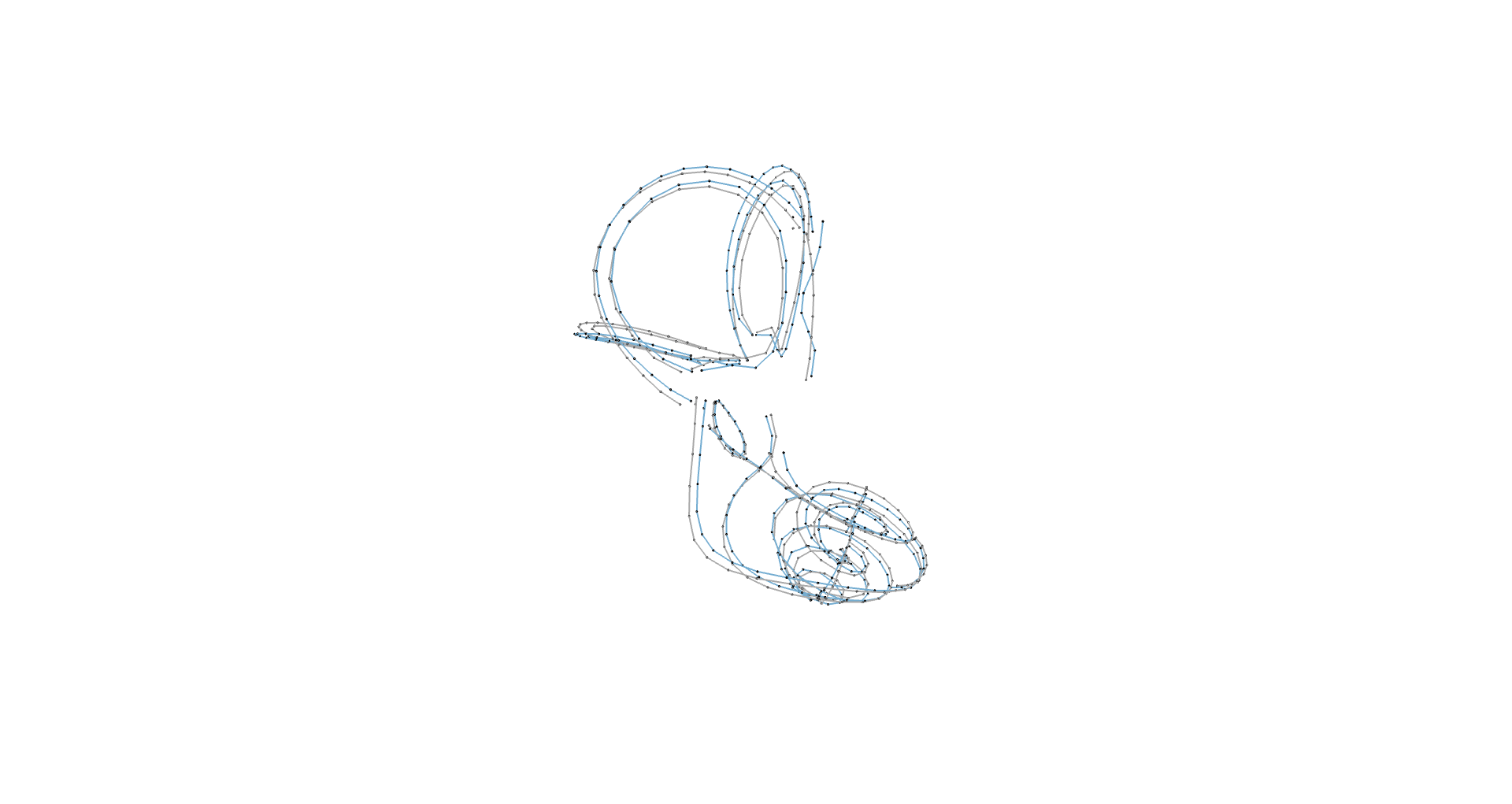

Supplement: Supplementary file 3 — Supplementary Data 1 [file 41467_2022_34656_MOESM3_ESM.zip › Supplementary data_1/Supplementary_material_1-1 Geometric morphometrics/bgPCA_306/mean_shapes_per_clade_bgPCA/Giraffidae-oc.png]

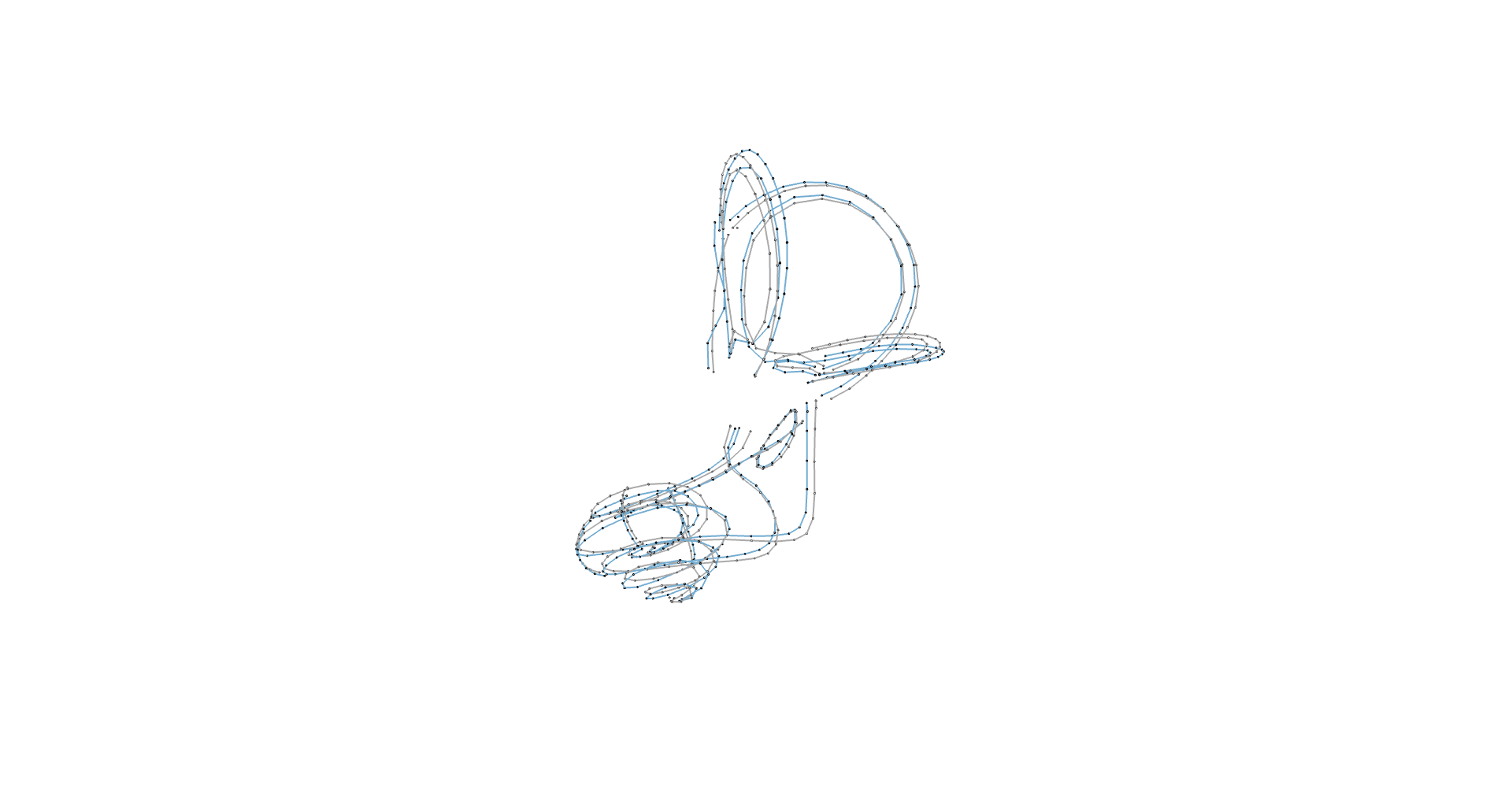

Supplement: Supplementary file 3 — Supplementary Data 1 [file 41467_2022_34656_MOESM3_ESM.zip › Supplementary data_1/Supplementary_material_1-1 Geometric morphometrics/bgPCA_306/mean_shapes_per_clade_bgPCA/Giraffidae-ro.png]

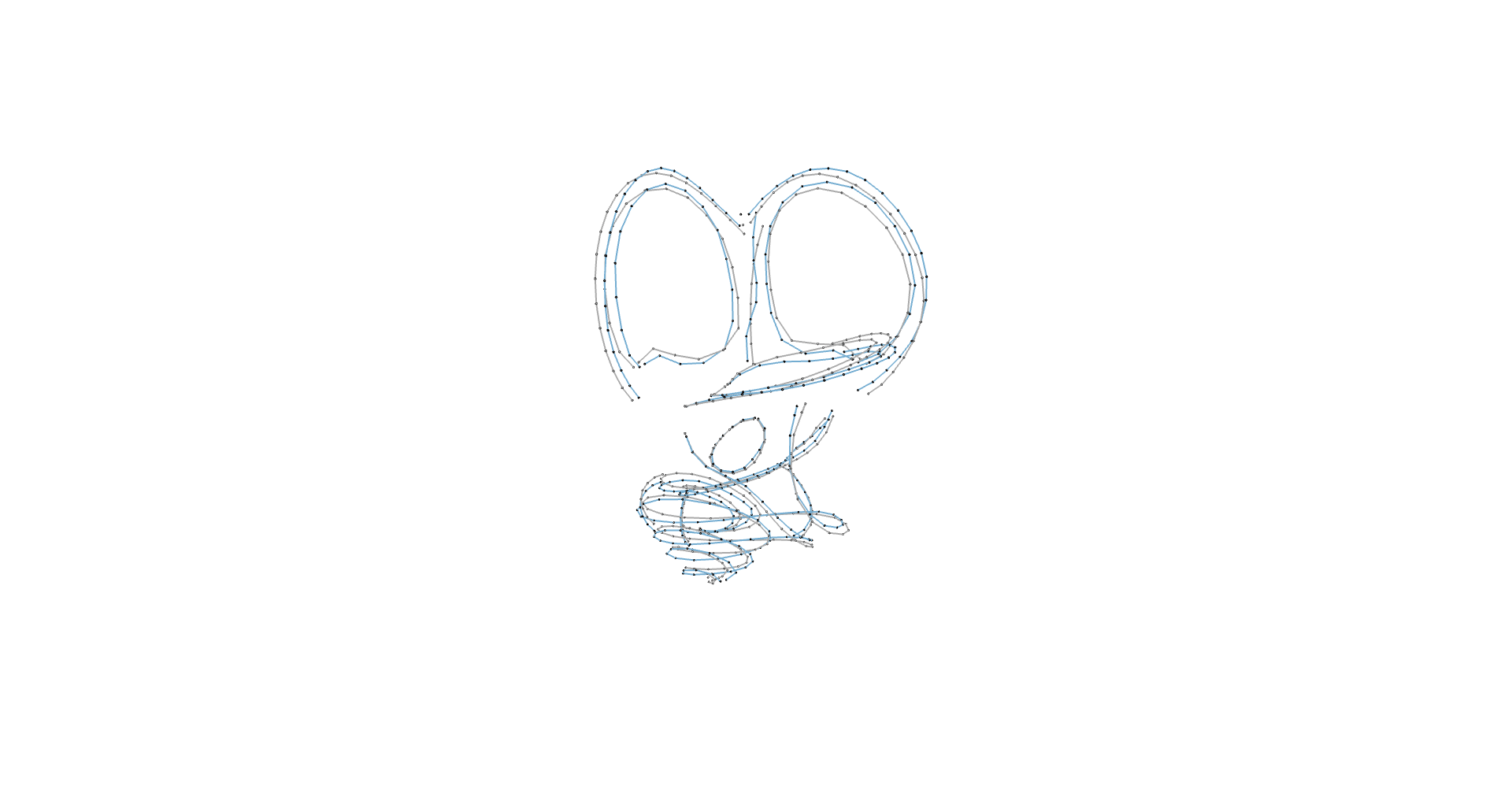

Supplement: Supplementary file 3 — Supplementary Data 1 [file 41467_2022_34656_MOESM3_ESM.zip › Supplementary data_1/Supplementary_material_1-1 Geometric morphometrics/bgPCA_306/mean_shapes_per_clade_bgPCA/Giraffidae-vl.png]

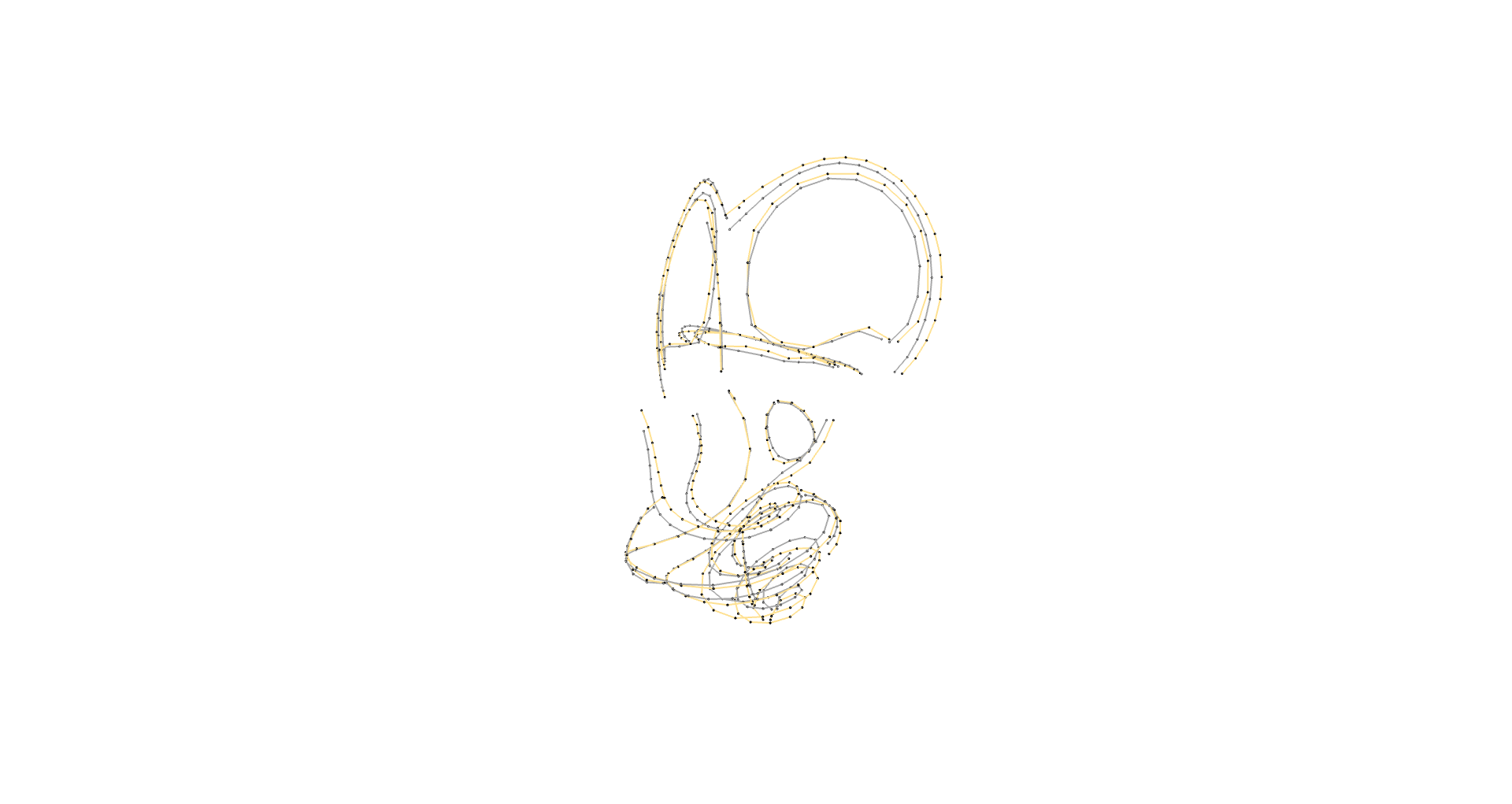

Supplement: Supplementary file 3 — Supplementary Data 1 [file 41467_2022_34656_MOESM3_ESM.zip › Supplementary data_1/Supplementary_material_1-1 Geometric morphometrics/bgPCA_306/mean_shapes_per_clade_bgPCA/Moschidae-dl.png]

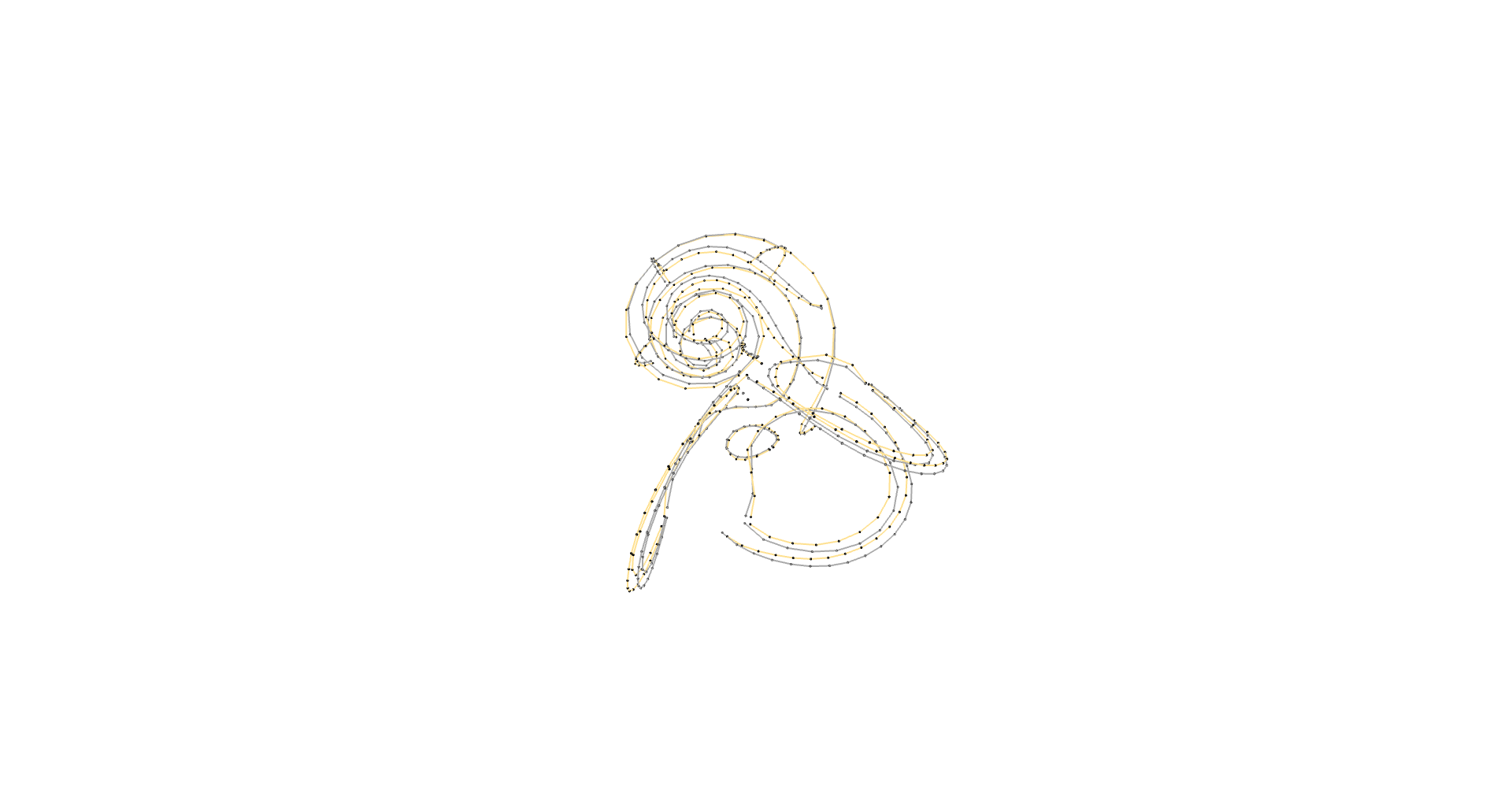

Supplement: Supplementary file 3 — Supplementary Data 1 [file 41467_2022_34656_MOESM3_ESM.zip › Supplementary data_1/Supplementary_material_1-1 Geometric morphometrics/bgPCA_306/mean_shapes_per_clade_bgPCA/Moschidae-do.png]

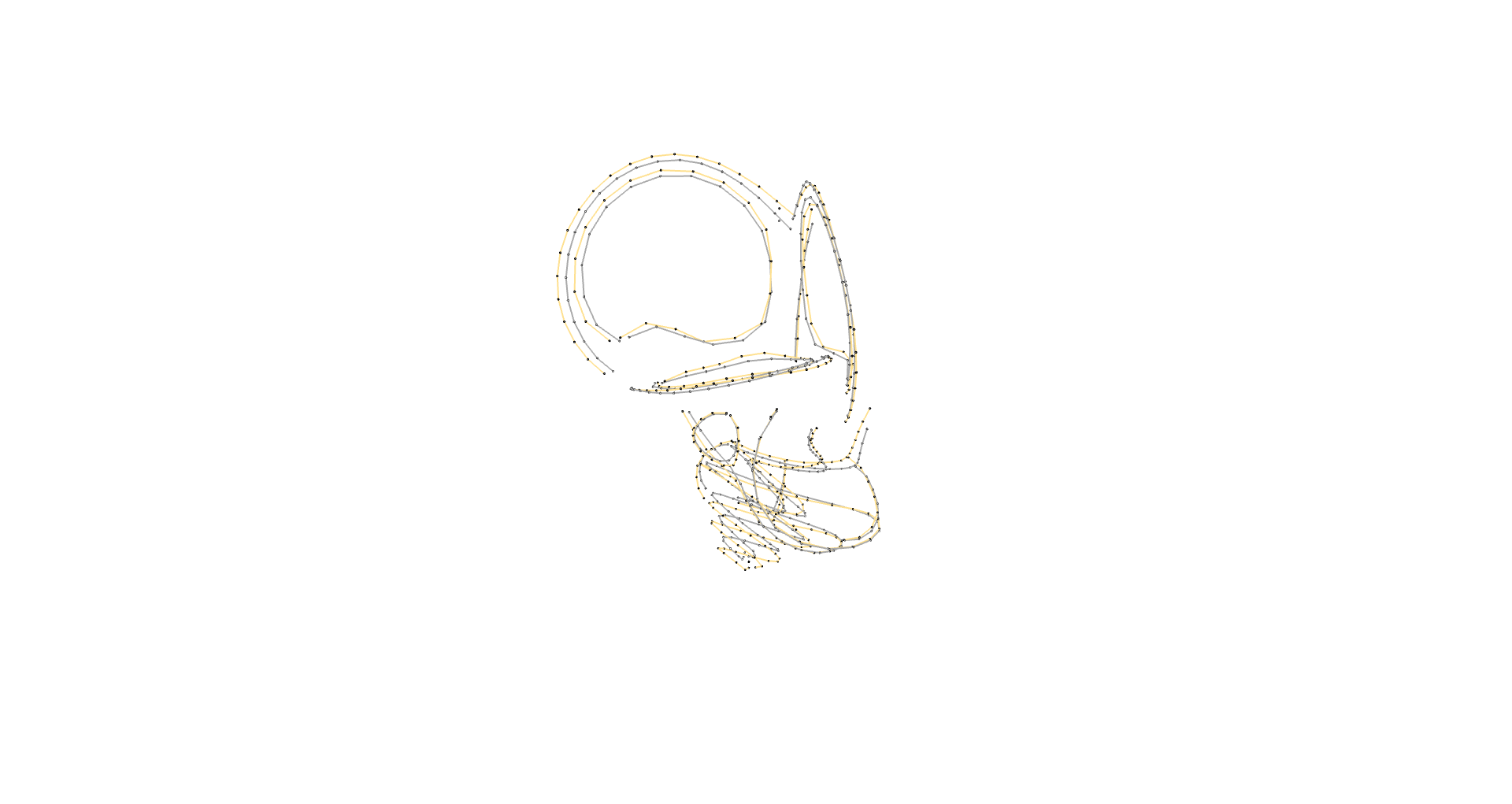

Supplement: Supplementary file 3 — Supplementary Data 1 [file 41467_2022_34656_MOESM3_ESM.zip › Supplementary data_1/Supplementary_material_1-1 Geometric morphometrics/bgPCA_306/mean_shapes_per_clade_bgPCA/Moschidae-la.png]

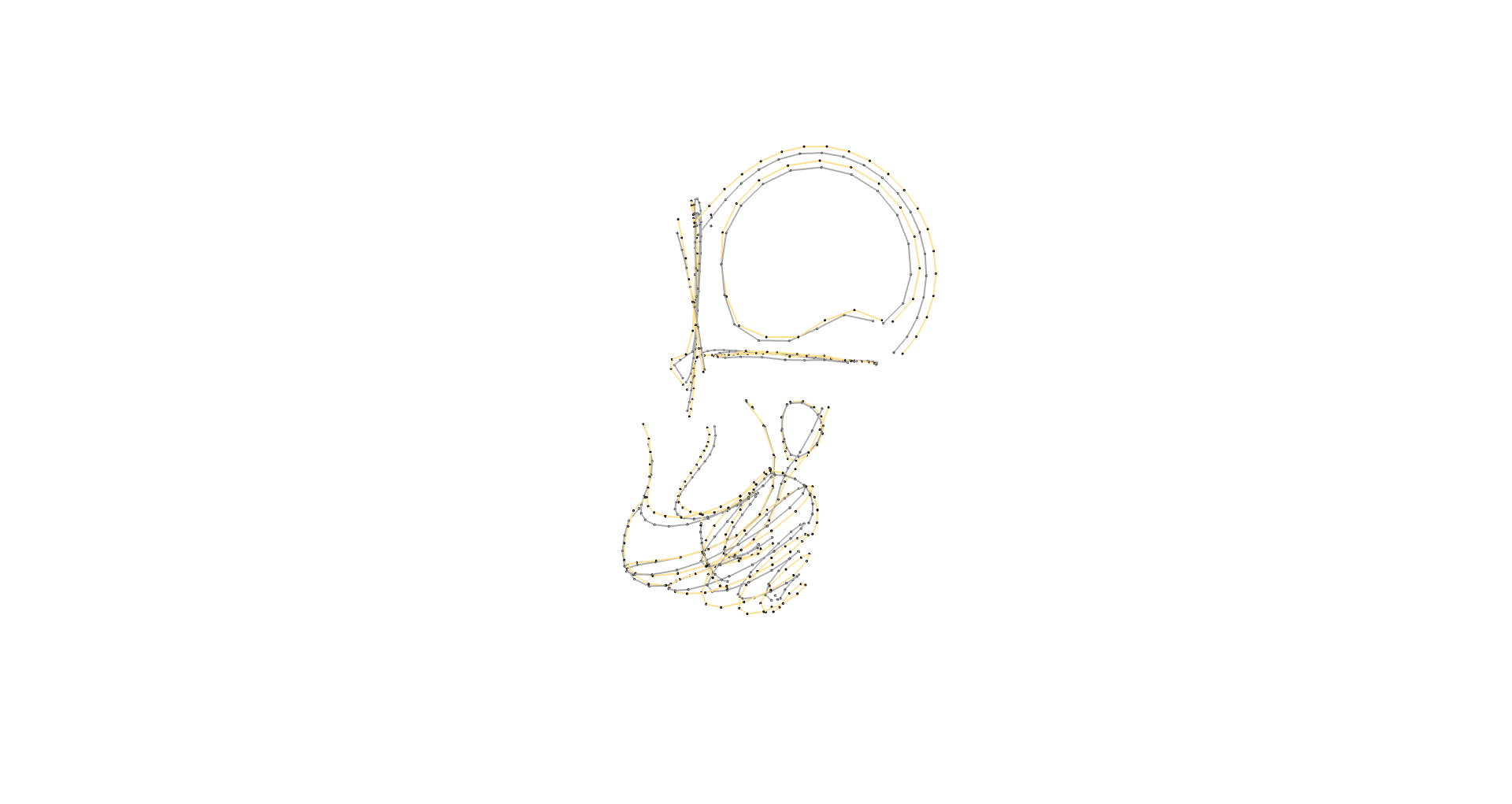

Supplement: Supplementary file 3 — Supplementary Data 1 [file 41467_2022_34656_MOESM3_ESM.zip › Supplementary data_1/Supplementary_material_1-1 Geometric morphometrics/bgPCA_306/mean_shapes_per_clade_bgPCA/Moschidae-me.png]

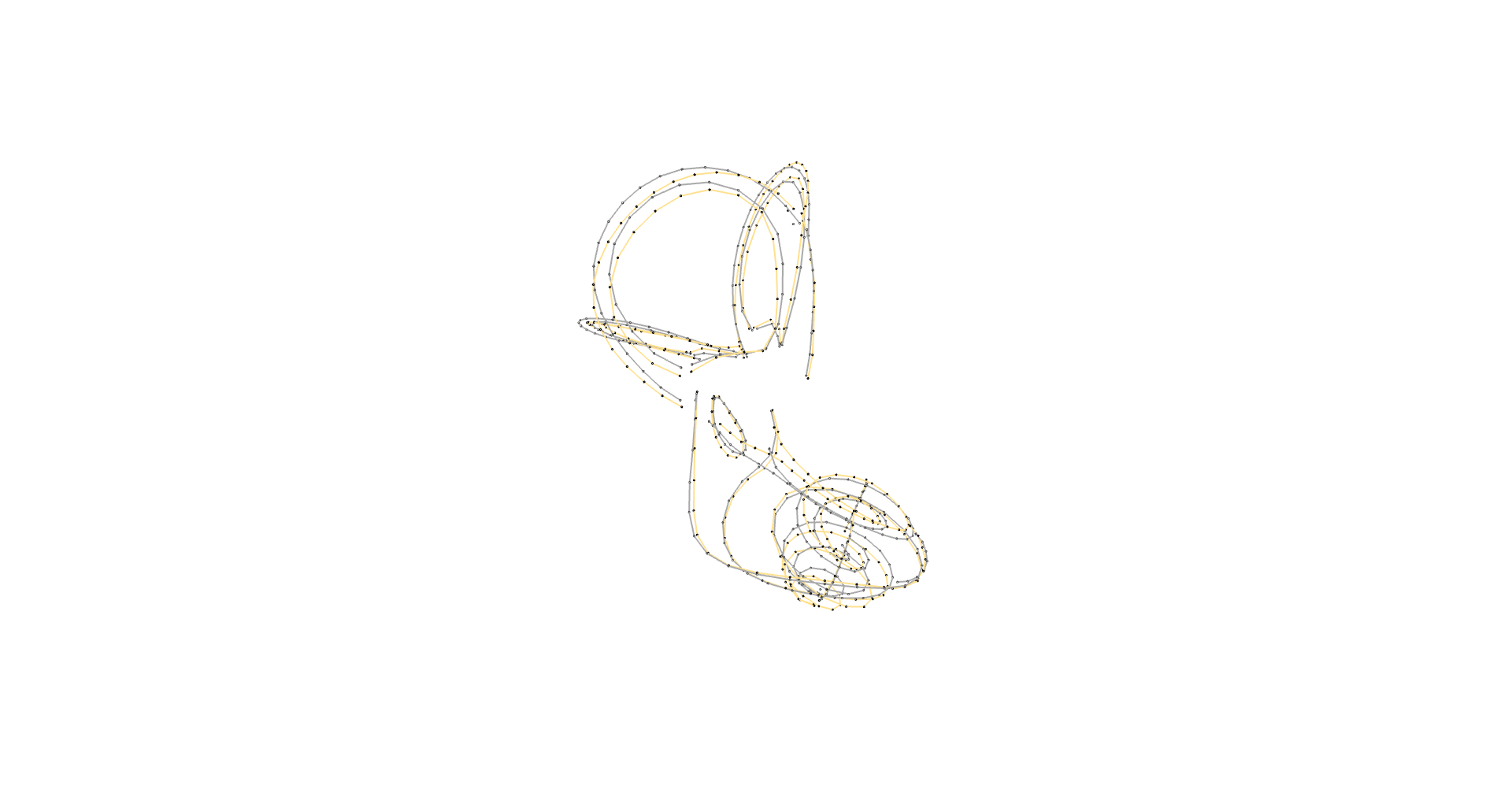

Supplement: Supplementary file 3 — Supplementary Data 1 [file 41467_2022_34656_MOESM3_ESM.zip › Supplementary data_1/Supplementary_material_1-1 Geometric morphometrics/bgPCA_306/mean_shapes_per_clade_bgPCA/Moschidae-oc.png]

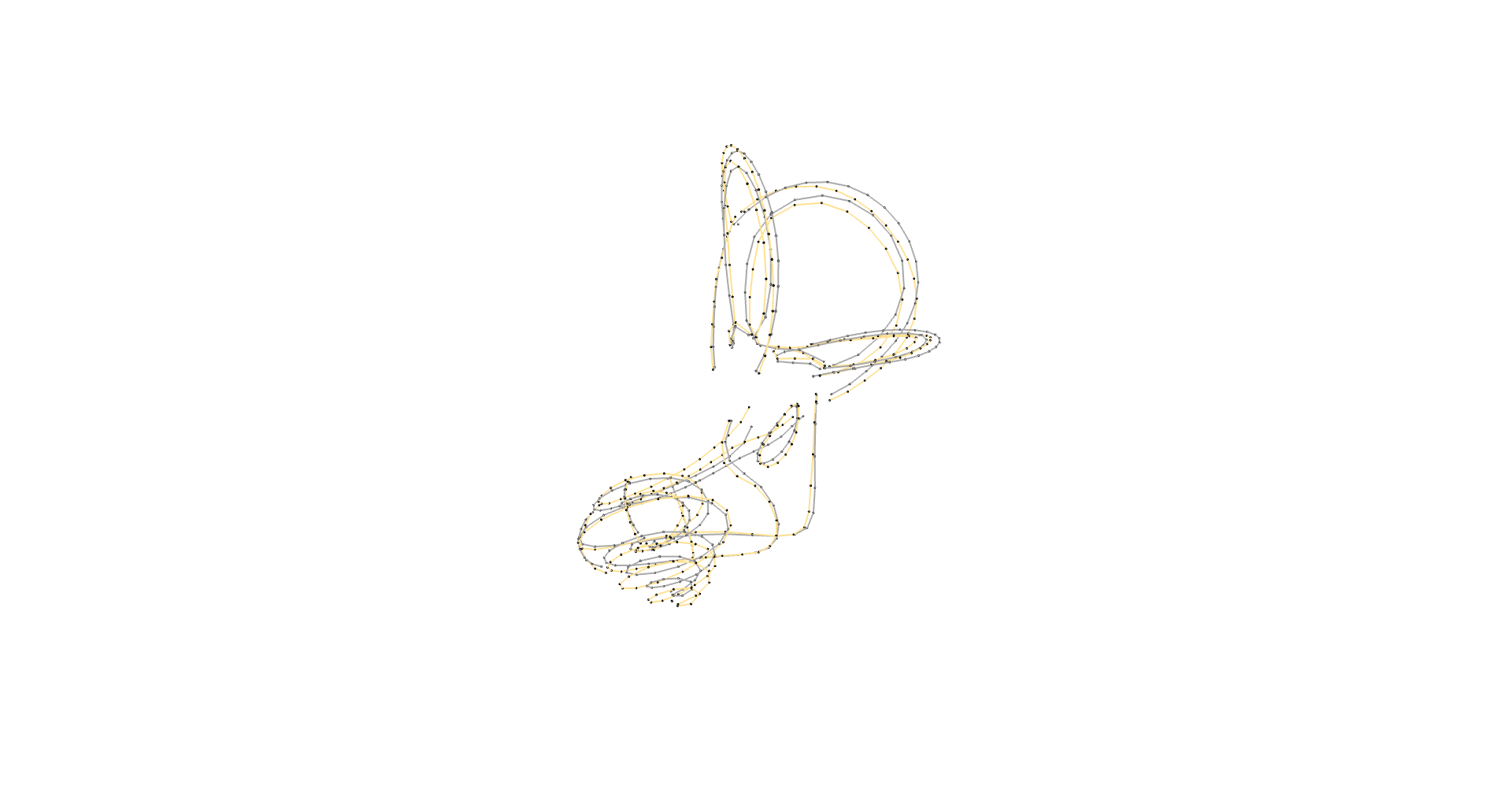

Supplement: Supplementary file 3 — Supplementary Data 1 [file 41467_2022_34656_MOESM3_ESM.zip › Supplementary data_1/Supplementary_material_1-1 Geometric morphometrics/bgPCA_306/mean_shapes_per_clade_bgPCA/Moschidae-ro.png]

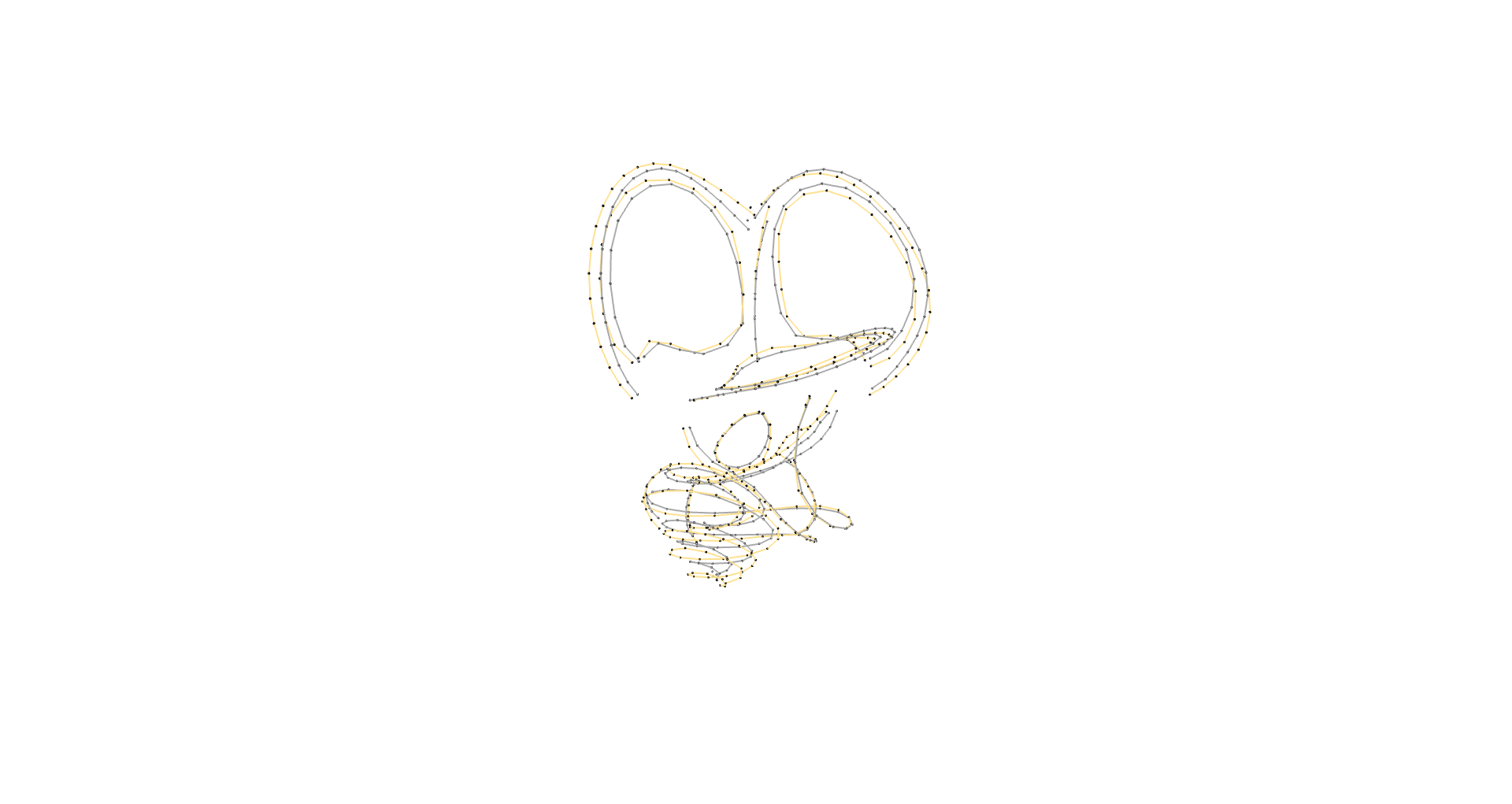

Supplement: Supplementary file 3 — Supplementary Data 1 [file 41467_2022_34656_MOESM3_ESM.zip › Supplementary data_1/Supplementary_material_1-1 Geometric morphometrics/bgPCA_306/mean_shapes_per_clade_bgPCA/Moschidae-vl.png]

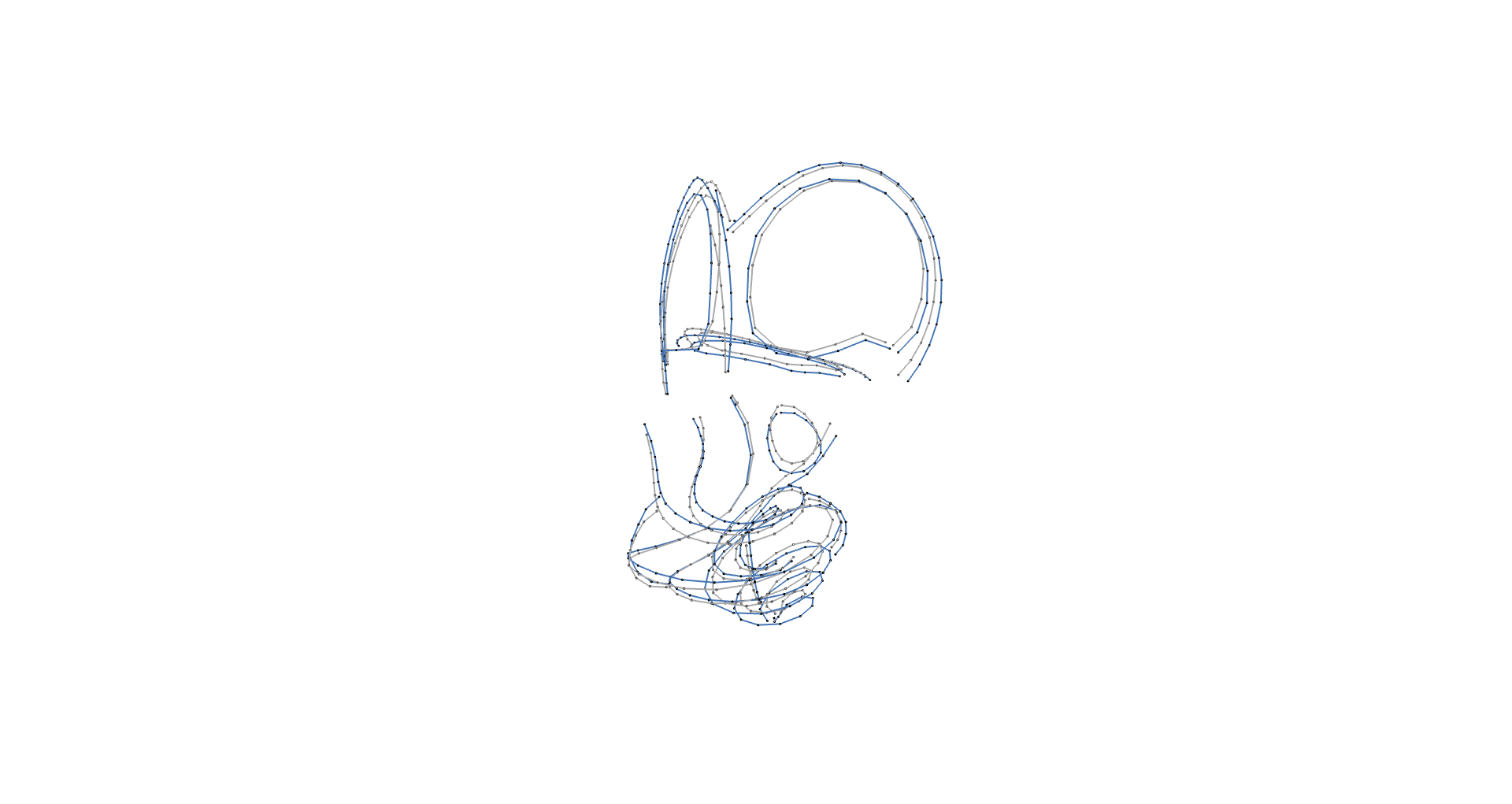

Supplement: Supplementary file 3 — Supplementary Data 1 [file 41467_2022_34656_MOESM3_ESM.zip › Supplementary data_1/Supplementary_material_1-1 Geometric morphometrics/bgPCA_306/mean_shapes_per_clade_bgPCA/Stem_Pecora-dl.png]

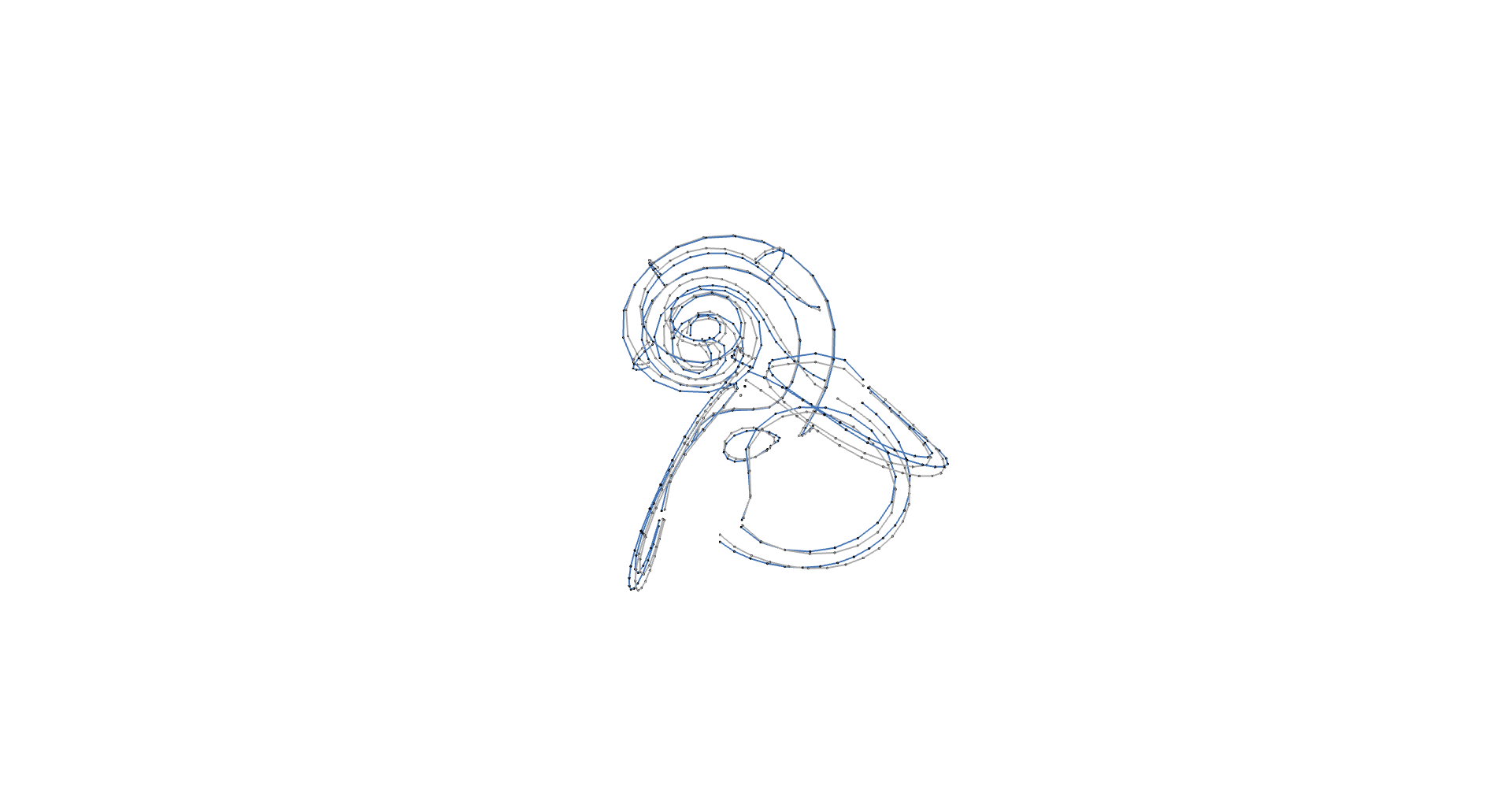

Supplement: Supplementary file 3 — Supplementary Data 1 [file 41467_2022_34656_MOESM3_ESM.zip › Supplementary data_1/Supplementary_material_1-1 Geometric morphometrics/bgPCA_306/mean_shapes_per_clade_bgPCA/Stem_Pecora-do.png]

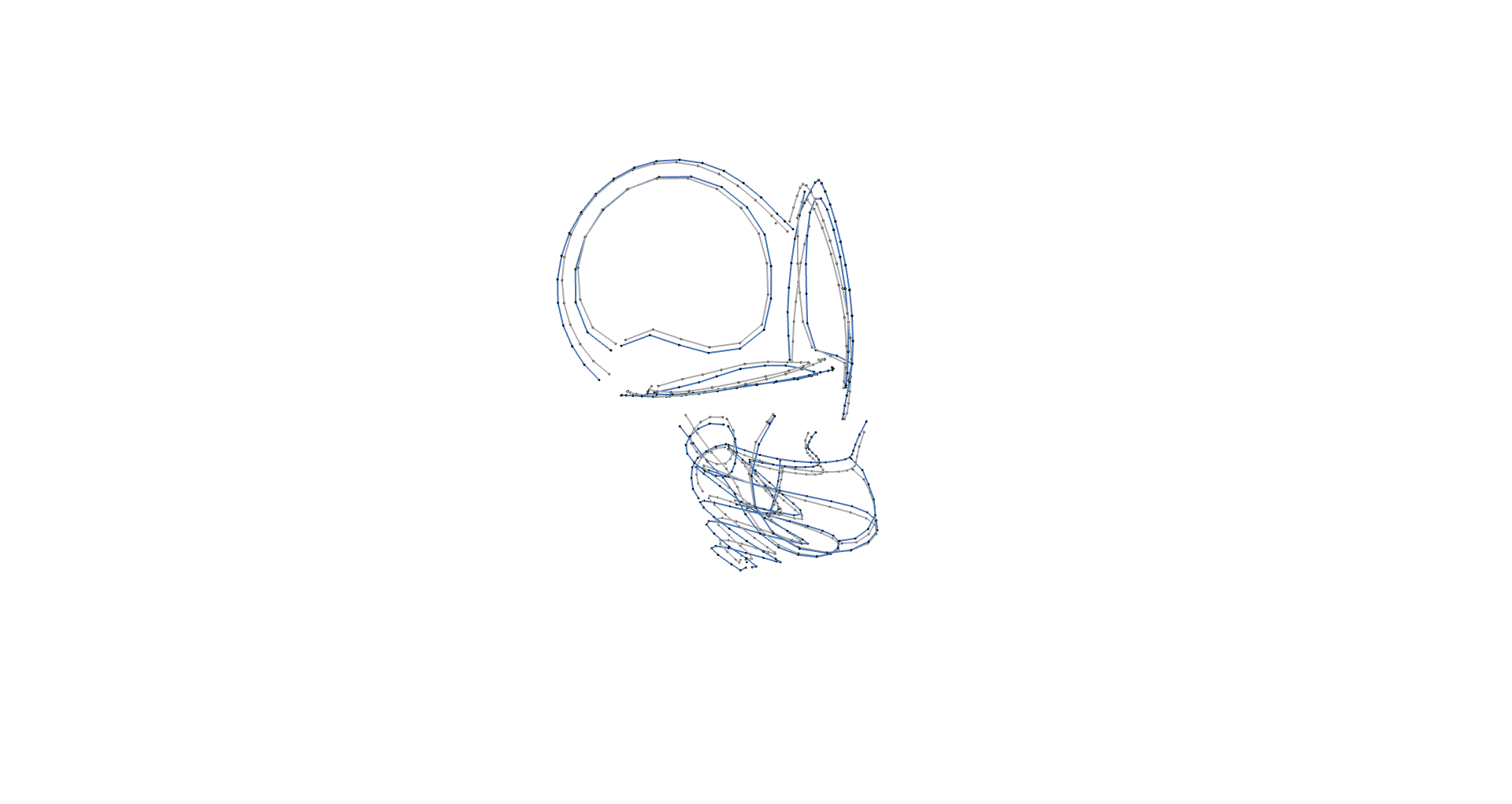

Supplement: Supplementary file 3 — Supplementary Data 1 [file 41467_2022_34656_MOESM3_ESM.zip › Supplementary data_1/Supplementary_material_1-1 Geometric morphometrics/bgPCA_306/mean_shapes_per_clade_bgPCA/Stem_Pecora-la.png]

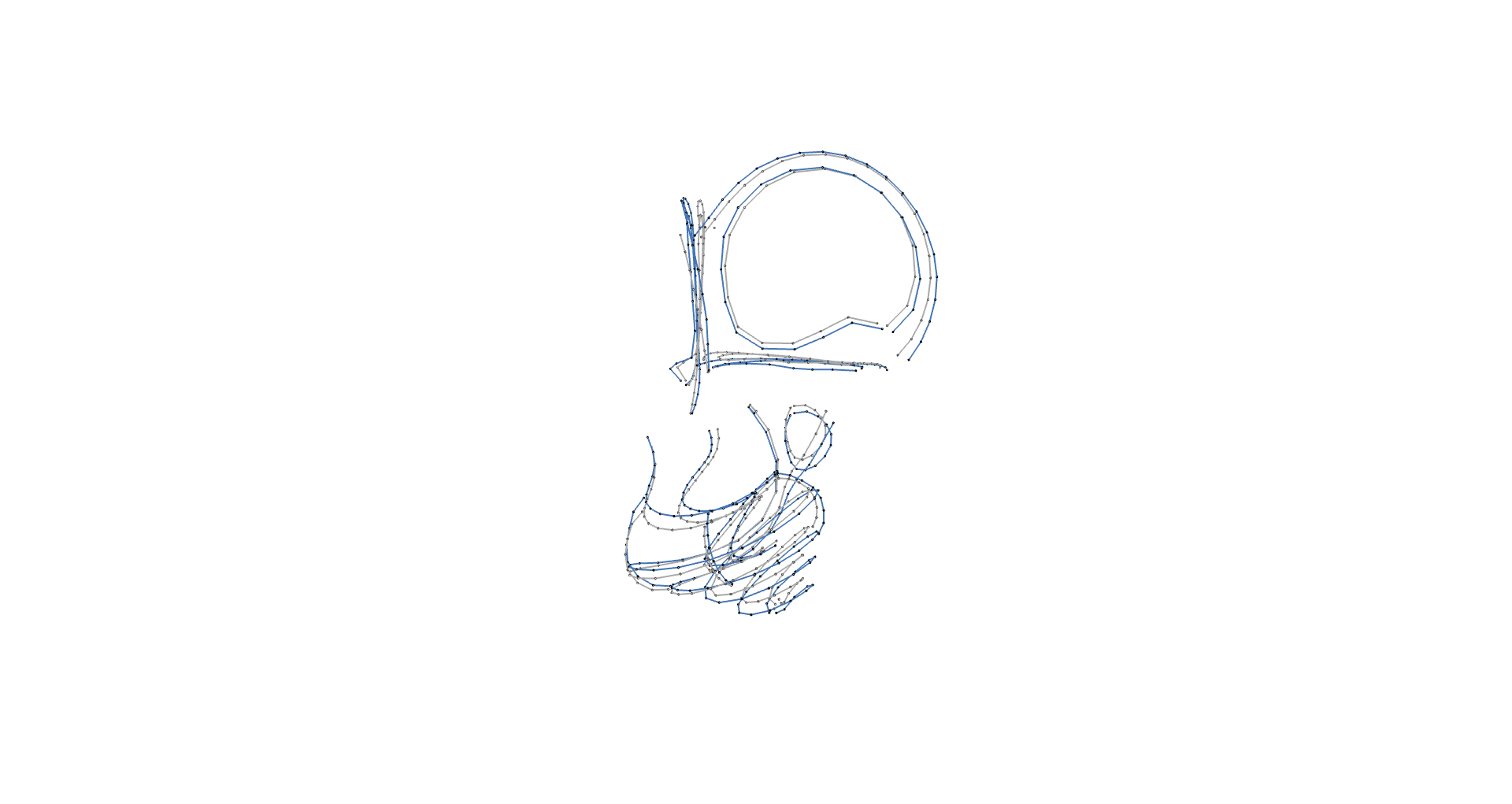

Supplement: Supplementary file 3 — Supplementary Data 1 [file 41467_2022_34656_MOESM3_ESM.zip › Supplementary data_1/Supplementary_material_1-1 Geometric morphometrics/bgPCA_306/mean_shapes_per_clade_bgPCA/Stem_Pecora-me.png]

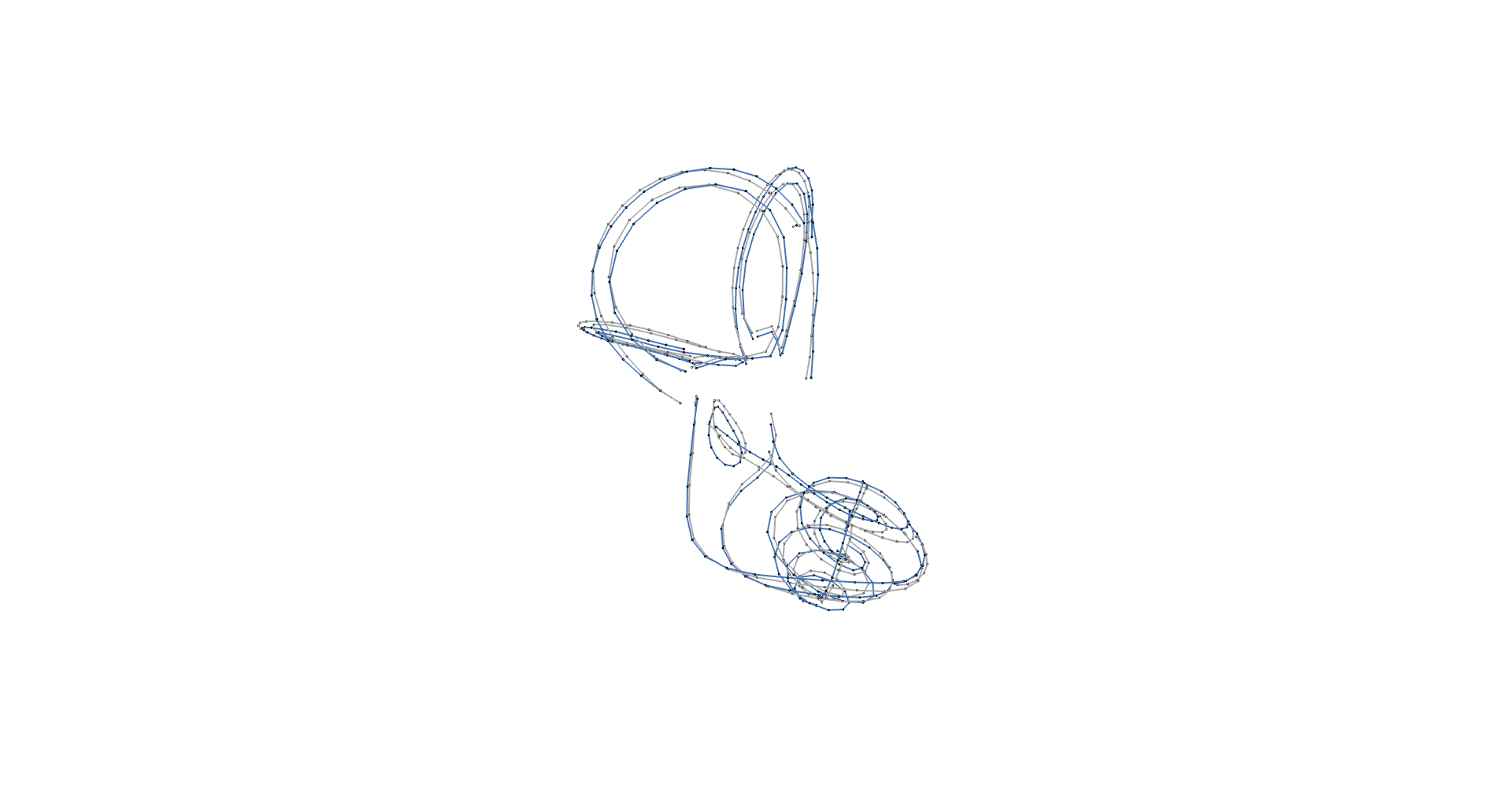

Supplement: Supplementary file 3 — Supplementary Data 1 [file 41467_2022_34656_MOESM3_ESM.zip › Supplementary data_1/Supplementary_material_1-1 Geometric morphometrics/bgPCA_306/mean_shapes_per_clade_bgPCA/Stem_Pecora-oc.png]

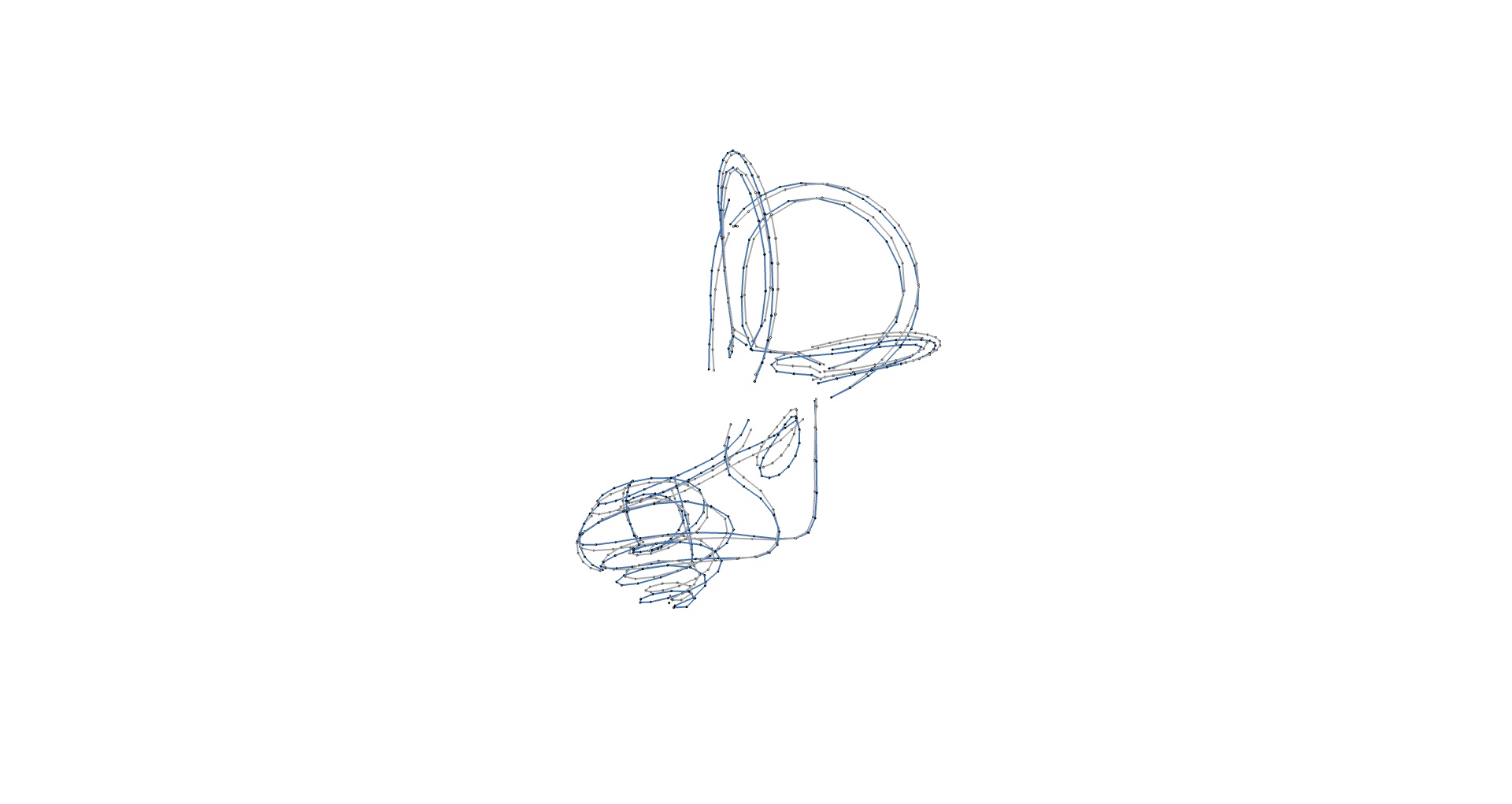

Supplement: Supplementary file 3 — Supplementary Data 1 [file 41467_2022_34656_MOESM3_ESM.zip › Supplementary data_1/Supplementary_material_1-1 Geometric morphometrics/bgPCA_306/mean_shapes_per_clade_bgPCA/Stem_Pecora-ro.png]

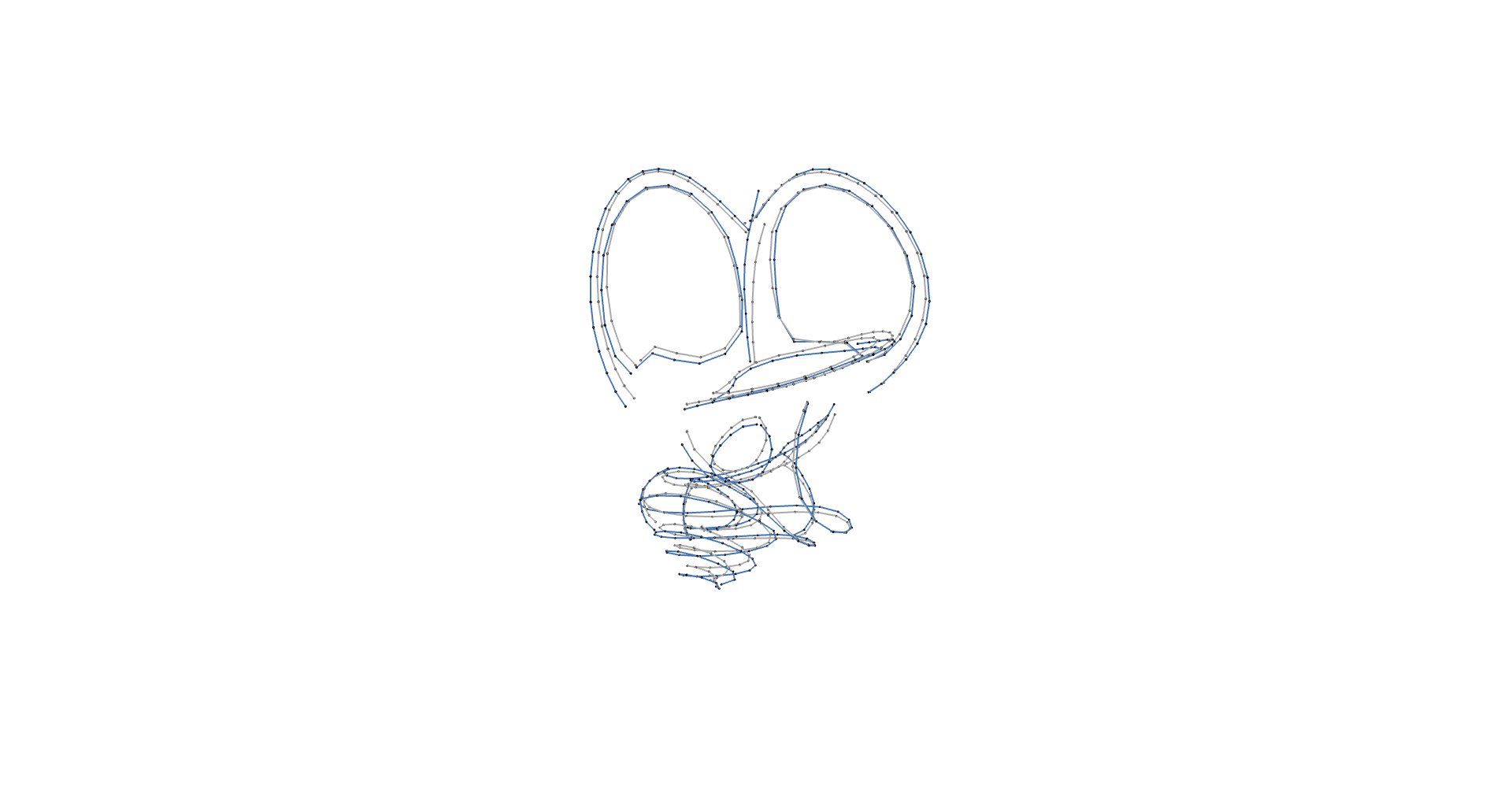

Supplement: Supplementary file 3 — Supplementary Data 1 [file 41467_2022_34656_MOESM3_ESM.zip › Supplementary data_1/Supplementary_material_1-1 Geometric morphometrics/bgPCA_306/mean_shapes_per_clade_bgPCA/Stem_Pecora-vl.png]

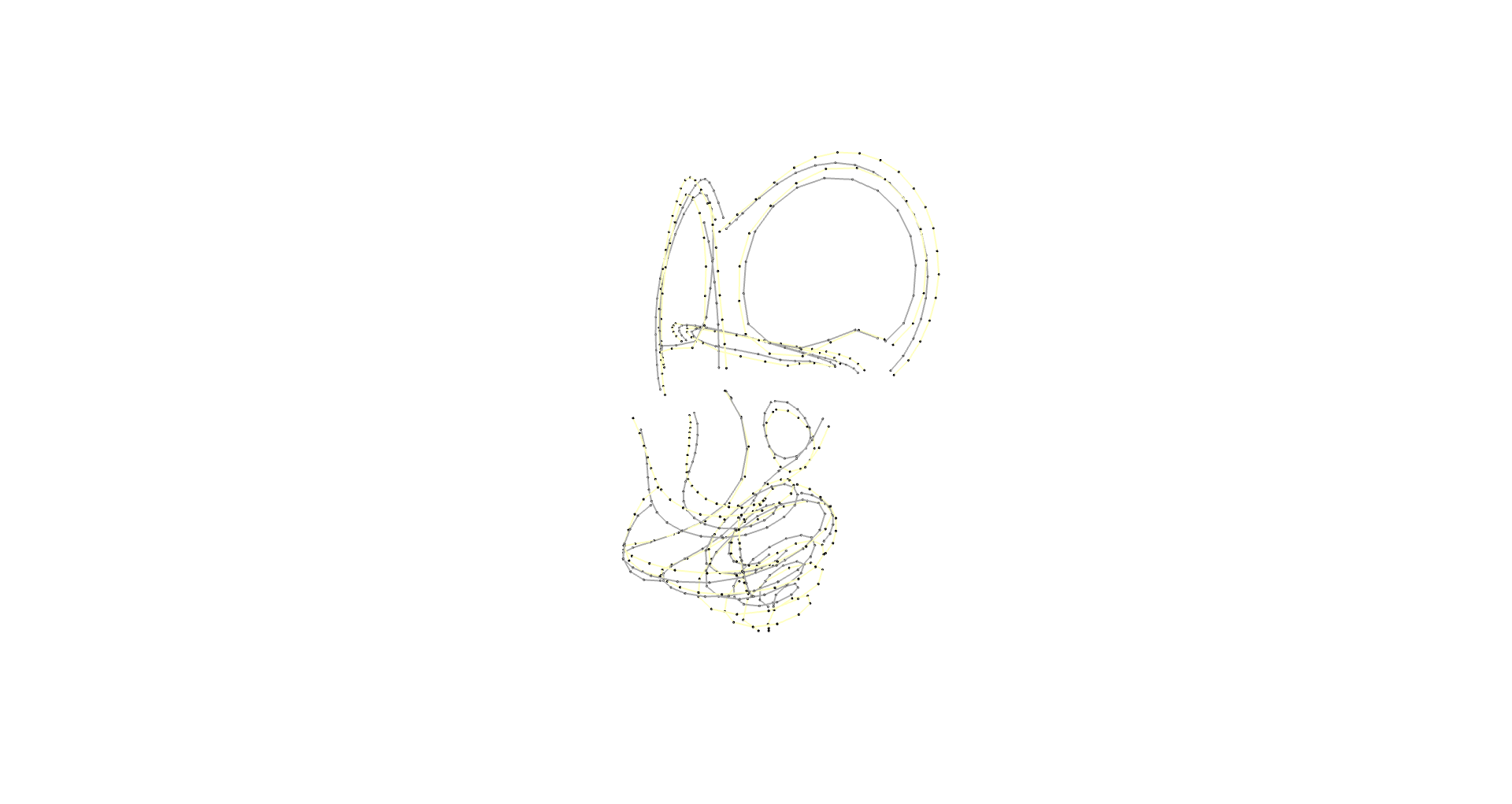

Supplement: Supplementary file 3 — Supplementary Data 1 [file 41467_2022_34656_MOESM3_ESM.zip › Supplementary data_1/Supplementary_material_1-1 Geometric morphometrics/bgPCA_306/mean_shapes_per_clade_bgPCA/Stem_Ruminantia-dl.png]

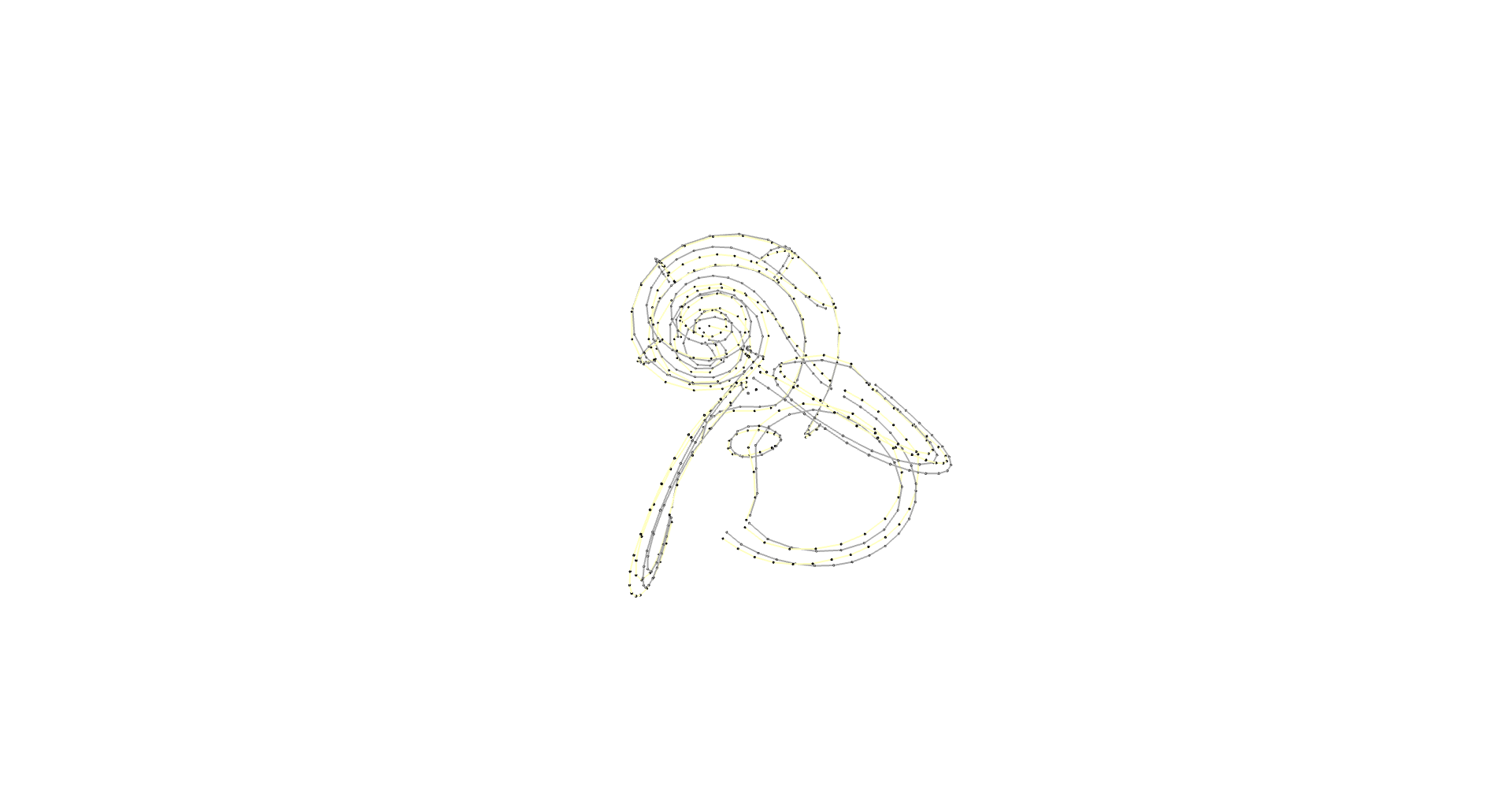

Supplement: Supplementary file 3 — Supplementary Data 1 [file 41467_2022_34656_MOESM3_ESM.zip › Supplementary data_1/Supplementary_material_1-1 Geometric morphometrics/bgPCA_306/mean_shapes_per_clade_bgPCA/Stem_Ruminantia-do.png]

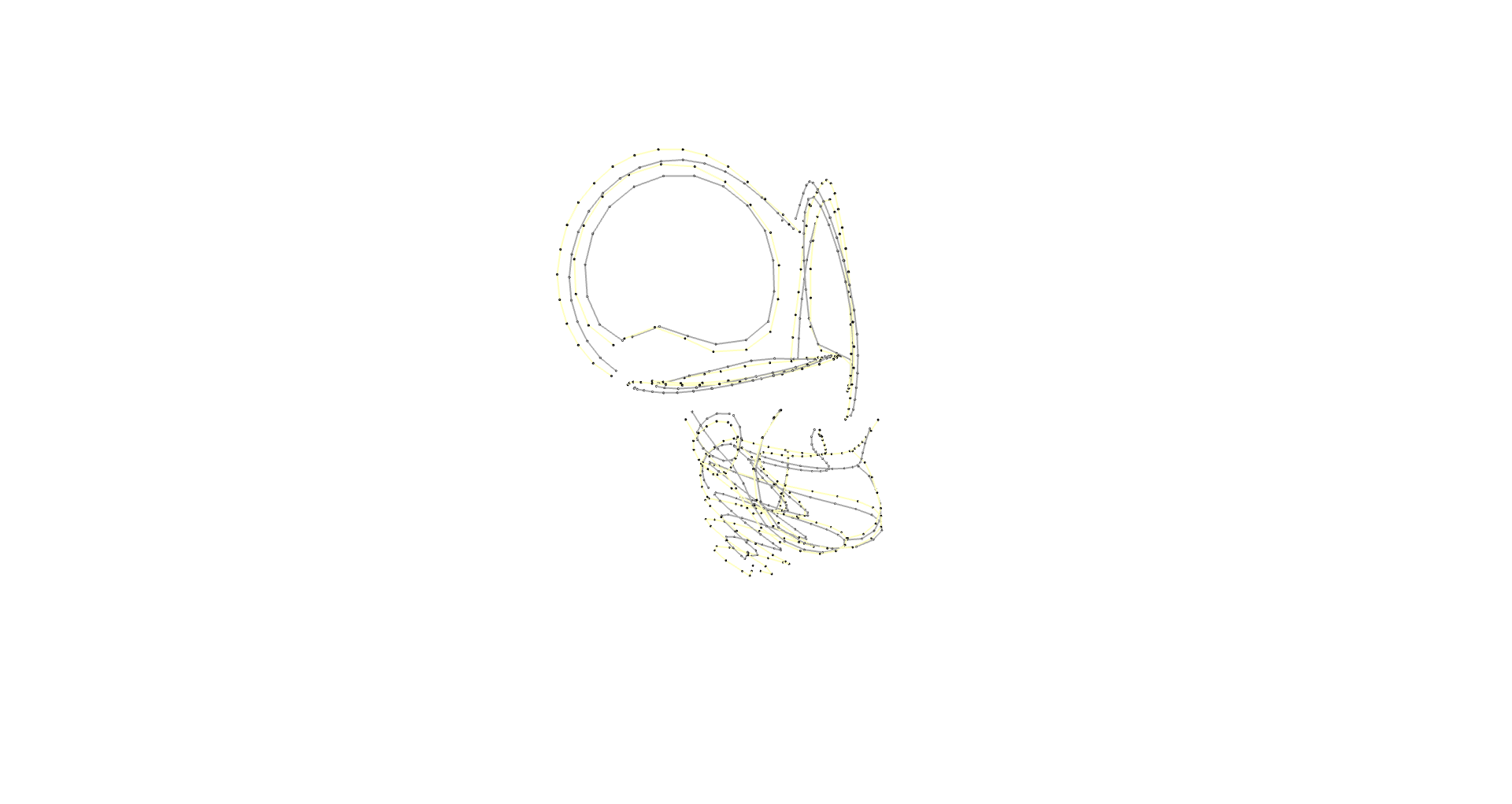

Supplement: Supplementary file 3 — Supplementary Data 1 [file 41467_2022_34656_MOESM3_ESM.zip › Supplementary data_1/Supplementary_material_1-1 Geometric morphometrics/bgPCA_306/mean_shapes_per_clade_bgPCA/Stem_Ruminantia-la.png]

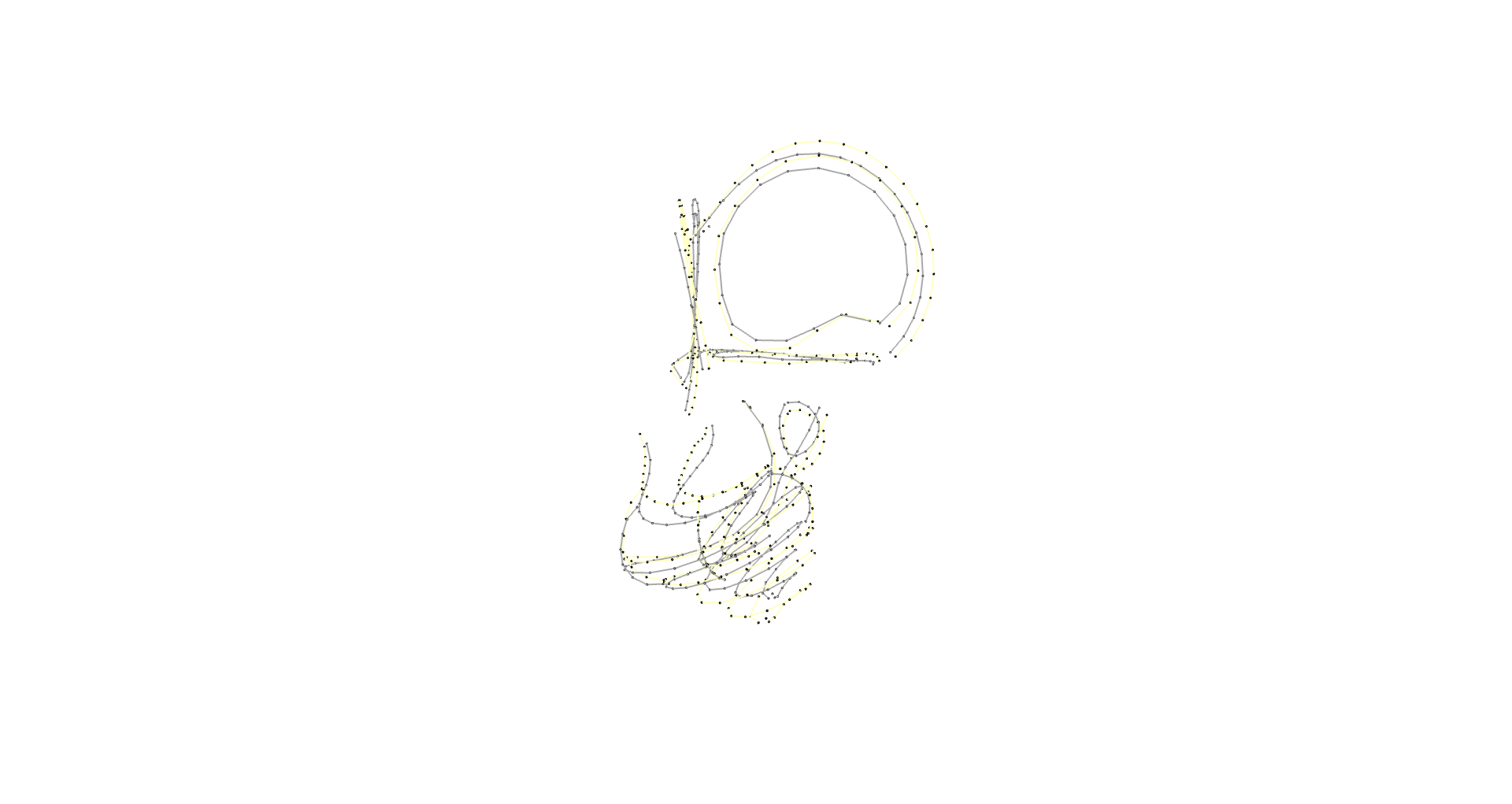

Supplement: Supplementary file 3 — Supplementary Data 1 [file 41467_2022_34656_MOESM3_ESM.zip › Supplementary data_1/Supplementary_material_1-1 Geometric morphometrics/bgPCA_306/mean_shapes_per_clade_bgPCA/Stem_Ruminantia-me.png]

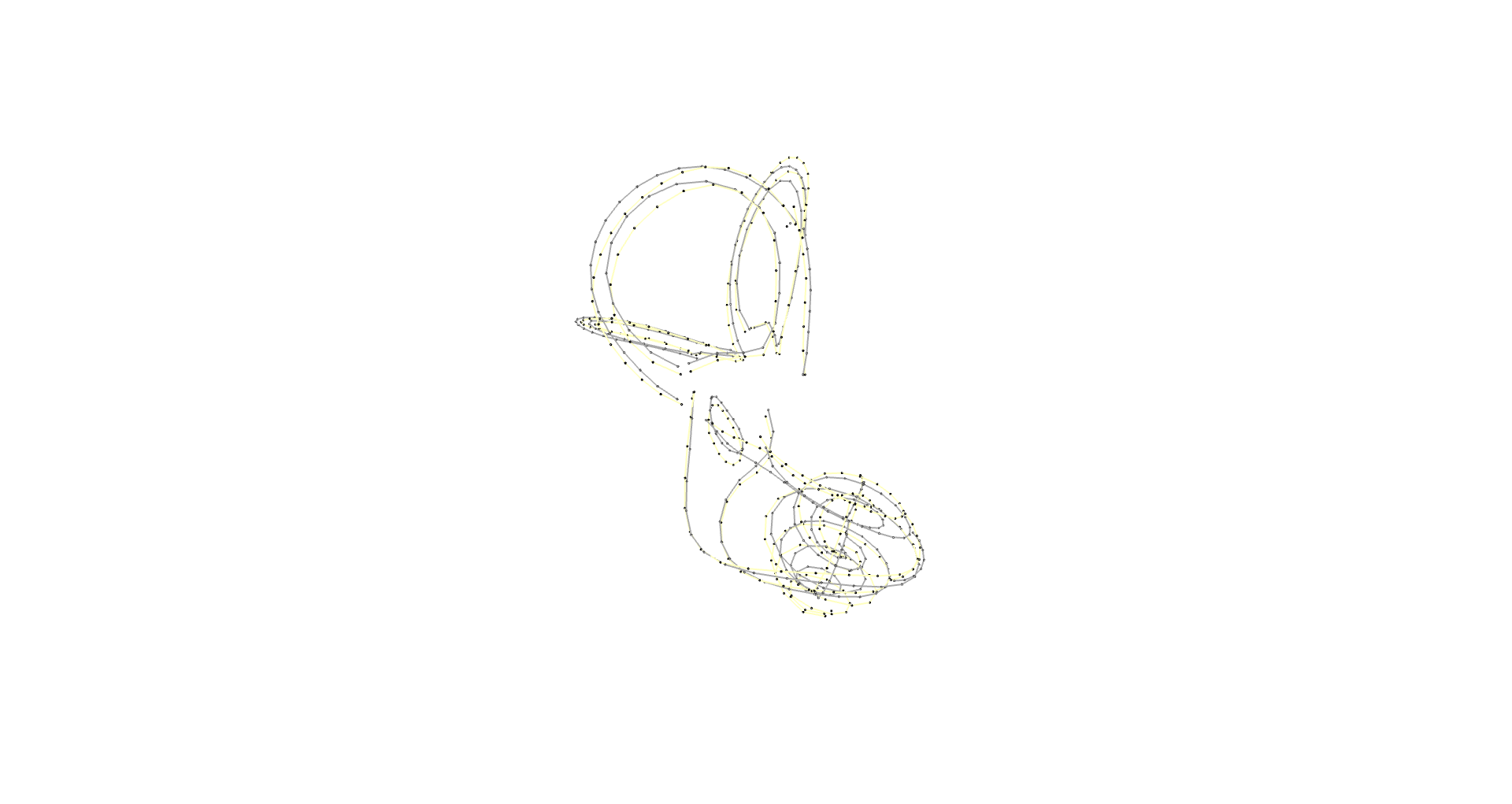

Supplement: Supplementary file 3 — Supplementary Data 1 [file 41467_2022_34656_MOESM3_ESM.zip › Supplementary data_1/Supplementary_material_1-1 Geometric morphometrics/bgPCA_306/mean_shapes_per_clade_bgPCA/Stem_Ruminantia-oc.png]

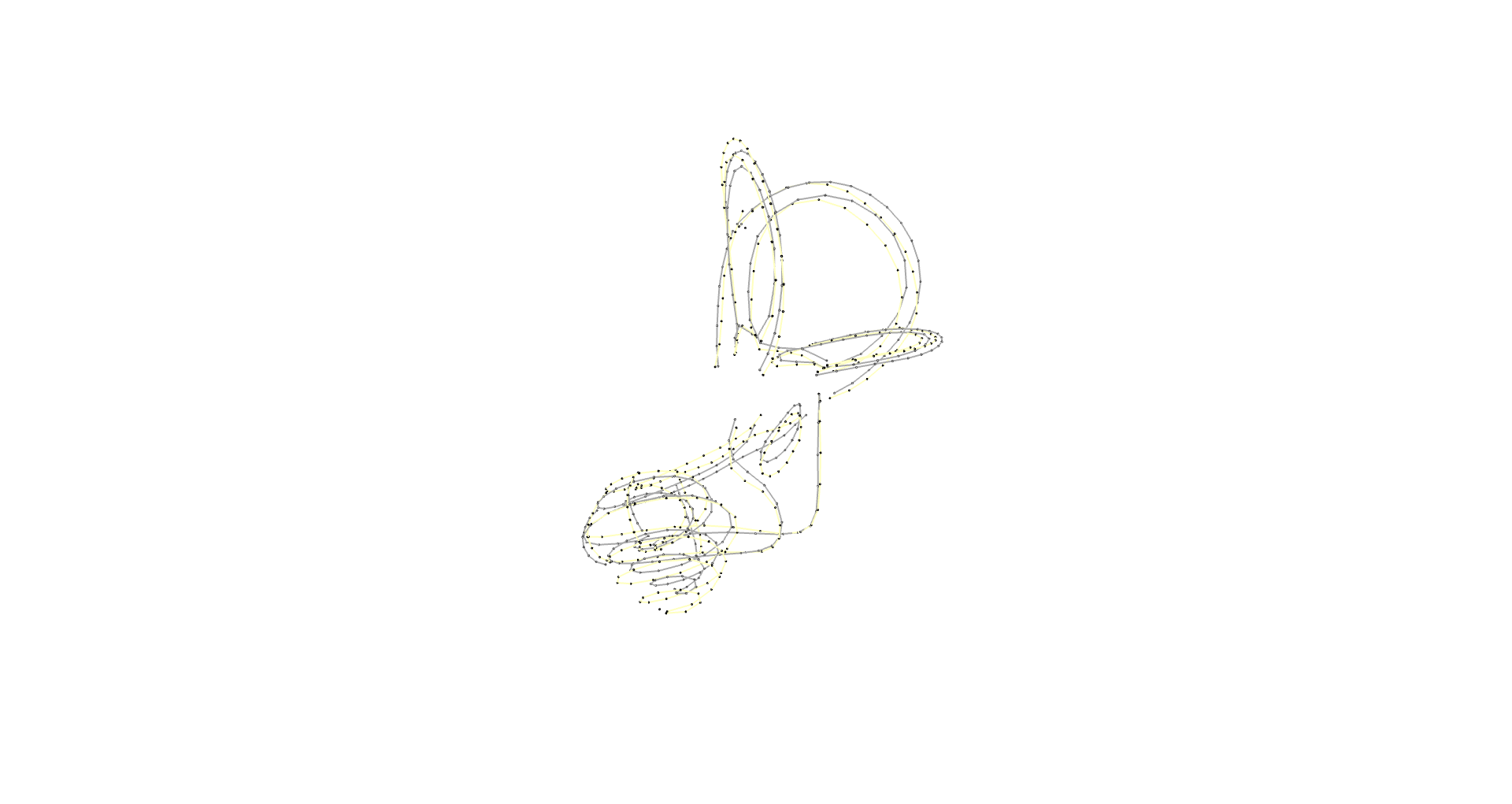

Supplement: Supplementary file 3 — Supplementary Data 1 [file 41467_2022_34656_MOESM3_ESM.zip › Supplementary data_1/Supplementary_material_1-1 Geometric morphometrics/bgPCA_306/mean_shapes_per_clade_bgPCA/Stem_Ruminantia-ro.png]

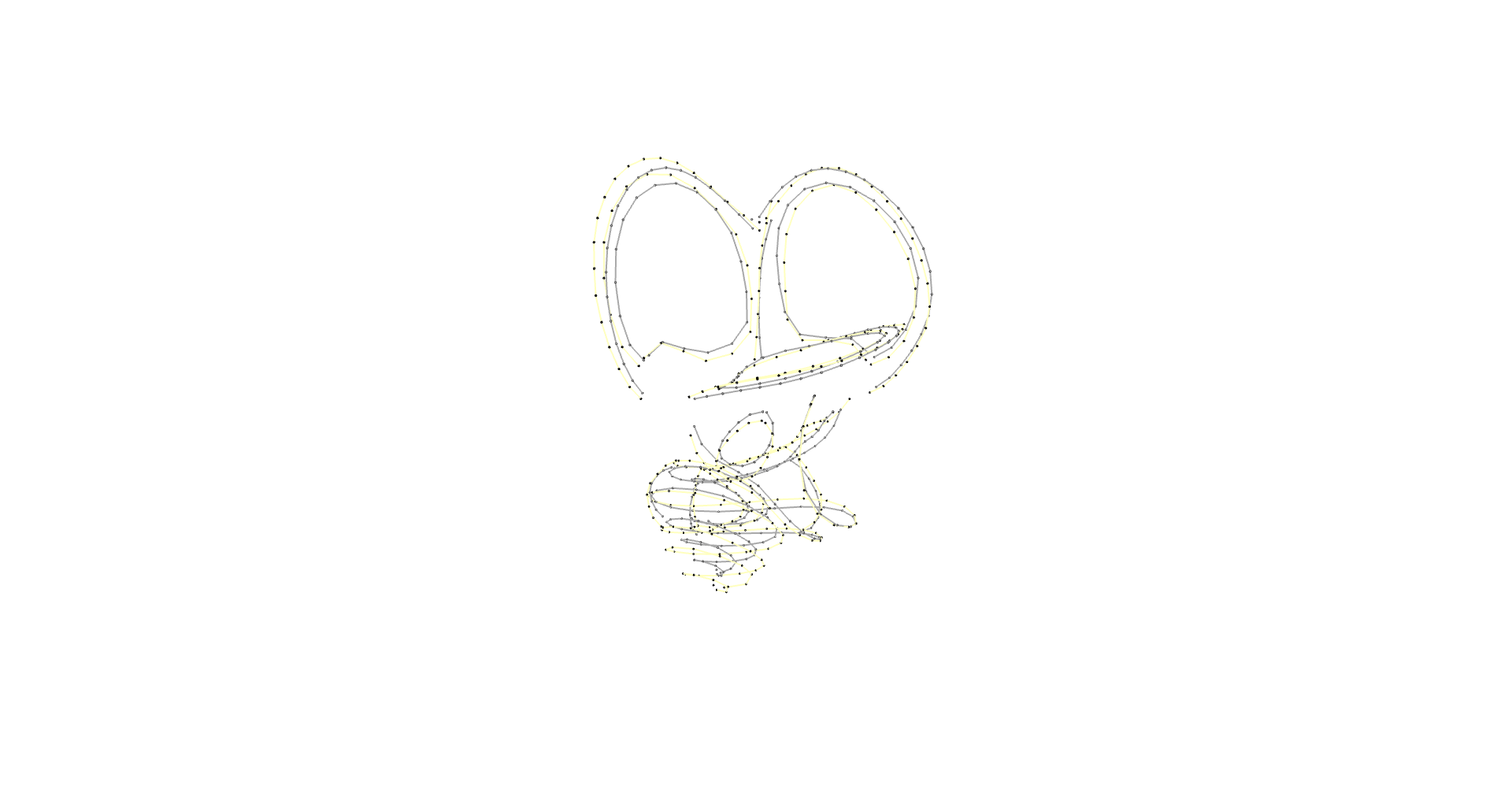

Supplement: Supplementary file 3 — Supplementary Data 1 [file 41467_2022_34656_MOESM3_ESM.zip › Supplementary data_1/Supplementary_material_1-1 Geometric morphometrics/bgPCA_306/mean_shapes_per_clade_bgPCA/Stem_Ruminantia-vl.png]

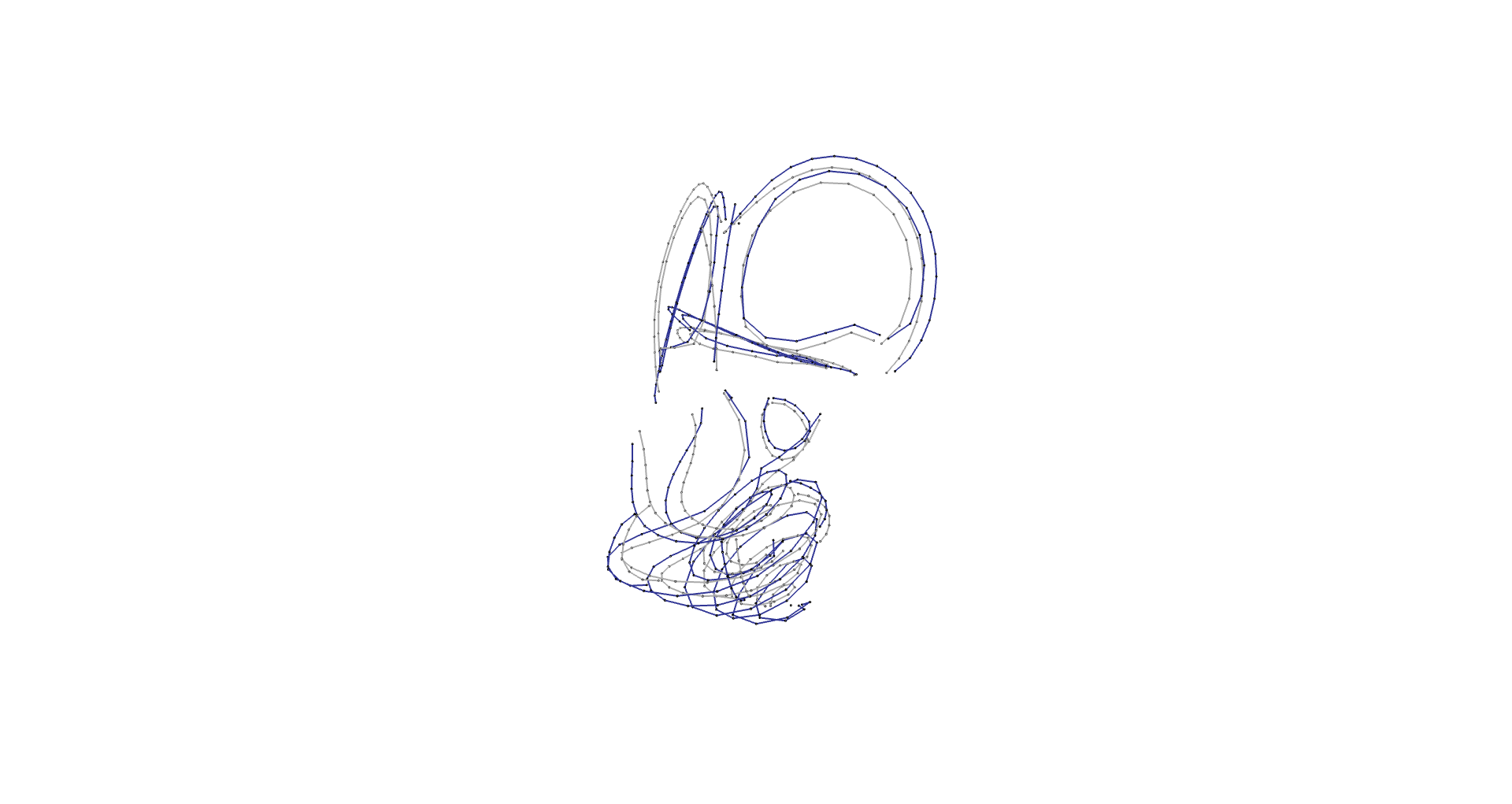

Supplement: Supplementary file 3 — Supplementary Data 1 [file 41467_2022_34656_MOESM3_ESM.zip › Supplementary data_1/Supplementary_material_1-1 Geometric morphometrics/bgPCA_306/mean_shapes_per_clade_bgPCA/Tragulidae-dl.png]

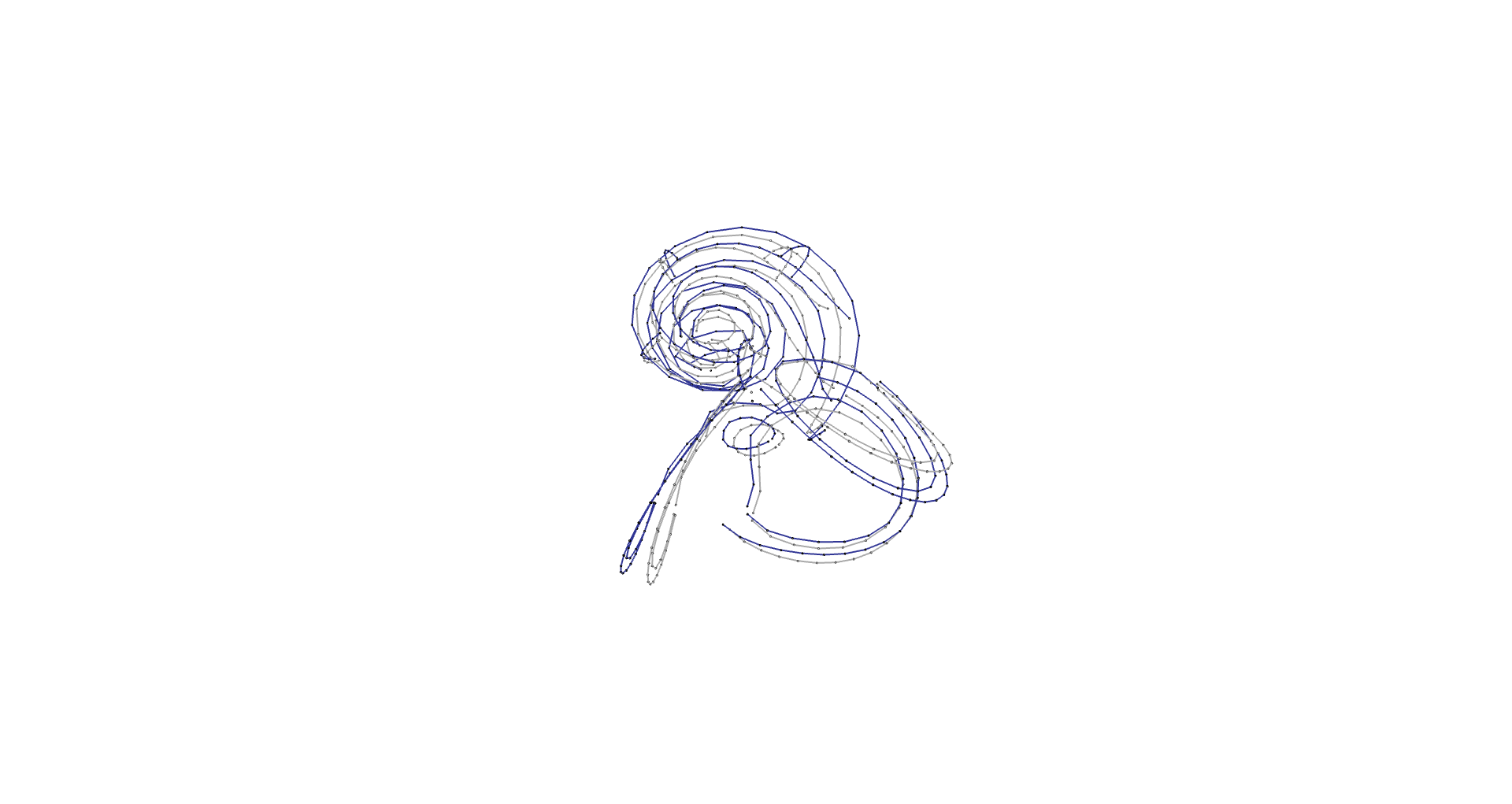

Supplement: Supplementary file 3 — Supplementary Data 1 [file 41467_2022_34656_MOESM3_ESM.zip › Supplementary data_1/Supplementary_material_1-1 Geometric morphometrics/bgPCA_306/mean_shapes_per_clade_bgPCA/Tragulidae-do.png]

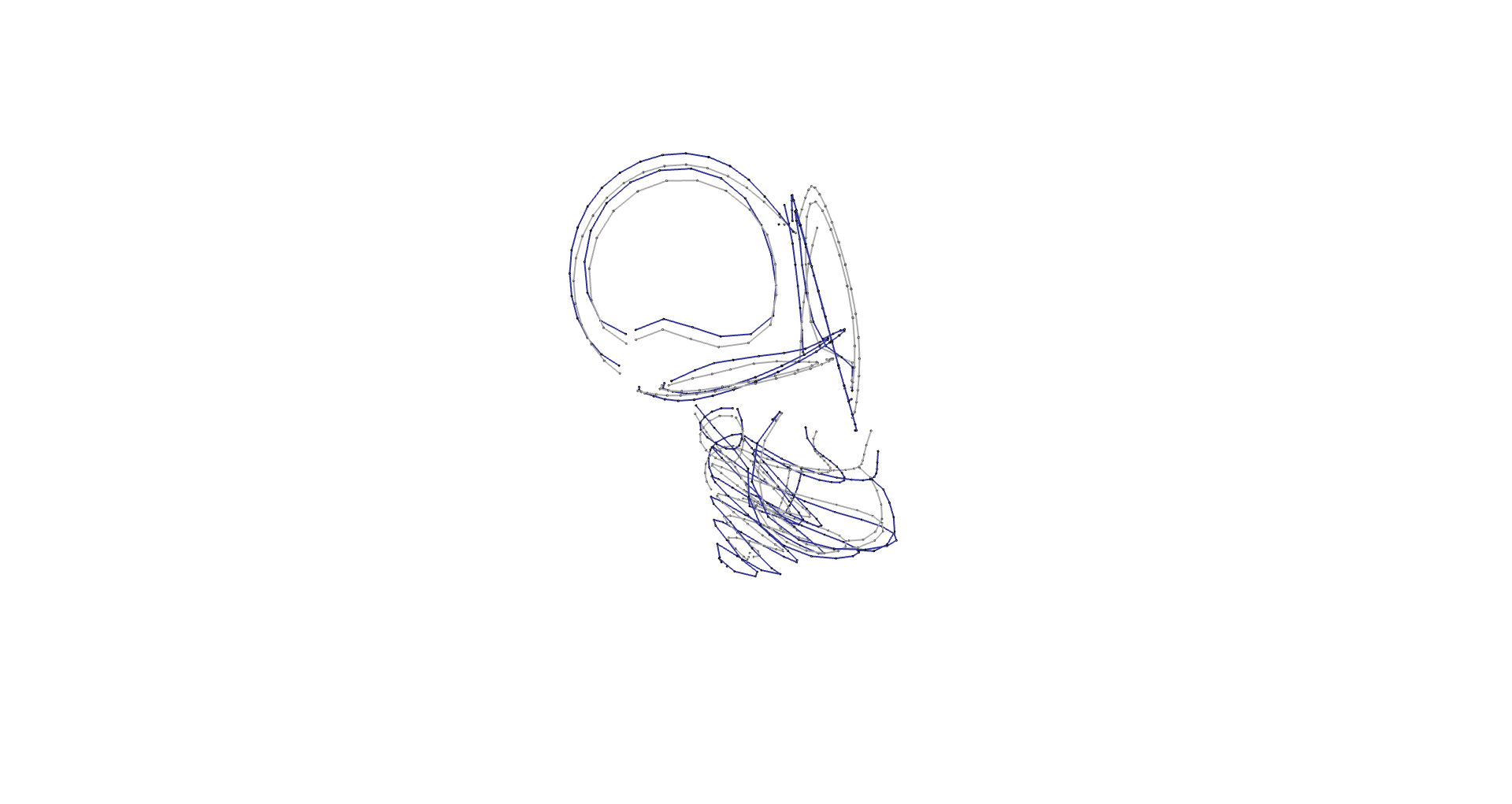

Supplement: Supplementary file 3 — Supplementary Data 1 [file 41467_2022_34656_MOESM3_ESM.zip › Supplementary data_1/Supplementary_material_1-1 Geometric morphometrics/bgPCA_306/mean_shapes_per_clade_bgPCA/Tragulidae-la.png]

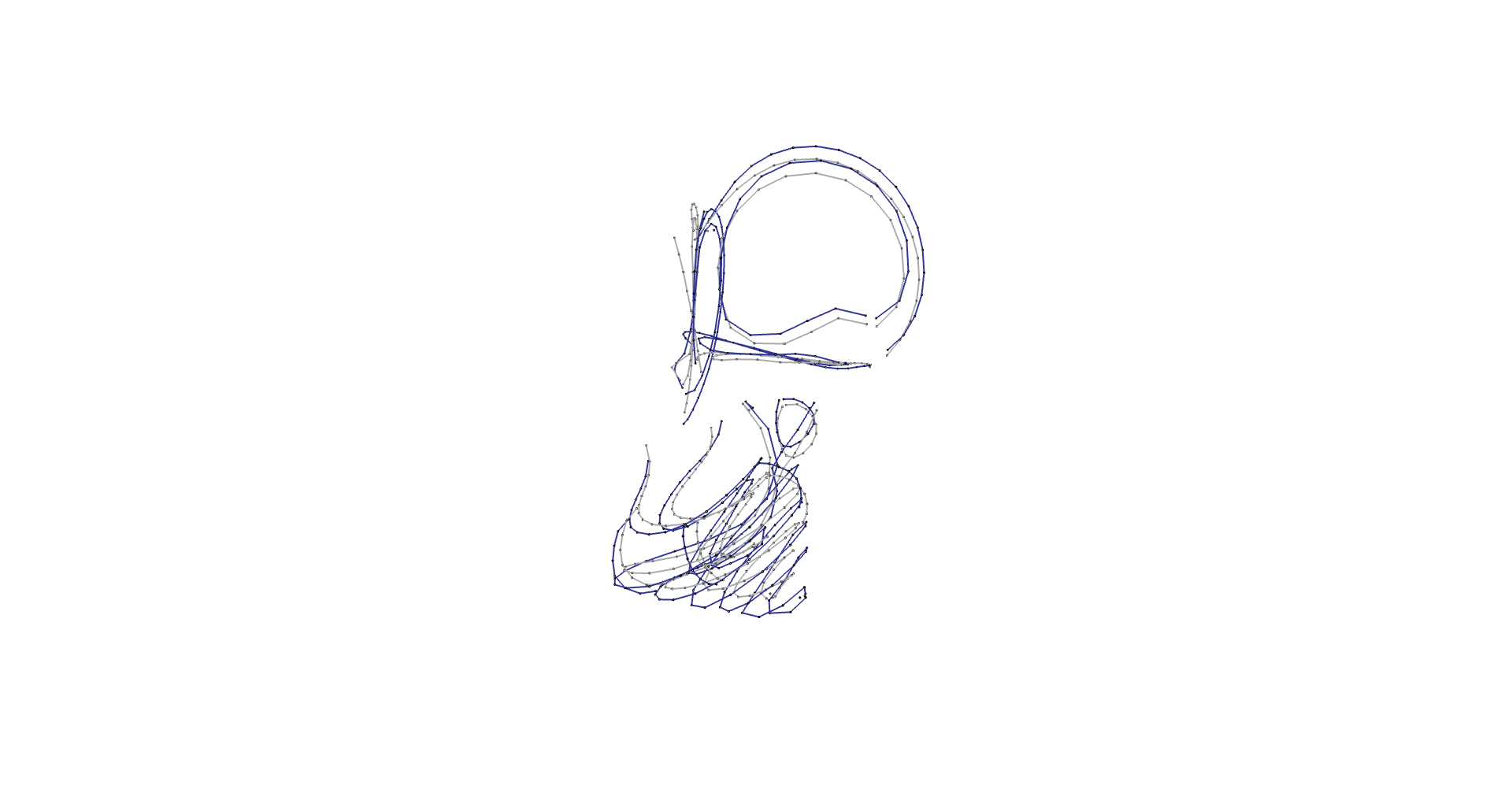

Supplement: Supplementary file 3 — Supplementary Data 1 [file 41467_2022_34656_MOESM3_ESM.zip › Supplementary data_1/Supplementary_material_1-1 Geometric morphometrics/bgPCA_306/mean_shapes_per_clade_bgPCA/Tragulidae-me.png]

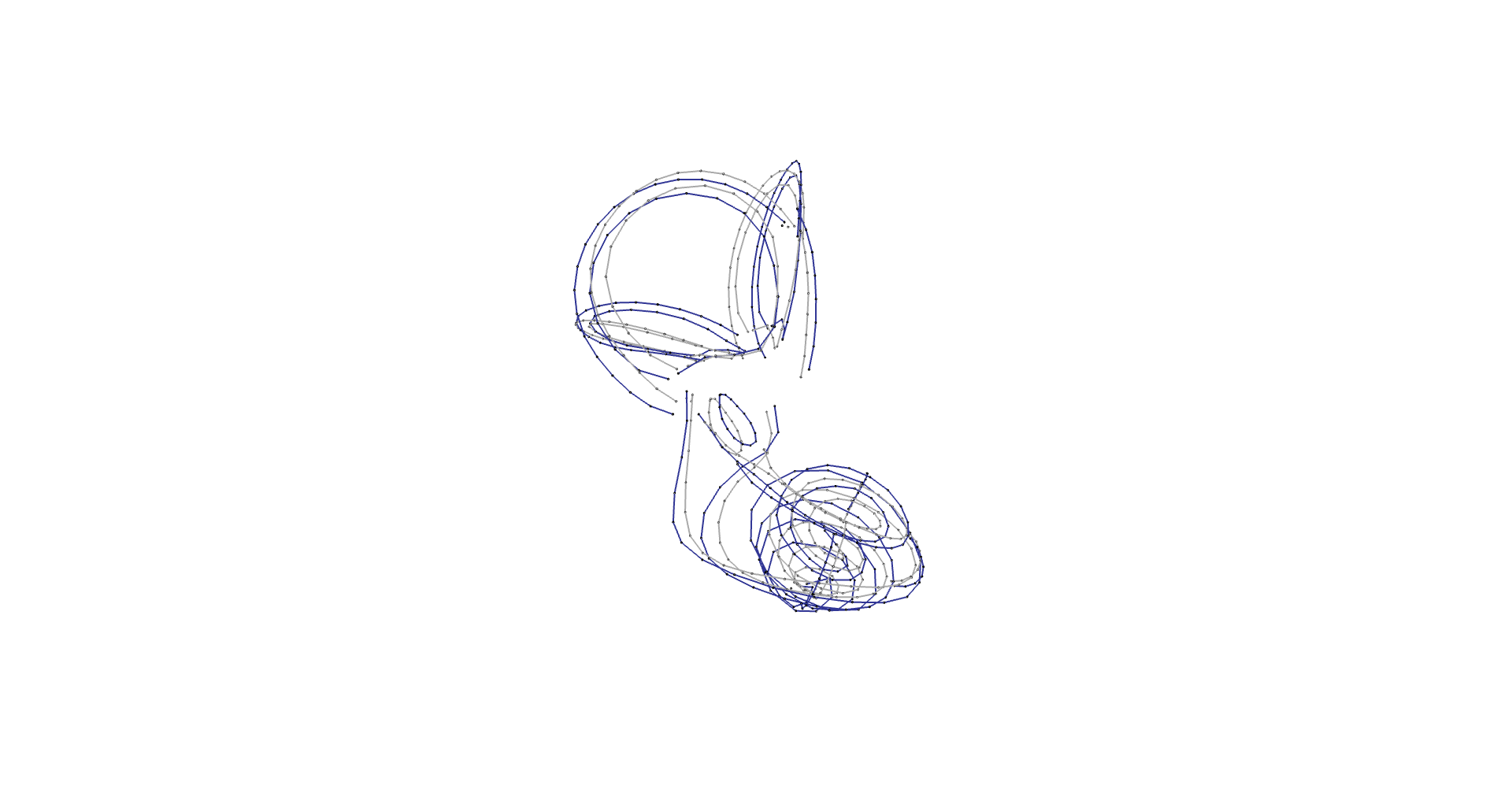

Supplement: Supplementary file 3 — Supplementary Data 1 [file 41467_2022_34656_MOESM3_ESM.zip › Supplementary data_1/Supplementary_material_1-1 Geometric morphometrics/bgPCA_306/mean_shapes_per_clade_bgPCA/Tragulidae-oc.png]

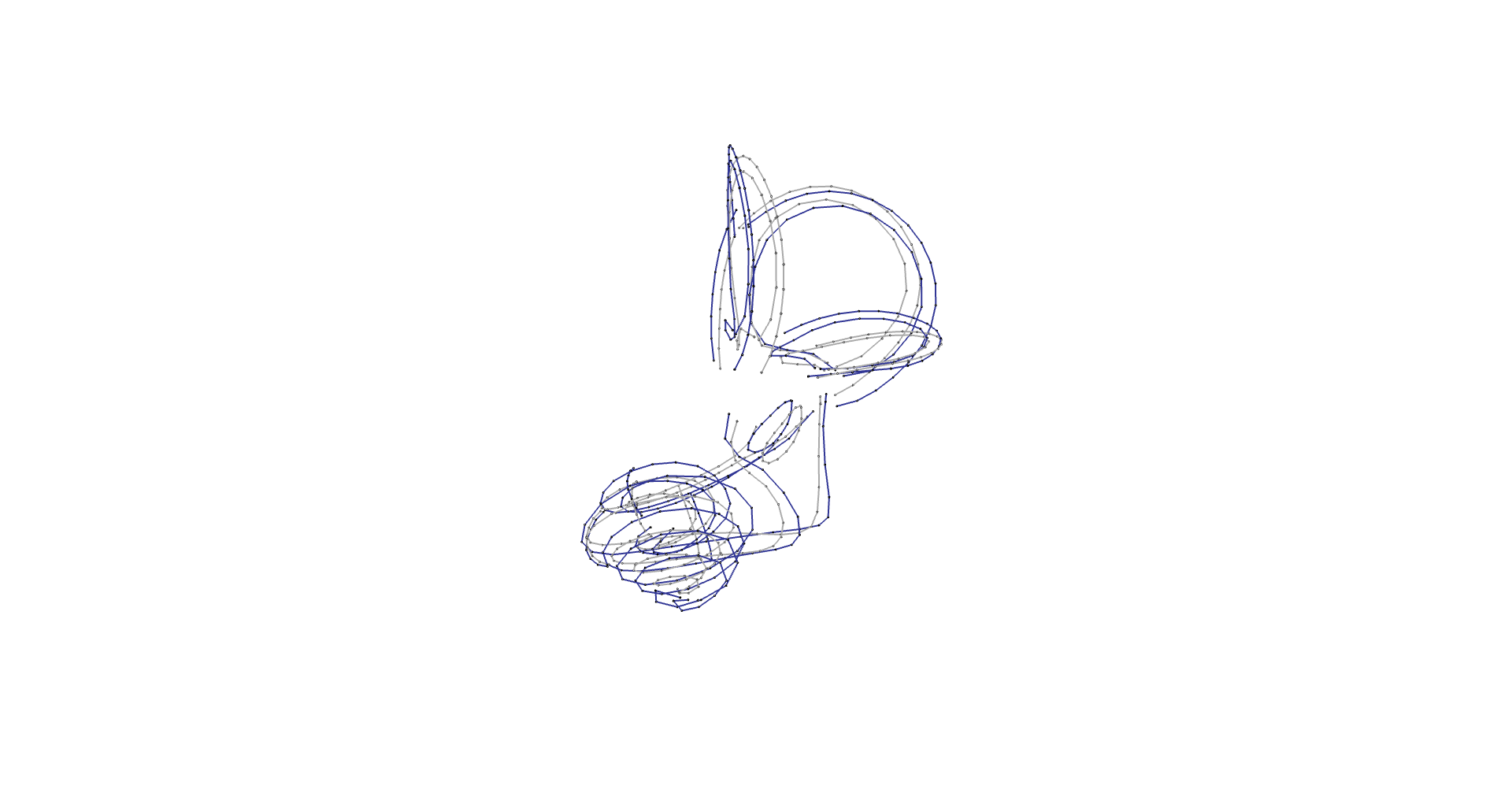

Supplement: Supplementary file 3 — Supplementary Data 1 [file 41467_2022_34656_MOESM3_ESM.zip › Supplementary data_1/Supplementary_material_1-1 Geometric morphometrics/bgPCA_306/mean_shapes_per_clade_bgPCA/Tragulidae-ro.png]

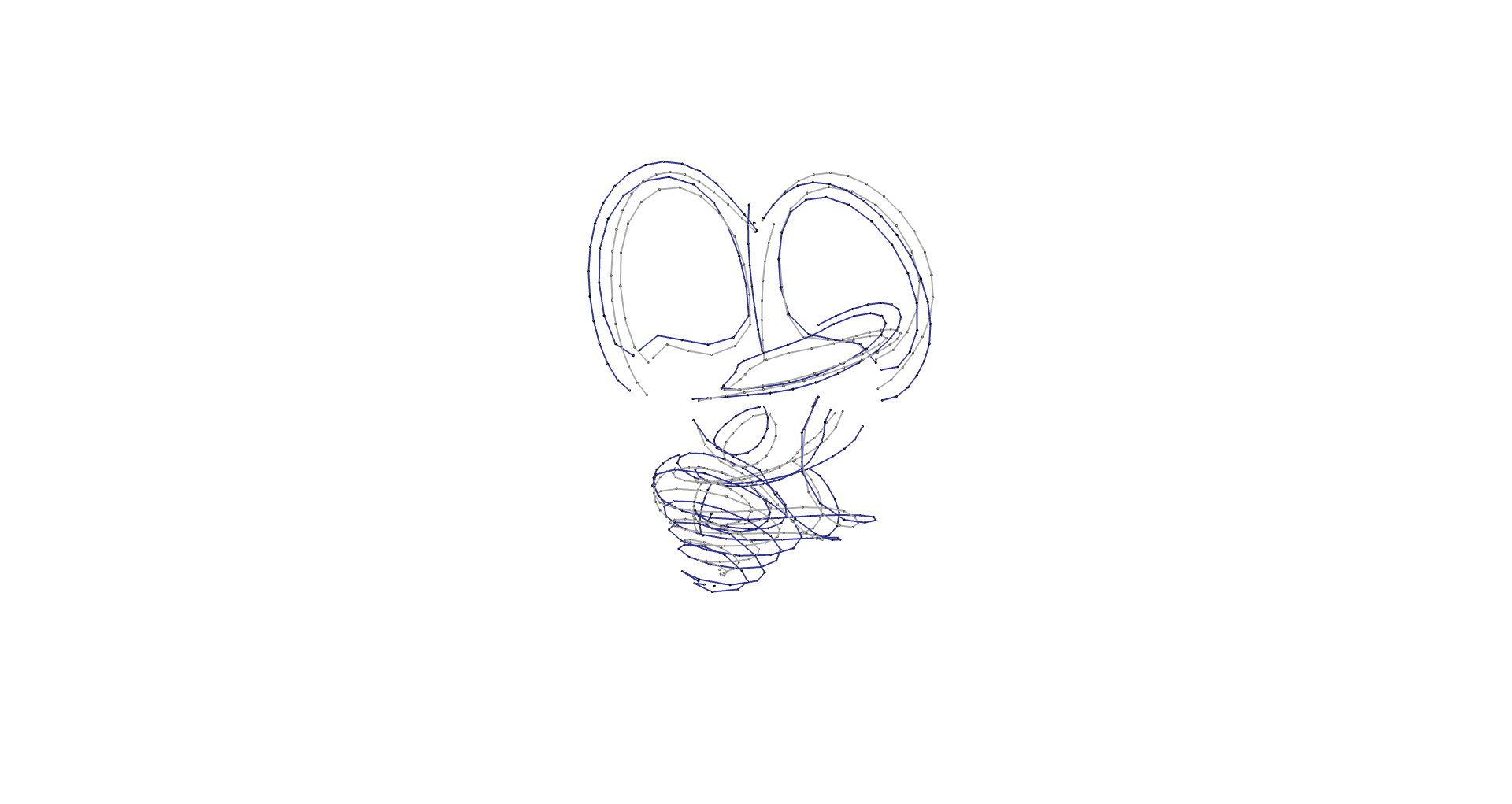

Supplement: Supplementary file 3 — Supplementary Data 1 [file 41467_2022_34656_MOESM3_ESM.zip › Supplementary data_1/Supplementary_material_1-1 Geometric morphometrics/bgPCA_306/mean_shapes_per_clade_bgPCA/Tragulidae-vl.png]

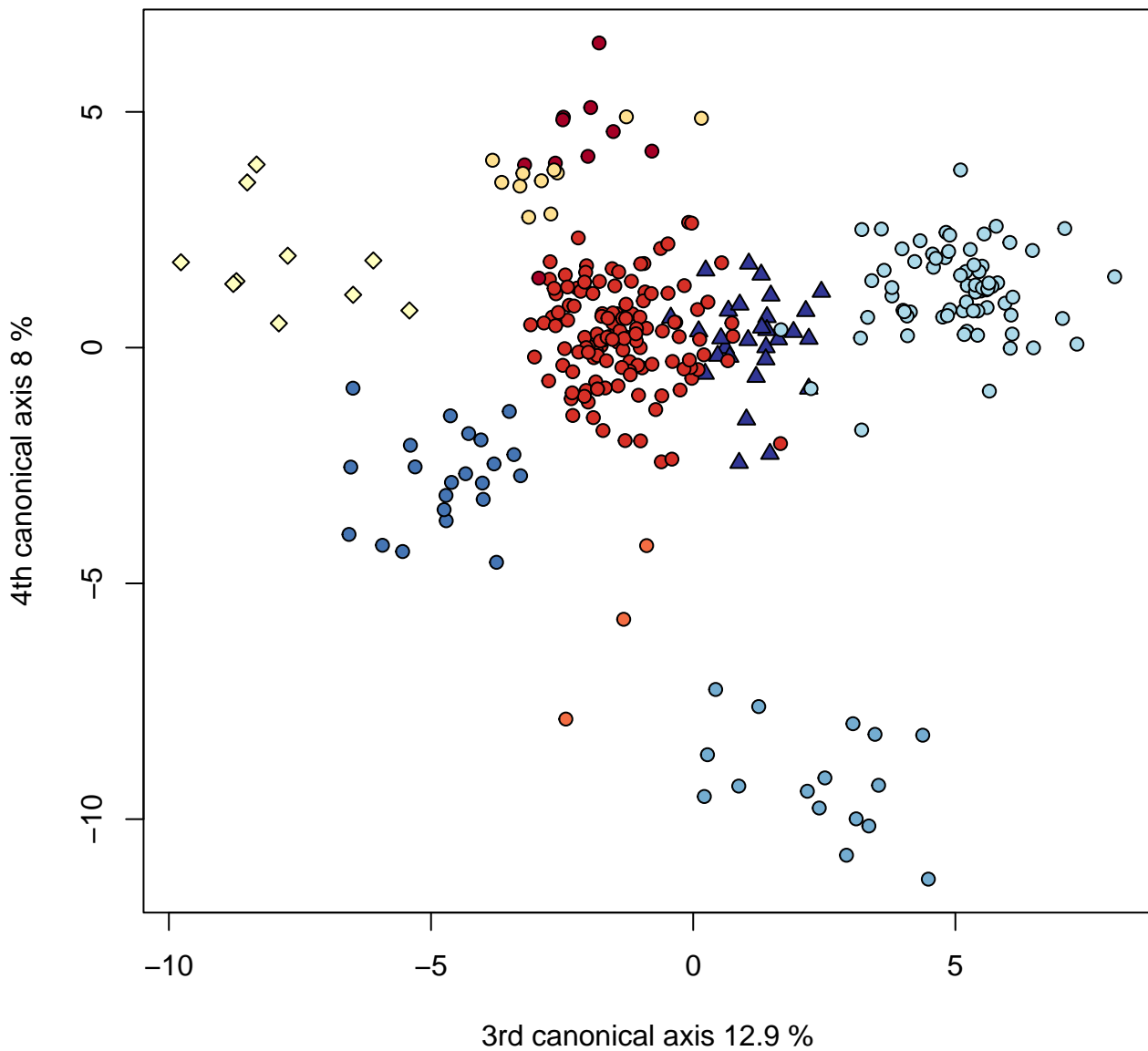

Supplement: Supplementary file 3 — Supplementary Data 1 [file 41467_2022_34656_MOESM3_ESM.zip › Supplementary data_1/Supplementary_material_1-1 Geometric morphometrics/CVA_306/CVA_306_CV3vsCV4_ruminants.pdf]

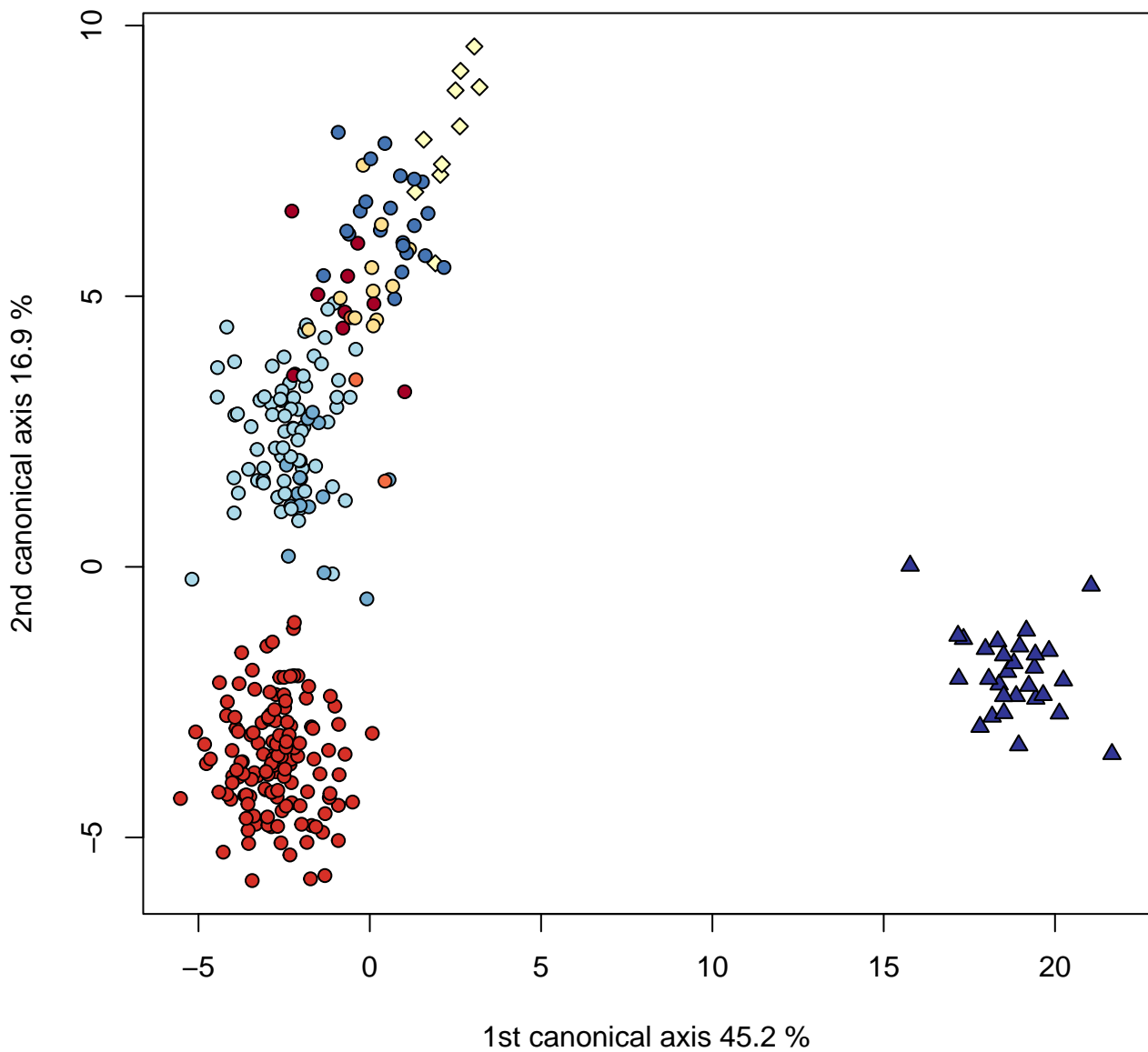

Supplement: Supplementary file 3 — Supplementary Data 1 [file 41467_2022_34656_MOESM3_ESM.zip › Supplementary data_1/Supplementary_material_1-1 Geometric morphometrics/CVA_306/CVA_306_ruminants.pdf]

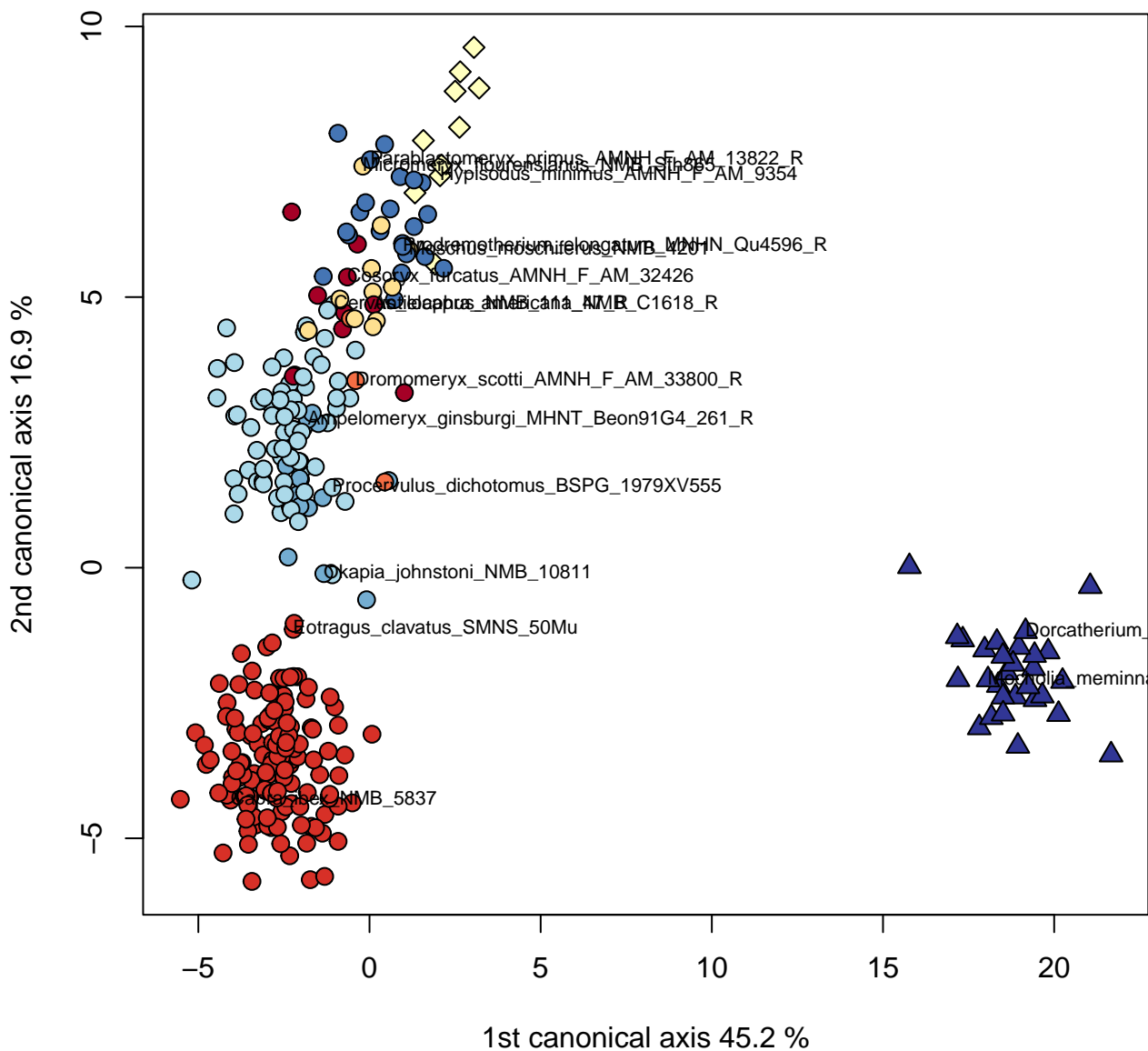

Supplement: Supplementary file 3 — Supplementary Data 1 [file 41467_2022_34656_MOESM3_ESM.zip › Supplementary data_1/Supplementary_material_1-1 Geometric morphometrics/CVA_306/CVA_306_ruminants_names.pdf]

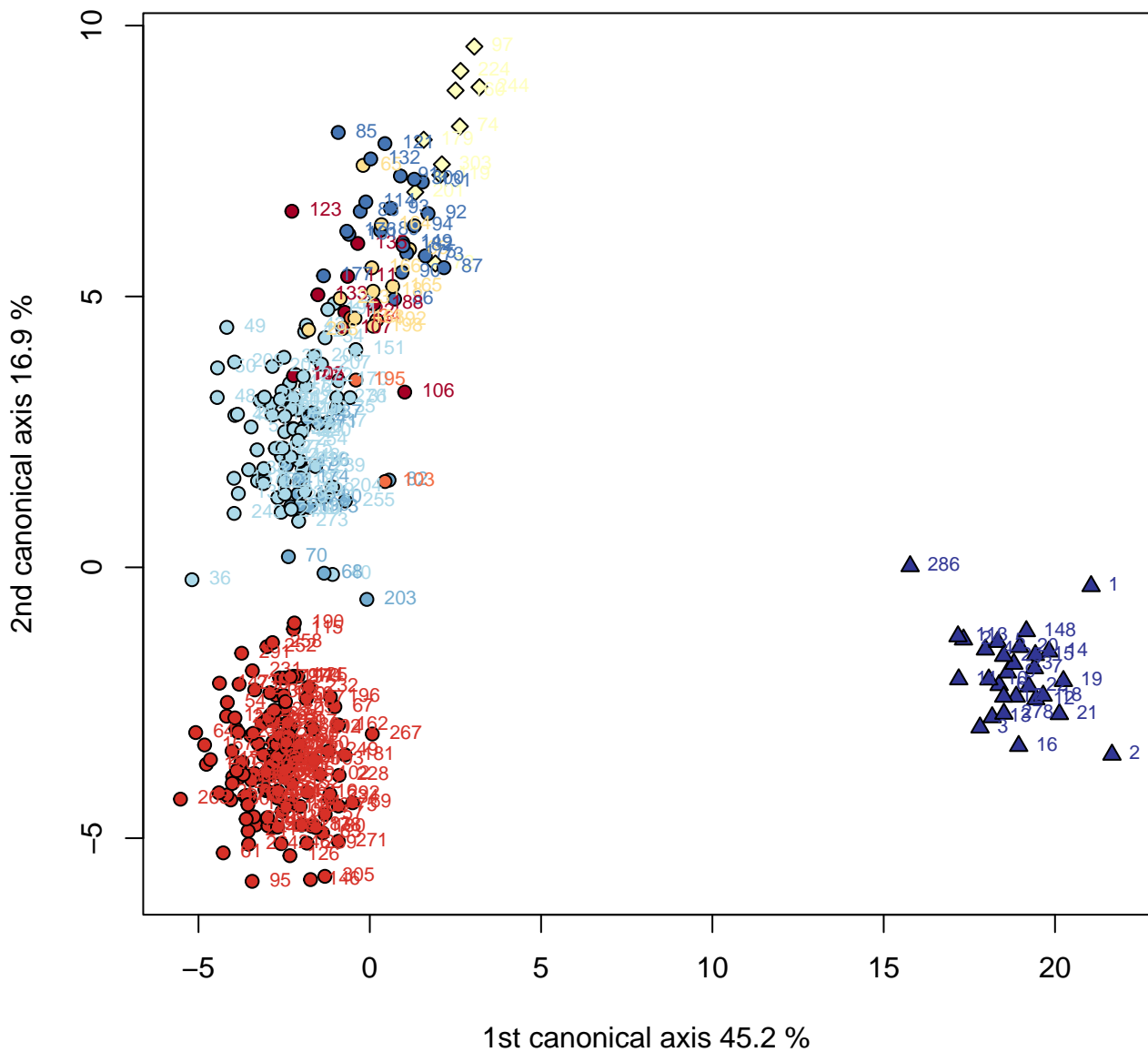

Supplement: Supplementary file 3 — Supplementary Data 1 [file 41467_2022_34656_MOESM3_ESM.zip › Supplementary data_1/Supplementary_material_1-1 Geometric morphometrics/CVA_306/CVA_306_ruminants_numbers.pdf]

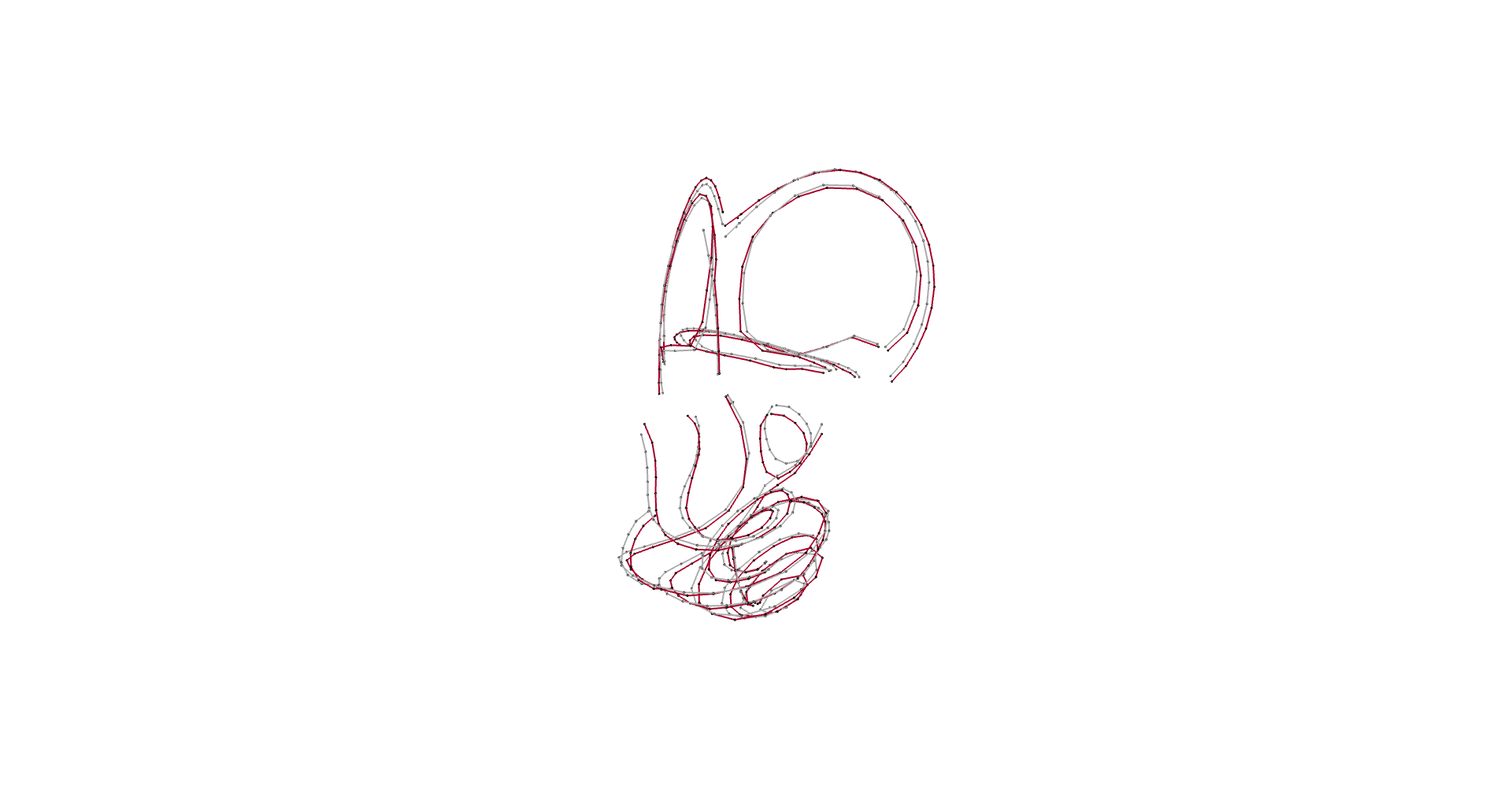

Supplement: Supplementary file 3 — Supplementary Data 1 [file 41467_2022_34656_MOESM3_ESM.zip › Supplementary data_1/Supplementary_material_1-1 Geometric morphometrics/CVA_306/mean_shapes_per_clade_CVA/Antilo-dl.png]

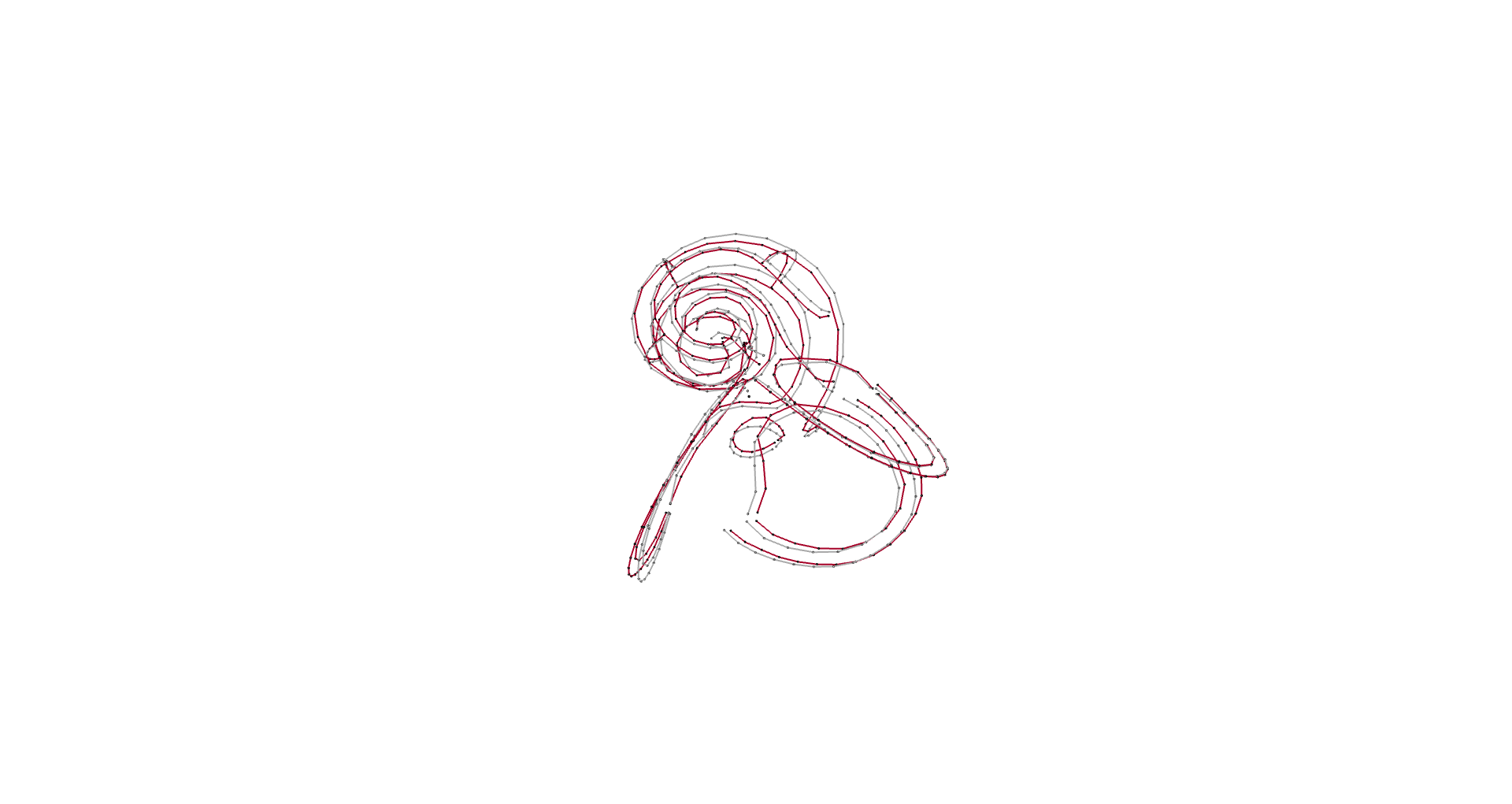

Supplement: Supplementary file 3 — Supplementary Data 1 [file 41467_2022_34656_MOESM3_ESM.zip › Supplementary data_1/Supplementary_material_1-1 Geometric morphometrics/CVA_306/mean_shapes_per_clade_CVA/Antilo-do.png]

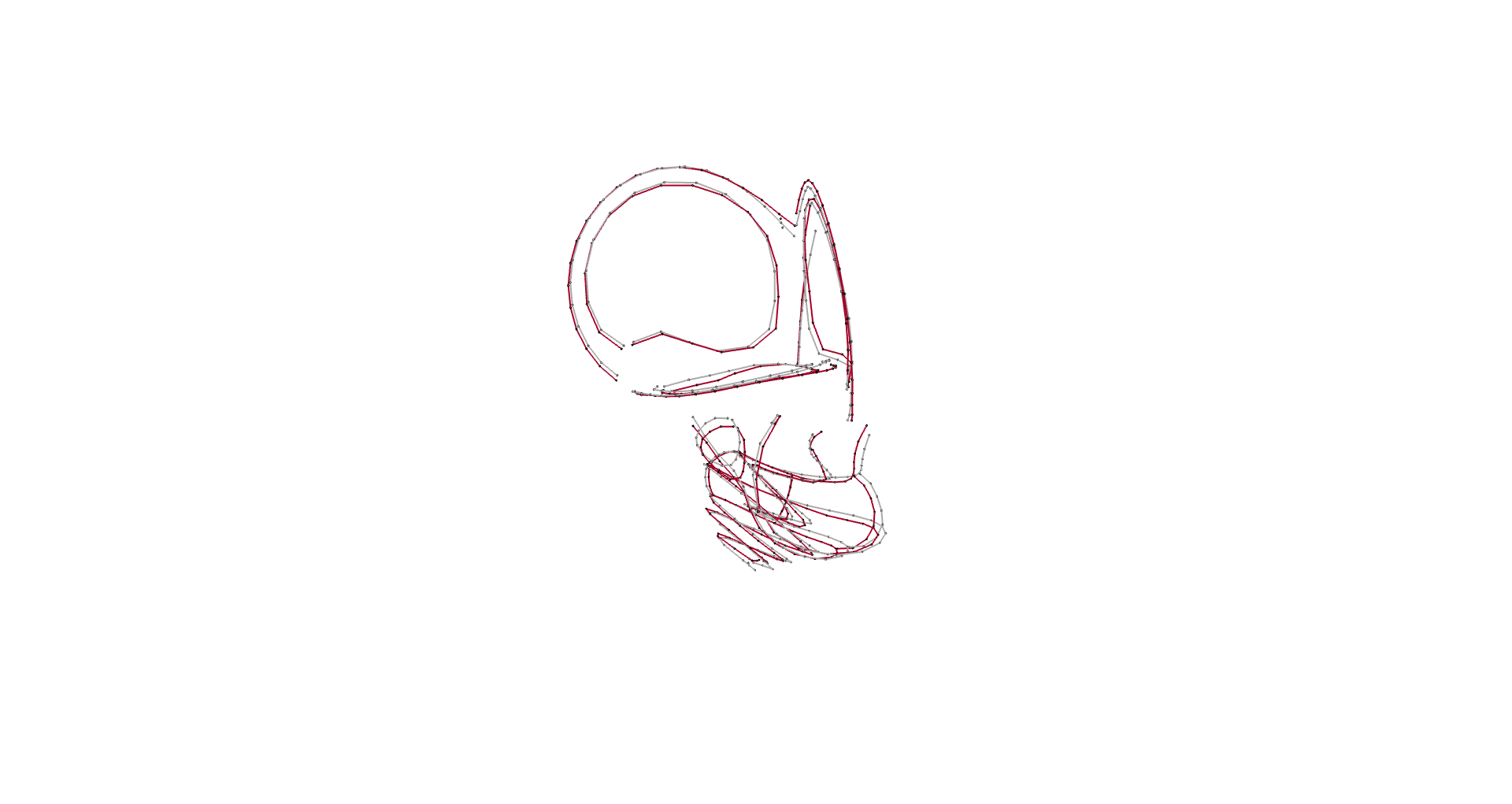

Supplement: Supplementary file 3 — Supplementary Data 1 [file 41467_2022_34656_MOESM3_ESM.zip › Supplementary data_1/Supplementary_material_1-1 Geometric morphometrics/CVA_306/mean_shapes_per_clade_CVA/Antilo-la.png]

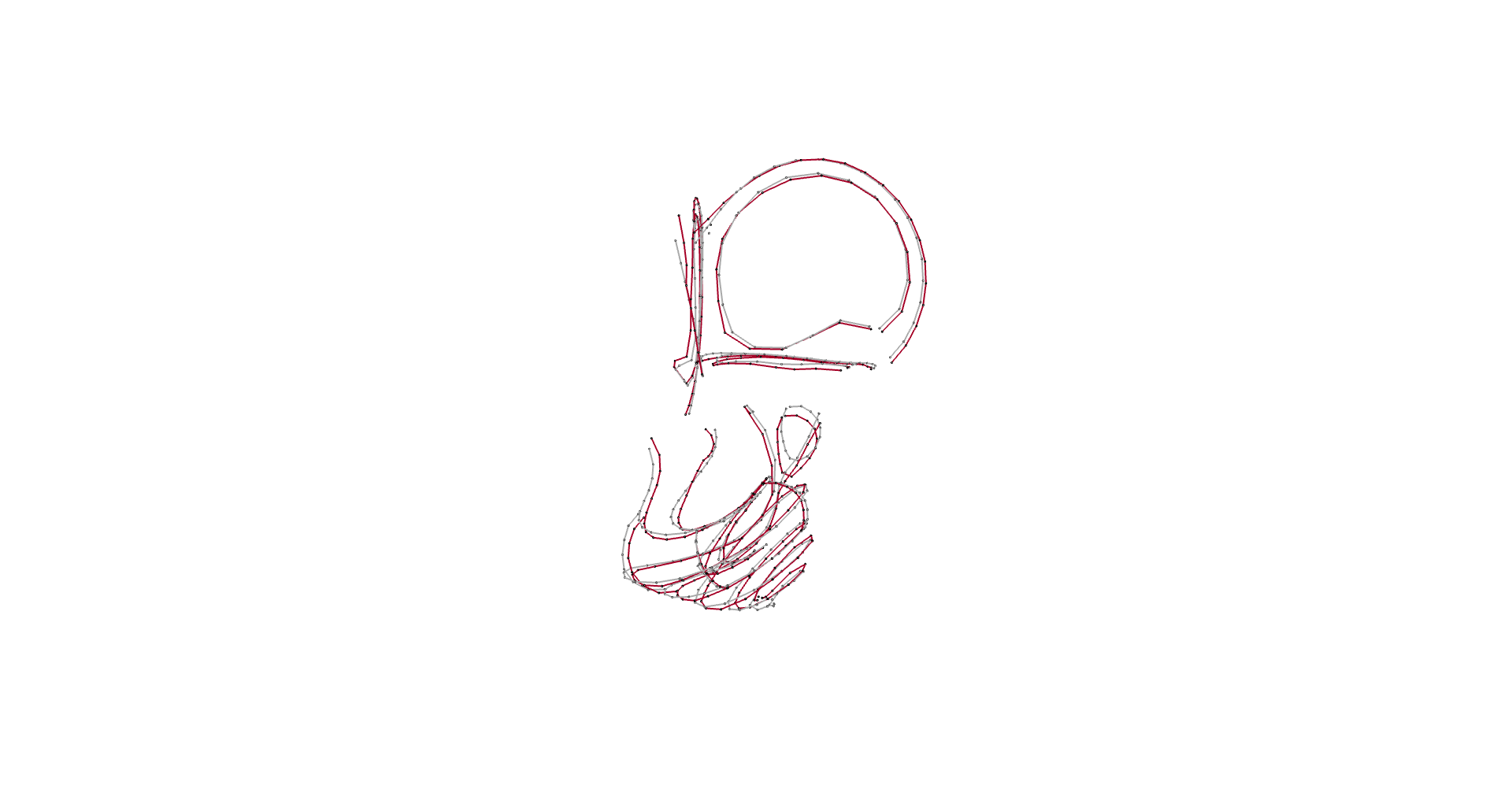

Supplement: Supplementary file 3 — Supplementary Data 1 [file 41467_2022_34656_MOESM3_ESM.zip › Supplementary data_1/Supplementary_material_1-1 Geometric morphometrics/CVA_306/mean_shapes_per_clade_CVA/Antilo-me.png]

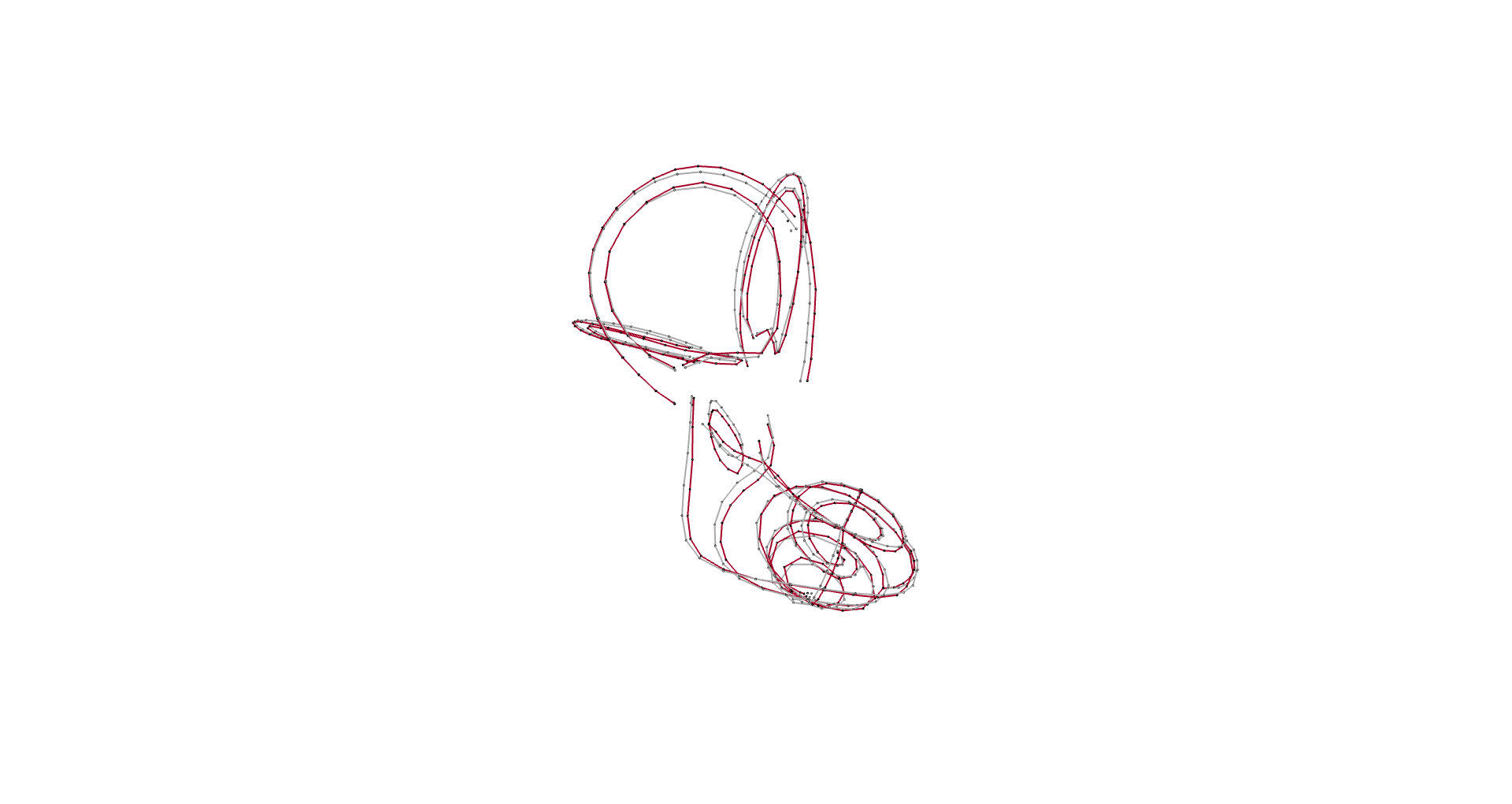

Supplement: Supplementary file 3 — Supplementary Data 1 [file 41467_2022_34656_MOESM3_ESM.zip › Supplementary data_1/Supplementary_material_1-1 Geometric morphometrics/CVA_306/mean_shapes_per_clade_CVA/Antilo-oc.png]

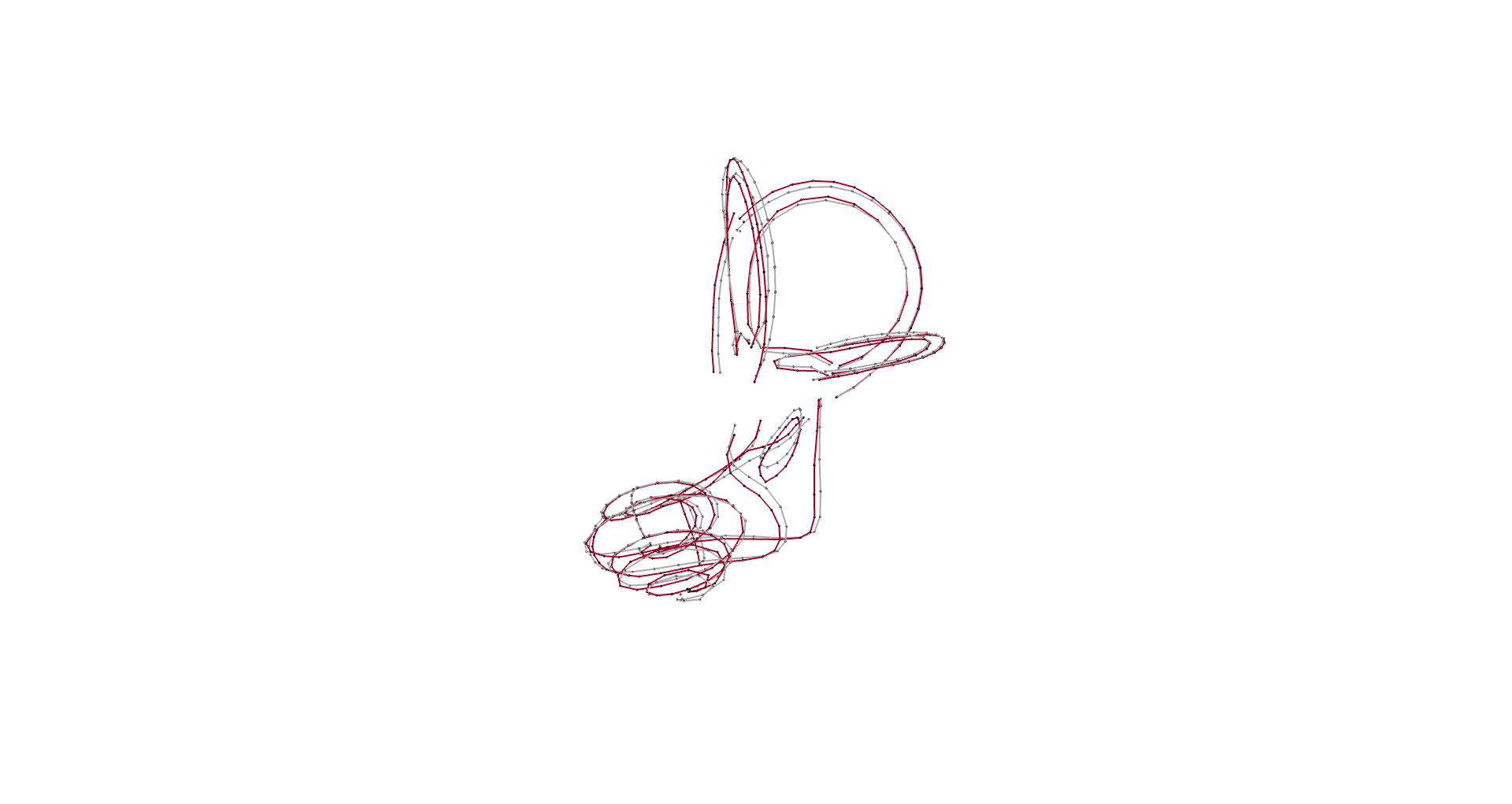

Supplement: Supplementary file 3 — Supplementary Data 1 [file 41467_2022_34656_MOESM3_ESM.zip › Supplementary data_1/Supplementary_material_1-1 Geometric morphometrics/CVA_306/mean_shapes_per_clade_CVA/Antilo-ro.png]

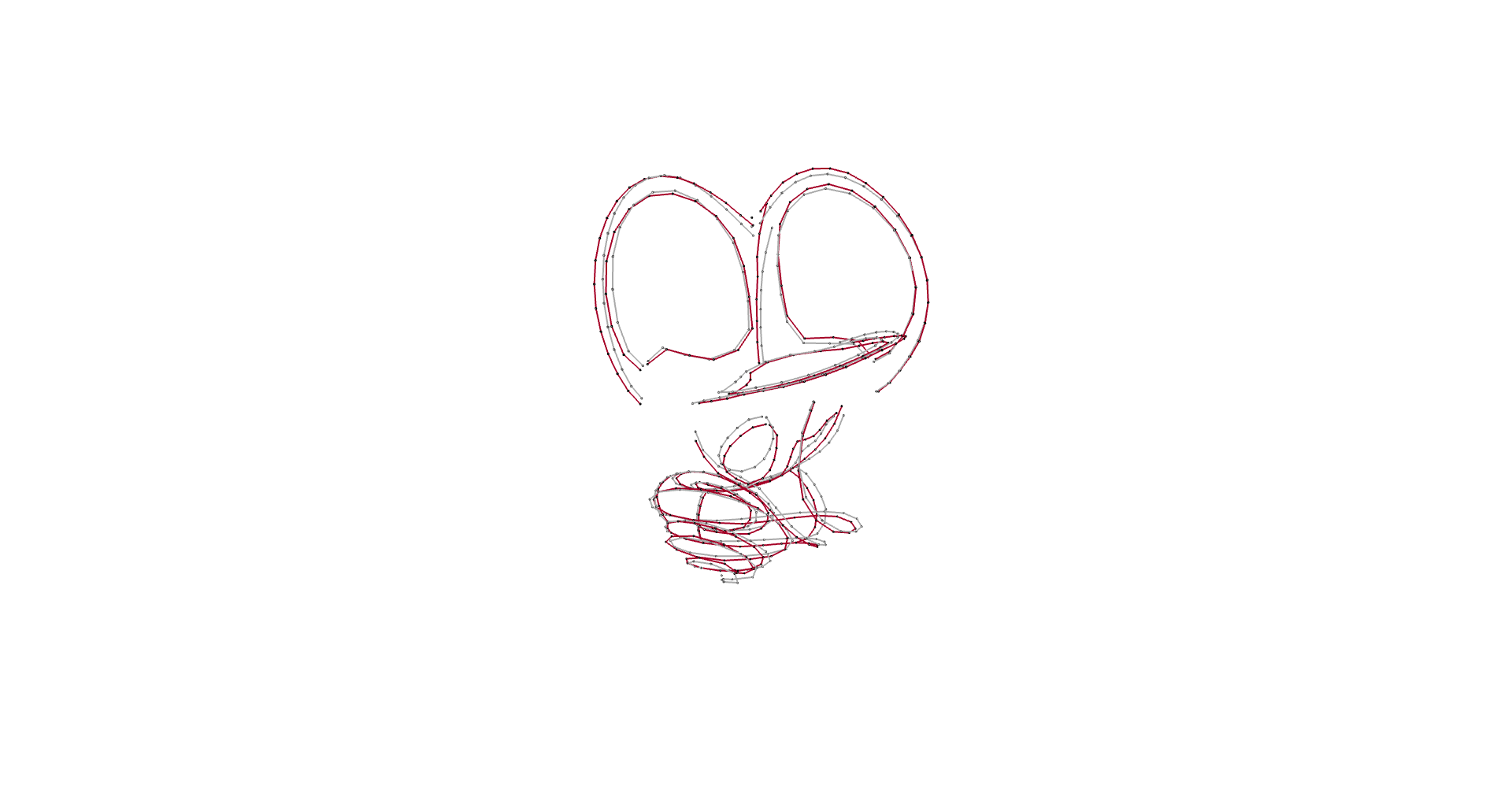

Supplement: Supplementary file 3 — Supplementary Data 1 [file 41467_2022_34656_MOESM3_ESM.zip › Supplementary data_1/Supplementary_material_1-1 Geometric morphometrics/CVA_306/mean_shapes_per_clade_CVA/Antilo-vl.png]

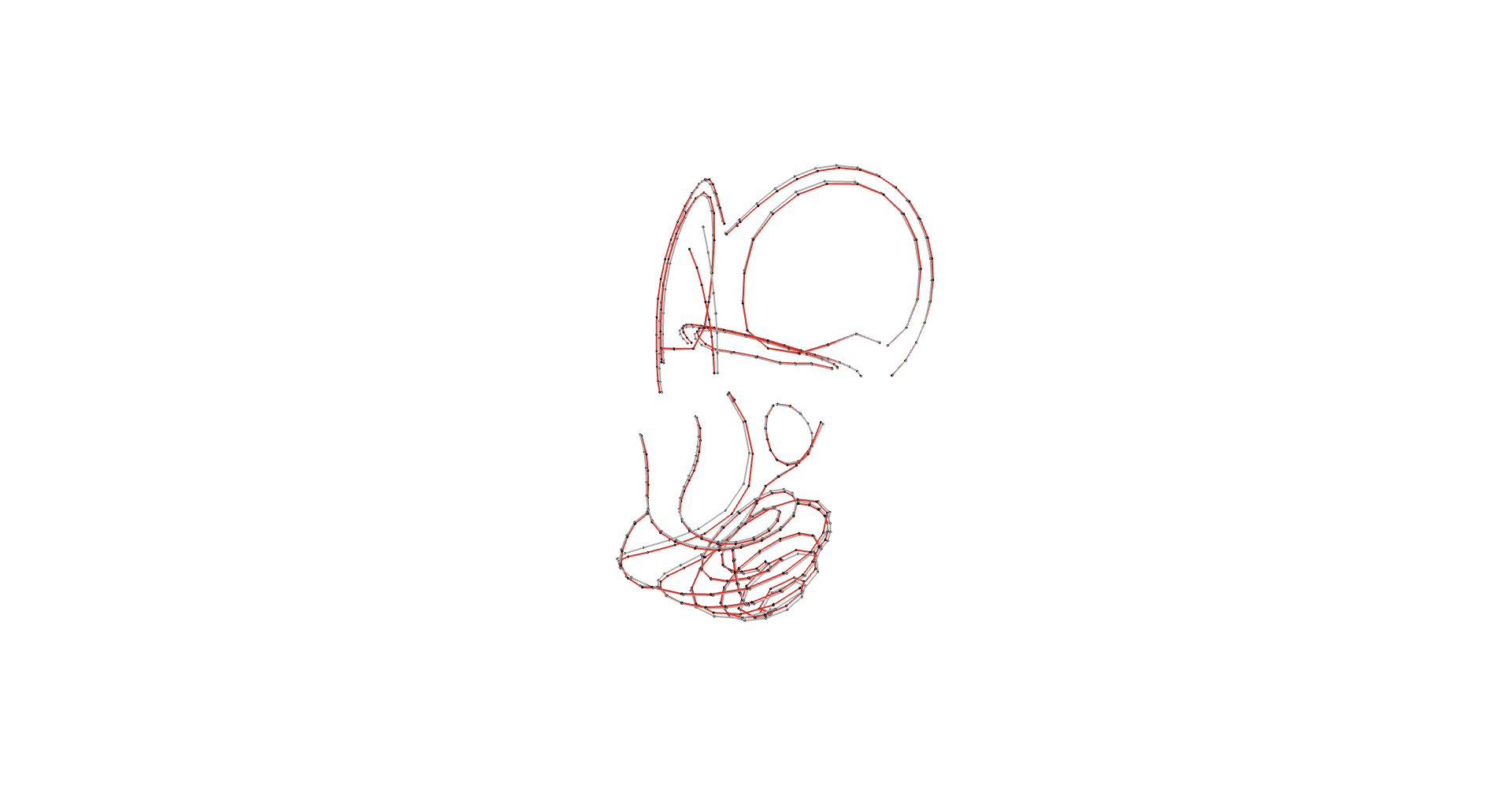

Supplement: Supplementary file 3 — Supplementary Data 1 [file 41467_2022_34656_MOESM3_ESM.zip › Supplementary data_1/Supplementary_material_1-1 Geometric morphometrics/CVA_306/mean_shapes_per_clade_CVA/Bovidae-dl.png]

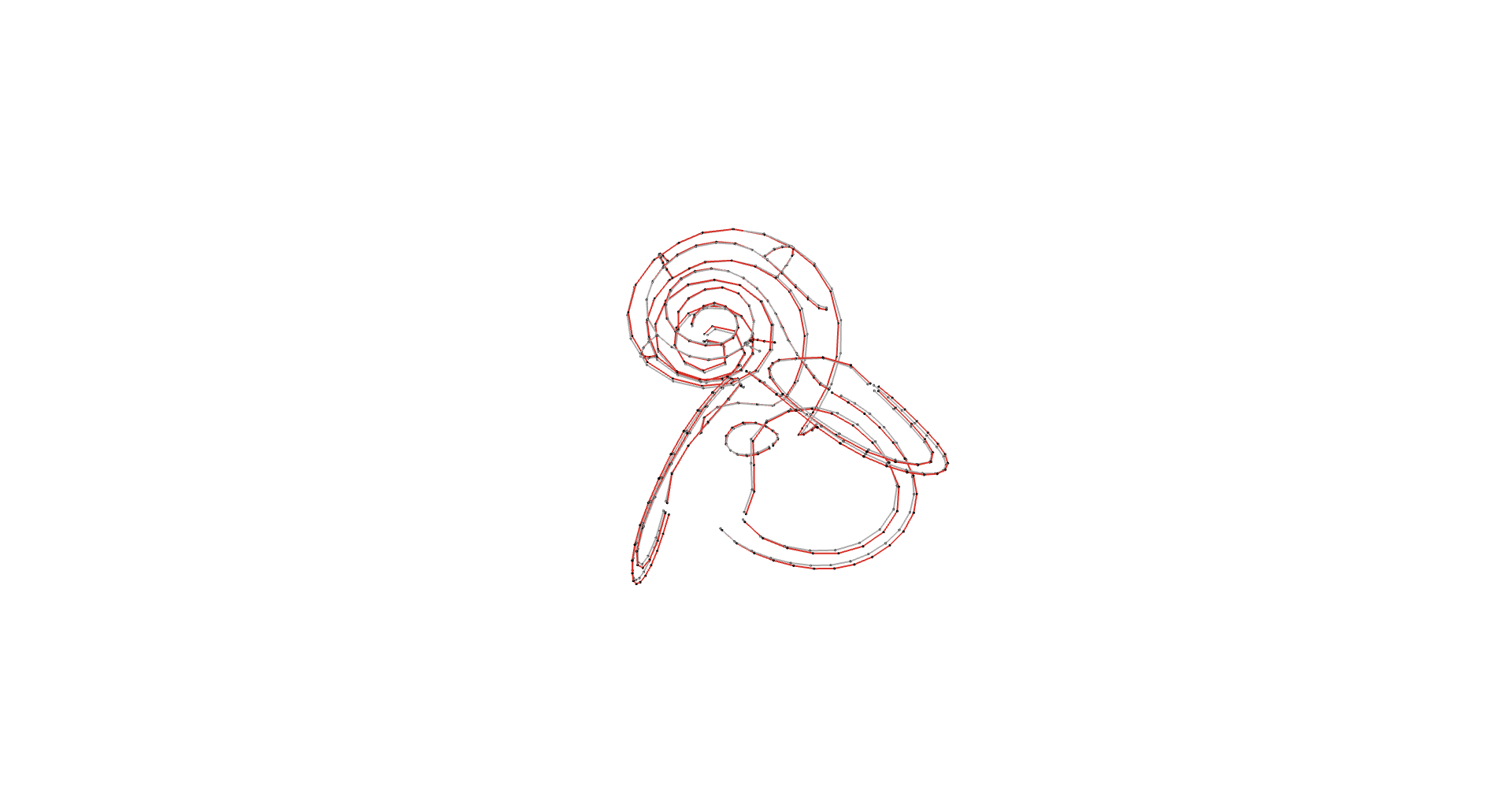

Supplement: Supplementary file 3 — Supplementary Data 1 [file 41467_2022_34656_MOESM3_ESM.zip › Supplementary data_1/Supplementary_material_1-1 Geometric morphometrics/CVA_306/mean_shapes_per_clade_CVA/Bovidae-do.png]

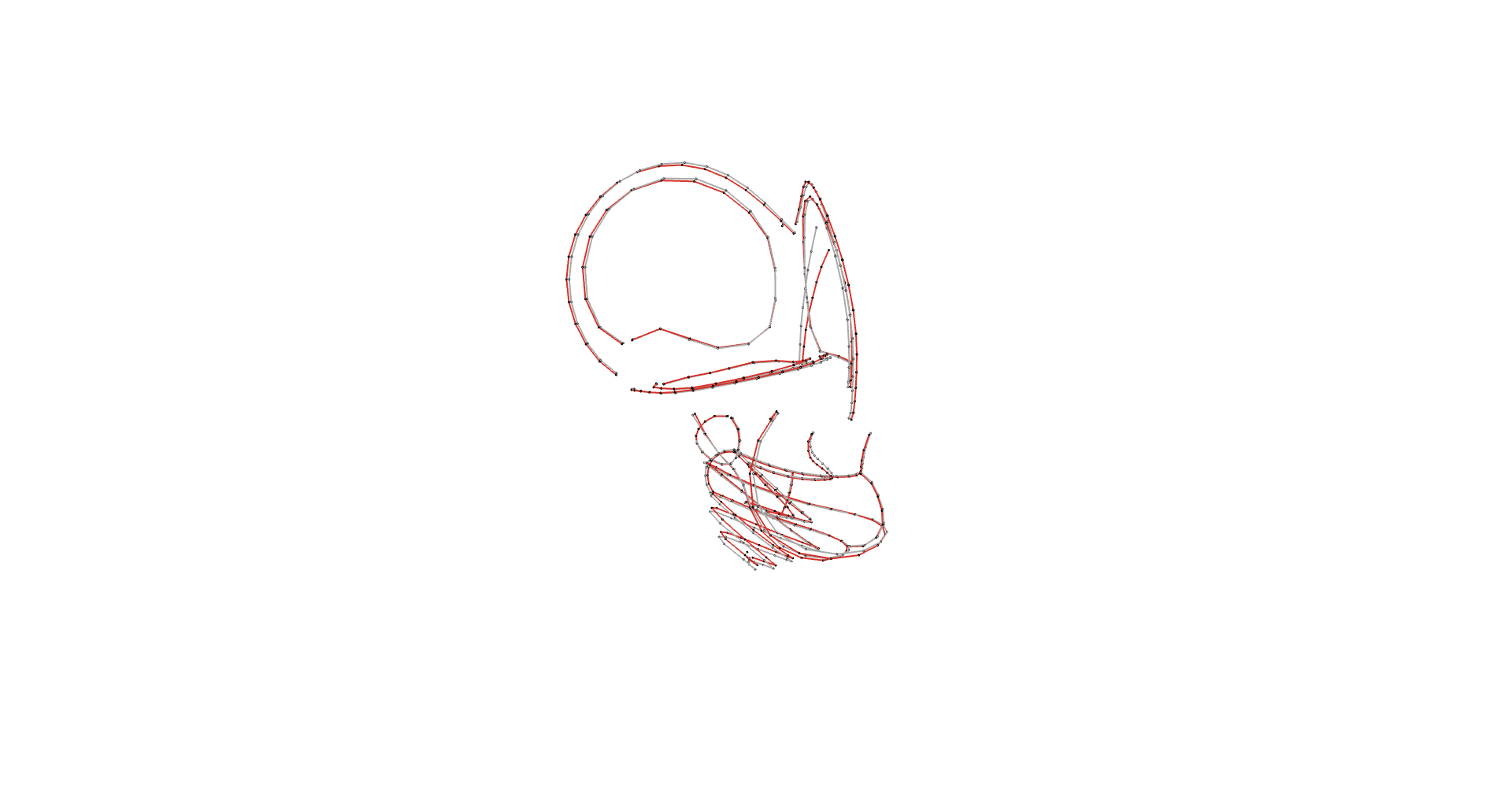

Supplement: Supplementary file 3 — Supplementary Data 1 [file 41467_2022_34656_MOESM3_ESM.zip › Supplementary data_1/Supplementary_material_1-1 Geometric morphometrics/CVA_306/mean_shapes_per_clade_CVA/Bovidae-la.png]

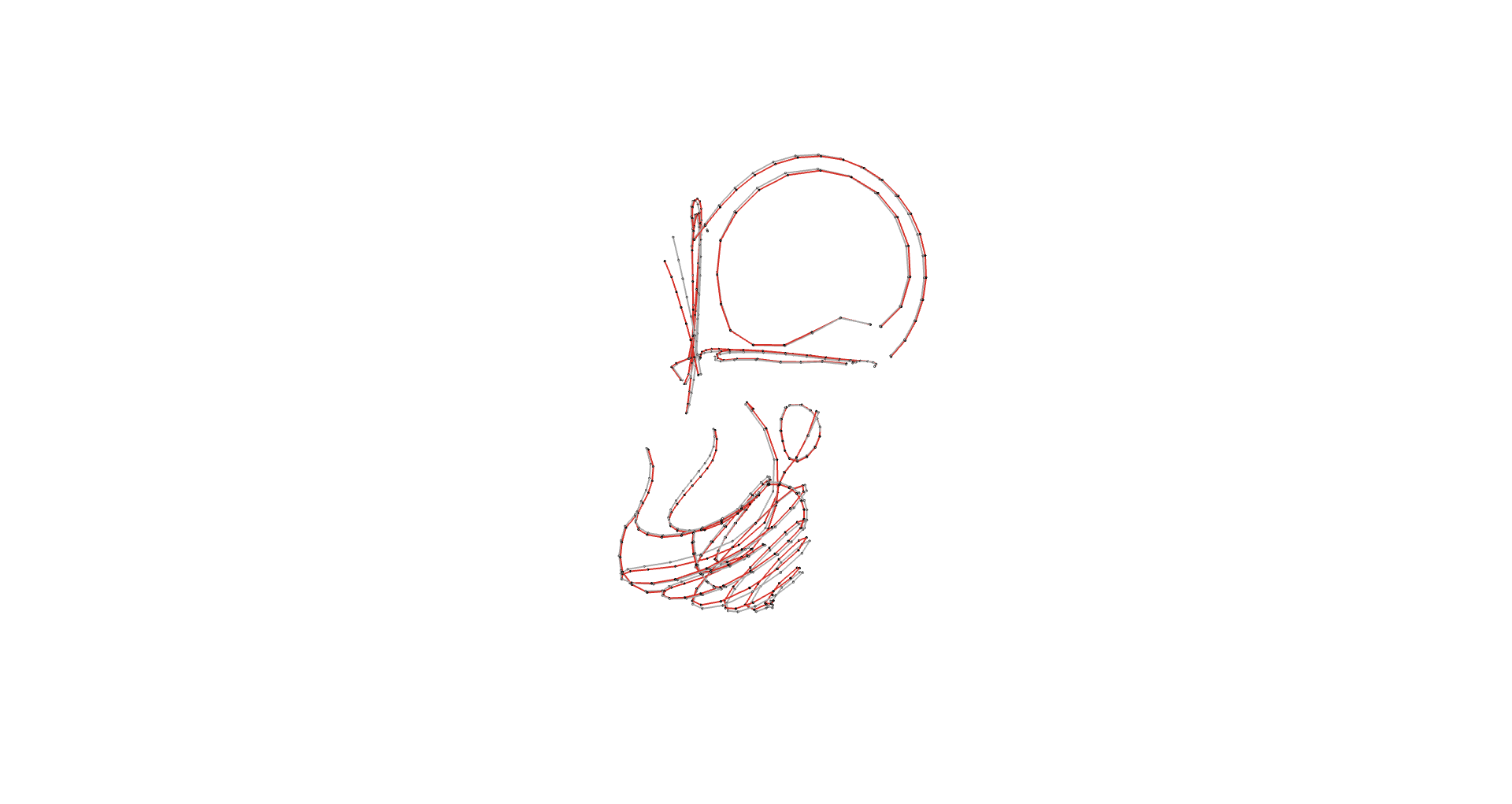

Supplement: Supplementary file 3 — Supplementary Data 1 [file 41467_2022_34656_MOESM3_ESM.zip › Supplementary data_1/Supplementary_material_1-1 Geometric morphometrics/CVA_306/mean_shapes_per_clade_CVA/Bovidae-me.png]

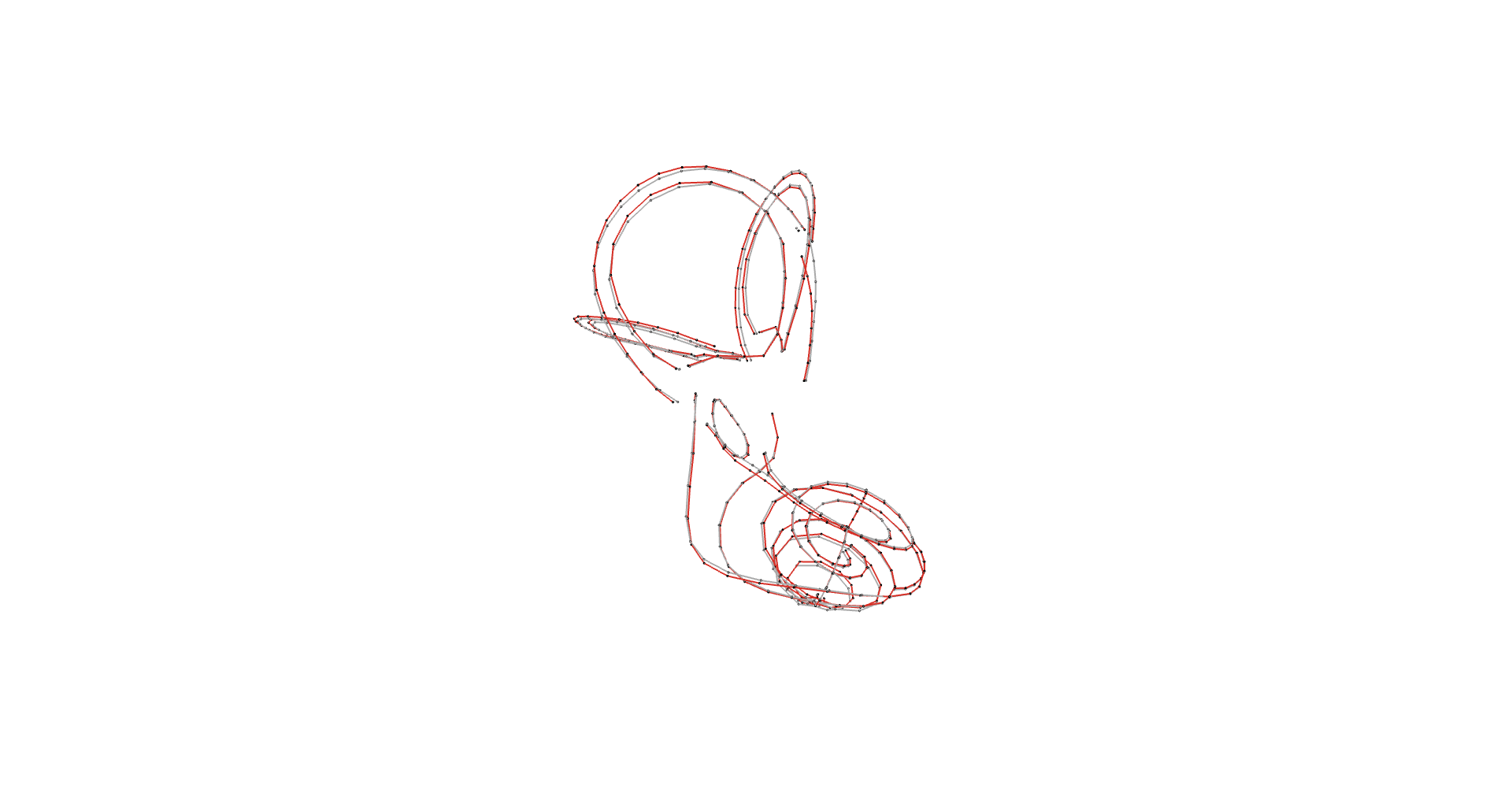

Supplement: Supplementary file 3 — Supplementary Data 1 [file 41467_2022_34656_MOESM3_ESM.zip › Supplementary data_1/Supplementary_material_1-1 Geometric morphometrics/CVA_306/mean_shapes_per_clade_CVA/Bovidae-oc.png]

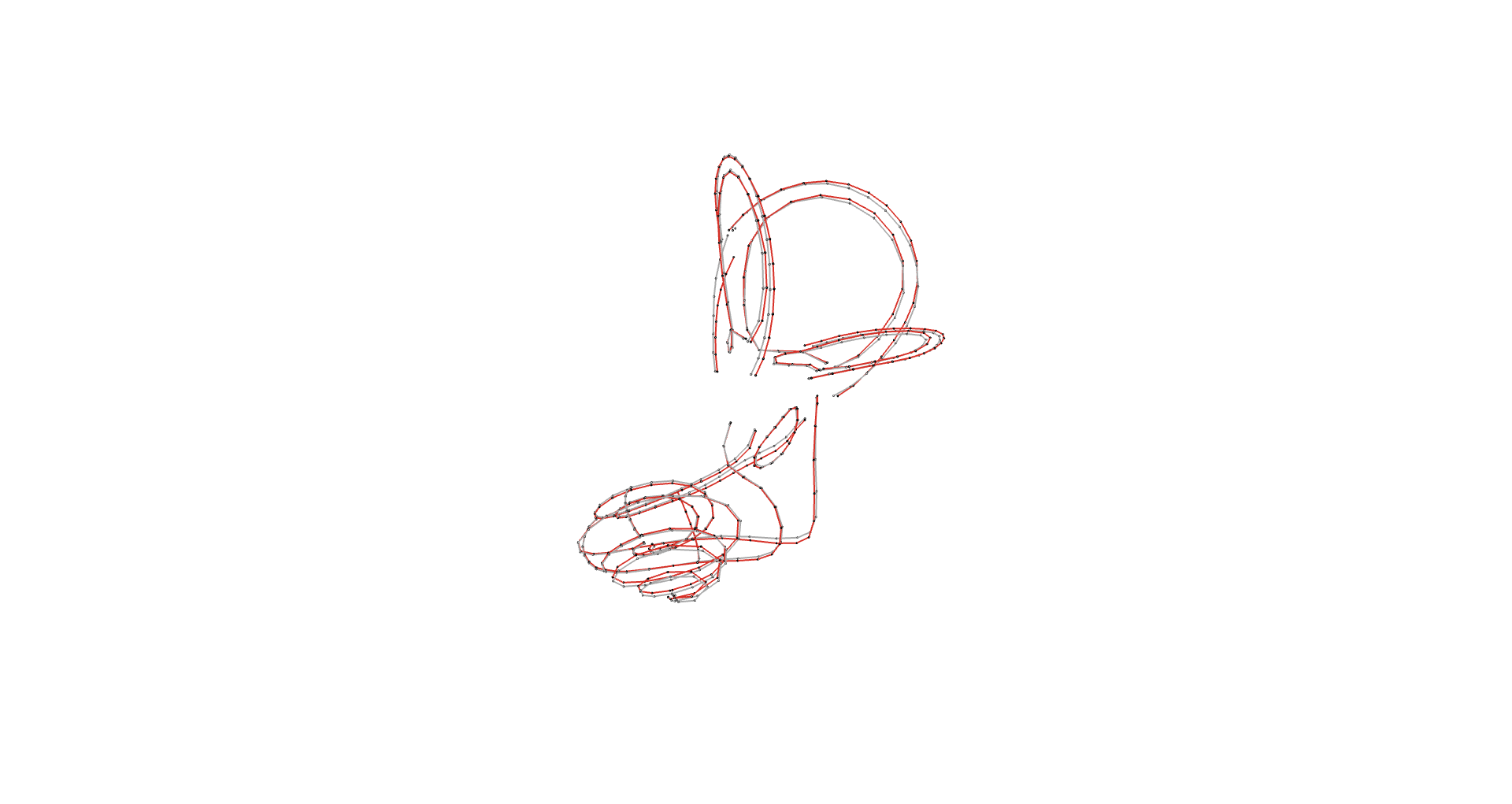

Supplement: Supplementary file 3 — Supplementary Data 1 [file 41467_2022_34656_MOESM3_ESM.zip › Supplementary data_1/Supplementary_material_1-1 Geometric morphometrics/CVA_306/mean_shapes_per_clade_CVA/Bovidae-ro.png]

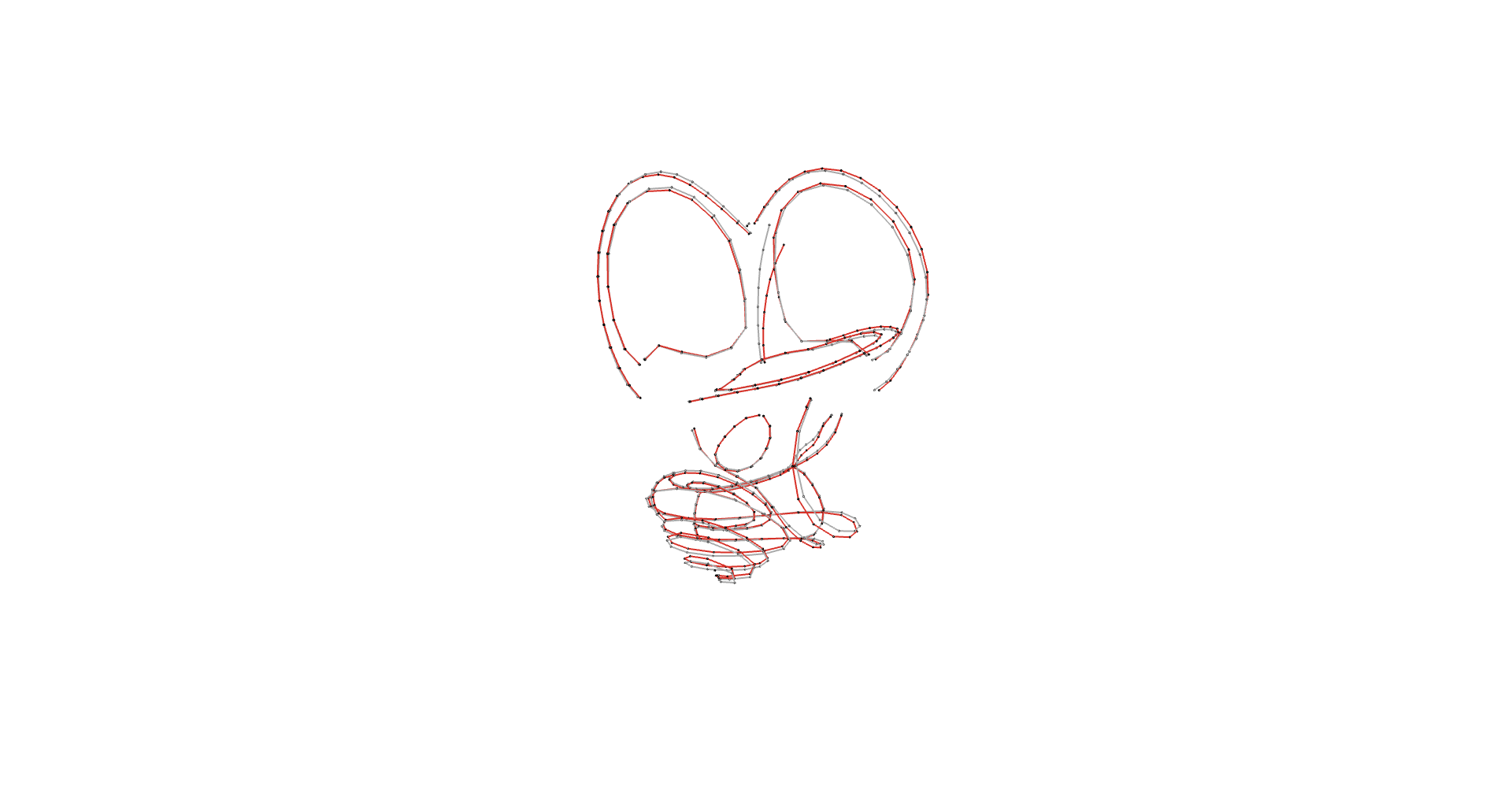

Supplement: Supplementary file 3 — Supplementary Data 1 [file 41467_2022_34656_MOESM3_ESM.zip › Supplementary data_1/Supplementary_material_1-1 Geometric morphometrics/CVA_306/mean_shapes_per_clade_CVA/Bovidae-vl.png]

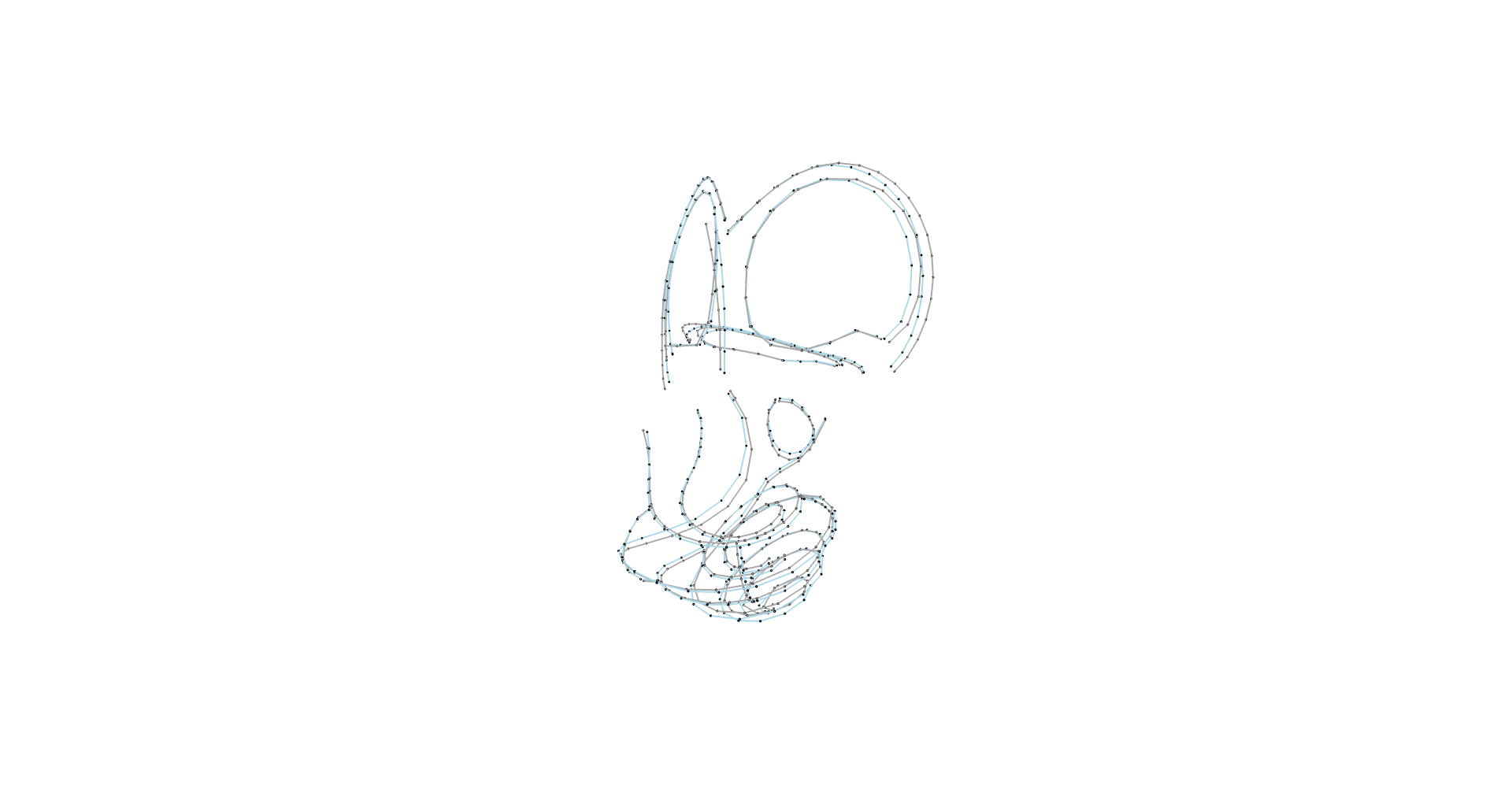

Supplement: Supplementary file 3 — Supplementary Data 1 [file 41467_2022_34656_MOESM3_ESM.zip › Supplementary data_1/Supplementary_material_1-1 Geometric morphometrics/CVA_306/mean_shapes_per_clade_CVA/Cervidae-dl.png]

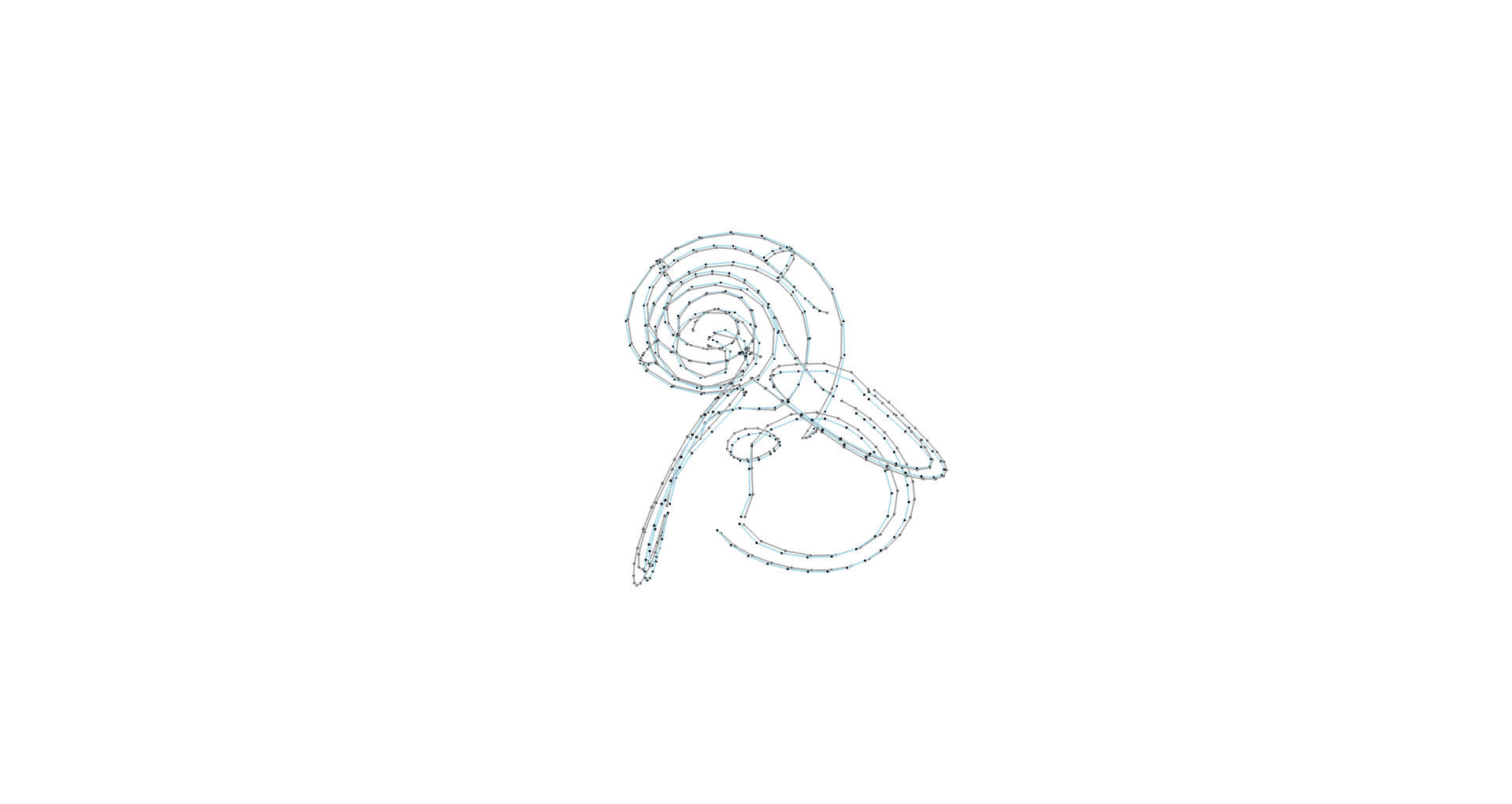

Supplement: Supplementary file 3 — Supplementary Data 1 [file 41467_2022_34656_MOESM3_ESM.zip › Supplementary data_1/Supplementary_material_1-1 Geometric morphometrics/CVA_306/mean_shapes_per_clade_CVA/Cervidae-do.png]

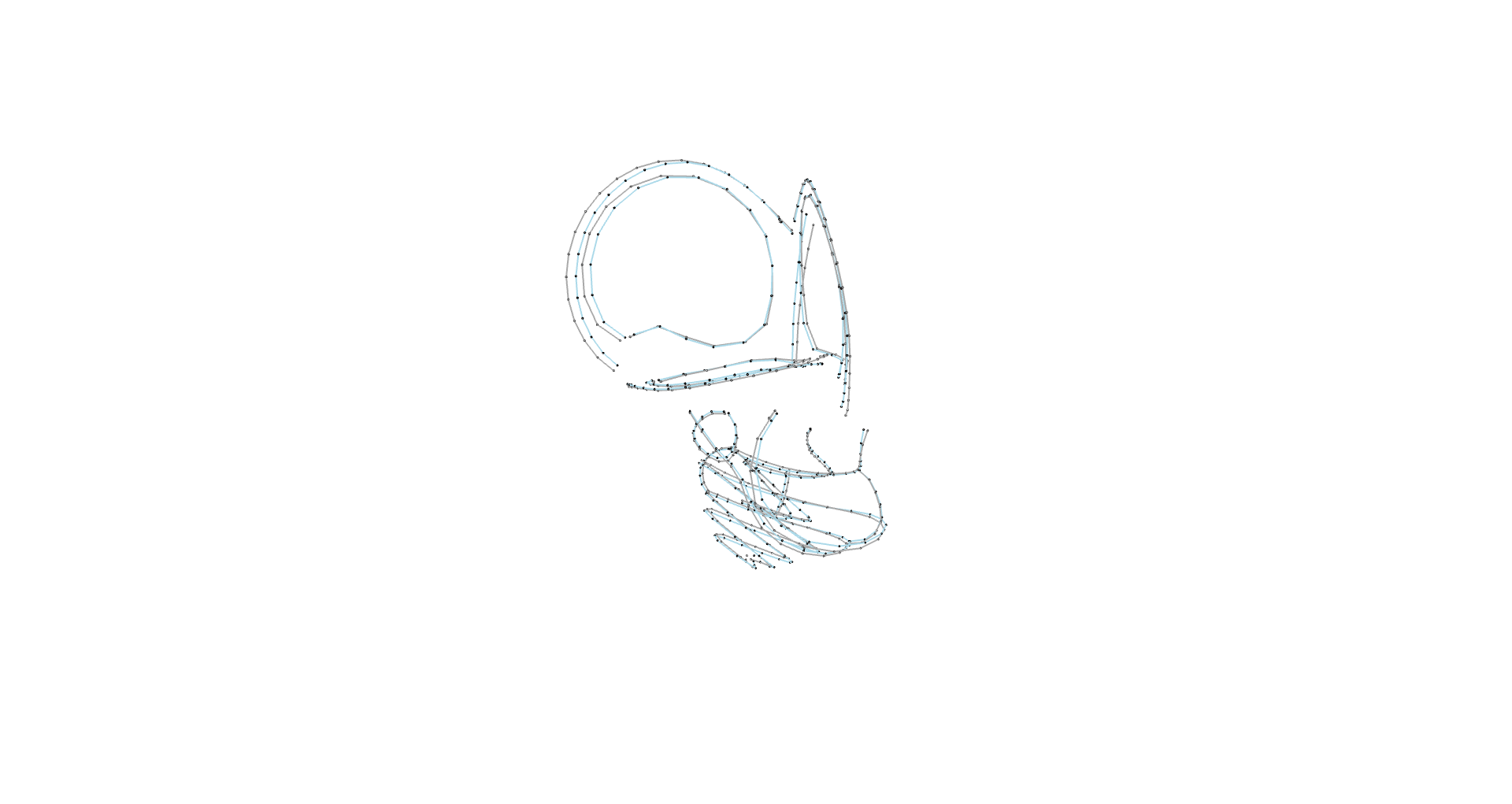

Supplement: Supplementary file 3 — Supplementary Data 1 [file 41467_2022_34656_MOESM3_ESM.zip › Supplementary data_1/Supplementary_material_1-1 Geometric morphometrics/CVA_306/mean_shapes_per_clade_CVA/Cervidae-la.png]

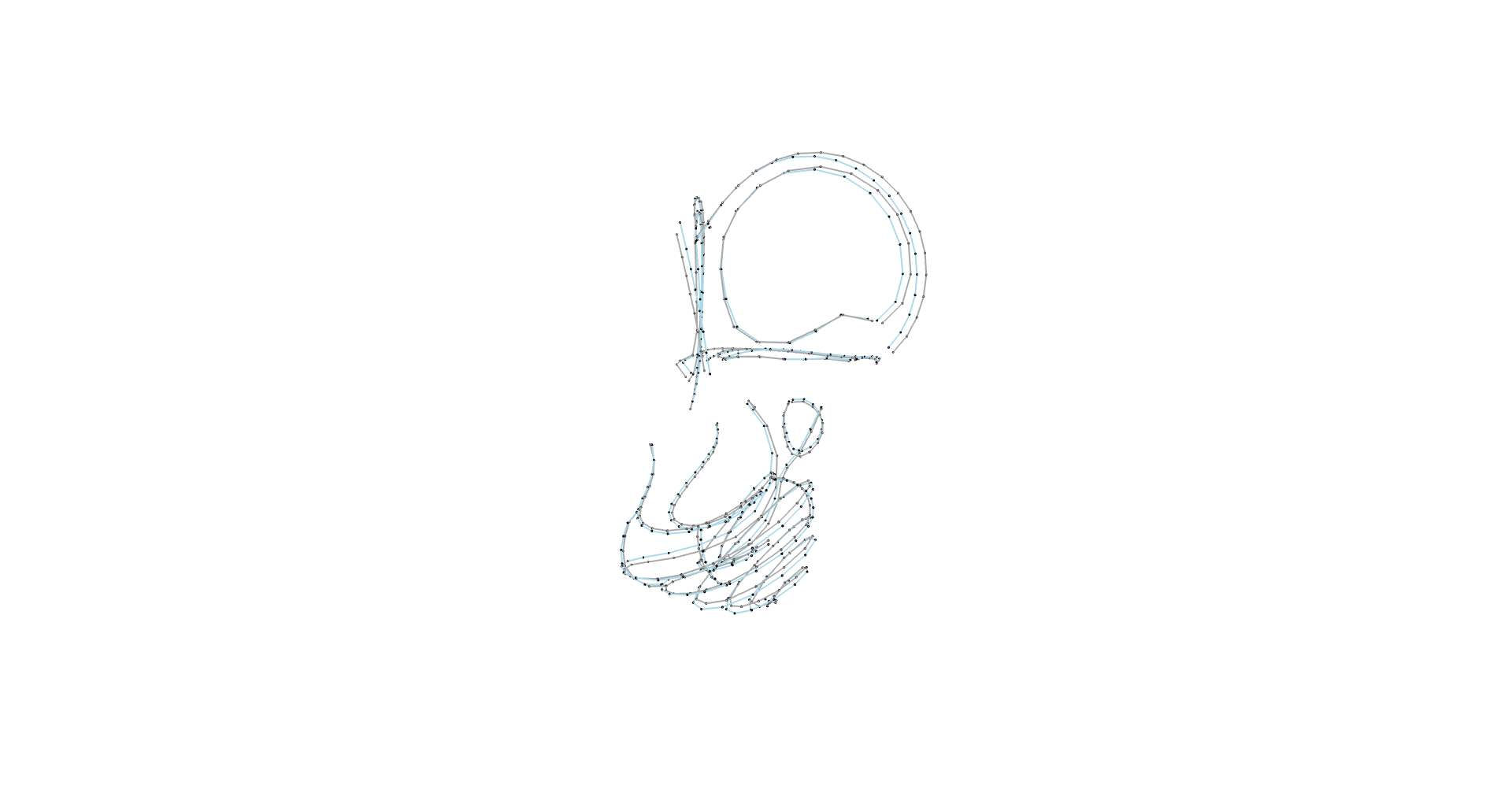

Supplement: Supplementary file 3 — Supplementary Data 1 [file 41467_2022_34656_MOESM3_ESM.zip › Supplementary data_1/Supplementary_material_1-1 Geometric morphometrics/CVA_306/mean_shapes_per_clade_CVA/Cervidae-me.png]

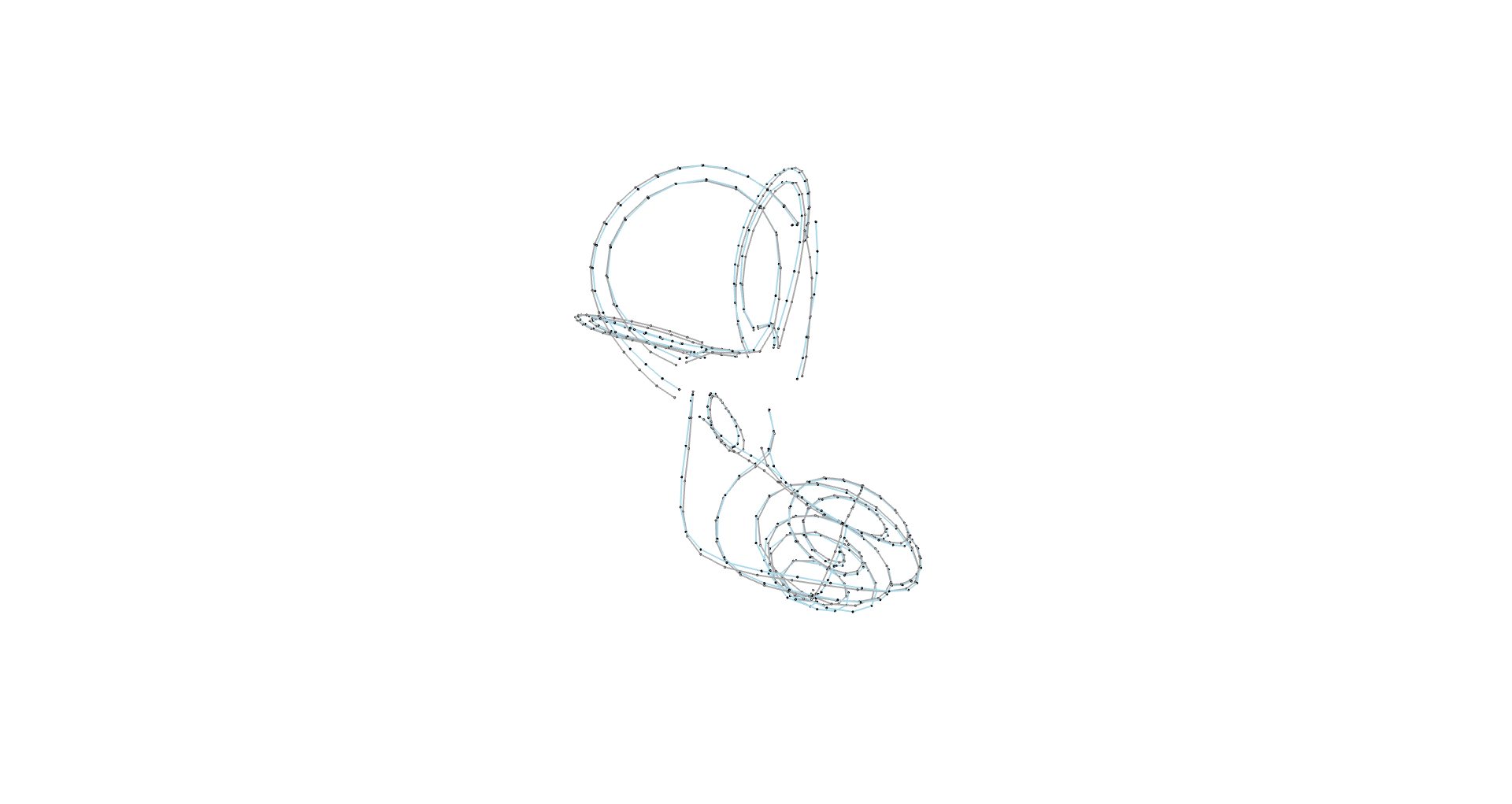

Supplement: Supplementary file 3 — Supplementary Data 1 [file 41467_2022_34656_MOESM3_ESM.zip › Supplementary data_1/Supplementary_material_1-1 Geometric morphometrics/CVA_306/mean_shapes_per_clade_CVA/Cervidae-oc.png]

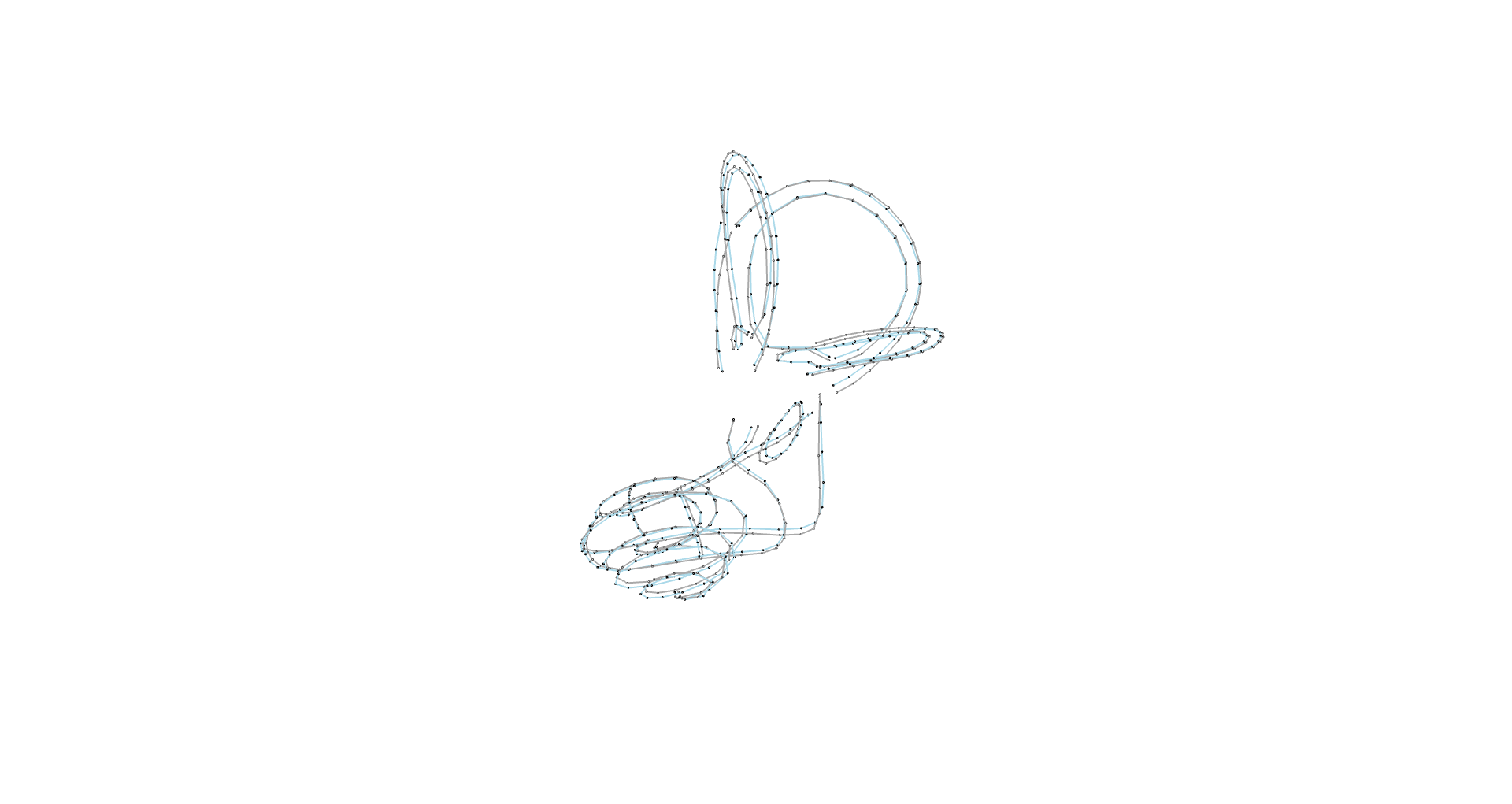

Supplement: Supplementary file 3 — Supplementary Data 1 [file 41467_2022_34656_MOESM3_ESM.zip › Supplementary data_1/Supplementary_material_1-1 Geometric morphometrics/CVA_306/mean_shapes_per_clade_CVA/Cervidae-ro.png]
