# Supplementary material for: Ruminant inner ear shape records 35 million years of neutral evolution
Source: Nat Commun. 2022 Dec 6;13:7222. doi: 10.1038/s41467-022-34656-0 (PMC9726890; doi:10.1038/s41467-022-34656-0)
Supplement: Supplementary file 6 — Reporting Summary [file 41467_2022_34656_MOESM6_ESM.pdf]

nature portfolio

Corresponding author(s): Mennecart Bastien

Last updated by author(s): Oct 7, 2022

Reporting Summary

Nature Portfolio wishes to improve the reproducibility of the work that we publish. This form provides structure for consistency and transparency in reporting. For further information on Nature Portfolio policies, see our Editorial Policies and the Editorial Policy Checklist.

Statistics

For all statistical analyses, confirm that the following items are present in the figure legend, table legend, main text, or Methods section.

n/a	Confirmed	
☐	☒	The exact sample size (*n*) for each experimental group/condition, given as a discrete number and unit of measurement
☐	☒	A statement on whether measurements were taken from distinct samples or whether the same sample was measured repeatedly
☐	☒	The statistical test(s) used AND whether they are one- or two-sided<br>*Only common tests should be described solely by name; describe more complex techniques in the Methods section.*
☐	☒	A description of all covariates tested
☐	☒	A description of any assumptions or corrections, such as tests of normality and adjustment for multiple comparisons
☐	☒	A full description of the statistical parameters including central tendency (e.g. means) or other basic estimates (e.g. regression coefficient) AND variation (e.g. standard deviation) or associated estimates of uncertainty (e.g. confidence intervals)
☐	☒	For null hypothesis testing, the test statistic (e.g. *F*, *t*, *r*) with confidence intervals, effect sizes, degrees of freedom and *P* value noted<br>*Give P values as exact values whenever suitable.*
☒	☐	For Bayesian analysis, information on the choice of priors and Markov chain Monte Carlo settings
☐	☒	For hierarchical and complex designs, identification of the appropriate level for tests and full reporting of outcomes
☒	☐	Estimates of effect sizes (e.g. Cohen's *d*, Pearson's *r*), indicating how they were calculated

Our web collection on statistics for biologists contains articles on many of the points above.

Software and code

Policy information about availability of computer code

Data collection	Avizo Lite 9.0; Landmark Editor 3.6; Mesquite 3.04
Data analysis	R version 4.1.3 (2021-11-01) - "Bird Hippie" - packages: geomorph v4.0.3, RRphylo v2.6.0, Morpho v2.9, phytools v1.0-3, RPANDA v2.0

For manuscripts utilizing custom algorithms or software that are central to the research but not yet described in published literature, software must be made available to editors and reviewers. We strongly encourage code deposition in a community repository (e.g. GitHub). See the Nature Portfolio guidelines for submitting code & software for further information.

Data

Policy information about availability of data

All manuscripts must include a data availability statement. This statement should provide the following information, where applicable:

- Accession codes, unique identifiers, or web links for publicly available datasets
- A description of any restrictions on data availability
- For clinical datasets or third party data, please ensure that the statement adheres to our policy

The datasets generated and analysed during the current study (Landmark data) are available in the Supplementary Data 1. All material from which shape data are generated are housed in museum collections. Details on these collections can be found in Supplementary Data 2. 3D reconstructed models of the bony labyrinths either are published and on open access at MorphoMuseuM (https://morphomuseum.com/) or will be. The not yet published models are available from the corresponding author on reasonable request.

Human research participants

Policy information about studies involving human research participants and Sex and Gender in Research.

Reporting on sex and gender	n/a
Population characteristics	n/a
Recruitment	n/a
Ethics oversight	n/a

Note that full information on the approval of the study protocol must also be provided in the manuscript.

Field-specific reporting

Please select the one below that is the best fit for your research. If you are not sure, read the appropriate sections before making your selection.

☐ Life sciences ☐ Behavioural & social sciences ☒ Ecological, evolutionary & environmental sciences

For a reference copy of the document with all sections, see nature.com/documents/nr-reporting-summary-flat.pdf

Ecological, evolutionary & environmental sciences study design

All studies must disclose on these points even when the disclosure is negative.

Study description	We have studied the evolutionary rates of the bony labyrinth morphology. Shifts in the evolutionary rates have been tested for significance using the function “search.shift” in the R package RRphylo v2.5.0. Since the evolutionary rates fluctuate through time we have tested the significance of the (anti)correlations between the evolutionary rates and the global climatic curves using the function “fit_t_env“ in the R package RPANDA v1.9.
Research sample	We have chosen our sample to encompass a maximum of extinct ruminant taxa from their evolutionary origin as well as a good representativity of the current diversity (half of the species known) based on material from public collections from all around the world (and one specimen that will be soon deposited in a museum which scanned data are open access). We were also interested in intraspecific variation, ontogeny, and time variation. Then, we inspected and scanned petrosal bones, which house the Bony Labyrinth, from 306 specimens representing 191 ruminant species from the early Oligocene (ca. 33 Ma) to the present. The resulting dataset encompasses one of the most extensive study using original data of the ear region. The following ruminant families are included in the analysis, and each pecoran lineage includes representatives of the oldest known genera: Antilocapridae, Bovidae, Cervidae, Dromomerycidae, Girafformorpha, Moschidae, and Tragulidae, as well as several stem Pecora and stem Ruminantia.
Sampling strategy	The project has run during 7 years and we have scanned a maximum of ruminant species during that time from all around the world to get half of the current diversity and ca. 10% of the fossil one, most of the fossil species known being only described based on isolated teeth. Almost all the specimens available (fossil and extent ones) at the Natural History Museum Basel were scanned during this time interval. Scans in other instutions were done to fill phylogenetical and/or temporal gaps. At the end, we have scanned a maximum of specimens to reach a very significant portion of the entire extant diversity of the ruminant, including several taxa for each tribe. Considering the fossil record, we have scanned several species for each extant families including the oldest reprentative and well distributed time markers to have a good idea of the evolutionary history. For stem taxa, we have scanned a maximum of what was available to us. Hence we have a good representativity for species all around the world and from the Paleogene to now enabling us to encompass most of the ruminant evolutionary history.
Data collection	All the contributors of the manuscript contributed to the data collection. After selecting specimens from the institutions, the petrosal bones were scanned using high resolution hard X-ray computed tomography from the following institutions: Biomaterials Science Center of the University of Basel (CH), nanotom® m (phoenix|x-ray, GE Sensing & Inspection Technologies); Department of Anthropology of the University of Zurich (CH), Nikon XTH 225 ST; Department of Geosciences of the University of Fribourg (CH), Bruker Skyscan 2211; Plateforme d’Accès Scientifique à la Tomographie à Rayon X (AST-RX) of the Muséum national d’Histoire naturelle in Paris (FR), GE Sensing and Inspection Technologies phoenix X-ray v|tome|x L240-180; Plateforme Montpellier Ressources Imagerie (MRI) of the University of Montpellier 2 (FR), Skyscan 1076 in vivo; Microscopy and Imaging Facility (MIF) of the American Museum of Natural History (USA), GE Phoenix Vtome x L240; Nanoscale Research Facility of the University of Florida (USA), Phoenix v|tome|x M (GE’s Measurement & Control business; Staatliche Naturwissenschaftliche Sammlungen Bayerns (GE), nanotom® m (phoenix|x-ray, GE Sensing & Inspection Technologies); Staatliches Museum für Naturkunde Stuttgart (GE), Bruker Skyscan 1272; The Natural History Museum London of United Kingdom (UK), Nikon Metrology HMX-ST 225; Institute of Vertebrate Paleontology and Paleoanthropology, Chinese Academy of Sciences (CHINA), GE v|tome|x m300&180 (GE Measurement & Control, Wuntsdorf, Germany); Museo Nacional de Ciencias Naturales-CSIC (ES), NIKON CT-SCAN- XT H-160; University Museum, University of Tokyo (JA), TX225-ACTIS (TESCO Corporation) and ScanXmate-B100TSS110 (Comscantecno Co. Ltd.). Pixel resolution varies between 15 and 60 µm. During each acquisition, 1440 equiangular radiographs were taken over 360° using an adjustable range of accelerating voltage of 90 kV and a beam current of 200 µA for recent material to 180 kV with a beam current of 30 µA for fossils. Segmentation was performed with AVIZO® 9.0 Lite software (Visualization Sciences Group) mostly by Mennecart B., Costeur L., and Laurens F. The landmarking of the specimens has been done by Mennecart B. using Landmark Editor 3.6, the landmarks that are the primary data used in this article. Dziomber L. did the resampling procedure of the landmarks using R version 4.1.3.

Timing and spatial scale	The scanning project started in 03.2013 and we have scanned specimens for this specific project until 06.2019. Specimens from the Natural History Museum Basel were scanned during between 2014-2016 and 2018-2019 at the Biomaterials Science Center Basel in the frame of an interinstitute collaboration. Other specimens have been scanned at various institutes on a global scale based on punctual projects and collaborations (e.g. New York, Gainesville, London, Paris, Tokyo, Beijing).
Data exclusions	No data has been excluded.
Reproducibility	All the landmarks are provided with the code so that everybody can reuse the dataset and the script.
Randomization	Their is no randomisation since we know the taxonomy and phylogeny of the specimens.
Blinding	There was no blinding since it was not a test based on experimentation.

Did the study involve field work? ☐ Yes ☒ No

Reporting for specific materials, systems and methods

We require information from authors about some types of materials, experimental systems and methods used in many studies. Here, indicate whether each material, system or method listed is relevant to your study. If you are not sure if a list item applies to your research, read the appropriate section before selecting a response.

Materials & experimental systems

n/a	Involved in the study
☒	☐ Antibodies
☒	☐ Eukaryotic cell lines
☐	☒ Palaeontology and archaeology
☒	☐ Animals and other organisms
☒	☐ Clinical data
☒	☐ Dual use research of concern

Methods

n/a	Involved in the study
☒	☐ ChIP-seq
☒	☐ Flow cytometry
☒	☐ MRI-based neuroimaging

Palaeontology and Archaeology

Specimen provenance	Anabo Koma (Dj); Arrisdrift (Na); Artenay (Fr); Barstow (USA); Broken Hill (Za); Chilleurs aux bois (Fr); Chiro 10c (It); Chiro 26 (It); Chiro d4 (It); Chiro13 (It); Coderet (Fr); Contres (Fr); Crevillent (Sp); Cueva de Son Muleta (SP); Darbys Cave (A&B); Etouaires (Fr); Fort Meade Mine (USA); Gajambira (Ja); Gargano (It); Gebel Zelten (Li); Gohezu Cave (Ja); Gordon Creek (USA); Haile 7C (USA); Hamalagai (Ch); Holow Horn Bear Quarry (USA); IKH-A-1 (Mo); Inglis 1A (USA); Itardie (Fr); Kinkazan Island (Ja); Kohfidisch (Au); La Grive (Fr); Langenau (Ge); Long quarry (USA); Los Valles de Fuentiduena (Sp); Loss acres racetrack (USA); Ma Chi Lien Kou (Ch); Michigan (USA); Millenium Park (USA); Minas Gerais (Br); Missouri (USA); Möhren 13 (Ge); Monte Olivola (It); Montpellier (Fr); Montréal du Gers (Fr); Murs (Fr); Nasario 4 (It); Olduvai (Ta); Pai Tao Tsun (Ch); Papago Springs cave (USA); Pawnee Buttes (USA); Perin island (In); Perpignan (Fr); Quercy (Fr); Rauscheröd (Ge); Roccaneyra (It); Ronzon (Fr); Saint Gérand le Puy (Fr); Saint Vallier (Fr); Sandelzhausen (Ge); Sansan (Fr); Senèze (Fr); Sioux County (USA); Snake River (USA); Soblay (Fr); Steinheim am Albuch (Ge); Tabün (Is); TGR-C2 (Mo); TGW-A (Mo); Thomas farm (USA); Thomson quarry (USA); Toril-3 (Sp); UNCH-A (Mo); Upper Rosebud (USA); Val d'Arno (It); Val di Chiana (It); Westown Naul (Ir); Wintershof West (Ge); Xiatamaicun (Ch); Xizuizigou (Ch); Xmax quarry (USA). Agreements with currating museums was done to work on this material. Since no material has been excavated in the frame of this study, no field permit was done.
Specimen deposition	Adrien de Perthuis Collection; American Museum of Natural History; Arrisdrift, Namibia; Bayerische Staatssammlung für Paläontologie und Geologie, Munich; Institute of Vertebrate Paleontology and Paleoanthropology, Beijing; Musée Confluences Lyon; Musée de Djibouti; Museo Nacional de Ciencias Naturales Madrid; Museu de Geologia de la Universitat de Valencia; Muséum d'histoire naturelle, Toulouse; Muséum national d'Histoire naturelle, Paris; Natural History Museum, London; Naturhistorisches Museum Basel; Naturhistorisches Museum Wien; Netherland Centre for Biodiversity Leiden; Okinawa Prefectural Museum & Art Museum; Parc Naturel Régional du Luberon; Staatliches Museum für Naturkunde Stuttgart; Steinheim_MeteorkraterMuseum; The University Museum, The University of Tokyo; Université Claude Bernard, Lyon 1; Université Montpellier 2; University of Florida, Gainesville.
Dating methods	Ages were provided by literature

☒ Tick this box to confirm that the raw and calibrated dates are available in the paper or in Supplementary Information.

Ethics oversight	No ethical approval or guidance was required because the specimens belong to official collections and we got the agreement to study it. The study does involve partial destruction of the specimens, on a very limited basis (less than 2% of the material was extracted physically from skulls prior to scanning).

Note that full information on the approval of the study protocol must also be provided in the manuscript.
