# Supplementary material for: Ruminant inner ear shape records 35 million years of neutral evolution
Source: Nat Commun. 2022 Dec 6;13:7222. doi: 10.1038/s41467-022-34656-0 (PMC9726890; doi:10.1038/s41467-022-34656-0)
Supplement: Supplementary file 3 — Supplementary Data 1 [file 41467_2022_34656_MOESM3_ESM.zip › Supplementary data_1/Supplementary_material_1-2 RRphylo/PCA/PCA_evorates_no_ancestral_states.pdf]

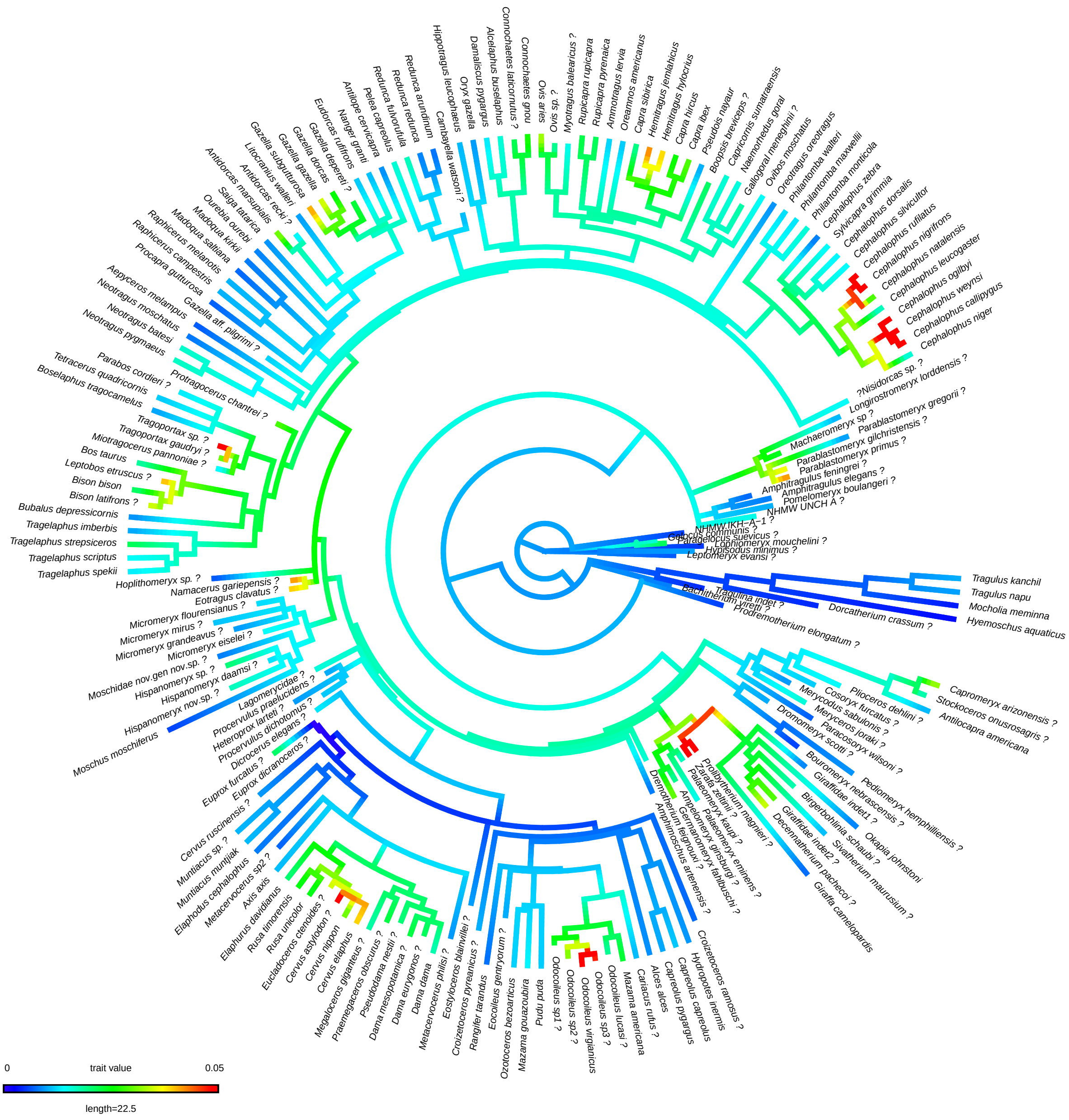
Tragulus kanchil
Tragulus napu
Mocholia meminna
Hyemoschus aquaticus
Dorcatherium crassum ?
Prodremotherium elongatum ?
Bachitherium viretti ?
Tragulina indet ?
Leptomeryx evansi ?
Hypisodus minimus ?
Lophiomeryx mouchelini ?
Paradelocus suevicus ?
Gelocus communis ?
NHMW.IKH−A−1 ?
NHMW UNCH A ?
Pomelomeryx boulangeri ?
Amphitragulus elegans ?
Amphitragulus feningrei ?
Parablastomeryx primus ?
Parablastomeryx gilchristensis ?
Machaeromeryx sp ?
Parablastomeryx gregorii ?
Longirostromeryx lorddensis ?
?Nisidorcas sp. ?
Cephalophus niger
Cephalophus callipygus
Cephalophus weynsi
Cephalophus ogilbyi
Cephalophus leucogaster
Cephalophus natalensis
Cephalophus nigrifrons
Cephalophus rufilatus
Cephalophus silvicultor
Cephalophus dorsalis
Sylvicapra grimmia
Cephalophus zebra
Philantomba monticola
Philantomba maxwellii
Philantomba walteri
Oreotragus oreotragus
Ovibos moschatus
Gallogoral meneghinii ?
Naemorhedus goral
Capricornis sumatraensis
Boopsis breviceps ?
Pseudois nayaur
Capra ibex
Capra hircus
Hemitragus hylocrius
Hemitragus jemlahicus
Capra sibirica
Oreamnos americanus
Ammotragus lervia
Rupicapra pyrenaica
Rupicapra rupicapra
Myotragus balearicus ?
Ovis sp. ?
Ovis aries
Connochaetes gnou
Connochaetes laticornutus ?
Alcelaphus buselaphus
Damaliscus pygargus
Oryx gazella
Hippotragus leucophaeus
Cambayella watsoni ?
Redunca arundinum
Redunca redunca
Redunca fulvorufula
Pelea capreolus
Antilope cervicapra
Nanger granti
Eudorcas rufifrons
Gazella depereti ?
Gazella dorcas
Gazella gazella
Gazella subgutturosa
Litocranius walleri
Antidorcas recki ?
Antidorcas marsupialis
Saiga tatarica
Ourebia ourebi
Madoqua kirkii
Madoqua saltiana
Raphicerus melanotis
Raphicerus campestris
Procapra gutturosa
Gazella aff. pilgrimi ?
Aepyceros melampus
Neotragus moschatus
Neotragus batesi
Neotragus pygmaeus
Protragocerus chantrei ?
Parabos cordieri ?
Tetracerus quadricornis
Boselaphus tragocamelus
Tragoportax sp. ?
Tragoportax gaudryi ?
Miotragocerus pannoniae ?
Bos taurus
Leptobos etruscus ?
Bison bison
Bison latifrons ?
Bubalus depressicornis
Tragelaphus imberbis
Tragelaphus strepsiceros
Tragelaphus scriptus
Tragelaphus spekii
Hoplithomeryx sp. ?
Namacerus gariepensis ?
Eotragus clavatus ?
Micromeryx flourensianus ?
Micromeryx mirus ?
Micromeryx grandeavus ?
Micromeryx eiselei ?
Moschidae nov.gen nov.sp. ?
Hispanomeryx sp. ?
Hispanomeryx daamsi ?
Hispanomeryx nov.sp. ?
Moschus moschiferus
Lagomerycidae ?
Procervulus praelucidens ?
Heteroprox larteti ?
Procervulus dichotomus ?
Dicrocerus elegans ?
Euprox furcatus ?
Euprox dicranoceros ?
Cervus ruscinensis ?
Muntiacus sp. ?
Muntiacus muntjak
Elaphodus cephalophus
Metacervocerus sp2 ?
Axis axis
Elaphurus davidianus
Rusa timorensis
Rusa unicolor
Eucladoceros ctenoides ?
Cervus astylodon ?
Cervus nippon
Cervus elaphus
Megaloceros giganteus ?
Praemegaceros obscurus ?
Pseudodama nestii ?
Dama mesopotamica ?
Dama eurygonos ?
Dama dama
Metacervocerus philisi ?
Eostyloceros blainvillei ?
Croizetoceros pyreanicus ?
Rangifer tarandus
Eocoileus gentryorum ?
Ozotoceros bezoarticus
Mazama gouazoubira
Pudu puda
Odocoileus sp1 ?
Odocoileus sp2 ?
Odocoileus virginianus
Odocoileus sp3 ?
Odocoileus lucasi ?
Mazama americana
Cariacus rufus ?
Alces alces
Capreolus pygargus
Capreolus capreolus
Hydropotes inermis
Croizetoceros ramosus ?
Amphimoschus artenensis ?
Dremotherium feignouxi ?
Ampelomeryx ginsburgi ?
Germanomeryx fahlbuschi ?
Palaeomeryx eminens ?
Palaeomeryx kaupi ?
Zarafa zelteni ?
Prolibytherium magnieri ?
Giraffa camelopardis
Decennatherium pachecoi ?
Giraffidae indet2 ?
Sivatherium maurusium ?
Birgerbohlinia schaubi ?
Okapia johnstoni
Giraffidae indet1 ?
Bouromeryx nebrascensis ?
Pediomeryx hemphilliensis ?
Dromomeryx scotti ?
Paracosoryx wilsoni ?
Meryceros joraki ?
Merycodus sabulonis ?
Cosoryx furcatus ?
Plioceros dehlini ?
Antilocapra americana
Stockoceros onusrosagris ?
Capromeryx arizonensis ?
0
trait value
0.05
length=22.5
