# Supplementary material for: Ruminant inner ear shape records 35 million years of neutral evolution
Source: Nat Commun. 2022 Dec 6;13:7222. doi: 10.1038/s41467-022-34656-0 (PMC9726890; doi:10.1038/s41467-022-34656-0)
Supplement: Supplementary file 3 — Supplementary Data 1 [file 41467_2022_34656_MOESM3_ESM.zip › Supplementary data_1/Supplementary_material_1-3 RPANDA/Rpanda_PCA/RPANDA_PCA_VS_temperature_Pecora-Tragulina.pdf]

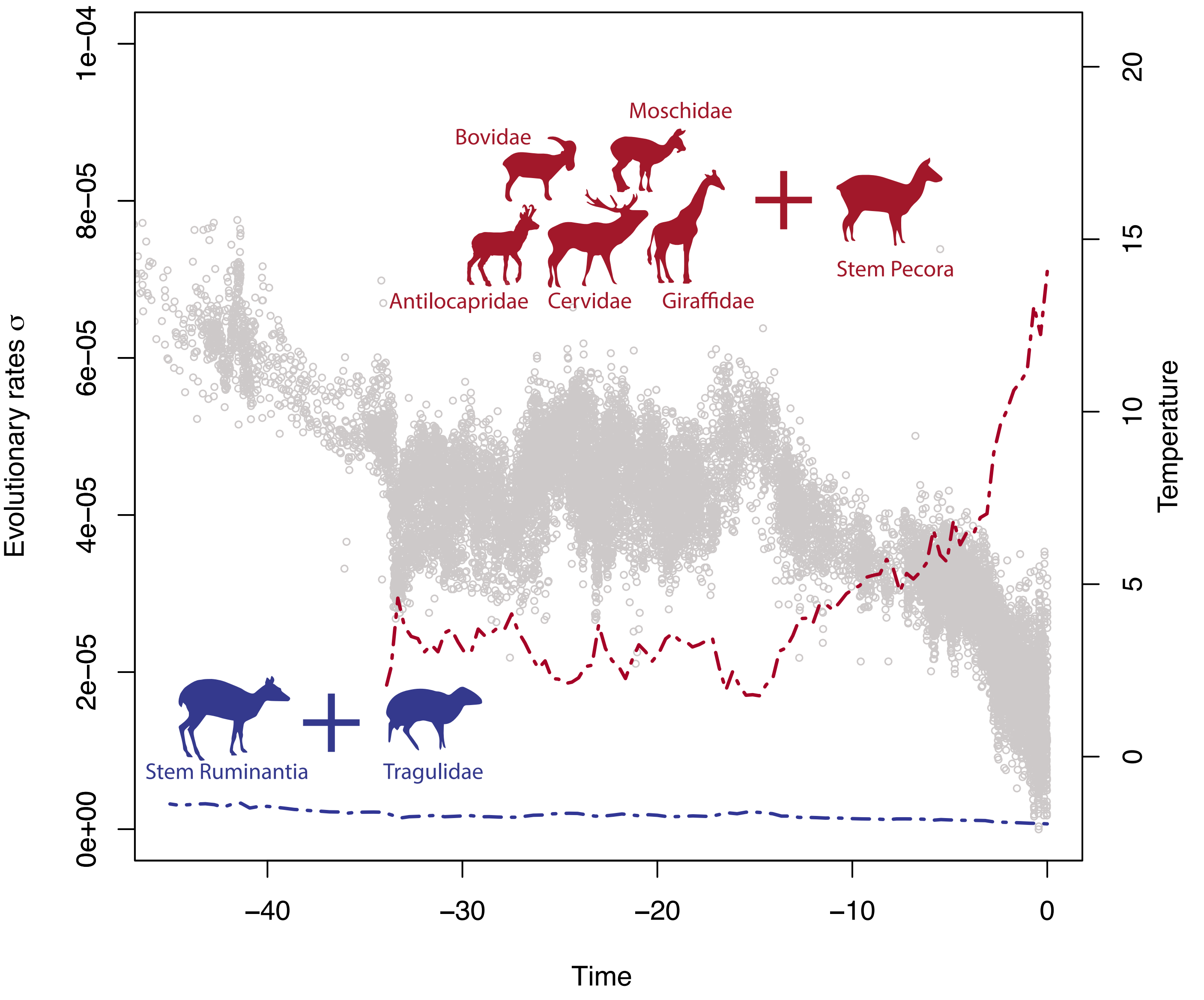
Moschidae
Bovidae
Antilocapridae
Cervidae
Giraffidae
Stem Pecora
Stem Ruminantia
Tragulidae
Evolutionary rates σ
Temperature
Time
1e−04
8e−05
6e−05
4e−05
2e−05
0e+00
20
15
10
5
0
−40
−30
−20
−10
0
