# Supplementary material for: Ruminant inner ear shape records 35 million years of neutral evolution
Source: Nat Commun. 2022 Dec 6;13:7222. doi: 10.1038/s41467-022-34656-0 (PMC9726890; doi:10.1038/s41467-022-34656-0)
Supplement: Supplementary file 3 — Supplementary Data 1 [file 41467_2022_34656_MOESM3_ESM.zip › Supplementary data_1/Supplementary_material_1-4 Raw dataset and R code/Data_191 species/LEGEND_ALL_GRAPHS.pdf]

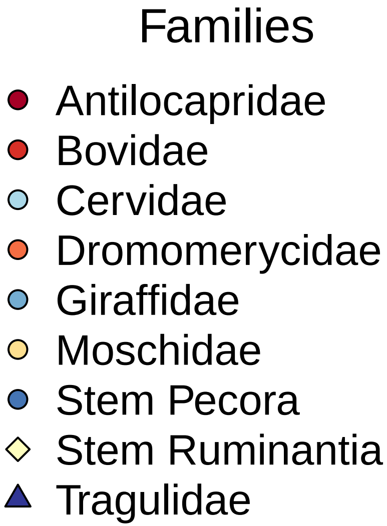
Families
Antilocapridae
Bovidae
Cervidae
Dromomerycidae
Giraffidae
Moschidae
Stem Pecora
Stem Ruminantia
Tragulidae
